# Structure-Guided Design of ISOX-DUAL-Based Degraders Targeting BRD4 and CBP/EP300: A Case of Degrader Collapse

**DOI:** 10.1021/acs.jmedchem.5c00395

**Published:** 2025-04-17

**Authors:** Anthony K. Edmonds, Dimitrios-Ilias Balourdas, Graham P. Marsh, Robert Felix, Bradley Brasher, Jeff Cooper, Cari Graber-Feesl, Madhu Kollareddy, Karim Malik, Helen Stewart, Timothy J. T. Chevassut, Ella Lineham, Simon Morley, Oleg Fedorov, James Bennett, Mohan B. Rajasekaran, Samuel Ojeda, Drew A. Harrison, Christopher J. Ott, Andreas C. Joerger, Hannah J. Maple, John Spencer

**Affiliations:** † Chemistry Department, School of Life Sciences, University of Sussex, Brighton BN1 9QJ, U.K.; ‡ Bio-Techne (Tocris), The Watkins Building, Atlantic Road, Avonmouth, Bristol BS11 9QD, U.K.; § Institute of Pharmaceutical Chemistry, Goethe University, Max-von-Laue-Str. 9, 60438 Frankfurt am Main, Germany; ∥ Structural Genomics Consortium (SGC), Buchmann Institute for Life Sciences, Max-von-Laue-Str. 15, 60438 Frankfurt am Main, Germany; ⊥ Bio-Techne (R&D Systems), 614 McKinley Place NE, Minneapolis 55413, United States; # Cancer Epigenetics Laboratory, School of Cellular and Molecular Medicine, University of Bristol, Bristol BS8 1TD, U.K.; ∇ Brighton and Sussex Medical School, University of Sussex, Brighton BN1 9PS, U.K.; ○ Biochemistry Department, School of Life Sciences, University of Sussex, Brighton BN1 9QQ, U.K.; ◆ Centre for Medicines Discovery, Nuffield Department of Medicine, NDM Research building, Old Road Campus, Oxford OX3 7FZ, U.K.; ¶ Sussex Drug Discovery Centre, School of Life Sciences, University of Sussex, Brighton BN1 9QJ, U.K.; †† Krantz Family Center for Cancer Research, Massachusetts General Hospital, Charlestown, Massachusetts 02129, United States; ‡‡ Department of Medicine, Harvard Medical School, Boston, Massachusetts 02115, United States

## Abstract

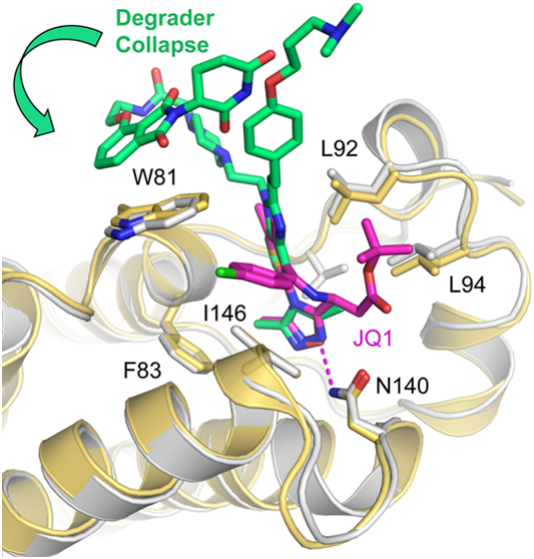

Degraders with dual activity against BRD4 and CBP/EP300 were designed. A structure-guided design approach was taken to assess and test potential exit vectors on the dual BRD4 and CBP/EP300 inhibitor, ISOX-DUAL. Candidate degrader panels revealed that VHL-recruiting moieties could mediate dose-responsive ubiquitination of BRD4. A panel of CRBN-recruiting thalidomide-based degraders was unable to induce ubiquitination or degradation of target proteins. High-resolution protein cocrystal structures revealed an unexpected interaction between the thalidomide moiety and Trp81 on the first bromodomain of BRD4. The inability to form a ternary complex provides a potential rationale for the lack of degrader activity with these compounds, some of which have remarkable affinities close to those of (+)-JQ1, as low as 65 nM in a biochemical assay, vs 1.5 μM for their POI ligand, ISOX-DUAL. Such a “degrader collapse” may represent an under-reported mechanism by which some putative degrader molecules are inactive with respect to target protein degradation.

## Introduction

The *MYC* proto-oncogene is a master regulator of transcription with a central role in cancer cell pathophysiology. The expression of *MYC* is tightly controlled in healthy cells but, in up to 70% of human cancers, *MYC* expression is elevated or dysregulated.^1,2^ Pharmacological inactivation of the c-Myc oncoprotein is therefore considered an attractive approach as a therapeutic strategy for cancer, with multiple lines of evidence suggesting that *MYC* inactivation leads to tumor regression.^3−5^ Directly targeting the c-Myc oncoprotein as a therapeutic strategy is challenging, however, due to a lack of a binding pocket amenable to small-molecule inhibitor development.^6^

Alternative strategies to target c-Myc indirectly have therefore been pursued, through inhibition or degradation of upstream and downstream proteins including BET bromodomains^7,8^ and the immune cell-specific transcription factor, interferon regulatory factor 4 (IRF4).^9^ Knockdown of IRF4 is toxic in multiple myeloma (MM) cell lines, while pharmacological inhibition of the bromodomain histone acetyltransferases CBP/EP300 is reported to lead to direct transcriptional suppression of IRF4 and concomitant reduction in *MYC* expression.^10,11^ More recent work suggests that MM cell death following treatment with CBP/EP300 inhibitors is not mediated by reduced IRF4 expression, but indirectly through *MYC* itself. CBP/EP300 bromodomain inhibition was sufficient to reduce IRF4 mRNA levels but not IRF4 protein levels, which might partly be explained by the long half-life of IRF4 in MM cell lines (33–61 h), compared to the short half-life of c-Myc (30 min).^12^

The strategy of targeted protein degradation through proteolysis targeting chimeras (PROTACs, commonly referred to as degraders) provides an alternative approach for small-molecule modulation of target proteins. The advantages of degraders as a modality have been extensively reviewed elsewhere and include an altered pharmacokinetic/pharmacodynamic (PK/PD) regime that can elicit beneficial outcomes compared to small-molecule inhibition.^13−22^ For example, degraders can induce durable PD responses extending beyond the detectable presence of the degrader itself, resulting in a long-lasting reduction in protein levels, particularly those with long half-lives.^23^ Further, efficacious degraders can be designed using poorly active small-molecule inhibitors.^24,25^

**Figure 1:**
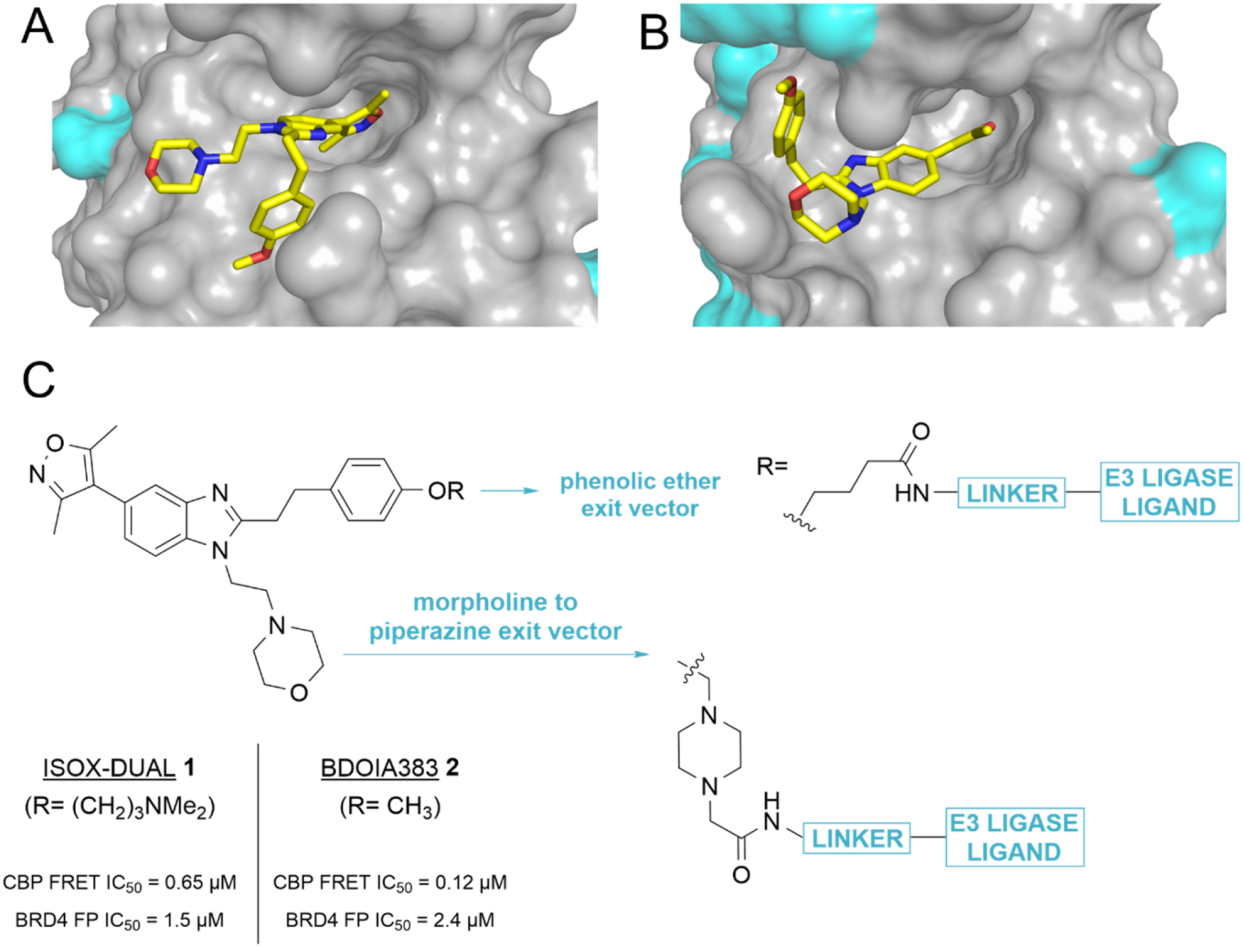
Choice of the starting scaffold and exit vectors. (A) Crystal structure of the CBP bromodomain with bound inhibitor **2** (5CGP). (B) Structure of the first bromodomain of BRD4 (5CFW) with the same inhibitor, showing ether (OMe) and morpholine solvent-exposed prospective exit vectors. Surface lysines are shown in cyan. (C) Structure of ISOX-DUAL **1** and **2** with representative structural design of the proposed phenolic ether and piperazine exit vectors.

Reduction of c-Myc levels has been demonstrated following treatment with degraders independently targeting both CBP/EP300^26−31^ and BRD4.^32−42^ Here, we pursued a strategy of targeting c-Myc through dual degradation of both CBP/EP300 and BRD4, via the small-molecule inhibitor ISOX-DUAL **1**(43) (Figure 1). The latter inhibits BRD4 and CBP/EP300 with micromolar potency and was deemed to be a suitable candidate to assess whether a dual degradation approach would offer a synergistic benefit in the reduction of c-Myc, compared to inhibition or degradation of the proteins individually. We recently successfully optimized the synthesis of **1**,^44^ providing ready access to larger quantities of precursors suitable for onward chemistry to enable synthesis of panels of candidate degraders. Here, we report the design, synthesis, and evaluation of libraries of molecules based on **1**, as the protein of interest binder (POI), with suitable exit vectors, various linkers, and E3 ligase ligands, to explore the potential for rational design of a degrader with a balanced dual degradation profile toward CBP/EP300 and BRD4.

## Results and Discussion

BDOIA383, **2**, an early chemical probe with the same core phenotype as ISOX-DUAL, **1**, provided a convenient starting point for determining suitable exit vectors from the ISOX-DUAL scaffold. Cocrystal structures of the first bromodomain of BRD4 (BRD4 BD1) and CBP bromodomains in complex with **2**(43,45) revealed two potential solvent-facing vectors for linker attachment: the phenolic ether and morpholine groups (Figure 1A,B). We proposed modification to enable linker attachment at either position: dealkylation of the ether, to reveal a phenol, and replacement of the morpholine group by a piperazine (Figure 1C).

**Scheme 1:**
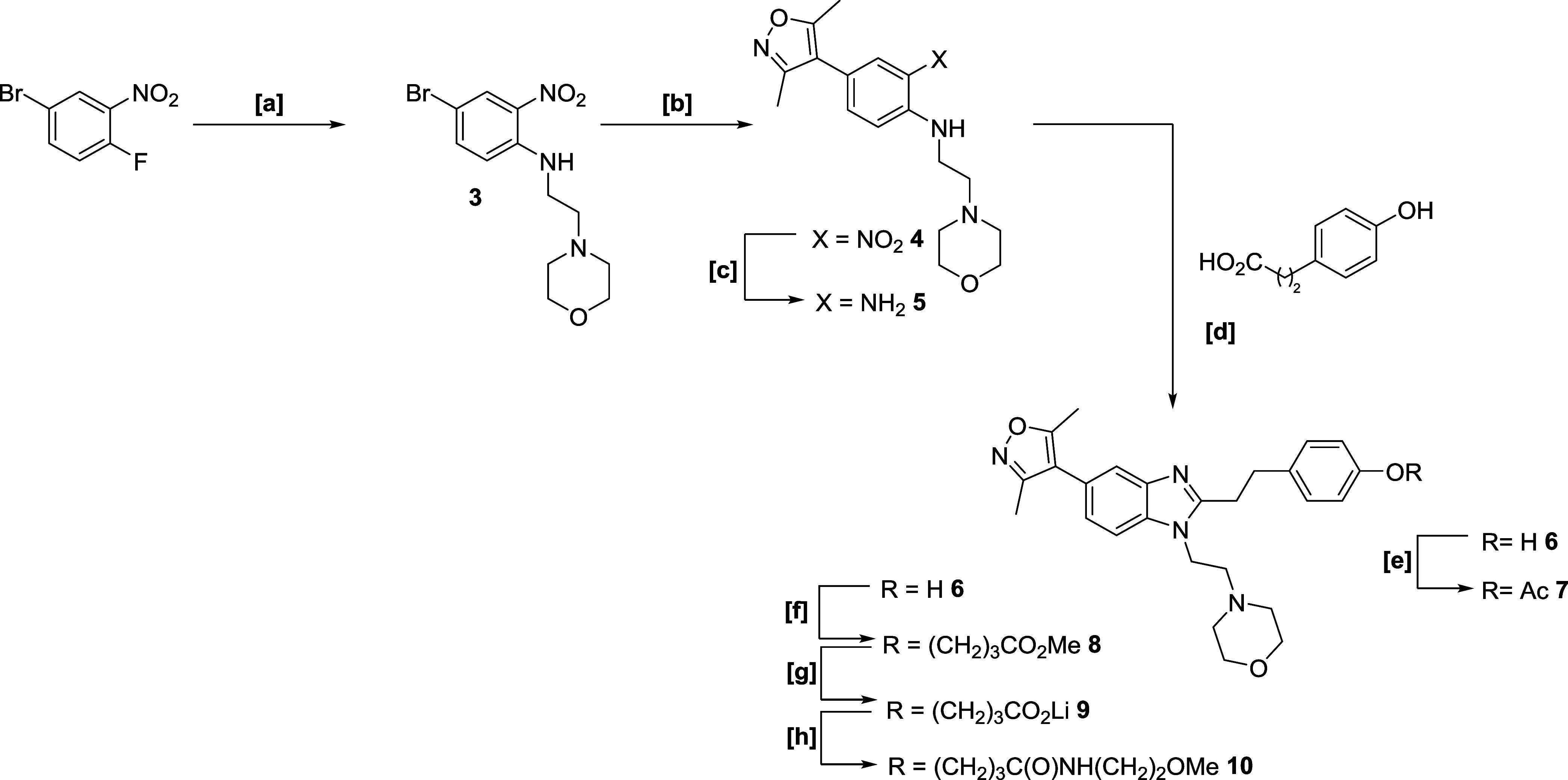
Synthetic Approach to ISOX-DUAL Analogues for the Validation of the Phenolic Exit Vector Reagents & conditions: [a] 4-(2-aminoethyl)morpholine, Et_3_N, dimethyl sulfoxide (DMSO), 80 °C, 90%; [b] 3.5-dimethylisoxazole-4-boronic acid pinacol ester, K_3_PO_4_, PdCl_2_(dppf).CH_2_Cl_2_, 1,4-dioxane, water, reflux, 94%; [c] (i) 1 M Na_2_S_2_O_4_ (aq), EtOH, 80 °C; (ii) 10% NH_3_ (aq), 81%; [d] (i) HATU, Et_3_N, *N*,*N*-dimethylformamide (DMF); (ii) AcOH, reflux 30%; [e] Ac_2_O, DCM, pyridine, rt, 80%; [f] K_2_CO_3_, methyl-4-bromobutyrate, MeCN, reflux, 70%; [g] LiOH, tetrahydrofuran (THF), water, rt, 94%; [h] 2-methoxyethylamine, NEt_3_, DMF, HATU, rt, 79%.

We first explored the phenolic ether exit vector strategy, synthesizing a small panel of analogues to assess the feasibility of functionalizing this part of the molecule while retaining balanced binary affinity to both target bromodomains, BRD4 and CBP (Scheme 1). Starting with the nitro analogue **3**, known intermediate **5** was subjected to coupling/cyclization procedures previously developed for related analogues.^44^ A series of products, **6**–**10**, were synthesized. The biochemical binary binding affinities of selected compounds to both BRD4 and CBP were measured using a fluorescence resonance energy transfer (FRET) assay (Table 1) using compounds **1**, **2** as controls. Phenol **6** had a similar affinity to both bromodomains as the parent compounds **1** and **2**, and affinities toward BRD4 were improved upon the incorporation of an acetyl (**7**), alkyl ester (**8**), and longer amide–ether linkages (**10**), the latter two representing model compounds for exit vectors and potential degraders. Overall, the phenol ether exit vector represented a promising platform for degrader synthesis because biological activity, in biochemical assays, was not unduly compromised.

**Table 1:** Structure–Activity Relationships for BRD4 BD1 and CBP Binding as Determined by FRET for the Phenolic Exit Vector Analogues^a^

compound	R	BRD4 IC_50_ (μM)	CBP IC_50_ (μM)
**6**	H	3.0 ± 0.1	0.3 ± 0.1
**7**	Ac	1.3 ± 0.1	0.17 ± 0.04
**8**	(CH_2_)_3_CO_2_Me	1.55 ± 0.01	0.5 ± 0.1
**10**	(CH_2_)_3_C(O)NH(CH_2_)_2_OMe	1.62 ± 0.01	0.33 ± 0.05
**2**	Me	2.4^b^	0.12^b^
**1**	(CH_2_)_3_NMe_2_	1.5^b^	0.65^b^

a Values given as mean ± SD (*n* = 3).

b Values from literature.^43^

**Scheme 2:**
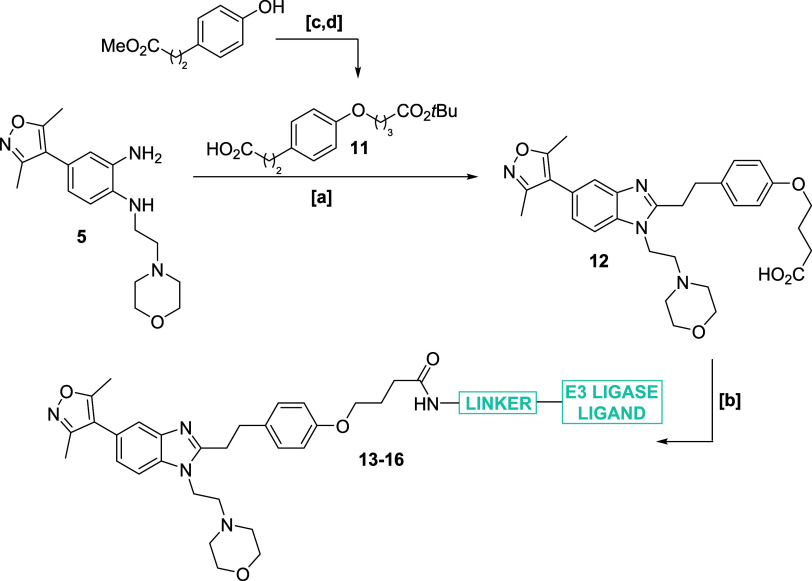
Synthesis of ISOX-DUAL Phenolic Ether Degraders Reagents & Conditions: [a] (i) HATU, Et_3_N, DMF; (ii) AcOH, reflux; (iii) HCl (4 M in 1,4-dioxane), 46%; [b] H_2_N-LINKER-E3 ligand, HATU, Et_3_N, DMF; [c] *tert*-butylbromoacetate, K_2_CO_3_, MeCN, reflux, 75%; [d] 1 M LiOH (aq), THF, 94%.

We proceeded to synthesize a small panel of candidate degraders based on this exit vector strategy, using **5** as a key intermediate. Routine cyclization chemistry, involving the ester-acid lithium salt **11**, led to the important precursor **12**, which underwent amide couplings with a small range of amine-linker-E3 ligase analogues to afford candidate degraders **13**–**16** (Scheme 2). Affinities of the degraders to both BRD4 and CBP were again assessed biochemically using a FRET assay (Table 2). Good balances of affinity were observed for degraders **13**, **14**, and **16**, albeit with a marked increase in CBP IC_50_ values, whereas compound **15** was less active toward both bromodomains.

**Table 2:**
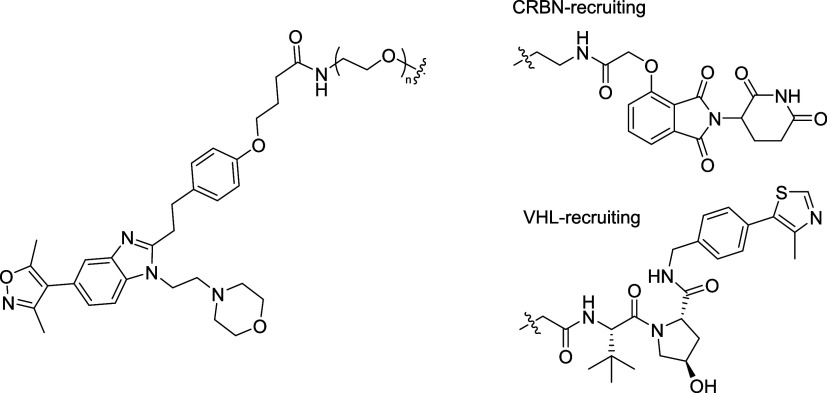
Structure–Activity Relationships for BRD4 BD1 and CBP Binding as Determined by FRET for the Phenolic Exit Vector Degraders^a^

compound (yield, %)	n	E3 ligase recruited	BRD4 IC_50_ (μM)^b^	CBP IC_50_ (μM)^b^
**13** (60)	3	CRBN	2.0 ± 0.1	1.4 ± 0.1
**14** (55)	4	CRBN	1.84 ± 0.04	1.5 ± 0.2
**15** (75)	3	VHL	4.6 ± 0.1	3.4 ± 0.3
**16** (65)	4	VHL	1.08 ± 0.03	1.5 ± 0.5
ISOX-DUAL	N/A	N/A	1.5^b^	0.65^b^

a Values given as mean ± SD (*n* = 3).

b Values from literature.^43^

**Scheme 3:**
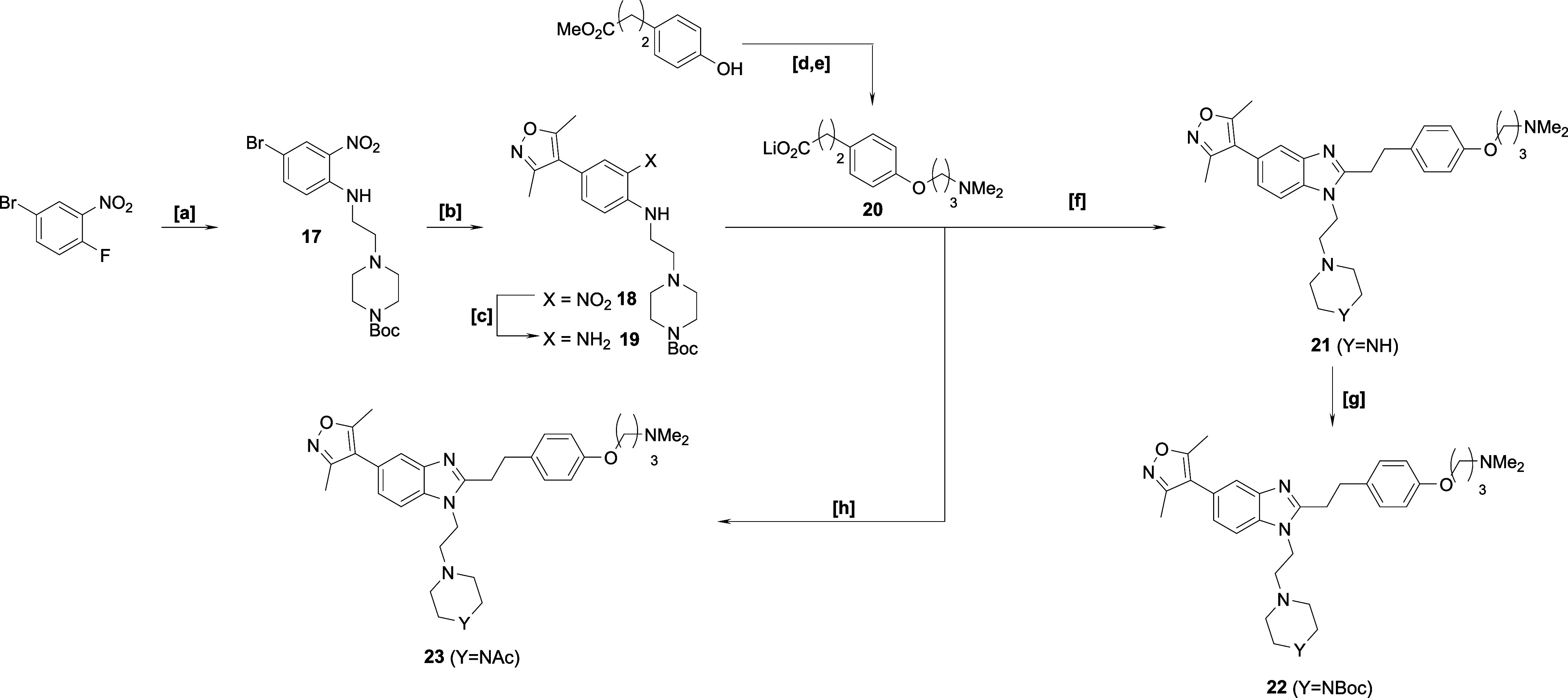
Synthesis of ISOX-DUAL Analogues for Validation of the Piperazine Exit Vector Reagents & conditions: [a] 4-(2-aminoethyl)-1-Boc-piperazine, Et_3_N, MW, 125 °C, 10 min, 98%; [b] 3,5-dimethylisoxazole-4-boronic acid pinacol ester, PdCl_2_(dppf)·CH_2_Cl_2_ (5 mol %), K_3_PO_4_, 1,4-dioxane, water, reflux, 80%; [c] (a) 1 M Na_2_S_2_O_4_ (aq), EtOH, 80 °C; (b) 10% NH_3_ (aq), 83%; [d] Br(CH_2_)_3_NMe_2_, *N*,*N*-diisopropylethylamine (DIPEA), DMF, 87%; [e] 1 M LiOH (aq), THF, quant. [f] (i) HATU, Et_3_N, DMF; (ii) 4 N HCl dioxane, MeOH, reflux, 37%; [g] Boc_2_O, DMAP, NEt_3_, DCM, rt, 43%; [h] (i) HATU, Et_3_N, DMF; (ii) AcOH, reflux, 34%.

Given the significant reduction in CBP affinities observed with our initial set of candidate degraders, we next explored the piperazine exit vector, aiming to obtain degrader molecules with more potent binary affinities to both targets. As before, a small panel of analogues was first synthesized to validate this position as a suitable exit vector and, in this instance, the ISOX-DUAL dimethylamino side chain was retained. Standard reduction chemistry delivered diamine **19**, which was cyclized with known **20**(44) to give benzimidazoles **21** and **23**. Reacting **21** in the presence of Boc anhydride afforded **22** (Scheme 3).

**Scheme 4:**
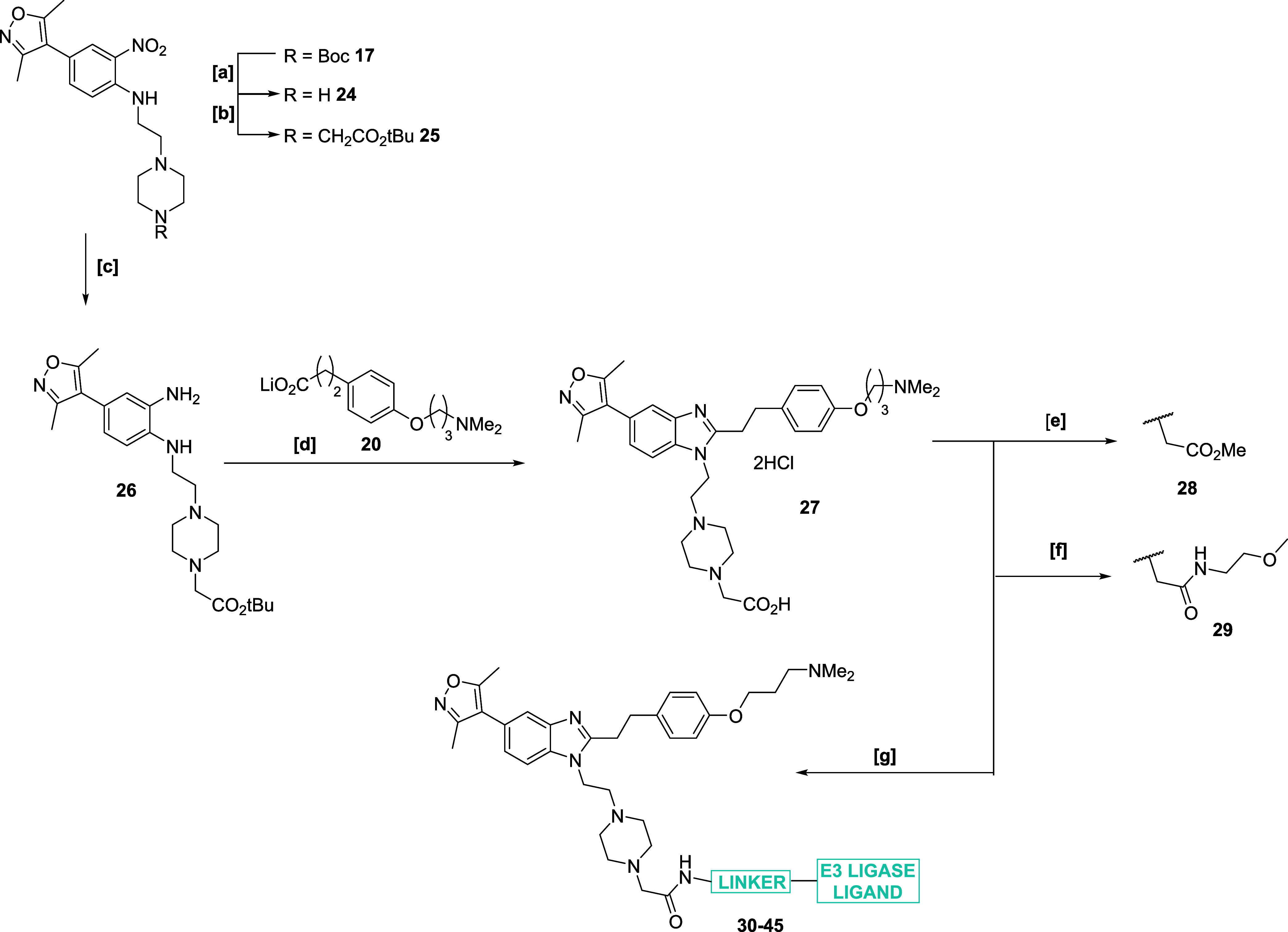
Synthesis of ISOX-DUAL Piperazine Analogues and Degraders Reagents & conditions: [a] TFA, DCM, 99%; [b] *tert*-butylbromoacetate, DIPEA, DCM, 80%; [c] (a) 1 M Na_2_S_2_O_4_ (aq), EtOH, 80 °C; (b) 10% NH_3_ (aq), 70%; [d] (i) HATU, Et_3_N, DMF; (ii) AcOH, reflux; (iii) HCl (4 M in 1,4-dioxane), 36%; [e] H_2_SO_4_, MeOH, reflux, 88%; [f] NEt_3_, 2-methoxyethylamine, HATU. DMF, rt, 77%; [g] H_2_N-LINKER-E3 Ligand, HATU, Et_3_N, DMF.

Instead of attempting to perform the derivatization of the piperazine late into the synthesis of our degrader precursor, the strategy was redesigned (Scheme 4); deprotecting intermediate **17** with trifluoroacetic acid (TFA) afforded free piperazine **24**, which was alkylated with *tert*-butylbromoacetate to afford **25**. Subsequent reduction of the nitro moiety afforded **26**, which was treated with the standard amide coupling and cyclization chemistry performed previously to afford our precursor compound **27**. From here we expanded our analogue library to methyl ester **28** and 2-methoxyethylamide **29**. Compounds **21**–**23**, **28** and **29** were analyzed in biochemical assays (Table 3) where the reference values for ISOX-DUAL were found to be around 2x higher than the literature value. Here, a significant loss of affinity toward both targets was observed in most cases, except for **29**, which had a good dual affinity toward both targets and was a preferable exit vector for degrader synthesis. Encouraged by the binary affinities observed with compound **29**, based on this exit vector strategy and utilizing key intermediate **27**, we designed a library of degraders (**30**–**45**).

**Table 3:** Structure–Activity Relationships for CBP and BRD4 BD1 Binding as Determined by FRET for the Piperazine Exit Vector Analogues^a^

compound (yield, %)	Y	BRD4 IC_50_ (μM)	CBP IC_50_ (μM)
ISOX-DUAL	O	3.6 ± 0.6 (1.5)^b^	1.20 (0.65)^b^
**21** (37%)	NH	5 ± 1	3.5 ± 0.1
**22** (43%)	NBoc	3.2 ± 0.6	1.2 ± 0.1
**23** (34%)	NAc	8 ± 3	2.1 ± 0.1
**28** (88%)	NCH_2_CO_2_Me	6 ± 2	2.1 ± 0.1
**29** (77%)	NCH_2_C(O)NH(CH_2_)_2_OMe	1.5 ± 0.4	0.83 ± 0.04

a Values given as mean ± SD (*n* = 2).

b Literature values.

A disappointing drop in CBP affinity, in biochemical assays, was observed for all compounds in this series with a surprising gain in affinity for BRD4 (Table 4), with candidate degraders displaying BRD4 affinities in the 60–210 nM range. It was unclear why addition of the linker and E3 ligase ligand results in such a clear drop in CBP binary affinity, given the strong validation of the exit vector at this position (Table 3). We decided to further test some of the candidate degraders to assess whether, despite the unbalanced potency, they might be able to induce productive ternary complex formation.

**Table 4:**
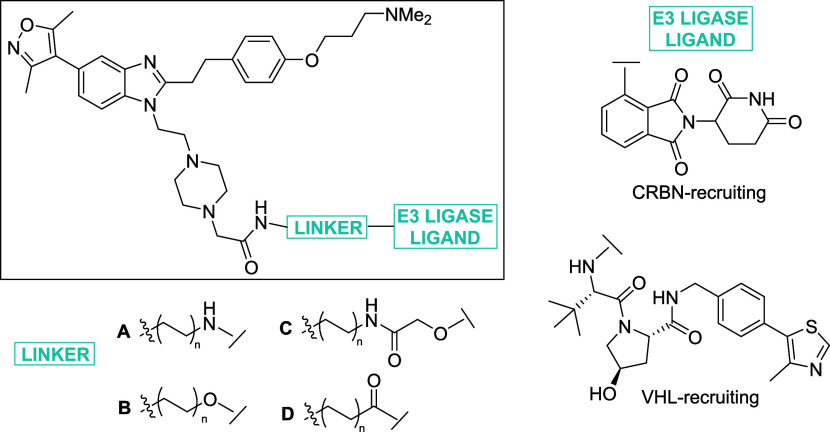
Structure–Activity Relationships for BRD4 and CBP Binding as Determined by FRET for the Piperazine Exit Vector Degraders^a^

compound (yield, %)	linker type	n	E3 ligase recruited	BRD4 IC_50_ (nM)	CBP IC_50_ (μM)	BRD4/CBP selectivity
**30** (36)	A	1	CRBN	160 ± 10	>20	>127
**31** (31)	A	2	CRBN	122 ± 4	8.3 ± 2.1	71
**32** (44)	A	3	CRBN	83 ± 3	4.5 ± 0.9	54
**33** (24)	A	4	CRBN	81 ± 4	10.0 ± 0.3	123
**34** (35)	B	1	CRBN	65 ± 6	6.7 ± 0.6	104
**35** (37)	B	2	CRBN	88 ± 2	14.0 ± 0.1	163
**36** (35)	B	3	CRBN	114 ± 4	>20	>175
**37** (52)	B	4	CRBN	88 ± 2	11.0 ± 1.1	121
**38** (16)	C	1	CRBN	74 ± 4	6.0 ± 0.39	82
**39** (32)	C	2	CRBN	111 ± 3	4.6 ± 1.5	42
**40** (43)	C	3	CRBN	211 ± 6	8.8 ± 4.5	42
**41** (20)	C	4	CRBN	122	13.0 ± 1.5	110
**42** (39)	D	1	VHL	161 ± 6	3.6 ± 0.13	22
**43** (22)	D	2	VHL	133 ± 14	3.7 ± 0.003	29
**44** (36)	D	3	VHL	101 ± 3	13.0 ± 0.3	131
**45** (35)	D	4	VHL	131 ± 8	9.1 ± 1.6	70
ISOX-DUAL	N/A	N/A	N/A	1500^b^	0.65^b^	0.43

a Values given as mean ± SD (*n* = 3).

b Values from the literature.^43^

**Figure 2:**
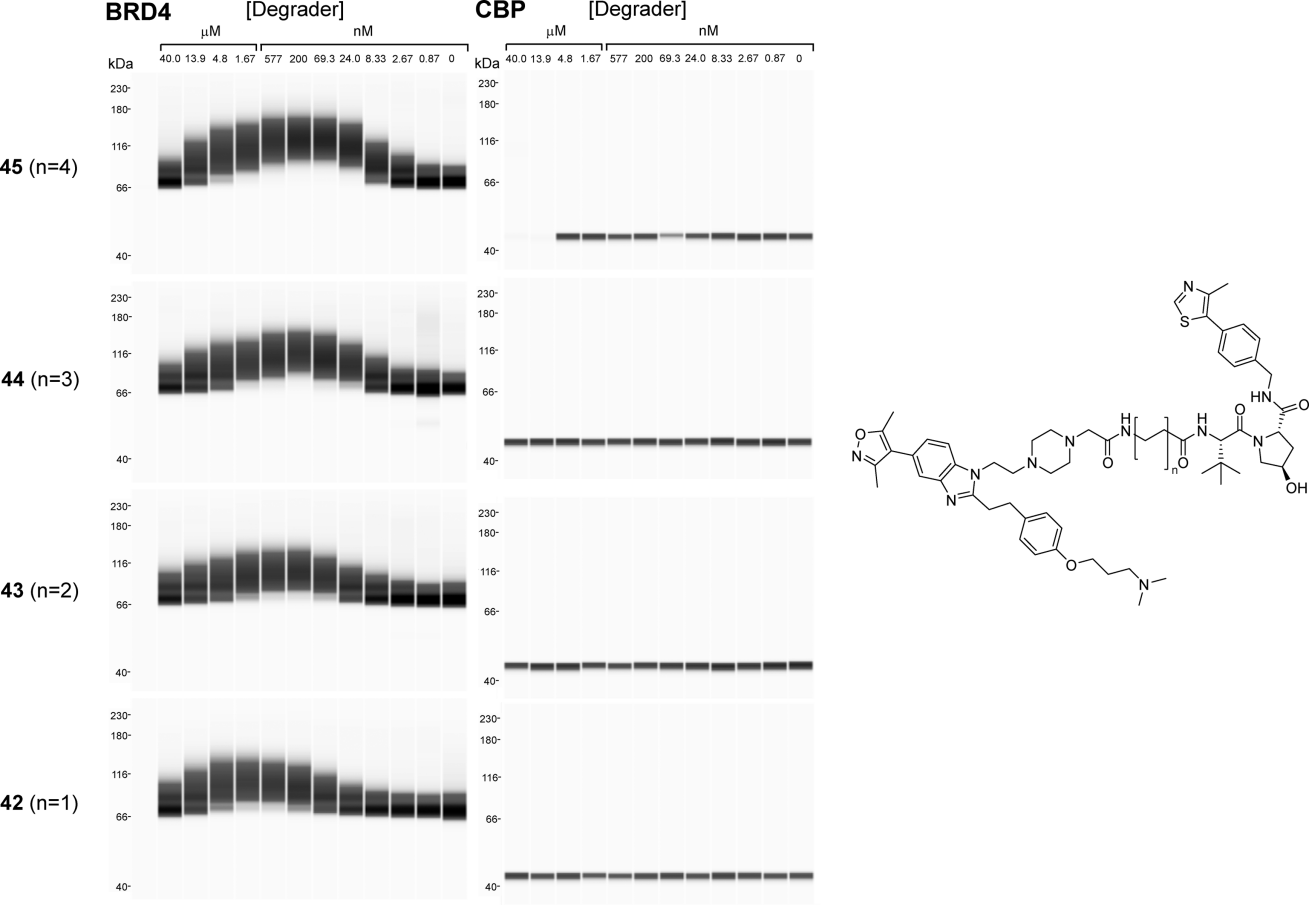
*In vitro* ubiquitination assays for VHL-recruiting degraders **42**–**45** with BRD4 (left panel) and CBP (right panel). FLAG-BRD4 and GST-CREBBP were detected using capillary electrophoresis (Simple Western, Wes) and anti-FLAG and anti-GST antibodies.

An *in vitro* ubiquitination assay was employed for an initial assessment of the ability for ISOX-DUAL-based degraders to induce productive ternary complex formation. This assay follows the ubiquitination of the target proteins in a cell-free system, removing potentially confounding factors such as degrader cell permeability and efflux.^19^ We first tested a subset of VHL-recruiting degraders (compounds **42**–**45**) and observed clear dose-dependent ubiquitination of BRD4, but not CBP, with all four degraders tested (Figure 2). A hook effect was evident for BRD4 ubiquitination, with structure–activity relationship (SAR) suggesting a greater degree of ubiquitination at lower compound concentration with increased linker length.

**Figure 3:**
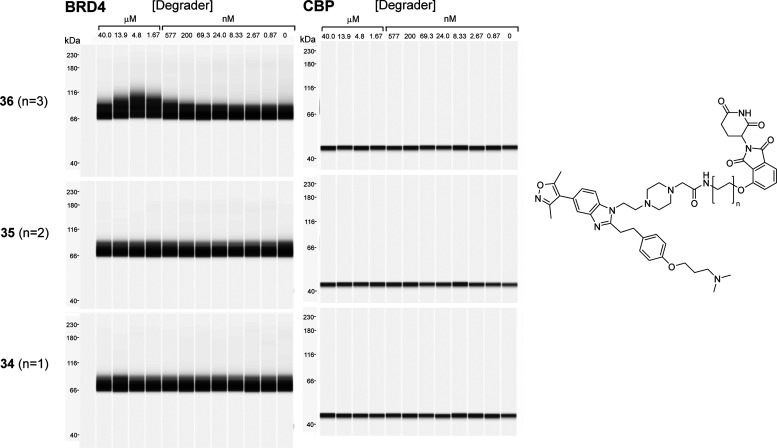
*In vitro* ubiquitination assays for CRBN-recruiting degraders **34**–**36** with BRD4 (left panel) and CBP (right panel). FLAG-BRD4 and GST-CREBBP were detected using capillary electrophoresis (Simple Western, Wes) and anti-FLAG and anti-GST antibodies.

A small panel of CRBN-recruiting degraders (compounds **34**–**36**) were also tested for their ability to ubiquitinate BRD4 and CBP in a cell-free environment (Figure 3). Limited ubiquitination of BRD4, but not CBP, was observed with the longest linker tested (compound **36**), with no observable ubiquitination for the degraders with shorter linkers (compounds **34** and **35**).

**Figure 4:**
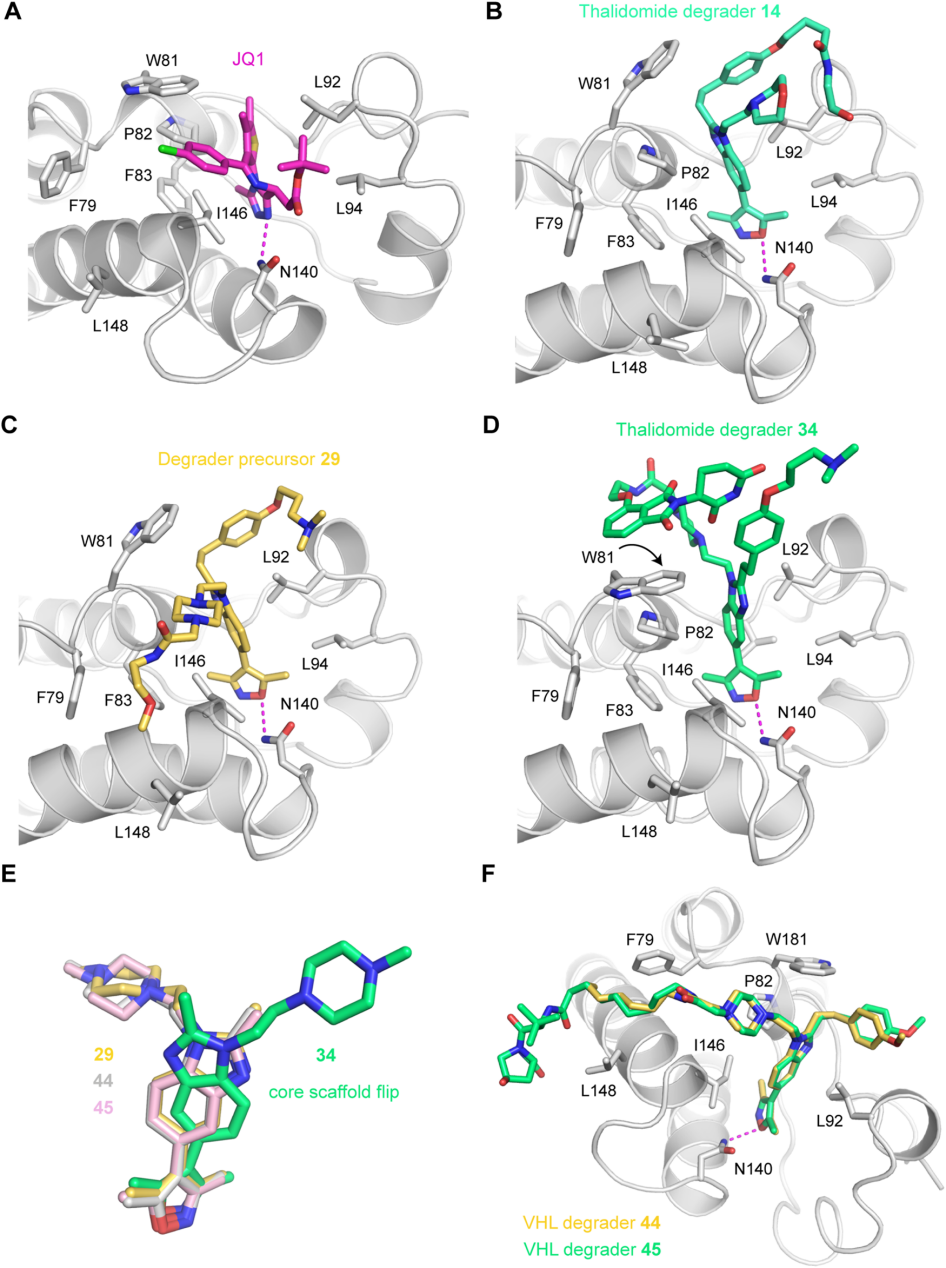
Crystal structures of the first bromodomain of human BRD4 in complex with JQ1 and ISOX-DUAL-based degraders. The ligand in each structure is shown as a stick model and the protein as a ribbon diagram, with selected side chains in the binding site highlighted as stick models. The binding mode of the isoxazole moiety of all degraders is conserved, forming a hydrogen bond with Asn140 at the bottom of the binding site (highlighted as a magenta dashed line) and hydrophobic interactions with Phe83, Val87, Leu94, and the gatekeeper residue Ile146. The central benzimidazole moiety is sandwiched between Pro82 and Leu92, with its relative orientation depending on the substitution pattern and degrader warhead. (A) BRD4 BD1 with bound (+)-JQ1 (PDB entry 3MXF). (B) BRD4 BD1 with thalidomide-based degrader **14**. The thalidomide moiety was not resolved in the crystal structure. (C) BRD4 BD1 with ISOX-DUAL inhibitor derivative **29**. (D) BRD4 BD1 with thalidomide-based degrader **34**. The thalidomide moiety was fully resolved in the structure, folding back onto Trp81 in the WPF-shelf region, thereby increasing binding affinity. (E) Superimposition of the core ISOX-DUAL scaffold in the BRD4 BD1 complexes with **29**, **34**, **44**, and **45**, highlighting the thalidomide-induced flip of the central benzimidazole scaffold upon binding of degrader **34**. (F) Superimposition of the binding modes of VHL-based degraders **44** (chain D) and **45** (chain B). The aliphatic linker packed against the surface patch between Phe79 and Leu148, whereas the VHL moiety was largely unresolved in the crystal structure, indicating high flexibility. For clarity, only the protein chain for the complex with **44** is shown.

We sought to rationalize the different affinities and ubiquitination patterns through the cocrystallization of representative degraders with BRD4 and the evaluation of the binding modes. Several high-resolution crystal structures (1.1–1.9 Å) of BRD4-degrader complexes were determined (Supporting Table S2). Degrader **14** bound with a similar pose to the parent scaffold **BDOIA383**, with the isoxazole oxygen forming the typical hydrogen bond with the highly conserved Asn140 at the bottom of the binding site, and the benzimidazole moiety packing between Pro82 in the WPF shelf and Leu92. The linker-thalidomide portion protruded into the solvent and was not resolved in the crystal structure (Figure 4B). We can therefore rationalize the similar BRD4 binding affinities for phenolic ether degrader **14** and **BDOIA383** (2.4 and 1.8 μM, respectively). The piperazine-based degrader **34**, however, adopted a surprising binding mode in the cocrystal structure. There was excellent electron density for the entire degrader molecule in this example, and the thalidomide moiety was found to fold back onto the protein, packing against the side chain of Trp81, which had flipped relative to its orientation in the complex with **BDOIA383** and the thalidomide-free parent molecule **29** (Figure 4C,D). Also, the central benzimidazole ring of **34** was rotated by about 180° and was slightly tilted compared with the orientation seen in the other complexes with this core scaffold (Figure 4E). This orientation of **34** was also stabilized via an interesting intramolecular interaction of the thalidomide piperidine-2,6-dione with the aromatic ring of the phenol ether moiety of the inhibitor. The packing of the thalidomide moiety against the Trp81 side chain is likely to contribute to the overall activity observed for this compound against BRD4 (Table 4) but could also rationalize the lack of ability to induce ubiquitination (Figure 3) since the thalidomide binding moiety is sequestered by BRD4 BD1 Trp81 and therefore not freely available for binding to form a ternary complex. The ‘collapse’ of degraders leading to E3 ligase ligand-target protein binding interactions has also been observed for VHL-recruiting degraders^46^ and may represent an under-reported mechanism by which some putative degrader molecules are inactive with respect to target protein degradation.^47^ Degrader **34** displayed a nanomolar (65 nM) activity against BRD4 BD1, similar to **(+)-JQ1**, and this prompted us to compare the binding poses of both. Indeed, **34** mimics **(+)-JQ1** by flipping Trp81 and interacting with it; the packing against Trp81, however, occurs from two different sides of the indole ring (Figure 4A,D and Supporting Figure S1). The crystal structure of the complex with **34** also showed an interesting stacking interaction of thalidomide moieties from symmetry-related molecules (Supporting Figure S2).

Cocrystal structures for VHL-based degraders **44** and **45** were solved with structures determined in a crystal form with four molecules in the asymmetric unit. The VHL-binding moiety was always disordered in these structures, but the linker was visible in most of the chains. When the linker was visible, it always packed against the hydrophobic surface patch between Phe79 and Leu148 (Figure 4F), although this may be influenced by crystal packing. The increased affinity compared with the parent scaffold may be due to such a hydrophobic interaction of the linker with a hydrophobic surface patch. The E3 ligase ligand is clearly solvent-exposed (as opposed to **35**) and this may also explain its ability to ubiquitinate BRD4.

Evaluation of the panel of degraders in cell-based assays led to ambiguous results, likely due to the cytotoxicity of the compounds confounding the degradation results at high treatment concentrations. Nonetheless, many of these compounds do serve as useful tools for biochemical investigation.

## Conclusions

We sought to rationally design degraders with dual activity against BRD4 and CBP/p300, using the parent inhibitor compound ISOX-DUAL. X-ray cocrystal structure informed the selection of two potential exit vectors, which were explored for degrader design. Several compounds displayed dual inhibitory activity, albeit in many cases with reduced affinity for one or both target proteins, in biochemical assays. Structural studies furthered our understanding of compound activity, with high-resolution X-ray cocrystal structures revealing an unexpected interaction between the E3 ligase recruiting ligand, thalidomide, and Trp81 on BRD4 BD1, resulting from the tryptophan side chain flipping in its relative orientation and effectively sequestering thalidomide, thereby preventing its binding to CRBN and abrogating degrader activity. Such a “degrader collapse” might be a hitherto under-represented mechanism for the lack of action in prototypical degrader design and may merit reinvestigation of past failures or prompt structural evaluation of binary complexes in future cases where the experimental results fail to align with biochemical binding results.

## Experimental Section

### General Methods

All reagents and solvents were purchased from commercial sources and used without further purification. Nuclear magnetic resonance spectra were recorded on Varian NMR machines operating at 600, 500, or 400 MHz for ^1^H NMR and at 151 or 126 MHz for ^13^C NMR, or on a Bruker Advance III HD spectrometer operating at 400 MHz for ^1^H NMR and 100 MHz ^13^C NMR. ^1^H NMR and ^13^C NMR chemical shifts (δ) are reported in parts per million (ppm) and are referenced to residual protium in solvent and to the carbon resonances of the residual solvent peak, respectively. DEPT and correlation spectra were run in conjunction to aid the assignment. Coupling constants (*J*) are quoted in Hertz (Hz), and the following abbreviations were used to report multiplicity: s = singlet, d = doublet, dd = doublet of doublets, ddd = double doublet of doublets, t = triplet, q = quartet, m = multiplet, br s = broad singlet. Purification by flash column chromatography was carried out using Teledyne ISCO purification systems. Analytical thin-layer chromatography was performed on commercial glass plates precoated with silica gel with visualization being achieved using UV light (254 nm) and/or by staining with alkaline potassium permanganate dip. Reaction monitoring LCMS analyses and purity determinations were conducted using a Shimadzu 2020 Mass Directed Automated Purification (MDAP) system or an Agilent Infinity Lab LC/MSD system. All final compounds are >95% pure by HPLC analysis except for **7** (92%), **28** (91%) and **45** (87%). HRMS analyses were conducted by Dr. Alaa Abdul-Sada in the laboratories of the University of Sussex Chemistry Department using a Bruker Daltonics Apex III, using Apollo ESI as the ESI source. For EI mass spectra, a Fissions VG Autospec instrument was used at 70 eV. Analyses are for the molecular ion peak [M]+ and are given in *m*/*z*, mass to charge ratio. Alphascreen assays were carried out following literature protocols.^48^

#### Synthesis of Compounds

##### 4-Bromo-*N*-(2-morpholinoethyl)-2-nitroaniline (**3**)

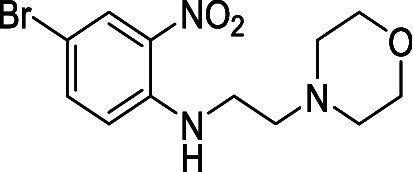



To a stirred solution of 4-bromo-1-fluoro-2-nitrobenzene **(2)** (29.9 g, 136 mmol) in DMSO (300 mL) at ambient temperature was added triethylamine (56 mL, 408 mmol, 3 equiv) followed by 4-(2-aminoethyl)morpholine (18.7 mL, 143 mmol,) in a dropwise fashion. The reaction mixture was then heated to 80 °C for 2 h. Upon completion of the reaction, the mixture was cooled to ambient temperature and partitioned between ethyl acetate (500 mL) and water (500 mL). The organic layer was collected and the aqueous was extracted with ethyl acetate (3 × 750 mL). The combined organic extracts were washed successively with sodium hydrogen carbonate solution (sat. aq.) (1L) and brine (1 L), dried over anhydrous magnesium sulfate, and concentrated under reduced pressure to give the title compound as an orange solid (40.6 g, 90%). ^1^H NMR (400 MHz CDCl_3_): δ 8.53 (br s, 1H), 8.33 (d, *J* = 2 Hz, 1H), 7.49 (dd, *J* = 9, 2 Hz, 1H), 6.73 (d, *J* = 9 Hz, 1H), 3.79–3.72 (m, 4H), 3.34 (q, *J* = 6 Hz, 2H), 2.72 (t, *J* = 6 Hz, 2H), 2.56–2.48 (m, 4H); LCMS (5–95% MeCN over 5 min) *t*_R_ = 3.178, Purity >99%; *m*/*z* (ES+): 332.1 [M+H^+^]^+^.

##### 4-(3,5-Dimethylisoxazol-4-yl)-*N*-(2-morpholinoethyl)-2-nitroaniline (**4**)

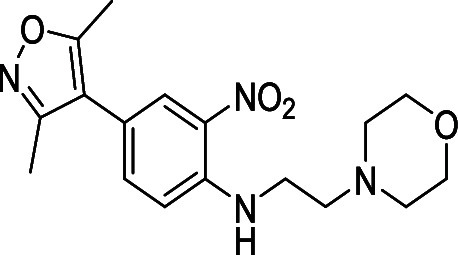



A stirred solution of 4-bromo-*N*-(2-morpholinoethyl)-2-nitroaniline **(3)** (38 g, 115 mmol), potassium phosphate (63.5 g, 299 mmol), and 3,5-dimethylisoxazole boronic acid pinacol ester (25.6 g, 115 mmol) in 1,4-dioxane (1.2 L) and water (120 mL) was degassed with argon (×3) before the addition of PdCl_2_(dppf)·DCM (4.7 g, 5.75 mmol). The reaction mixture was then degassed and refilled with argon once, heated to reflux, and stirred overnight under a stream of nitrogen (g). The reaction mixture was then cooled to ambient temperature and filtered through a pad of Celite before concentrating under reduced pressure to approximately 300 mL. The residue was then partitioned between water (600 mL) and ethyl acetate (600 mL), the organic phase was collected, and the aqueous phase was extracted with ethyl acetate (3 × 250 mL). The combined organic extracts were washed with brine (3 × 400 mL), dried over anhydrous magnesium sulfate, and concentrated under reduced pressure. Purification by flash column chromatography and elution with 0–80% ethyl acetate in hexane, afforded the title compound as an orange solid (37.4 g, 94%). ^1^H NMR (400 MHz, CDCl_3_): δ 8.58 (s, 1H), 8.09 (d, *J* = 2 Hz, 1H), 7.34 (dd, *J* = 9, 2 Hz, 1H), 6.91 (d, *J* = 9 Hz, 1H), 3.80–3.73 (m, 4H), 3.43 (q, *J* = 5.5 Hz, 2H), 2.75 (t, *J* = 5.5 Hz, 2H), 2.57–2.50 (m, 4H), 2.40 (s, 3H), 2.26 (s, 3H); LCMS (5–95% MeCN over 5 min) *t*_R_ = 3.266, Purity >99%; *m*/*z* (ES+): 347.2 [M+H^+^]^+^.

##### 4-(3,5-Dimethylisoxazol-4-yl)-N1-(2-morpholinoethyl)benzene-1,2-diamine (**5**)

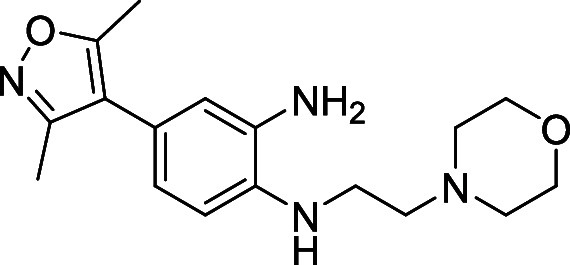



To a stirred suspension of 4-(3,5-dimethylisoxazol-4-yl)-*N*-(2-morpholinoethyl)-2-nitroaniline **(4)** (17.4 g, 50 mmol) in EtOH (800 mL) was added 1 M aqueous sodium dithionite solution (800 mL), and the resulting mixture was heated at 80 °C for 1 h. The reaction mixture was then cooled and partitioned between 10% aqueous ammonia solution (800 mL) and ethyl acetate (400 mL). The organic phase was separated, and the aqueous phase was extracted with ethyl acetate (4 × 400 mL). The combined organic extracts were washed with brine (2 × 500 mL), dried over anhydrous magnesium sulfate, and concentrated under reduced pressure to afford the title compound as a beige solid (12.70 g, 81%). ^1^H NMR (400 MHz, CDCl_3_): δ 6.68 (s, 2H), 6.59 (s, 1H), 4.08 (br s, 1H), 3.76–3.69 (m, 4H), 3.45 (br s, 2H), 3.23–3.17 (m, 2H), 2.71 (t, *J* = 5.9 Hz, 2H), 2.54–2.46 (s, 4H), 2.38 (s, 3H), 2.25 (s, 3H); LCMS (5–95% MeCN over 5 min) *t*_R_ = 2.364, Purity >99%; *m*/*z* (ES+): 317.2 [M+H^+^]^+^.

##### 4-(2-(5-(3,5-Dimethylisoxazol-4-yl)-1-(2-morpholinoethyl)-1*H*-benzo[*d*]imidazol-2-yl)ethyl)phenol (**6**)

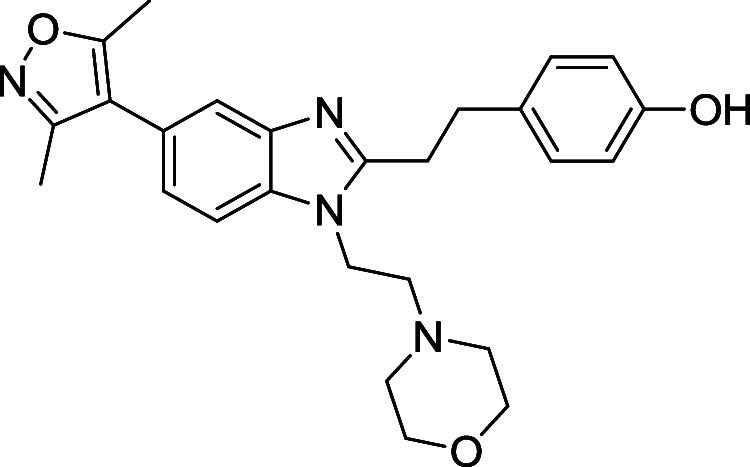



To a solution of 4-hydroxyphenyl propionic acid (686 mg, 4.13 mmol) and HATU (1.99 g, 5.25 mmol) in DMF (30 mL) was added triethylamine (1.6 mL, 11.3 mmol) followed by a solution of **5** (1.3 g, 3.75 mmol.) in DMF (5 mL). The stirring solution was left to stir overnight at ambient temperature. The reaction mixture was partitioned between dichloromethane (100 mL) and water (100 mL). The aqueous phase was then extracted with dichloromethane (3 × 25 mL). The combined organic phases were washed with saturated aqueous sodium hydrogen carbonate solution (150 mL) and brine (200 mL), dried over anhydrous magnesium sulfate, and concentrated under reduced pressure. The residue was dissolved in acetic acid (50 mL) and heated to reflux for 2 h. The reaction mixture was then cooled, concentrated under reduced pressure and dichloromethane (50 mL) was added before neutralization with saturated aqueous sodium hydrogen carbonate solution. The organic phase was separated, and the aqueous component was extracted with dichloromethane (4 × 50 mL), before being combined and washed with brine (200 mL), dried over anhydrous magnesium sulfate, and concentrated under reduced pressure. Purification by flash column chromatography and elution with 0–20% methanol (with 0.5% NH_4_OH) in dichloromethane afforded the title compound as a colorless solid. (502 mg, 30%). ^1^H NMR (600 MHz, CDCl_3_): δ 8.07 (br s, 1H), 7.58 (s, 1H), 7.36 (d, *J* = 8.2 Hz, 1H), 7.13 (d, *J* = 8.2 Hz, 1H), 7.00 (d, *J* = 7.8 Hz, 2H), 6.73 (d, *J* = 7.8 Hz, 2H), 4.17 (t, *J* = 6.8 Hz, 2H), 3.72–3.66 (m, 4H), 3.23–3.13 (m, 4H), 2.65 (t, *J* = 6.8 Hz, 2H), 2.52–2.45 (m, 4H), 2.39 (s, 3H), 2.26 (s, 3H); ^13^C NMR (151 MHz, CDCl_3_): δ 165.2, 159.1, 155.6, 155.4, 142.6, 134.2, 131.8, 129.5, 124.6, 123.7, 119.8, 117.1, 115.9, 109.7, 66.9, 57.7, 54.2, 41.7, 33.3, 29.9, 11.7, 11.0; LCMS (5–95% MeCN over 20 min) *t*_R_ = 3.23 min, Purity >97%; *m*/*z* (ES+): 447.05 [M+H^+^]^+^; HRMS-ESI (*m*/*z*): [M+H^+^]^+^ calculated for C_29_H_31_N_4_O_3_, 447.2391; found, 447.2367.

##### 4-(2-(5-(3,5-Dimethylisoxazol-4-yl)-1-(2-morpholinoethyl)-1*H*-benzo[*d*]imidazol-2-yl)ethyl)phenyl Acetate (**7**)

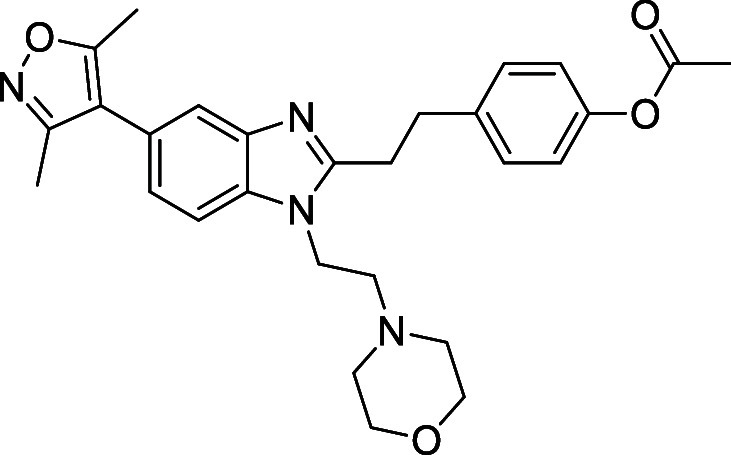



To a stirred solution of **6** (87 mg, 0.195 mmol) in dichloromethane (5 mL), was added pyridine (0.032 mL, 0.39 mmol) and acetic anhydride (0.037 mL, 0.39 mmol) at ambient temperature, and the mixture was left to stir for 1 h. The reaction mixture was then quenched with saturated aqueous NH_4_Cl (10 mL) and extracted with DCM (10 mL). The organic layer was collected, washed with brine (10 mL), dried over anhydrous magnesium sulfate, and concentrated under reduced pressure. Purification by flash column chromatography and elution with 0–10% methanol (with 0.5% NH_4_OH) in dichloromethane afforded the title compound as a clear oil. (76 mg, 80%).^1^H NMR (600 MHz, CDCl_3_): δ 7.63 (s, 1H), 7.35 (d, *J* = 8.2 Hz, 1H), 7.24 (d, *J* = 7.9 Hz, 2H), 7.12 (d, *J* = 8.2 Hz, 1H), 7.01 (d, *J* = 7.7 Hz, 2H), 4.10 (t, *J* = 6.8 Hz, 2H), 3.68–3.64 (m, 4H), 3.33–3.25 (m, 2H), 3.21–3.15 (m, 2H), 2.61 (t, *J* = 6.7 Hz, 2H), 2.48–2.43 (m, 4H), 2.42 (3H s), 2.30–2.26 (m, 6H). ^13^C NMR (151 MHz, CDCl_3_): 169.7, 165.1, 159.1, 155.2, 149.4, 143.1, 138.3, 134.4, 129.5, 124.4, 123.6, 121.9, 120.0, 117.2, 109.5, 66.9, 57.7, 54.1, 41.6, 33.3, 29.7, 21.2, 11.7, 11.0. LCMS (5–95 MeCN in 20 min) *t*_R_ = 4.10 min, Purity = 92%, HRMS-ESI (*m*/*z*): [M+H^+^]^+^ calculated for C_28_H_33_N_4_O_4_, 489.2496; found, 489.2477.

##### Methyl-4-(4-(2-(5-(3,5-dimethylisoxazol-4-yl)-1-(2-morpholinoethyl)-1*H*-benzo[*d*]imidazol-2-yl)ethyl)phenoxy)butanoate (**8**)

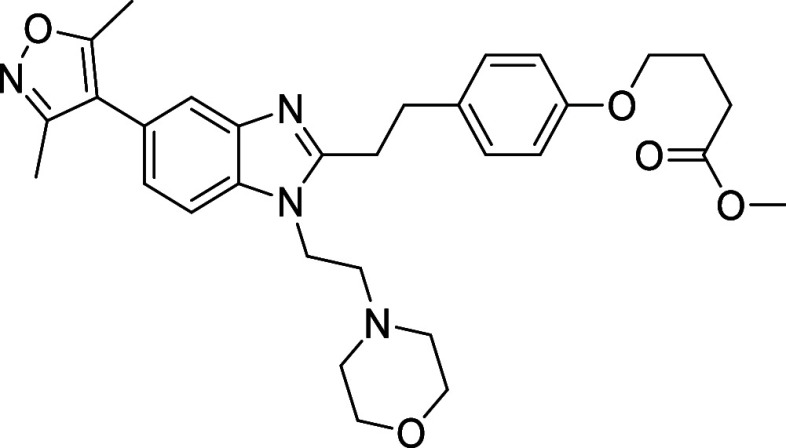



To a stirred solution of **6** (1.4 g, 3.1 mmol) in acetonitrile (50 mL) was added potassium carbonate(s) (0.857 g, 6.2 mmol), followed by methyl-4-bromobutyrate (1.1224 g, 6.2 mmol) before leaving to stir overnight at reflux. The reaction was cooled and partitioned between ethyl acetate (50 mL) and water (50 mL). The organic phase was collected, and the aqueous layer was extracted with ethyl acetate (3 × 25 mL). The organics were combined and washed with brine (100 mL), dried over anhydrous magnesium sulfate, and concentrated under reduced pressure. Purification by flash column chromatography and elution with 0–5% methanol in dichloromethane with 0.5% NH_4_OH, afforded the title compound as a clear oil (1.12 g, 70%). ^1^H NMR (500 MHz, CDCl_3_): δ 7.62 (s,1H), 7.35 (d, *J* = 8 Hz, 1H), 7.15–7.10 (m, 3H), 6.83 (d, *J* = 8 Hz, 2H), 4.12 (t, *J* = 7 Hz, 2H), 3.99 (t, *J* = 6 Hz, 2H), 3.7–3.64 (m, 7H), 3.25–3.12 (m, 4H), 2.60 (t, *J* = 7 Hz, 2H), 2.54 (t, *J* = 7 Hz, 2H), 2.48–2.41 (m, 7H), 2.30 (s, 3H), 2.12–2.08 (m, 2H); ^13^C NMR (126 MHz, CDCl_3_): δ 173.8, 165.2, 159.2, 157.6, 155.6, 143.2, 134.4, 133.1, 129.5, 124.3, 123.5, 120.0, 117.2, 114.8, 110.1, 109.5, 67.0, 66.8, 57.7, 54.2, 51.8, 41.6, 33.2, 30.7, 30.1, 24.7, 15.4, 11.7, 11.0; LCMS (5–95% MeCN over 20 min) *t*_R_ = 12.30 min, Purity >96%; *m*/*z* (ES+): 547.20 [M+H^+^]^+^; HRMS-ESI (*m*/*z*): [M+H^+^]^+^ calculated for C_31_H_39_N_4_O_5_, 547.2915; found, 547.2892.

##### Lithium-4-(4-(2-(5-(3,5-dimethylisoxazol-4-yl)-1-(2-morpholinoethyl)-1*H*-benzo[*d*]imidazol-2-yl)ethyl)phenoxy)butanoate (**9**)

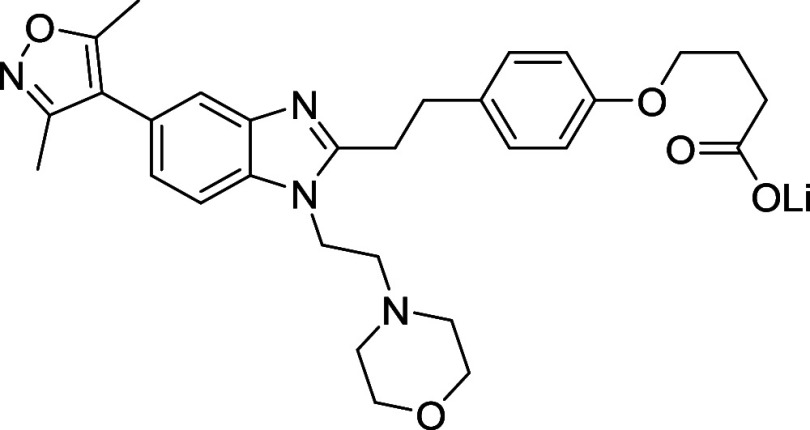



To a stirred solution of **8** (1.1 g, 2.5 mmol) in THF (100 mL) and water (20 mL) was added lithium hydroxide monohydrate (0.115 g, 2.75 mmol) and was left to stir at ambient temperature overnight. Upon reaction completion, the resultant solution was concentrated under reduced pressure, and reconcentrated from THF (5 × 50 mL), to give the title compound as a colorless solid, which was used directly in the subsequent reaction without further purification (1.0 g, 94%). LCMS (5–95% MeCN over 20 min) *t*_R_ = 11.04, Purity >95%; *m*/*z* (ES-): 531.15 [M-Li^+^]^−^.

##### 4-(4-(2-(5-(3,5-Dimethylisoxazol-4-yl)-1-(2-morpholinoethyl)-1*H*-benzo[*d*]imidazol-2-yl)ethyl)phenoxy)-N-(2-methoxyethyl)butanamide (**10**)

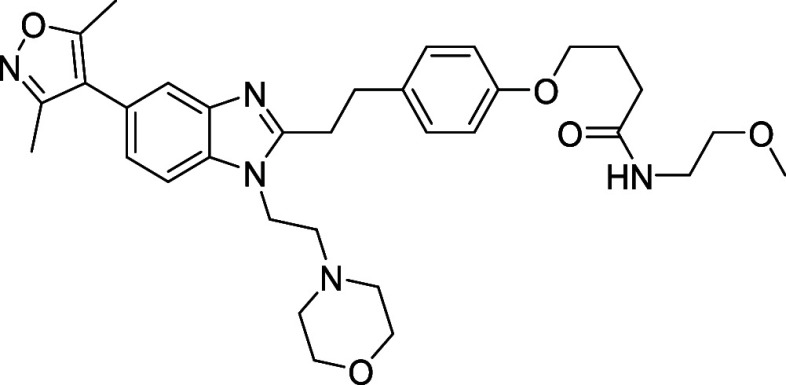



To a stirred solution of **9** (100 mg, 0.19 mmol, 1 equiv) and HATU (83.7 mg, 0.22 mmol, 1.2 equiv) in DMF (mL) was added triethylamine (26.5 μL, 0.19 mmol, 1.2 equiv) followed by 2-methoxyethylamine (14.3 mg, 0.19 mmol, 1 equiv) before leaving the reaction to stir at ambient temperature overnight. The mixture was partitioned between dichloromethane (25 mL) and water (25 mL). The organic layer was collected and washed with saturated aqueous sodium hydrogen carbonate solution (25 mL) and brine (3 × 10 mL), dried over anhydrous magnesium sulfate and concentrated under reduced pressure. Purification by flash column chromatography and elution with 0–10% methanol in dichloromethane with 0.5% NH_4_OH, afforded the title compound as a colorless oil (88 mg, 79%). ^1^H NMR (600 MHz, CDCl_3_): δ 7.62 (s, 1H), 7.34 (d, *J* = 9 Hz, 1H), 7.15–7.10 (m, 3H), 6.82 (d, *J* = 9 Hz, 2H), 5.94 (s, 1H), 4.11 (t, *J* = 7 Hz, 2H), 3.98 (t, *J* = 6 Hz, 2H), 3.68–3.63 (m, 4H), 3.45–3.42 (m, 4H), 3.32 (s, 3H), 3.24–3.19 (m 2H), 3.18–3.14 (m, 2H), 2.59 (t, *J* = 6.9 Hz, 2H), 2.48–2.43 (m, 4H), 2.42 (s, 3H), 2.39 (t, *J* = 7.3 Hz, 2H), 2.29 (s, 3H), 2.15–2.08 (m, 2H). ^13^C NMR (151 MHz, CDCl_3_): δ 172.4, 165.2, 159.1, 157.6, 155.5, 143.1, 134.4, 133.1, 129.5, 124.3, 123.5, 120.0, 117.2, 114.8, 109.5, 71.3, 67.0, 66.9, 58.9, 57.7, 54.1, 41.6, 39.3, 33.1, 33.0, 30.0, 11.7, 11.0; LCMS (5–95% MeCN over 20 min) *t*_R_ = 11.08 min, Purity >99%; *m*/*z* (ES+): 590.20 [M+H^+^]^+^; HRMS-ESI (*m*/*z*): [M+Na^+^]^+^ calculated for C_33_H_43_N_5_O_5_Na, 612.3156; found, 612.3143.

##### *tert*-Butyl 4-(4-(3-Methoxy-3-oxopropyl)phenoxy)butanoate (**11a**)

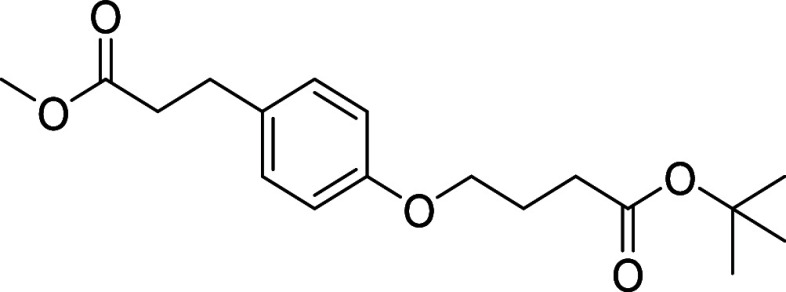



To a solution of methyl 3-(4-hydroxyphenyl)propionate (20 g, 111 mmol) in acetonitrile (200 mL) was added potassium carbonate (30.6 g, 222 mmol), followed by the dropwise addition of a solution of *tert*-butyl 4-bromobutanoate (25 g, 111 mmol.) in acetonitrile (100 mL). The reaction mixture was then heated to reflux overnight. Upon cooling the reaction mixture was filtered and the filtrate was and partitioned between dichloromethane (700 mL) and water (500 mL). The organic phase was separated and washed with 1 M (aq) potassium carbonate solution (10 × 250 mL) and brine (2 × 300 mL) before being dried over anhydrous magnesium sulfate and concentrated under reduced pressure. Purification by flash column chromatography and elution with 5–20% ethyl acetate in petroleum ether (40–60), afforded the title compound as a colorless oil, which crystallized upon standing (27 g, 75%). ^1^H NMR (400 MHz, CDCl_3_): δ 7.10 (d, *J* = 9 Hz, 2H), 6.81 (d, *J* = 9 Hz, 2H), 3.96 (t, *J* = 7 Hz, 2H), 3.66 (s, 3H), 2.88 (t, *J* = 8 Hz, 2H), 2.59 (t, *J* = 8 Hz, 2H), 2.41 (t, *J* = 7 Hz, 2H), 2.08–2.01 (m, 2H), 1.45 (s, 9H); LCMS (5–95% MeCN over 5 min) *t*_R_ = 5.879, Purity >96%; *m*/*z* (ES+): 289.2 [M-*t*Bu+Na^+^]^+^.

##### 4-(4-(3-Methoxy-3-oxopropyl)phenoxy)butanoic Acid (**11**)

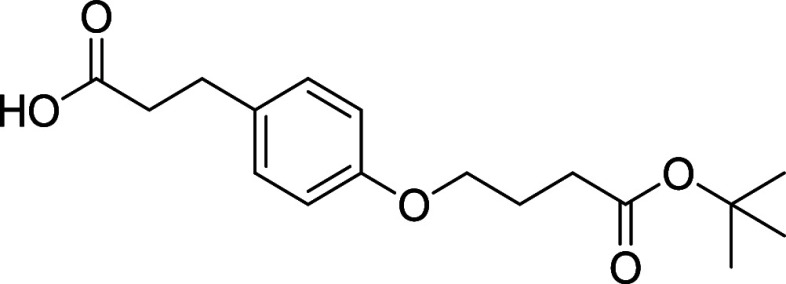



To a stirred solution of *tert*-butyl 4-(4-(3-methoxy-3-oxopropyl)phenoxy)butanoate (27 g, 87.7 mmol) in THF (200 mL) was added lithium hydroxide (200 mL, 1 M aqueous solution). The reaction mixture was left to stir until completion by TLC. The reaction was then acidified to pH 2 with 2 M HCl and extracted with ethyl acetate (4 × 200 mL). The combined organic extracts were washed with brine, dried over anhydrous magnesium sulfate, and concentrated under reduced pressure affording the title compound as a colorless solid (24.3 g, mmol, 94%). ^1^H NMR (400 MHz, CDCl_3_): δ 12.09 (br s, 1H), 7.11 (d, *J* = 8.5 Hz, 2H), 6.81 (d, *J* = 8.5 Hz, 2H), 3.94–3.90 (m, 2H), 2.74 (t, *J* = 7.5 Hz, 2H), 2.46 (t, *J* = 7.5 Hz, 2H), 2.34 (t, 7.5 Hz, 2H), 1.93–1.87 (m, 2H), 1.40 (s, 9H).

##### 4-(4-(2-(5-(3,5-Dimethylisoxazol-4-yl)-1-(2-morpholinoethyl)-1*H*-benzo[*d*]imidazol-2-yl)ethyl)phenoxy)butanoic Acid (**12**)

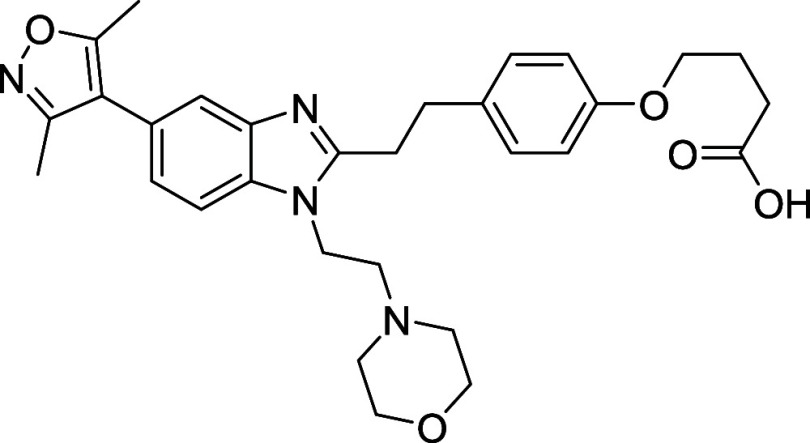



To a suspension of compound **11** (12.38 g, 40.14 mmol) in DMF (200 mL), was added triethylamine (16.8 mL, 120 mmol) and HATU (19.84 g, 15.1 mmol). The reaction mixture was degassed with argon and stirred for 1 h before the addition of a solution of compound **5** (12.7 g, 40.14 mmol) in DMF (150 mL). After stirring at ambient temperature overnight, the reaction mixture was partitioned between ethyl acetate (500 mL) and water (2 L). The organic phase was separated, and the aqueous component was extracted with ethyl acetate (3 × 300 mL). The organic extracts were combined and successively washed with saturated aqueous sodium hydrogen carbonate solution (150 mL) and brine (150 mL), before being dried over anhydrous magnesium sulfate and concentrated under reduced pressure. Purification by flash column chromatography and elution with 1–4% 7N methanolic ammonia solution in dichloromethane afforded a pale brown solid. This solid was dissolved in acetic acid and heated to reflux for 2 h, after which the reaction mixture was cooled, concentrated under reduced pressure, and successively reconcentrated from ethyl acetate (100 mL) and then heptane (3 × 300 mL). The residue was dissolved in ethyl acetate (300 mL) and poured into a saturated aqueous sodium hydrogen carbonate solution (400 mL). The organic phase was collected and washed with brine (500 mL), dried over anhydrous magnesium sulfate, and concentrated under reduced pressure. Purification by flash column chromatography and elution with 3–7% 7N methanolic ammonia solution in dichloromethane afforded a beige solid. This solid was dissolved in 1,4-dioxane (100 mL) and hydrogen chloride (50 mL, 200 mmol, 4 M solution in 1,4-dioxane) was added before leaving to stir for 4 h, after which the reaction mixture was concentrated and triturated overnight with acetonitrile. The resulting precipitate was filtered and washed with diethyl ether and dried under vacuum, affording the title compound as a colorless solid (10.5 g, 46%).

^1^H NMR (400 MHz, DMSO-*d*_6_): δ 12.57 (br s, 1H), 8.29 (d, 8.5 Hz, 1H), 7.81, (s, 1H), 7.60 (d, 8.6 Hz, 1H), 7.32 (d, 8.6 Hz, 2H), 6.88 (d, 8.6 Hz, 2H), 5.05–4.97 (m, 2H), 4.07–3.97 (m, 2H), 3.95 (t, *J* = 6.4 Hz, 2H), 3.91–3.82 (m, 2H), 3.58–3.45 (m, 6H), 3.26–3.15 (m, 4H), 2.43 (s, 3H), 2.37 (t, *J* = 7.3 Hz, 2H), 2.24 (s, 3H), 1.95–1.88 (m, 2H); ^13^C NMR (151 MHz, DMSO-*d*_6_): 174.1, 165.7, 158.2, 157.3, 155.0, 131.8, 131.2, 131.0, 129.7, 127.7, 126.5, 115.5, 114.7, 114.5, 113.4, 66.6, 66.4, 63.2, 51.9, 51.1, 40.1, 38.5, 31.3, 30.1, 27.4, 24.3, 11.3, 10.5. LCMS (30–95 MeCN over 20 min) *t*_R_ = 7.27 min, Purity >95%, *m*/*z* (ES+): 533.55 [M+H^+^]^+^. HRMS-ESI (*m*/*z*): [M+H^+^]^+^ calculated for C_30_H_37_N_4_O_5_, 533.2764; found, 533.2785.

#### General Procedure A for Degrader Synthesis

A solution of **12** (1 equiv) in DMF (typically 5 mL) was treated with the relevant commercially available amine-reactive degrader building block (E3 ligase ligand functionalized with a linker with amine termini) (1 equiv; typically 25 mg), triethylamine (3 equiv) and HATU (1.3 equiv) and stirred overnight at ambient temperature. The reaction mixture was partitioned between dichloromethane (25 mL) and water (50 mL), and the organic phase was separated. The aqueous component was extracted with further dichloromethane (4 × 10 mL), and the combined organics were then washed with saturated aqueous sodium hydrogen carbonate solution (50 mL) and brine (2 × 50 mL), dried over anhydrous magnesium sulfate, and concentrated under reduced pressure.

##### 4-(4-(2-(5-(3,5-Dimethylisoxazol-4-yl)-1-(2-morpholinoethyl)-1*H*-benzo[*d*]imidazol-2-yl)ethyl)phenoxy)-*N*-(1-((2-(2,6-dioxopiperidin-3-yl)-1,3-dioxoisoindolin-4-yl)oxy)-2-oxo-6,9,12-trioxa-3-azatetradecan-14-yl)butanamide (**13**)

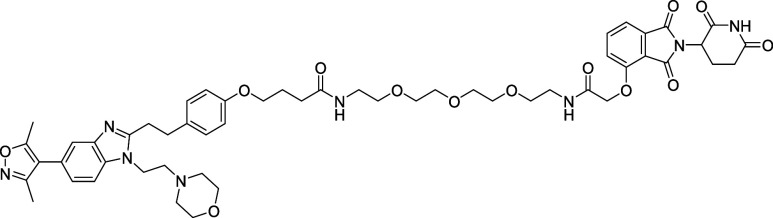



Degrader **13** was synthesized according to General Procedure A, using thalidomide 4′-oxyacetamide-PEG3-amine. Purification by flash column chromatography and elution with 0–20% methanol (with 0.5% NH_4_OH) in dichloromethane over 20 column volumes (cvs), afforded the title compound **13** as a colorless oil. (29.6 mg, 60%). ^1^H NMR (500 MHz, CDCl_3_): δ 9.64 (br s, 1H), 7.75–7.66 (m, 2H), 7.63 (d, *J* = 0.8 Hz, 1H), 7.53 (d, *J* = 7.3 Hz, 1H), 7.36 (d, *J* = 8.3 Hz, 1H), 7.16 (d, *J* = 8.3 Hz, 1H), 7.13 (d, *J* = 1.3 Hz, 1H), 7.11 (d, *J* = 8.5 Hz, 2H), 6.79 (d, *J* = 8.5 Hz, 2H), 6.73–6.68 (m, 1H), 5.60–5.40 (m, 2H), 4.93 (dd, *J* = 12.3, 5.4 Hz, 1H), 4.62 (s, 2H), 4.13 (t, *J* = 6.9 Hz, 2H), 3.95 (t, *J* = 6.1 Hz, 2H), 3.69–3.36 (m, 18H), 3.23–3.15 (m, 4H), 2.88–2.83 (m, 1H), 2.77–2.68 (m, 2H), 2.60 (t, *J* = 6.9 Hz, 2H), 2.49–2.45 (m, 3H), 2.42 (s, 3H), 2.37 (t, *J* = 7.3 Hz, 2H), 2.29 (s, 3H), 2.17–2.05 (m, 4H); ^13^C NMR (126 MHz, CDCl_3_): δ 172.8, 171.7, 171.7, 168.7, 166.9, 166.7, 166.0, 165.2, 159.1, 157.7, 155.49, 154.4, 137.1, 134.1, 133.7, 132.9, 129.5, 124.5, 123.7, 119.8, 119.4, 188.1, 117.4, 117.1, 114.8, 110.1, 109.7, 70.3, 70.3, 70.2, 70.2, 69.6, 67.8, 67.2, 66.8, 57.6, 54.1, 49.3, 41.4, 39.1, 33.1, 32.8, 31.5, 29.9, 25.3, 22.9, 22.8, 11.7, 11.0; LCMS (5–95% MeCN over 20 min) *t*_R_ = 11.77 min, purity >99%; *m*/*z* (ES+): 1043.40 [M+Na^+^]^+^; HRMS-ESI (*m*/*z*): [M+Na^+^]^+^ calculated for C_53_H_64_N_8_NaO_13_, 1043.4491; found, 1043.4453.

##### 4-(4-(2-(5-(3,5-Dimethylisoxazol-4-yl)-1-(2-morpholinoethyl)-1*H*-benzo[*d*]imidazol-2-yl)ethyl)phenoxy)-*N*-(1-((2-(2,6-dioxopiperidin-3-yl)-1,3-dioxoisoindolin-4-yl)oxy)-2-oxo-6,9,12,15-tetraoxa-3-azaheptadecan-17-yl)butanamide (**14**)

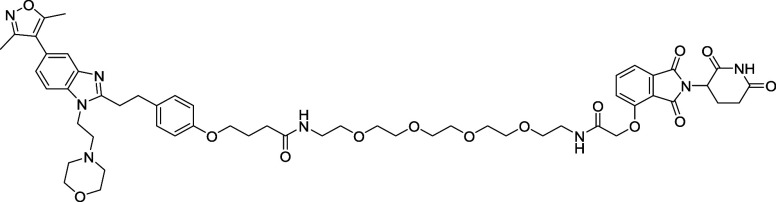



Degrader **14** was synthesized according to General Procedure A, using thalidomide 4′-oxyacetamide-PEG4-amine. Purification by flash column chromatography and elution with 0–20% methanol (with 0.5% NH_4_OH) in dichloromethane over 20 cvs, afforded the title compound as a colorless oil (25 mg, 55%). ^1^H NMR (600 MHz, CDCl_3_): δ 9.51 (br s, 1H), 7.75–7.62 (m, 3H), 7.53 (d, *J* = 7.3 Hz, 1H), 7.36 (d, *J* = 8.1 Hz, 1H), 7.17–7.09 (m, 3H), 6.79 (d, *J* = 8.1 Hz, 2H), 6.65–6.60 (m, 1H), 5.55–5.35, (m, 2H), 4.97–4.93 (m, 1H), 4.62 (s, 2H), 4.17–4.10 (m, 2H), 3.95 (t, *J* = 6 Hz, 2H), 3.71–3.52 (m, 18H), 3.45–3.40 (m, 2H), 3.23–3.15 (m, 4H), 2.89–2.68 (m, 4H), 2.61 (t, *J* = 7 Hz, 2H), 2.52–2.46 (m, 4H), 2.42 (s, 3H), 2.37 (t, *J* = 7 Hz, 2H), 2.29 (s, 3H), 2.17–2.03 (m, 5H). ^13^C NMR (151 MHz, CDCl_3_): δ 172.8, 171.5, 168.6, 166.9, 166.8, 166.0, 165.2, 159.1, 157.7, 155.5, 154.6, 137.12, 134.2, 133.8, 132.9, 129.5, 124.6, 123.7, 119.9, 119.5, 118.1, 117.4, 117.1, 114.8, 109.6, 70.6, 70.5, 70.45, 70.43, 70.3, 70.1, 70.1, 69.9, 67.9, 67.2, 66.8, 57.6, 54.7, 49.5, 39.3, 39.1, 33.2, 32.8, 31.6, 29.9, 25.4, 22.8, 22.7, 11.7, 11.0; LCMS (5–95% MeCN over 20 min) *t*_R_ = 11.92, Purity >95%, *m*/*z*(ES+): 1087.35 [M+Na^+^]^+^; HRMS (*m*/*z*): [M+Na^+^]^+^ calculated for C_55_H_68_N_8_NaO_14_, 1087.4753; found, 1087.4664.

##### (2*S*,4*R*)-1-((*S*)-2-(*tert*-Butyl)-19-(4-(2-(5-(3,5-Dimethylisoxazol-4-yl)-1-(2-morpholinoethyl)-1*H*-benzo[*d*]imidazol-2-yl)ethyl)phenoxy)-4,16-dioxo-6,9,12-trioxa-3,15-diazanonadecan-1-oyl)-4-hydroxy-*N*-(4-(4-methylthiazol-5-yl)benzyl)pyrrolidine-2-carboxamide (**15**)

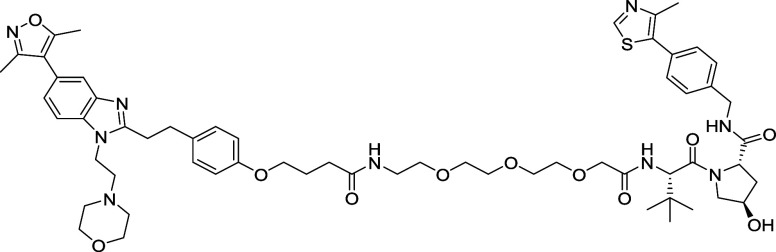



Degrader **15** was synthesized according to General Procedure A, using VH 032 amide-PEG3-amine. Purification by flash column chromatography and elution with 0–20% methanol (with 0.5% NH_4_OH) in dichloromethane over 20 cvs, afforded the title compound as a colorless oil (34 mg, 75%). ^1^H NMR (600 MHz, CDCl_3_): δ 8.65 (s, 1H), 7.60 (s, 1H), 7.48–7.43 (m, 1H), 7.36–7.32 (m, 4H), 7.29 (d, *J* = 9 Hz, 1H), 7.13–7.09 (m, 3H), 6.80 (d, *J* = 8 Hz, 2H), 6.58–6.52 (m, 1H), 5.71–5.50 (m, 2H), 4.67 (t, *J* = 8 Hz, 1H), 4.58–4.51 (m, 3H), 4.36–4.31 (m, 1H), 4.11 (t, *J* = 7 Hz, 2H), 4.05–3.97 (m, 3H), 3.95 (t, *J* = 6 Hz, 2H), 3.70–3.43 (m, 13H), 3.41–3.36 (m, 2H), 3.21–3.13 (m, 4H), 2.59 (t, *J* = 7 Hz, 2H), 2.49 (s, 3H), 2.47–2.43 (m, 3H), 2.41 (s, 3H), 2.35 (t, *J* = 7 Hz, 2H), 2.30–2.24 (m, 4H), 2.14–2.03 (m, Hz, 3H), 1.19 (t, *J* = 7 Hz, 2H), 0.95 (s, 9H). ^13^C NMR (151 MHz, CDCl_3_): δ 172.8, 171.1, 170.3, 165.1, 159.1, 157.6, 155.5, 150.4, 148.5, 143.1, 138.3, 134.4, 133.1, 131.7, 131.0, 129.6, 129.4, 128.2, 124.3, 123.5, 120.0, 117.2, 114.8, 109.6, 70.9, 70.6, 70.5, 70.1, 70.0, 67.2, 66.9, 65.9, 58.8, 57.7, 57.0, 56.9, 54.1, 43.3, 41.6, 39.4, 36.5, 35.5, 33.1, 32.8, 30.0, 26.5, 25.3, 16.1, 15.4, 11.7, 11.0. LCMS (5–95% MeCN over 20 min) *t*_R_ = 7.18, Purity >95%, *m*/*z* (ES+): 1134.50 [M+H^+^]^+^; HRMS (*m*/*z*): [M+Na^+^]^+^ calculated for C_60_H_79_N_9_NaO_11_S^+^, 1156.5512; found, 1156.5388.

##### (2*S*,4*R*)-1-((*S*)-2-(*tert*-Butyl)-22-(4-(2-(5-(3,5-dimethylisoxazol-4-yl)-1-(2-morpholinoethyl)-1*H*-benzo[*d*]imidazol-2-yl)ethyl)phenoxy)-4,19-dioxo-6,9,12,15-tetraoxa-3,18-diazadocosan-1-oyl)-4-hydroxy-*N*-(4-(4-methylthiazol-5-yl)benzyl)pyrrolidine-2-carboxamide (**16**)

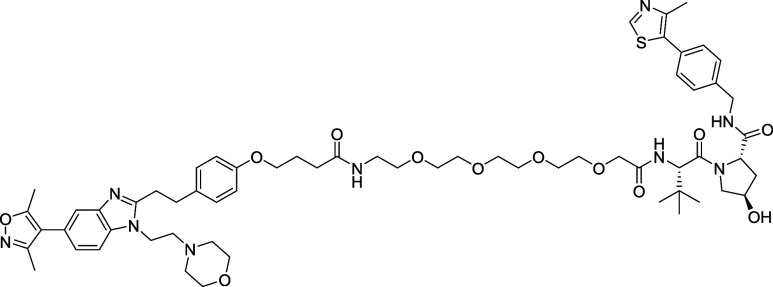



Degrader **16** was synthesized according to General Procedure A, using VH 032 amide-PEG4-amine. Purification by flash column chromatography and elution with 0–20% methanol (with 0.5% NH_4_OH) in dichloromethane over 20 cvs, afforded the title compound as a colorless oil (28 mg, 65%). ^1^H NMR (600 MHz, CDCl_3_): δ 8.65 (s, 1H), 7.60 (s, 1H), 7.39 (t, *J* = 6 Hz, 1H), 7.36–7.30 (m, 4H), 7.26 (s, 2H), 7.09–7.13 (m, 3H), 6.80 (d, *J* = 8 Hz, 2H), 6.50 (br s, 1H), 5.75–5.45 (m, 2H), 4.70 (t, *J* = 8 Hz, 1H), 4.55–4.50 (m, 3H), 4.37–4.31 (m, 1H), 4.12 (d, *J* = 7 Hz, 2H), 4.03–3.88 (m, 4H), 3.70–3.50 (m, 18H), 3.41 (q, *J* = 5 Hz, 2H), 3.19–3.11 (m, 4H), 2.59 (t, *J* = 7 Hz, 2H), 2.49 (s, 3H), 2.48–2.42 (m, 4H), 2.41 (s, 3H), 2.36 (t, *J* = 7 Hz, 2H), 2.28 (s, 3H), 2.15–2.04 (m, 4H), 0.94 (s, 9H). ^13^C NMR (151 MHz, CDCl_3_): δ 172.7, 171.3, 171.1, 170.2, 165.1, 159.1, 157.6, 155.5, 150.4, 148.5, 143.1, 138.3, 134.4, 133.1, 131.7, 131.0, 129.6, 129.4, 128.2, 124.3, 123.5, 120.0, 117.2, 114.8, 109.6, 71.0, 70.6, 70.6, 70.5, 70.2, 70.1, 70.0, 67.2, 66.9, 58.7, 57.7, 57.1, 56.9, 54.1, 43.3, 41.6, 39.3, 36.3, 35.4, 33.1, 32.8, 30.0, 26.5, 25.3, 16.1, 15.4, 11.7, 11.0. LCMS (5–95% MeCN over 20 min) *t*_R_ = 7.16, Purity >96%, *m*/*z* (ES+): 1178.55 [M+H^+^]^+^; HRMS (*m*/*z*): [M+Na^+^]^+^ calculated for C_62_H_83_N_9_NaO_12_S^+^, 1200.5780; found, 1200.5796.

##### *tert*-Butyl 4-(2-((4-Bromo-2-nitrophenyl)amino)ethyl)piperazine-1-carboxylate (**17**)

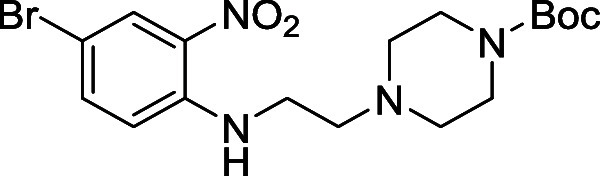



A microwave vial was equipped with a magnetic flea and flushed with argon. 4-(2-aminoethyl)-1-Boc-piperazine (5.05 g, 22 mmol) was added followed by triethylamine (15 mL). This was stirred for 3 min before the addition of 4-fluoro-3-nitrobromobenzene (4.40 g, 20 mmol). Following the addition, the vial was sealed and heated using the dynamic heating method, with max power set to 300 W, max pressure 300 psi, max temperature 125 °C, high stirring throughout, and power max turned off. This method was used to hold the temperature at 125 °C for 10 min. After cooling, the reaction mixture was transferred to a separating funnel where it was partitioned between water (250 mL) and ethyl acetate (200 mL). The organic phase was separated, and the aqueous component was extracted with ethyl acetate (3 × 75 mL). The combined organic extracts were washed with saturated aqueous sodium hydrogen carbonate solution (200 mL) and brine (200 mL), and then dried over anhydrous magnesium sulfate, and concentrated under reduced pressure to afford the title compound as an orange solid (8.40 g, 98%). ^1^H NMR (600 MHz,CDCl_3_): δ 8.51 (s, 1H), 8.32 (d, *J* = 2 Hz, 1H), 7.49 (dd, *J* = 9, 2 Hz, 1H), 6.73 (d, *J* = 9 Hz, 1H), 3.50–3.45 (m, 4H), 3.34 (q, *J* = 6 Hz, 2H), 2.73 (t, *J* = 6 Hz, 2H), 2.50–2.4 (m, 4H), 1.46 (s, 9H). ^13^C NMR (151 MHz, CDCl_3_): δ 154.9, 144.3, 139.0, 132.5, 129.1, 115.9, 106.4, 79.9, 55.6, 52.7, 39.8, 28.6. LCMS (5–95% MeCN over 20 min) *t*_R_ = 7.94 min, Purity >99%, *m*/*z* (ES+): 429.00 [M+H^+^]^+^; HRMS-ESI (*m*/*z*): [M+H^+^]^+^ calculated for C_17_H_26_BrN_4_O_4_, 429.1132; found, 429.1132.

##### *tert*-Butyl-4-(2-((4-(3,5-dimethylisoxazol-4-yl)-2-nitrophenyl)amino)ethyl)piperazine-1-carboxylate (**18**)

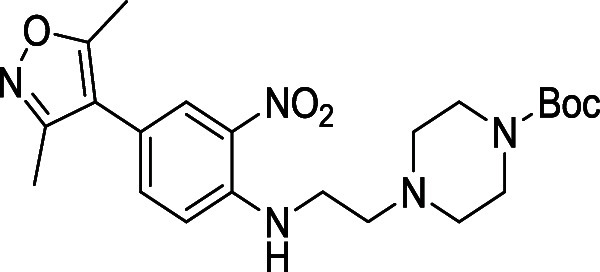



A mixture of **17** (8.40 g, 19.60 mmol), potassium phosphate (10.82 g, 50.96 mmol), PdCl_2_(dppf)•DCM (0.80 g, 0.98 mmol), and 3,5-dimethylisoxazol-4-boronic acid pinacol ester (4.90 g, 21.95 mmol) in 1,4-dioxane (200 mL) was degassed and backfilled with argon. The reaction was then heated to reflux and stirred overnight. After cooling, the reaction mixture was filtered through diatomaceous earth and concentrated under reduced pressure. Purification by flash column chromatography and elution with 0–100% ethyl acetate in hexane, afforded the title compound as an orange oil (6.99 g, 80%).^1^H NMR (600 MHz, CDCl_3_): δ 8.58 (s, 1H), 8.08 (d, *J* = 2 Hz, 1H), 7.34 (dd, *J* = 8.8, 2 Hz, 1H), 6.91 (d, *J* = 8.8 Hz, 1H), 3.52–3.46 (m, 4H), 3.41 (q, *J* = 5.8 Hz, 2H), 2.76 (t, *J* = 6.1 Hz, 2H), 2.52–2.45 (m, 4H), 2.40 (s, 3H), 2.26 (s, 3H), 1.47 (s, 9H). ^13^C NMR (151 MHz, CDCl_3_): δ 165.5, 158.7, 154.9, 144.6, 136.9, 132.1, 127.2, 117.5, 115.1, 114.9, 79.9, 55.7, 52.7, 44.4, 43.4, 39.8, 28.6, 11.7, 10.9. LCMS (5–95% MeCN over 20 min) *t*_R_ = 7.61 min, Purity >99%, *m*/*z* (ES+): 446.50 [M+H^+^]^+^; HRMS-ESI (*m*/*z*): [M+H^+^]^+^ calculated for C_22_H_32_N_5_O_5_, 446.2398; found, 446.2421.

##### *tert*-Butyl-4-(2-((2-amino-4-(3,5-dimethylisoxazol-4-yl)phenyl)amino)ethyl)piperazine-1-carboxylate (**19**)

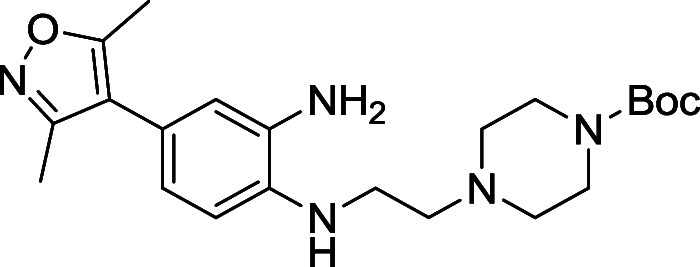



To a solution of **18** (1.50 g, 3.47 mmol) in EtOH (55 mL) was added 1 M aqueous sodium dithionite solution (55 mL), and the reaction was heated to 80 °C for 1 h. Upon cooling, the reaction mixture was treated with 10% ammonia solution (55 mL) and ethyl acetate (75 mL). The organic phase was separated, and the aqueous component was extracted with ethyl acetate (3 × 30 mL). The combined organics were washed with brine (3 × 100 mL), dried over anhydrous magnesium sulfate, and concentrated under reduced pressure to afford the title compound as a yellow oil, which was used directly in the next step without further purification or manipulation (1.20 g, 83%).

##### 3-(4-(2-(5-(3,5-Dimethylisoxazol-4-yl)-1-(2-(piperazin-1-yl)ethyl)-1*H*-benzo[*d*]imidazol-2-yl)ethyl)phenoxy)-*N*,*N*-dimethylpropan-1-amine (**21**)

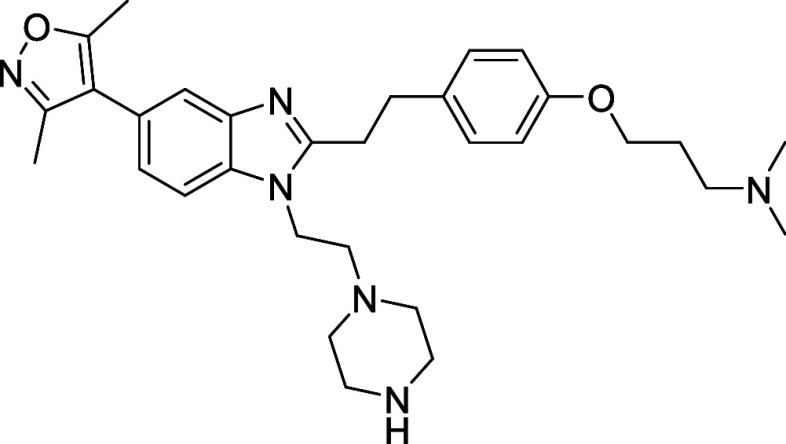



To a suspension of **20**(44) (0.818 g, 3.18 mmol) in DMF (10 mL), was added triethylamine (0.8 mL, 5.78 mmol) and HATU (1.43 g, 3.76 mmol). The reaction vessel was flushed with argon and left to stir for 1 h before the addition of **19** (1.20 g, 3.76 mmol) in DMF (10 mL). The reaction was then left to stir at ambient temperature overnight. The reaction mixture was then partitioned between ethyl acetate (50 mL) and water (50 mL). The organic phase was separated and washed with water (4 × 150 mL), saturated aqueous sodium hydrogen carbonate solution (100 mL) and brine (100 mL), dried over anhydrous magnesium sulfate, and concentrated under reduced pressure. Purification by flash column chromatography and elution with 0–20% 7N methanolic ammonia solution in dichloromethane, afforded the diamine intermediate. This was dissolved in methanol (20 mL) before the addition of HCl (2.6 mL, 10.5 mmol, 4 M solution in 1,4-dioxane), and the reaction was heated at reflux overnight. The reaction mixture was cooled and concentrated under reduced pressure before addition of dichloromethane (50 mL) and saturated aqueous sodium hydrogen carbonate solution (50 mL) solution. After stirring vigorously for 10 min, the organic phase was separated. The aqueous component was extracted with dichloromethane (3 × 50 mL), and the combined organic extracts were washed with brine, dried over anhydrous magnesium sulfate, and concentrated under reduced pressure. Purification by flash column chromatography and elution with 0–20% 7N methanolic ammonia solution in dichloromethane, afforded the title compound as a beige solid (564 mg, 37%). ^1^H NMR (600 MHz, CDCl_3_): δ 7.62 (s, 1H), 7.34 (d, *J* = 8.2 Hz, 1H), 7.15–7.09 (m, 3H), 6.84 (d, *J* = 8.2 Hz, 2H), 4.11 (t, *J* = 6.9 Hz, 2H), 3.99 (d, *J* = 6.4 Hz, 2H), 3.25–3.15 (m, 4H), 2.88–2.83 (m, 4H), 2.59 (t, *J* = 6.9 Hz, 2H), 2.49–2.43 (m, 5H), 2.42 (s, 3H), 2.33–2.39 (m, 4H), 2.27 (s, 6H), 2.01 (br s, 1H), 1.98–1.93 (m, 2H). ^13^C NMR (151 MHz, CDCl_3_): δ 165.1, 159.2, 157.8, 155.7, 143.2, 134.5, 133.0, 129.4, 124.3, 123.5, 120.0, 117.3, 114.8, 109.6, 66.4, 58.0, 56.5, 55.0, 46.1, 45.5, 41.7, 33.2, 30.1, 27.6, 11.7, 11.0. LCMS (5–95% MeCN over 20 min) *t*_R_ = 7.34 min, Purity >99%, *m*/*z* (ES+): 531.4 [M+H^+^]^+^; HRMS-ESI (*m*/*z*): [M+H^+^]^+^ calculated for C_31_H_43_N_6_O_2_, 531.3447; found, 531.3443.

##### *tert*-Butyl-4-(2-(2-(4-(3-(dimethylamino)propoxy)phenethyl)-5-(3,5-dimethylisoxazol-4-yl)-1*H*-benzo[*d*]imidazol-1-yl)ethyl)piperazine-1-carboxylate (**22**)

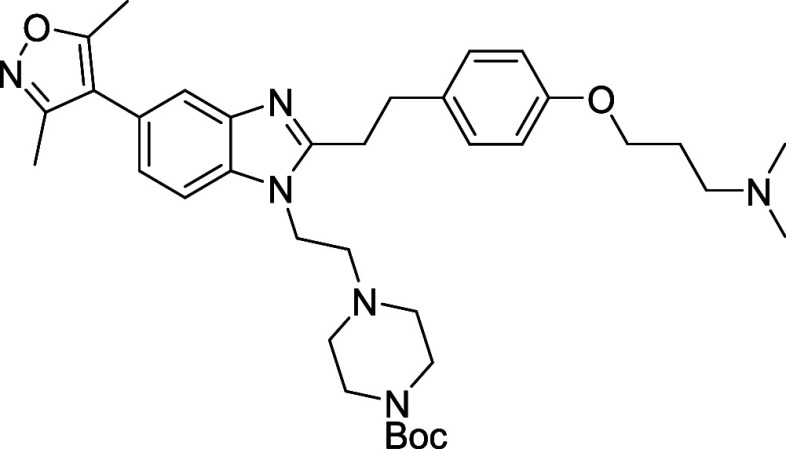



To a solution of di-*tert*-butyl dicarbonate (58 mg, 0.265 mmol) and DMAP (4.6 mg, 0.038 mmol) in dichloromethane (5 mL) was added a dropwise a solution of **21** (100 mg, 0.189 mmol) in dichloromethane (3 mL) and triethylamine (0.131 mL, 0.945 mmol), and the resulting mixture was stirred overnight. After this time, water (10 mL) was added, and the organic phase was separated. The aqueous component was extracted with dichloromethane (5 × 10 mL), and the combined organic extracts were washed with brine (100 mL), dried over anhydrous magnesium sulfate, and concentrated under reduced pressure. Purification by flash column chromatography and elution with 0–10% (7N ammonia in methanol) in dichloromethane, afforded the title compound as a colorless oil (52 mg, 43%). ^1^H NMR (600 MHz, CDCl_3_): δ 7.62 (s, 1H), 7.33 (d, *J* = 8.1 Hz, 1H), 7.15–7.09 (m, 3H), 6.83 (d, *J* = 8.1 Hz, 2H), 4.11 (d, *J* = 6.8 Hz, 2H), 4.00 (t, *J* = 6 Hz, 2H), 3.45–3.33 (m, 4H), 3.24–3.18 (m, 2H), 3.18–3.13 (m, 2H), 2.70–2.59 (m, 4H), 2.45–2.35 (m, 13H), 2.29 (s, 3H), 2.07 (q, *J* = 7 Hz, 2H), 1.44 (s, 9H). ^13^C NMR (151 MHz, CDCl_3_): δ 165.1, 159.1, 157.5, 155.5, 154.7, 143.1, 134.4, 133.1, 129.5, 129.4, 124.3, 123.5, 120.0, 117.2, 114.7, 114.5, 109.5, 80.0, 65.9, 57.3, 56.4, 45.0, 41.7, 33.1, 31.7, 30.1, 30.1, 28.5, 26.9, 22.8, 11.7, 11.1. LCMS (5–95% MeCN over 20 min) *t*_R_ = 7.38 min, Purity >99%, *m*/*z* (ES+): 631.45 [M+H^+^]^+^; HRMS-ESI (*m*/*z*): [M+H^+^]^+^ calculated for C_36_H_51_N_6_O_4_, 631.3972; found, 631.3998.

##### 1-(4-(2-(2-(4-(3-(Dimethylamino)propoxy)phenethyl)-5-(3,5-dimethylisoxazol-4-yl)-1*H*-benzo[*d*]imidazol-1-yl)ethyl)piperazin-1-yl)ethanone (**23**)

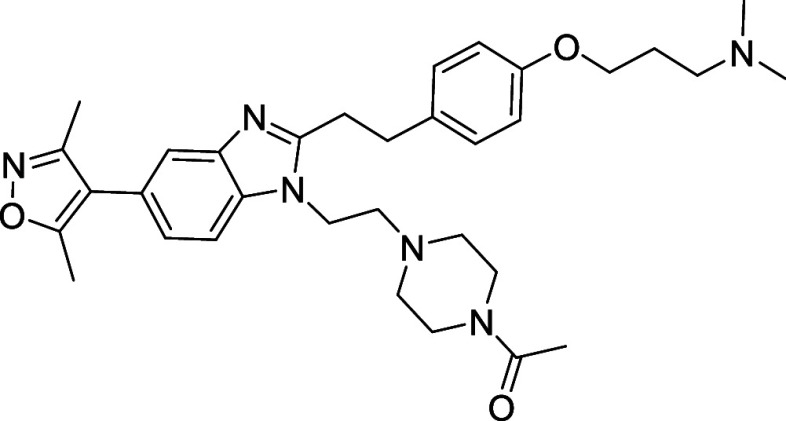



To a suspension of **19** (0.918 g, 3.57 mmol) in DMF (20 mL), was added triethylamine (1.36 mL, 9.75 mmol) and HATU (1.61 g, 4.23 mmol). The reaction vessel was degassed and backfilled with argon and left to stir for 1 h before the addition of a solution of **20** (1.35 g, 3.25 mmol) in DMF (30 mL). The reaction was then left to stir at ambient temperature overnight before being partitioned between ethyl acetate (50 mL) and water (50 mL). The organic phase was separated and washed with water (4 × 150 mL), saturated aqueous sodium hydrogen carbonate solution (100 mL), and brine (100 mL), dried over anhydrous magnesium sulfate, and concentrated under reduced pressure. Purification by flash column chromatography and elution with 0–20% (7N ammonia in methanol) in dichloromethane, afforded the diamine intermediate, which was dissolved in acetic acid and heated at reflux overnight. The reaction mixture was cooled, concentrated, and suspended in dichloromethane before neutralizing with saturated aqueous sodium hydrogen carbonate solution. The organic phase was separated, and the aqueous component was extracted with dichloromethane (4 × 50 mL). The combined organic extracts were washed with brine, dried over anhydrous magnesium sulfate, and concentrated under reduced pressure. Purification by flash column chromatography and elution with 0–20% (7N ammonia in methanol) in dichloromethane, afforded the title compound as a pale brown oil (640 mg, 34%). ^1^H NMR (600 MHz, CDCl_3_): δ 7.64–7.61 (m, 1H), 7.35–7.31 (m, 1H), 7.15–7.09 (m, 3H), 6.85–6.80 (m, 2H), 4.13–4.09 (m, 2H), 4.01–3.97 (m, 2H), 3.56–3.48 (m, 2H), 3.39–3.30 (m, 2H), 3.24–3.13 (m, 4H), 2.63–2.61 (m, 2H), 2.46–2.41 (m, 7H), 2.32–2.28 (m, 12H), 2.00–1.94 (m, 4H); ^13^C NMR (151 MHz, CDCl_3_): δ 165.2, 160.8, 159.1, 157.8, 155.5, 143.2, 134.3, 133.0, 129.5, 129.4, 124.4, 123.6, 120.1, 117.2, 114.8, 114.5, 109.5, 66.3, 57.2, 56.4, 54.3, 52.9, 45.6, 45.4, 41.8, 39.9, 33.2, 30.2, 27.4, 11.8, 11.1; LCMS (5–95% MeCN over 20 min) *t*_R_ = 6.97 min, purity >96%; *m*/*z* (ES+): 573.55 [M+H^+^]^+^; HRMS-ESI (*m*/*z*): [M+H^+^]^+^ calculated for C_33_H_45_N_6_O_3_, 573.3553; found, 573.3546.

##### 4-(3,5-Dimethylisoxazol-4-yl)-2-nitro-*N*-(2-(piperazin-1-yl)ethyl)aniline (**24**)

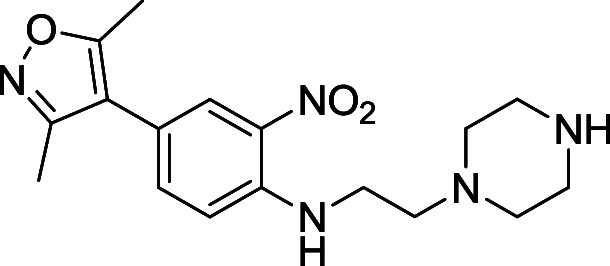



To a stirred solution of **17** (2.8 g, 6.3 mmol) in dichloromethane (200 mL) was added TFA (20 mL, 26.2 mmol), and the reaction was left to stir at ambient temperature overnight. The reaction mixture was concentrated under reduced pressure, and reconcentrated from dichloromethane (5 × 50 mL), affording the crude product as the TFA salt. This was partitioned between dichloromethane (50 mL) and saturated aqueous sodium hydrogen carbonate solution. The organic phase was separated, and the aqueous component was extracted with dichloromethane (5 × 50 mL). The combined organic extracts were washed with brine (2 × 100 mL), dried over anhydrous magnesium sulfate, and concentrated under reduced pressure affording the title compound as a red oil (2.2 g, 99%). ^1^H NMR (600 MHz, CDCl_3_): δ 8.58 (s, 1H), 8.09 (s, 1H), 7.34 (d, *J* = 9 Hz, 1H), 6.91 (d, *J* = 9 Hz, 1H), 3.40 (q, *J* = 6 Hz, 2H), 3.00–2.93 (m, 4H), 2.75 (t, *J* = 6.1 Hz, 2H), 2.60–2.50 (m, 4H), 2.40 (s, 3H), 2.35 (br s, 1H), 2.26 (s, 3H). ^13^C NMR (151 MHz, CDCl_3_): δ 165.5, 158.7, 144.6, 136.9, 132.1, 117.4, 115.1, 114.9, 56.2, 53.8, 46.1, 39.7, 11.7, 10.9. LCMS (5–95% MeCN over 20 min) *t*_R_ = 10.56 min, Purity >99%, *m*/*z* (ES+): 345.95 [M+H^+^]^+^; HRMS-ESI (*m*/*z*): [M+H^+^]^+^ calculated for C_17_H_24_N_5_O_3_, 346.1874; found, 346.1859.

##### *tert*-Butyl-2-(4-(2-((4-(3,5-dimethylisoxazol-4-yl)-2-nitrophenyl)amino)ethyl)piperazin-1-yl)acetate (**25**)

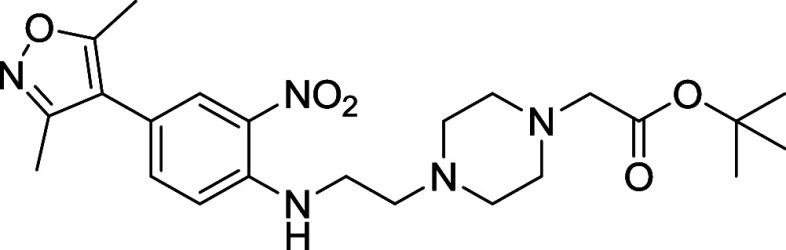



To a stirred solution of **24** (2.1 g, 6.3 mmol) in dichloromethane (100 mL) was added DIPEA (4.40 mL, 25.2 mmol) followed by *tert*-butylbromoacetate (1.11 mL, 7.56 mmol), and the resulting solution was left to stir overnight at ambient temperature. The reaction mixture was washed with water (50 mL), saturated aqueous sodium hydrogen carbonate solution (50 mL) and brine (100 mL), before being dried over anhydrous magnesium sulfate and concentrated under reduced pressure. Purification by flash column chromatography and elution with 0–100% ethyl acetate in hexane, afforded the title compound as an orange oil (2.31 g 80%). ^1^H NMR (600 MHz, CDCl_3_): δ 8.57 (s, 1H), 8.08 (s, 1H), 7.33 (d, *J* = 8.5 Hz, 1H), 6.91 (d, *J* = 8.5 Hz, 1H), 3.39 (q, *J* = 6 Hz, 2H), 3.13 (s, 2H), 2.76 (t, *J* = 6 Hz, 2H), 2.73–2.52 (s, 8H), 2.40 (s, 3H), 2.26 (s, 3H), 1.47 (s, 9H). ^13^C NMR (151 MHz, CDCl_3_): δ 169.6, 165.3, 158.6, 144.5, 136.7, 131.9, 127.0, 117.2, 115.0, 114.7, 81.1, 59.9, 55.5, 53.1, 52.5, 39.7, 28.2, 11.6, 10.8. LCMS (5–95% MeCN over 20 min) *t*_R_ = 12.59 min, Purity >99%, *m*/*z* (ES+): 460.10 [M+H^+^]^+^; HRMS-ESI (*m*/*z*): [M+H^+^]^+^ calculated for C_23_H_34_N_5_O_5_^+^, 460.2554; found, 460.2542.

##### *tert*-Butyl-2-(4-(2-((2-amino-4-(3,5-dimethylisoxazol-4-yl)phenyl)amino) ethyl)piperazin-1-yl)acetate (**26**)

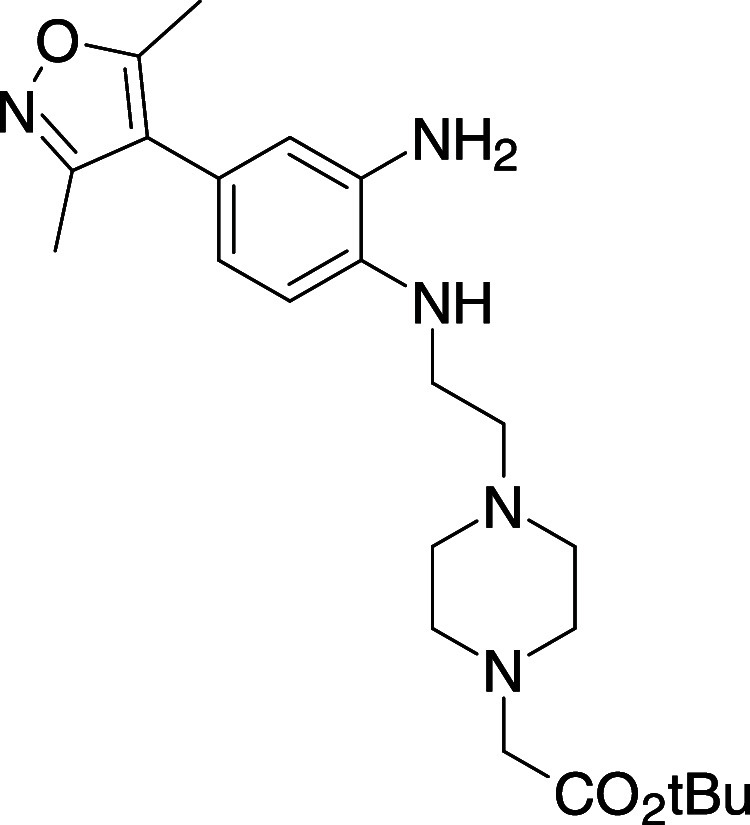



To a suspension of **25** (2.30 g, 5.01 mmol) in ethanol (75 mL) was added 1 M aqueous sodium dithionite solution (75 mL), and the reaction was heated to 80 °C for 1 h. Upon cooling, the reaction mixture was partitioned between 10% ammonium hydroxide solution (75 mL) and ethyl acetate (75 mL). The organic phase was separated, and the aqueous component was extracted with ethyl acetate (3 × 25 mL). The combined organic extracts were washed with brine (100 mL), dried over anhydrous magnesium sulfate, and concentrated under reduced pressure to give the title compound as a yellow oil, which was used directly in the next step without any further purification (1.36 g, 70%). ^1^H NMR (600 MHz, CDCl_3_): δ 6.68 (s, 2H), 6.58 (s, 1H), 3.48 (br s, 2H), 3.20 (t, *J* = 6 Hz, 2H), 3.12 (s, 2H), 2.71 (t, *J* = 6 Hz, 2H), 2.65–2.50 (m, 8H), 2.37 (s, 3H), 2.25 (s, 3H), 1.47 (s, 9H). ^13^C NMR (151 MHz, CDCl_3_): δ 169.5, 164.4, 159.0, 137.1, 134.6, 121.2, 120.2, 116.8, 116.5, 111.6, 81.1, 59.6, 56.7, 53.00, 52.8, 40.6, 28.1, 11.5, 10.8. LCMS (30–95% MeCN over 20 min) *t*_R_ = 17.02 min, Purity >95%, *m*/*z* (ES+): 430.05 [M+H^+^]^+^; HRMS-ESI (*m*/*z*): [M+Na^+^]^+^ calculated for C_23_H_35_N_5_NaO_3_^+^, 452.2632; found, 452.2638.

##### 2-(4-(2-(2-(4-(3-(Dimethylamino)propoxy)phenethyl)-5-(3,5-dimethylisoxazol-4-yl)-1*H*-benzo[*d*]imidazol-1-yl)ethyl)piperazin-1-yl)acetic Acid Dihydrochloride (**27**)

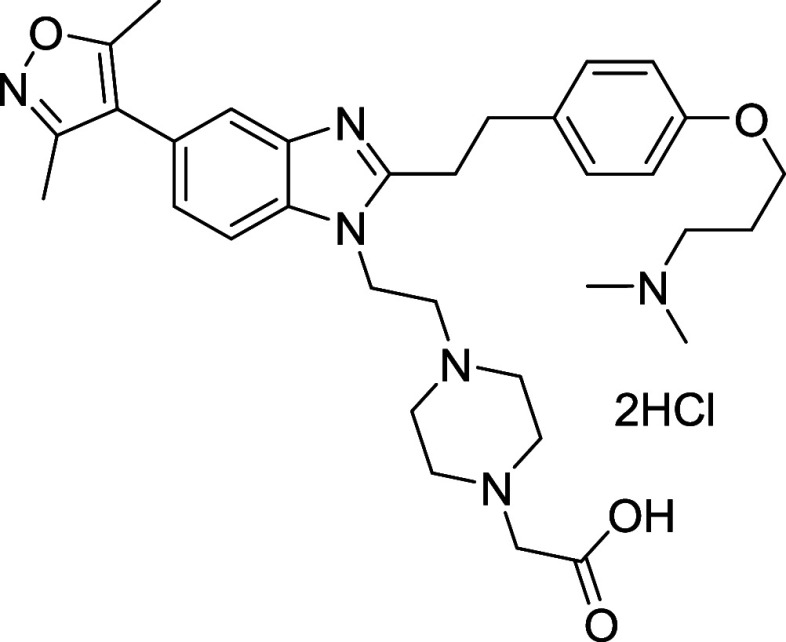



To a stirred suspension of **20** (3.3 g, 12.8 mmol) in DMF (30 mL), was added triethylamine (3.23 mL, 23.2 mmol) and HATU (5.7 g, 15.1 mmol). The reaction vessel was flushed with argon and left to stir for 1 h before the addition of a solution of **26** (5 g, 11.6 mmol) in DMF (30 mL), and the reaction mixture was then left to stir at ambient temperature overnight. The reaction mixture was partitioned between dichloromethane (150 mL) and water (150 mL). The organic phase was separated and washed with water (4 × 150 mL), saturated aqueous sodium hydrogen carbonate solution (150 mL), and brine (150 mL), dried over anhydrous magnesium sulfate, and concentrated under reduced pressure. Purification by flash column chromatography and elution with 3–5% methanol (with up to 0.5% NH_4_OH) in dichloromethane, produced a pale brown gum (2.59 g, 3.908 mmol). The residue was then dissolved in acetic acid (50 mL) and heated to reflux for 1.5 h, concentrated under reduced pressure, and reconcentrated from heptane (5 × 75 mL). The brown gum was dissolved in anhydrous ethyl acetate (60 mL) and purged with nitrogen (g). To this stirring solution was added hydrogen chloride (6 mL, 11.72 mmol, 2 M solution in diethyl ether) and a solid immediately formed. Excess diethyl ether was added, and the solution was left to stir overnight at ambient temperature. The precipitate was then collected by filtration, dried under vacuum, and freeze-dried, affording the title compound as an off-white solid (3.04 g, 36%). ^1^H NMR (600 MHz, *d*_6_-DMSO): δ 11.03 (br s, 1H), 8.22 (d, *J* = 8.2 Hz, 1H), 7.79 (s, 1H), 7.57 (d, *J* = 8.2 Hz, 1H), 7.33 (d, *J* = 9 Hz, 2H), 6.89 (d, *J* = 9 Hz, 2H), 4.85 (br s, 2H), 4.10–3.83 (m, 5H), 3.76–3.09 (m, 15H), 2.75–2.70 (m, 6H), 2.42 (s, 3H), 2.24 (s, 3H), 2.10–2.17 (m, 2H). ^13^C NMR (151 MHz, *d*_6_-DMSO): δ 165.7, 158.3, 157.1, 154.8, 131.6, 131.1, 129.7, 127.6, 126.5, 115.5, 114.6, 113.5, 64.9, 55.0, 53.9, 42.0, 31.3, 27.3, 23.9, 11.4, 10.5. LCMS (30–95 MeCN over 20 min) *t*_R_ = 7.36 min, Purity >95%, *m*/*z* (ES+): 589.25 [M+H^+^]^+^; HRMS-ESI (*m*/*z*): [M+H^+^]^+^ calculated for C_33_H_45_N_6_O_4_, 589.3502; found, 589.3577.

##### Methyl 2-(4-(2-(2-(4-(3-(Dimethylamino)propoxy)phenethyl)-5-(3,5-dimethylisoxazol-4-yl)-1*H*-benzo[*d*]imidazol-1-yl)ethyl)piperazin-1-yl)acetate Hydrochloride (**28**)

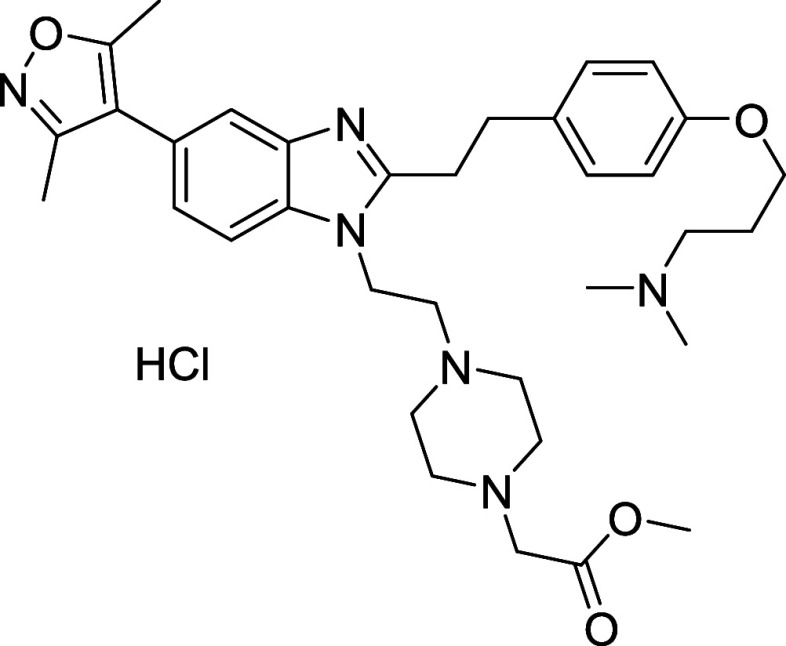



A solution of **27** (100 mg, 0.151 mmol) in methanol (5 mL) was treated with sulfuric acid (1 drop) and heated to reflux overnight. Upon cooling, the reaction mixture was concentrated under reduced pressure, partitioned between ethyl acetate (10 mL) and water (10 mL), and the biphasic mixture was treated with saturated aqueous sodium hydrogen carbonate solution (10 mL). The organic phase was separated and washed with brine, dried over anhydrous magnesium sulfate, and concentrated under reduced pressure. Purification by flash column chromatography and elution with 0–20% methanol in dichloromethane, afforded a colorless oil, which was dissolved in anhydrous ethyl acetate (10 mL) and purged with nitrogen (g). To this stirring solution was added hydrogen chloride (0.132 mL, 0.27 mmol, 2 M solution in diethyl ether), whereupon a solid immediately formed, excess diethyl ether was added and the solution was left to stir overnight at ambient temperature. The precipitate was then collected by filtration, affording the title compound as a beige solid (85 mg, 88%). ^1^H NMR (600 MHz, CDCl_3_): δ 7.62 (s, 1H), 7.34 (d, *J* = 8.5 Hz, 1H), 7.16–7.10 (m, 3H), 6.83 (d, *J* = 8.5 Hz, 2H), 4.10 (t, *J* = 7.0 Hz, 2H), 4.00 (t, *J* = 6.3 Hz, 2H), 3.72 (s, 3H), 3.24–3.19 (m, 4H), 3.18–3.14 (m, 2H), 2.62 (t, *J* = 7.0 Hz, 2H), 2.60–2.52 (m, 10H), 2.43 (s, 3H), 2.33 (s, 6H), 2.30 (s, 3H), 2.03–1.97 (m, 2H).^13^C NMR (151 MHz, CDCl_3_): δ 170.8, 165.2, 159.2, 157.7, 155.6, 143.2, 134.4, 133.1, 129.5, 124.3, 123.5, 120.0, 117.3, 114.8, 109.6, 66.2, 59.4, 57.3, 56.5, 53.5, 53.0, 51.9, 45.4, 41.7, 33.2, 30.1, 27.3, 11.8, 11.1. LCMS (30–95 MeCN over 20 min) *t*_R_ = 7.03 min, Purity >91%, *m*/*z* (ES+): 603.25 [M+H^+^]^;^ HRMS-ESI (*m*/*z*): [M+H^+^]^+^ calculated for C_34_H_47_N_6_O_4_, 603.3659; found, 603.3651.

##### 2-(4-(2-(2-(4-(3-(Dimethylamino)propoxy)phenethyl)-5-(3,5-dimethylisoxazol-4-yl)-1*H*-benzo[*d*]imidazol-1-yl)ethyl)piperazin-1-yl)-*N*-(2-methoxyethyl)acetamide Hydrochloride (**29**)

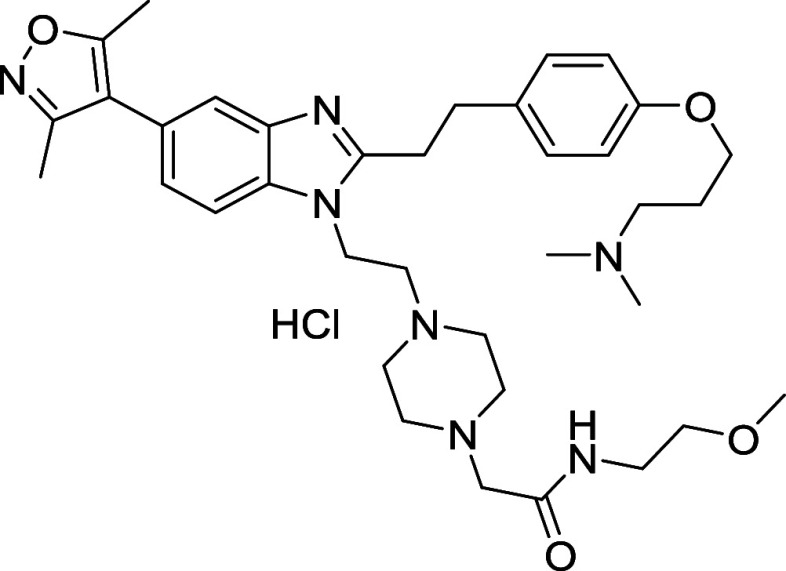



To a stirred solution of **27** (150 mg, 0.227 mmol) and HATU (112.2 mg, 0.295 mmol) in DMF (5 mL) was added triethylamine (95 μL, 0.681 mmol) followed by 2-methoxyethylamine (40 μL, 0.453 mmol) before leaving the reaction to stir at ambient temperature overnight. The mixture was then partitioned between dichloromethane (25 mL) and water (25 mL). The organic phase was separated and washed with saturated aqueous sodium hydrogen carbonate solution (25 mL), brine (3 × 10 mL), dried over anhydrous magnesium sulfate, and concentrated under reduced pressure. Purification by flash column chromatography and elution with 0–10% (7N ammonia in methanol) in dichloromethane, afforded the free base as a colorless oil. This was dissolved in anhydrous ethyl acetate (10 mL) and purged with nitrogen (g). To this stirring solution was added hydrogen chloride (0.17 mL, 0.33 mmol, 2 M solution in diethyl ether), whereupon a solid immediately formed. Excess diethyl ether was added, and the mixture was left to stir overnight at ambient temperature. The precipitate was collected by filtration to afford the title compound as a colorless solid (120 mg, 77%). ^1^H NMR (600 MHz, CDCl_3_): δ 7.62 (d, *J* = 1.5 Hz, 1H), 7.34 (d, *J* = 8.2 Hz, 2H), 7.15 (d, *J* = 8.6 Hz, 2H), 7.10–7.12 (m, 1H), 6.83 (d, *J* = 8.6 Hz, 2H), 4.11 (t, *J* = 6.7 Hz, 2H), 4.04 (t, *J* = 6.0 Hz, 2H), 3.48–3.42 (m, 4H), 3.34 (s, 3H), 3.23 (t, *J* = 7.0 Hz, 2H), 3.15–3.19 (m, 2H), 2.98 (s, 2H), 2.9–2.83 (m, 2H), 2.64 (t, *J* = 6.7 Hz, 2H), 2.57 (s, 6H), 2.55–2.45 (s, 8H), 2.42 (s, 3H), 2.29 (s, 3H), 2.20–2.12 (m, 2H). ^13^C NMR (151 MHz, CDCl_3_): δ 170.0, 165.0, 159.0, 157.2, 155.5, 143.1, 134.3, 133.4, 129.4, 124.2, 123.4, 119.9, 117.1, 114.6, 109.3, 71.3, 65.4, 61.4, 58.7, 57.1, 56.1, 53.6, 53.2, 44.3, 41.7, 38.6, 32.8, 29.8, 26.0, 22.6, 11.6, 10.9. LCMS (5–95% MeCN over 20 min) *t*_R_ = 8.69 min, Purity >96%, *m*/*z* (ES+): 646.30 [M+H^+^]^+^; HRMS-ESI (*m*/*z*): [M+H^+^]^+^ calculated for C_36_H_52_N_7_O_4_, 646.4081; found, 646.4094.

#### General Procedure B for Degrader Synthesis

A stirred suspension of **27** (150 mg, 0.227 mmol) in DMF (10 mL) was treated with the relevant commercially available amine-reactive degrader building block (E3 ligase ligand functionalized with a linker with amine termini) (1 equiv), triethylamine (221 μL, 1.587 mmol, 7 equiv) and HATU (112 mg, 0.295 mmol, 1.3 equiv) and stirred overnight at ambient temperature. The reaction was then diluted with water (100 mL) and extracted with ethyl acetate (3 × 25 mL). The combined organic extracts were washed with brine (50 mL), saturated aqueous sodium hydrogen carbonate solution (50 mL), and brine (2 × 50 mL), dried over anhydrous magnesium sulfate, and concentrated under reduced pressure.

##### 2-(4-(2-(2-(4-(3-(Dimethylamino)propoxy)phenethyl)-5-(3,5-dimethylisoxazol-4-yl)-1*H*-benzo[*d*]imidazol-1-yl)ethyl)piperazin-1-yl)-*N*-(2-((2-(2,6-dioxopiperidin-3-yl)-1,3-dioxoisoindolin-4-yl)amino)ethyl)acetamide (**30**)

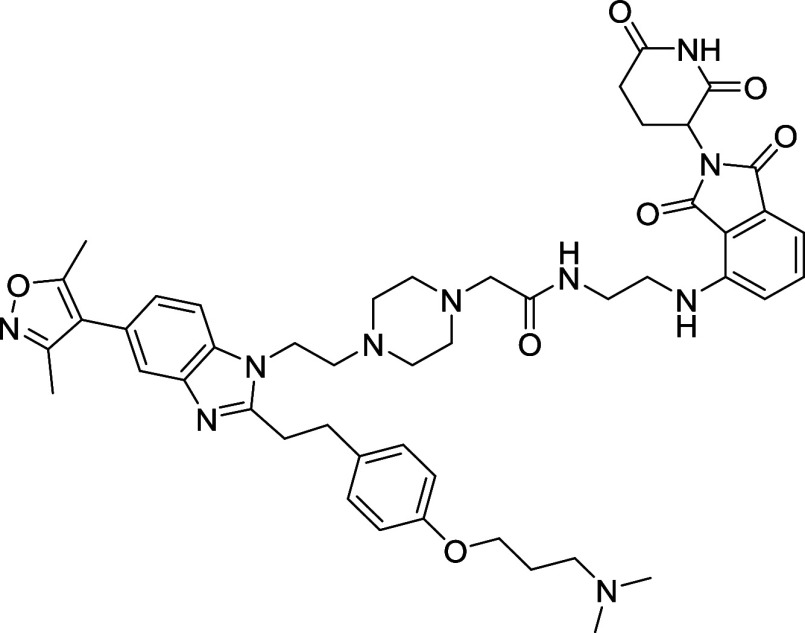



Degrader **30** was synthesized according to General Procedure B using pomalidomide 4′-alkylC2-amine. Purification by flash column chromatography and elution with 2–6% (7N ammonia in methanol) in dichloromethane, followed by lyophilization, afforded the title compound as a yellow solid (72 mg, 36%). ^1^H NMR (600 MHz, *d*_6_-DMSO): δ 11.11 (br s, 1H), 7.90 (t, *J* = 6 Hz, 1H), 7.58–7.54 (m, 2H), 7.21 (d, *J* = 9 Hz, 2H), 7.16 (dd, *J* = 8, 2 Hz, 1H), 7.02 (d, *J* = 7 Hz, 1H), 6.84 (d, *J* = 9 Hz, 2H), 6.71–6.66 (m, 1H), 5.05 (dd, *J* = 13, 5 Hz, 1H), 4.24 (t, *J* = 6 Hz, 2H), 3.93 (t, *J* = 6 Hz, 2H), 3.40–3.27 (m, 12H), 3.14–3.18 (m, 4H), 2.90–2.80 (m, 3H), 2.57–2.50 (m, 4H), 2.47–2.39 (m, 4H), 2.37–2.30 (m, 4H), 2.23 (s, 3H), 2.12 (s, 6H), 1.82–1.78 (m, 2H).^13^C NMR (151 MHz, *d*_6_-DMSO): δ 172.8, 170.1, 169.7, 168.7, 167.3, 164.5, 158.4, 157.0, 155.6, 146.4, 142.6, 136.2, 134. 5, 133.0, 132.2, 129.4, 122.7, 122.6, 122.6, 118.9, 117.3, 116.7, 114.3, 110.6, 110.3, 109.2, 65.7, 61.2, 57.1, 55.7, 52.9, 52.8, 48.5, 45.2, 41.3, 40.7, 40.0, 37.7, 31.8, 31.0, 28.6, 27.0, 22.2, 11.4, 10.6. LCMS (5–95% MeCN over 5 min) *t*_R_ = 3.212 min, Purity = 99%, *m*/*z* (ES+): 887.40 [M+H^+^]^+^; HRMS-ESI (*m*/*z*): [M+H^+^]^+^ calculated for C_48_H_59_N_10_O_7_, 887.4568; found, 887.4599.

##### 2-(4-(2-(2-(4-(3-(Dimethylamino)propoxy)phenethyl)-5-(3,5-dimethylisoxazol-4-yl)-1*H*-benzo[*d*]imidazol-1-yl)ethyl)piperazin-1-yl)-*N*-(4-((2-(2,6-dioxopiperidin-3-yl)-1,3-dioxoisoindolin-4-yl)amino)butyl)acetamide (**31**)

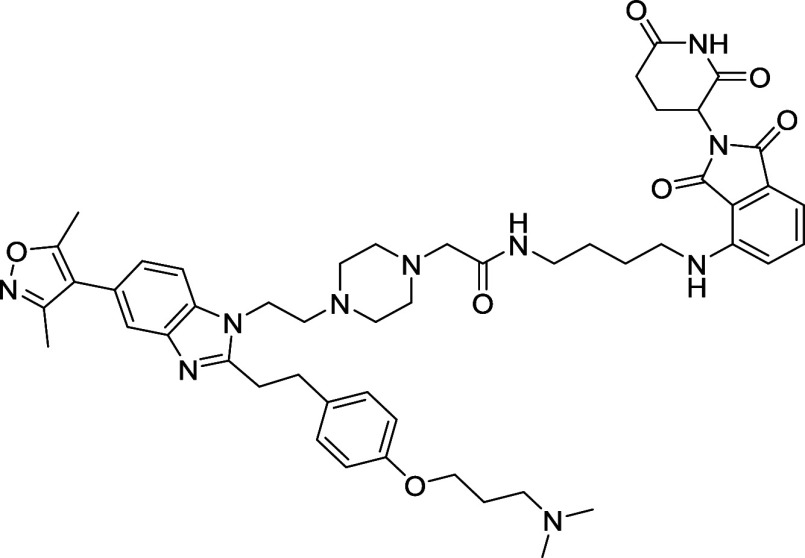



Degrader **31** was synthesized according to General Procedure B using pomalidomide 4′-alkylC4-amine. Purification by flash column chromatography and elution with 2–6% 7N (7N ammonia in methanol) in dichloromethane, followed by lyophilization, afforded the title compound as a yellow solid (65 mg, 31%). ^1^H NMR (600 MHz, *d*_6_-DMSO): δ 11.11 (br s, 1H), 7.73–7.68 (t, *J* = 6 Hz, 1H), 7.57–7.53 (m, 3H), 7.21 (d, *J* = 9 Hz, 2H), 7.16 (dd, *J* = 8, 2 Hz, 1H), 7.08 (d, *J* = 9 Hz, 1H), 7.00 (d, *J* = 7 Hz, 1H), 6.84 (d, *J* = 9 Hz, 2H), 6.57 (t, *J* = 6 Hz, 1H), 5.04 (dd, *J* = 13, 5 Hz, 1H), 4.24 (t, *J* = 6 Hz, 2H), 3.94 (t, *J* = 6 Hz, 2H), 3.30 (q, *J* = 7 Hz, 4H), 3.09–3.19 (m, 6H), 2.90–2.82 (m, 4H), 2.59–2.51 (m, 6H), 2.45–2.39 (m, 5H), 2.35–2.30 (m, *J* = 7 Hz, 4H), 2.23 (s, 3H), 2.13 (s, 6H), 1.97–2.02 (m, 1H), 1.83–1.78 (m, 2H), 1.45–1.52 (m, 3H). ^13^C NMR (151 MHz, *d*_6_-DMSO): δ 172.9, 170.1, 169.0, 167.3, 164.6, 158.4, 157.0, 155.6, 146.4, 142.6, 136.3, 134.5, 133.1, 132.2, 129.4, 122.7, 122.6, 118.9, 117.2, 116.7, 114.3, 110.4, 110.3, 109.0, 65.7, 61.3, 57.1, 55.7, 52.9, 52.8, 48.5, 45.2, 41.5, 40.1, 37.8, 31.8, 31.0, 28.6, 26.9, 26.7, 26.2, 22.2, 11.3, 10.6. LCMS (5–95% MeCN over 5 min) *t*_R_ = 3.212 min, Purity >99%, *m*/*z* (ES+): 915.5 [M+H^+^]^+^; HRMS-ESI (*m*/*z*): [M+H^+^]^+^ calculated for C_50_H_63_N_10_O_7_, 915.4881; found, 915.4950.

##### 2-(4-(2-(2-(4-(3-(Dimethylamino)propoxy)phenethyl)-5-(3,5-dimethylisoxazol-4-yl)-1*H*-benzo[*d*]imidazol-1-yl)ethyl)piperazin-1-yl)-*N*-(6-((2-(2,6-dioxopiperidin-3-yl)-1,3-dioxoisoindolin-4-yl)amino)hexyl)acetamide (**32**)

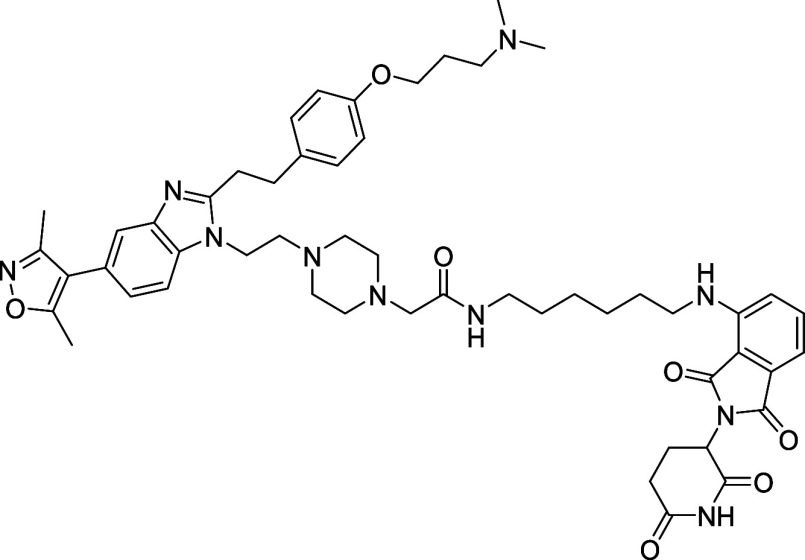



Degrader **32** was synthesized according to General Procedure B using pomalidomide 4′-alkylC6-amine. Purification by flash column chromatography and elution with 2–6% (7N ammonia in methanol) in dichloromethane, followed by lyophilization, afforded the title compound as a yellow solid (95 mg, 44%). ^1^H NMR (600 MHz, DMSO-*d*_6_): δ 11.11 (br s, 1H), 7.63 (t, *J* = 5.9 Hz, 1H), 7.57–7.53 (m, 3H), 7.20 (d, *J* = 8.6 Hz, 2H), 7.16 (dd, *J* = 8.2, 1.5 Hz, 1H), 7.07 (d, *J* = 8.6 Hz, 1H), 6.99 (d, *J* = 7.0 Hz, 1H), 6.83 (d, *J* = 8.6 Hz, 2H), 6.53 (t, *J* = 5.7 Hz, 1H), 5.04 (dd, *J* = 12.9, 5.7 Hz, 1H), 4.24 (t, *J* = 6.3 Hz, 2H), 3.93 (t, *J* = 6.4 Hz, 2H), 3.34–3.24 (m, 3H), 3.20–3.01 (m, 6H), 2.89–2.82 (m, 3H), 2.60–2.30 (m, 19H), 2.12 (s, 6H), 2.01–1.97 (m, 1H), 1.83–1.78 (m, 2H), 1.57–1.53 (m, 2H), 1.43–1.23 (m, 7H).^13^C NMR (151 MHz, DMSO-*d*_6_): δ 172.8, 170.1, 168.9, 168.8, 167.3, 164.5, 158.4, 157.0, 155.6, 146.4, 142.6, 136.3, 134.5, 133.0, 132.2, 129.4, 122.7, 122.6, 118.9, 117.2, 116.7, 114.3, 112.8, 110.4, 110.3, 110.1, 109.0, 65.7, 61.3, 57.1, 55.7, 52.9, 52.8, 48.5, 45.2, 41.8, 40.0, 38.1, 31.8, 30.9, 29.1, 28.6, 26.9, 26.1, 26.0, 22.1, 11.3, 10.5. LCMS (5–95% MeCN over 5 min) *t*_R_ = 3.681 min, Purity >97%, *m*/*z* (ES+): 943.6 [M+H^+^]^+^; HRMS-ESI (*m*/*z*): [M+H^+^]^+^ calculated for C_52_H_67_N_10_O_7_, 943.5194; found, 943.5203.

##### 2-(4-(2-(2-(4-(3-(Dimethylamino)propoxy)phenethyl)-5-(3,5-dimethylisoxazol-4-yl)-1*H*-benzo[*d*]imidazol-1-yl)ethyl)piperazin-1-yl)-*N*-(8-((2-(2,6-dioxopiperidin-3-yl)-1,3-dioxoisoindolin-4-yl)amino)octyl)acetamide (**33**)

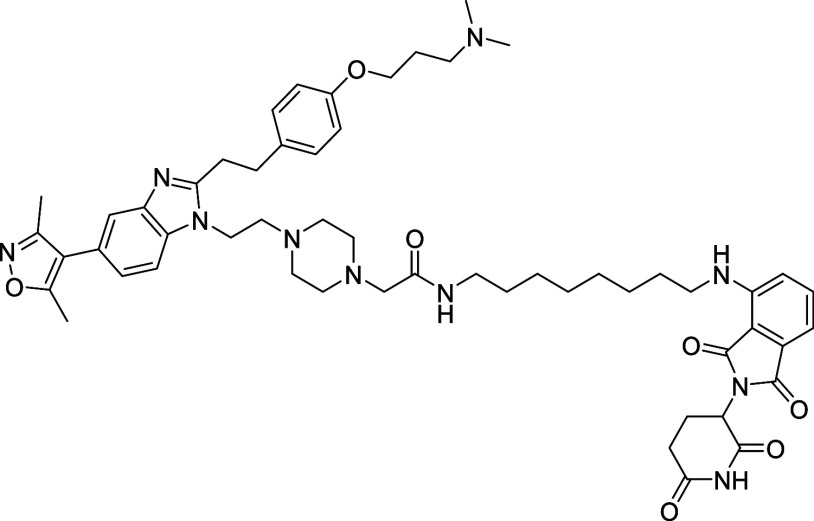



Degrader **33** was synthesized according to General Procedure B using pomalidomide 4′-alkylC8-amine. Purification by flash column chromatography and elution with 2–6% (7N ammonia in methanol) in dichloromethane, followed by lyophilization, afforded the title compound as a yellow solid (53 mg, 24%). ^1^H NMR (600 MHz, DMSO-*d*_6_): δ 11.11 (br s, 1H), 7.61 (t, *J* = 6 Hz, 1H), 7.53–7.56 (m, 3H), 7.20 (d, *J* = 9 Hz, 2H), 7.16 (dd, *J* = 8, 2 Hz, 1H), 7.07 (d, *J* = 9 Hz, 1H), 7.00 (d, *J* = 7 Hz, 1H), 6.83 (d, *J* = 9 Hz, 2H), 6.52 (t, *J* = 6 Hz, 1H), 5.04 (dd, *J* = 13, 6 Hz, 1H), 4.24 (t, *J* = 6 Hz, 2H), 3.93 (d, *J* = 6 Hz, 2H), 3.27 (d, *J* = 7 Hz, 2H), 3.14–3.17 (m, 2H), 3.09–3.12 (m, 2H), 3.05 (d, *J* = 7 Hz, 2H), 2.82–2.90 (m, 4H), 2.51–2.62 (m, 4H), 2.48–2.30 (m, 10H), 2.23 (s, 3H), 2.12 (s, 6H), 1.99–2.03 (m, 1H), 1.80 (d, *J* = 6 Hz, 2H), 1.55 (t, *J* = 7 Hz, 2H), 1.36–1.39 (m, 2H), 1.20–1.33 (m, 10H). ^13^C NMR (151 MHz, DMSO-*d*_6_): δ 172.9, 170.2, 168.8, 164.6, 158.4, 157.1, 155.6, 146.4, 142.6, 136.3, 134.5, 133.1, 132.2, 129.4, 122.7, 122.6, 118.9, 117.2, 116.7, 114.3, 110.4, 110.3, 65.7, 61.3, 57.1, 55.7, 53.0, 52.8, 48.5, 45.3, 41.8, 40.1, 38.1, 31.8, 31.0, 29.2, 28.7, 28.7, 27.0, 26.3, 26.3, 22.2, 11.3, 10.6. LCMS (5–95% MeCN over 5 min) *t*_R_ = 3.94 min, Purity = 97%, *m*/*z* (ES+): 971.5 [M+H^+^]^+^; HRMS-ESI (*m*/*z*): [M+H^+^]^+^ calculated for C_54_H_71_N_10_O_7_, 971.5507; found, 971.5601.

##### 2-(4-(2-(2-(4-(3-(Dimethylamino)propoxy)phenethyl)-5-(3,5-dimethylisoxazol-4-yl)-1*H*-benzo[*d*]imidazol-1-yl)ethyl)piperazin-1-yl)-*N*-(2-((2-(2,6-dioxopiperidin-3-yl)-1,3-dioxoisoindolin-4-yl)oxy)ethyl)acetamide (**34**)

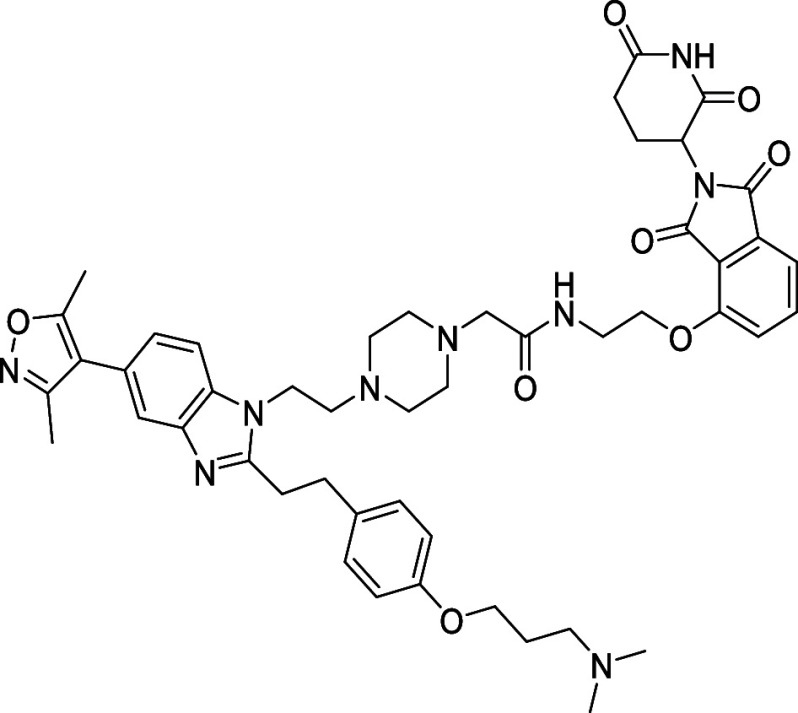



Degrader **34** was synthesized according to General Procedure B using thalidomide 4′-ether-alkylC2-amine. Purification by flash column chromatography and elution with 2–6% (7N ammonia in methanol) in dichloromethane, followed by lyophilization, afforded the title compound as a colorless solid (70 mg, 35%). ^1^H NMR (600 MHz, DMSO-*d*_6_): δ 11.11 (br s, 1H), 7.82 (t, *J* = 6 Hz, 1H), 7.78 (dd, *J* = 8, 7 Hz, 1H), 7.54–7.51 (m, 3H), 7.43 (d, *J* = 7 Hz, 1H), 7.17 (d, *J* = 9 Hz, 2H), 7.14 (dd, *J* = 8, 2 Hz, 1H), 6.81 (d, *J* = 9 Hz, 2H), 5.05 (dd, *J* = 13, 5 Hz, 1H), 4.27–4.18 (m, 4H), 3.91 (t, *J* = 6 Hz, 2H), 3.49 (q, *J* = 5 Hz, 2H), 3.15–3.05 (m, 4H), 2.77–2.87 (m, 4H), 2.52–2.45 (m, 4H), 2.42–2.27 (m, 12H), 2.21 (s, 3H), 2.11 (s, 6H), 1.92–1.97 (m, 1H), 1.84–1.78 (m, 2H). ^13^C NMR (151 MHz, DMSO-*d*_6_): δ 172.8, 169.9, 169.6, 166.8, 165.2, 164.6, 158.4, 157.0, 155.6, 155.6, 142.6, 137.1, 134.5, 133.3, 133.1, 129.4, 122.7, 122.6, 120.0, 118.9, 116.7, 116.4, 115.6, 114.3, 110.3, 67.3, 65.7, 61.1, 57.2, 55.7, 52.9, 52.8, 48.8, 45.2, 40.1, 37.5, 31.8, 30.9, 28.6, 26.9, 22.1, 11.4, 10.6. LCMS (5–95% MeCN over 5 min) *t*_R_ = 3.15 min, Purity >99%, *m*/*z* (ES+): 888.4 [M+H^+^]^+^; HRMS-ESI (*m*/*z*): [M+H^+^]^+^ calculated for C_48_H_58_N_9_O_8_, 888.4408; found, 888.4467.

##### 2-(4-(2-(2-(4-(3-(Dimethylamino)propoxy)phenethyl)-5-(3,5-dimethylisoxazol-4-yl)-1*H*-benzo[*d*]imidazol-1-yl)ethyl)piperazin-1-yl)-*N*-(4-((2-(2,6-dioxopiperidin-3-yl)-1,3-dioxoisoindolin-4-yl)oxy)butyl)acetamide (**35**)

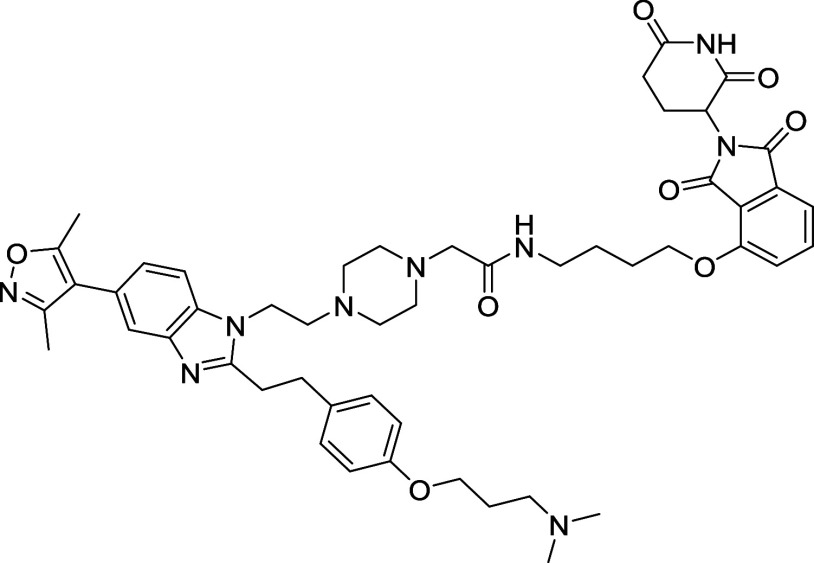



Degrader **35** was synthesized according to General Procedure B using thalidomide 4′-ether-alkylC4-amine. Purification by flash column chromatography and elution with 2–6% (7N ammonia in methanol) in dichloromethane, followed by lyophilization, afforded the title compound as a colorless solid (75 mg, 37%). ^1^H NMR (600 MHz, DMSO-*d*_6_): δ 11.10 (br s, 1H), 7.77 (dd, *J* = 9, 7 Hz, 1H), 7.71 (t, *J* = 6 Hz, 1H), 7.53 (d, *J* = 8 Hz, 2H), 7.47 (d, *J* = 9 Hz, 1H), 7.40 (d, *J* = 7 Hz, 1H), 7.18 (d, *J* = 9 Hz, 2H), 7.13 (dd, *J* = 8, 2 Hz, 1H), 6.81 (d, *J* = 9 Hz, 2H), 5.04 (dd, *J* = 13, 5 Hz, 1H), 4.23–4.17 (m, 4H), 3.91 (t, *J* = 6 Hz, 2H), 3.14 (dd, *J* = 8, 5 Hz, 4H), 3.09–3.06 (m, 2H), 2.87–2.81 (m, 3H), 2.56–2.48 (m, 5H), 2.44–2.27 (m, 12H), 2.20 (s, 3H), 2.11 (s, 6H), 2.00–1.95 (m, 1H), 1.84–1.79 (m, 2H), 1.76–1.70 (m, 2H), 1.62–1.56 (m, 2H). ^13^C NMR (151 MHz, DMSO-*d*_6_): δ 172.8, 170.0, 169.0, 166.9, 165.4, 164.6, 158.4, 157.0, 155.9, 155.6, 142.6, 137.1, 134.5, 133.3, 133.1, 129.4, 122.7, 122.6, 119.8, 118.9, 116.7, 116.2, 115.2, 114.3, 110.3, 68.5, 65.7, 61.3, 57.1, 55.7, 52.9, 52.9, 48.7, 45.2, 40.1, 37.7, 31.8, 31.0, 28.6, 26.9, 25.9, 25.8, 22.0, 11.3, 10.6. LCMS (5–95% MeCN over 5 min) *t*_R_ = 3.19 min, Purity >96%, *m*/*z* (ES+): 916.4 [M+H^+^]^+^; HRMS-ESI (*m*/*z*): [M+H^+^]^+^ calculated for C_50_H_62_N_9_O_8_, 916.4721; found, 916.4725.

##### 2-(4-(2-(2-(4-(3-(Dimethylamino)propoxy)phenethyl)-5-(3,5-dimethylisoxazol-4-yl)-1*H*-benzo[*d*]imidazol-1-yl)ethyl)piperazin-1-yl)-*N*-(6-((2-(2,6-dioxopiperidin-3-yl)-1,3-dioxoisoindolin-4-yl)oxy)hexyl)acetamide (**36**)

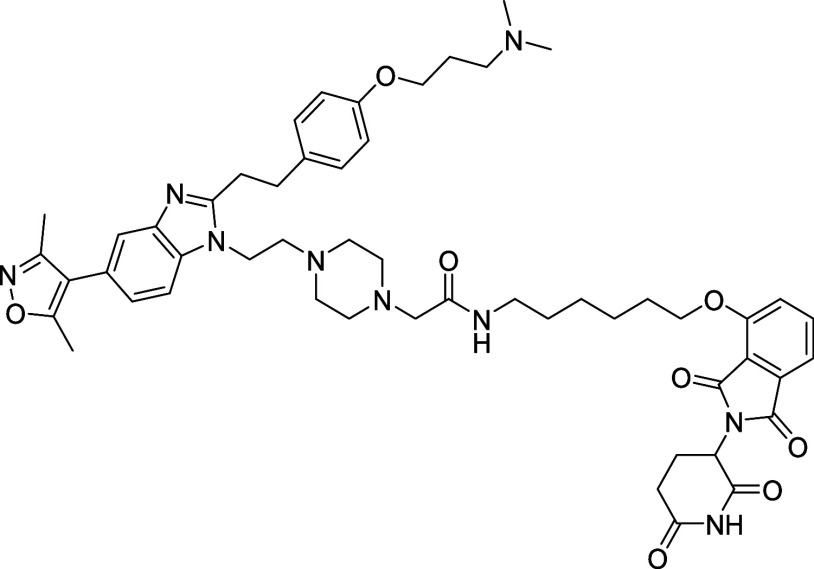



Degrader **36** was synthesized according to General Procedure B using thalidomide 4′-ether-alkylC6-amine. Purification by flash column chromatography and elution with 2–6% (7N ammonia in methanol) in dichloromethane, followed by lyophilization, afforded the title compound as a colorless solid (74 mg, 35%). ^1^H NMR (600 MHz, DMSO-*d*_6_): δ 11.11 (br s, 1H), 7.76 (dd, *J* = 8, 7 Hz, 1H), 7.60 (t, *J* = 6 Hz, 1H), 7.57–7.54 (m, 2H), 7.47 (d, *J* = 8 Hz, 1H), 7.40 (d, *J* = 7 Hz, 1H), 7.19 (d, *J* = 9 Hz, 2H), 7.14 (dd, *J* = 8, 2 Hz, 1H), 6.82 (d, *J* = 9 Hz, 2H), 5.06 (dd, *J* = 13, 5 Hz, 1H), 4.23 (t, *J* = 6 Hz, 2H), 4.16 (t, *J* = 6 Hz, 2H), 3.92 (t, *J* = 6 Hz, 2H), 3.16–3.12 (m, 2H), 3.11–3.07 (m, 2H), 3.04 (q, *J* = 7 Hz, 2H), 2.88–2.81 (m, 3H), 2.59–2.50 (m, 4H), 2.48–2.38 (m, 5H) 2.38–2.30 (m, 4H), 2.21 (s, 3H), 2.11 (s, 6H), 2.04–1.97 (m, 1H), 1.84–1.78 (m, 2H), 1.77–1.70 (m, 2H), 1.42–1.35 (m, 4H), 1.34–1.19 (m, 6H). ^13^C NMR (151 MHz, DMSO-*d*_6_): δ 172.8, 170.0, 168.8, 166.9, 165.3, 164.6, 158.4, 157.1, 156.0, 155.6, 142.6, 137.1, 134.5, 133.3, 133.1, 129.4, 122.7, 122.6, 119.8, 118.9, 116.7, 116.2, 115.2, 114.3, 110.3, 68.8, 65.7, 61.3, 57.1, 55.7, 53.0, 48.7, 45.2, 40.7, 40.1, 38.1, 31.8, 31.0, 29.2, 28.7, 28.4, 26.9, 26.3, 25.3, 22.0, 11.3, 10.6. LCMS (5–95% MeCN over 5 min) *t*_R_ = 3.42 min, Purity >99%, *m*/*z* (ES+): 944.5 [M+H^+^]^+^; HRMS-ESI (*m*/*z*) [M+H^+^]^+^ calculated for C_52_H_66_N_9_O_8_, 944.5034; found, 944.5126.

##### 2-(4-(2-(2-(4-(3-(Dimethylamino)propoxy)phenethyl)-5-(3,5-dimethylisoxazol-4-yl)-1*H*-benzo[*d*]imidazol-1-yl)ethyl)piperazin-1-yl)-*N*-(8-((2-(2,6-dioxopiperidin-3-yl)-1,3-dioxoisoindolin-4-yl)oxy)octyl)acetamide (**37**)

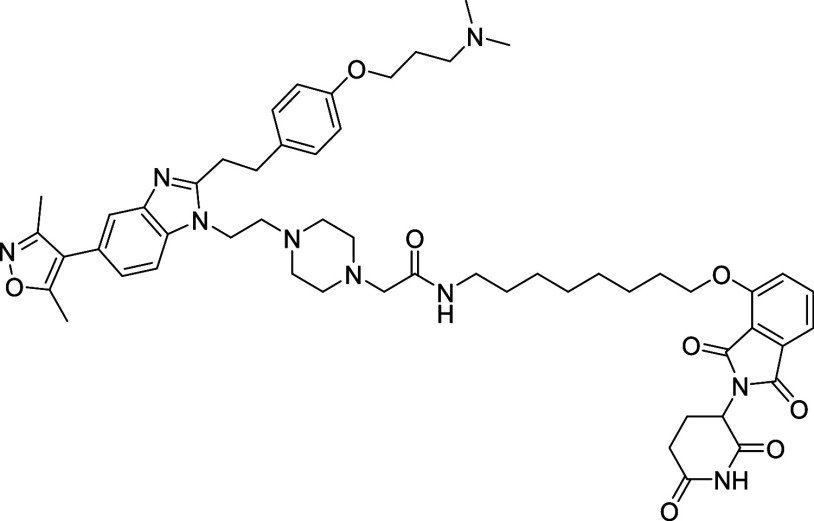



Degrader **37** was synthesized according to General Procedure B using thalidomide 4′-ether-alkylC8-amine. Purification by flash column chromatography and elution with 2–6% (7N ammonia in methanol) in dichloromethane, followed by lyophilization, afforded the title compound as a colorless solid (114 mg, 52%). ^1^H NMR (600 MHz, DMSO-*d*_6_): δ 11.12 (br s, 1H), 7.77 (t, 7 Hz, 1H), 7.64 (t, *J* = 6 Hz, 1H), 7.57–7.54 (m, 2H), 7.49 (d, *J* = 9 Hz, 1H), 7.42 (d, *J* = 7 Hz, 1H), 7.20 (d, *J* = 9 Hz, 2H), 7.16 (dd, *J* = 8, 2 Hz, 1H), 6.84 (d, *J* = 9 Hz, 2H), 5.07 (dd, *J* = 13, 5 Hz, 1H), 4.24 (t, *J* = 6 Hz, 2H), 4.18 (t, *J* = 6 Hz, 2H), 3.93 (t, *J* = 6 Hz, 2H), 3.19–3.14 (t, *J* = 7 Hz, 2H), 3.12–3.06 (m, 4H), 2.90–2.81 (m, 3H), 2.59–2.51 (m, 4H), 2.49–2.30 (m, 16H), 2.23 (s, 3H), 2.12 (s, 6H), 2.02–1.98 (m, 2H), 1.80–1.76 (m, 2H), 1.75–1.68 (m, 2H), 1.46–1.40 (m, 4H), 1.33–1.27 (m, 2H).^13^C NMR (151 MHz, DMSO-*d*_6_): δ 172.8, 170.0, 168.9, 166.9, 165.3, 164.6, 158.4, 157.1, 156.0, 155.6, 142.6, 137.1, 134.5, 133.3, 133.1, 129.4, 122.7, 122.6, 119.8, 118.9, 116.7, 116.2, 115.2, 114.3, 110.3, 109.6, 68.7, 65.7, 61.3, 57.1, 55.7, 52.9, 52.8, 48.7, 45.2, 40.7, 40.1, 38.1, 31.8, 31.0, 29.2, 28.6, 28.4, 27.0, 26.1, 25.0, 22.0, 11.3, 10.6. LCMS (5–95% MeCN over 5 min) *t*_R_ = 3.69 min, Purity >97%, *m*/*z* (ES+): 972.5 [M+H^+^]^+^; HRMS-ESI (*m*/*z*): [M+H^+^]^+^ calculated for C_54_H_70_N_9_O_8_, 972.5347; found, 972.5416.

##### 2-(4-(2-(2-(4-(3-(Dimethylamino)propoxy)phenethyl)-5-(3,5-dimethylisoxazol-4-yl)-1*H*-benzo[*d*]imidazol-1-yl)ethyl)piperazin-1-yl)-*N*-(2-(2-((2-(2,6-dioxopiperidin-3-yl)-1,3-dioxoisoindolin-4-yl)oxy)acetamido)ethyl)acetamide (**38**)

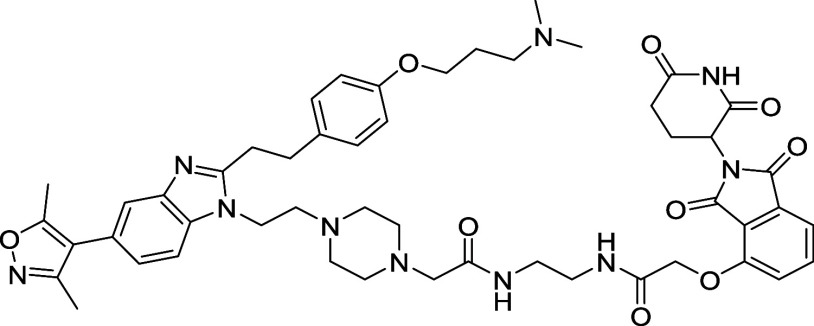



Degrader **38** was synthesized according to General Procedure B using thalidomide 4′-oxyacetamide-alkylC2-amine. Purification by flash column chromatography and elution with 2–6% (7N ammonia in methanol) in dichloromethane, followed by lyophilization, afforded the title compound as a colorless solid (34 mg, 16%). ^1^H NMR (600 MHz, DMSO-*d*_6_): δ 11.14 (br s, 1H), 8.05 (t, *J* = 6 Hz, 1H), 7.80–7.76 (m, 1H), 7.57–7.53 (m, 2H), 7.47 (d, *J* = 7 Hz, 1H), 7.37 (d, *J* = 8 Hz, 1H), 7.23–7.14 (m, 4H), 6.84 (d, *J* = 8 Hz, 2H), 5.11 (dd, *J* = 13, 5 Hz, 1H), 4.75 (s, 2H), 4.25–4.20 (m, 2H), 3.94 (t, *J* = 6 Hz, 2H), 3.24–3.08 (m, 10H), 2.91–2.78 (m, 4H), 2.62–2.53 (m, 2H), 2.45–2.22 (m, 12H), 2.22–2.18 (m, 8H), 2.03–1.90 (m, 2H), 1.85–1.79 (m, 2H). ^13^C NMR (151 MHz, DMSO-*d*_6_): δ 172.8, 169.9, 169.5, 167.2, 166.8, 165.5, 164.6, 158.4, 157.0, 155.6, 155.1, 142.6, 137.0, 134.5, 133.1, 133.1, 129.5, 129.4, 122.7, 122.6, 118.9, 116.8, 116.7, 116.1, 114.4, 114.3, 110.3, 67.6, 65.6, 65.4, 61.2, 57.1, 55.5, 52.9, 52.9, 48.8, 44.9, 40.1, 38.3, 38.1, 31.8, 31.0, 28.6, 22.0, 11.4, 10.6. LCMS (5–95% MeCN over 5 min) *t*_R_ = 3.17 min, Purity >99%, *m*/*z* (ES+): 945.4 [M+H^+^]^+^; HRMS-ESI (*m*/*z*): [M+H^+^]^+^ calculated for C_50_H_61_N_10_O_9_, 945.4623; found, 945.4614.

##### 2-(4-(2-(2-(4-(3-(Dimethylamino)propoxy)phenethyl)-5-(3,5-dimethylisoxazol-4-yl)-1*H*-benzo[*d*]imidazol-1-yl)ethyl)piperazin-1-yl)-*N*-(4-(2-((2-(2,6-dioxopiperidin-3-yl)-1,3-dioxoisoindolin-4-yl)oxy)acetamido)butyl)acetamide (**39**)

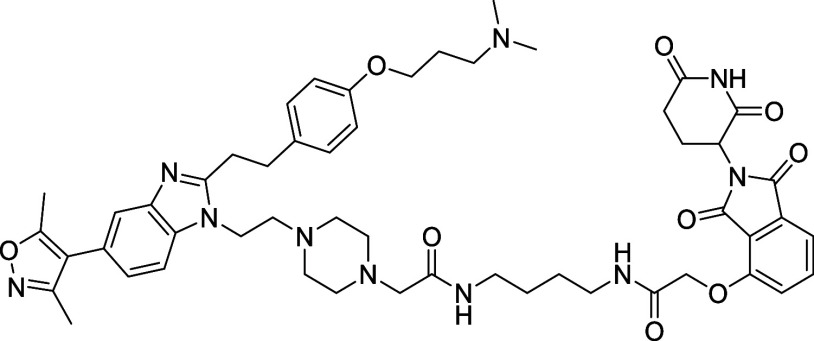



Degrader **39** was synthesized according to General Procedure B using thalidomide 4′-oxyacetamide-alkylC4-amine. Purification by flash column chromatography and elution with 2–6% (7N ammonia in methanol) in dichloromethane, followed by lyophilization, afforded the title compound as a colorless solid (72 mg, 32%). ^1^H NMR (600 MHz, DMSO-*d*_6_): δ 11.12 (br s, 1H), 7.96 (t, *J* = 6 Hz, 1H), 7.79–7.75 (m, 1H), 7.63 (t, *J* = 6 Hz, 1H), 7.53 (m, 2H), 7.45 (d, *J* = 7 Hz, 1H), 7.34 (d, *J* = 9 Hz, 1H), 7.19 (d, *J* = 8 Hz, 2H), 7.14 (d, *J* = 8 Hz, 1H), 6.82 (d, *J* = 9 Hz, 2H), 5.09 (dd, *J* = 13, 5 Hz, 1H), 4.74 (s, 2H), 4.66–4.51 (m, 2H), 4.25–4.19 (m, 2H), 3.92 (t, *J* = 6 Hz, 2H), 3.17–2.94 (m, 11H), 2.89–2.79 (m, 4H), 2.58–2.49 (m, 4H), 2.49–2.25 (m, 10H), 2.21–2.18 (m, 7H), 2.02–1.92 (m, 2H), 1.85–1.80 (m, 2H), 1.37 (d, *J* = 3 Hz, 4H). ^13^C NMR (151 MHz, DMSO-*d*_6_): δ 173.0, 172.8, 169.9, 169.0, 166.8, 166.7, 165.5, 164.6, 158.4, 157.0, 155.6, 155.1, 142.6, 137.0, 134.5, 133.1, 133.1, 129.4, 122.7, 122.6, 120.4, 118.9, 116.8, 116.7, 116.1, 114.3, 110.3, 67.6, 65.6, 61.3, 57.1, 55.5, 52.9, 52.9, 48.8, 44.8, 40.1, 38.1, 37.9, 31.8, 31.0, 28.6, 26.7, 26.5, 22.0, 11.4, 10.6. LCMS (5–95% MeCN over 5 min) *t*_R_ = 3.262 min, Purity >99%, *m*/*z* (ES+): 973.5 [M+H^+^]^+^; HRMS-ESI (*m*/*z*): [M+H^+^]^+^ calculated for C_52_H_65_N_10_O_9_, 973.4936; found, 973.5108.

##### 2-(4-(2-(2-(4-(3-(Dimethylamino)propoxy)phenethyl)-5-(3,5-dimethylisoxazol-4-yl)-1H-benzo[*d*]imidazol-1-yl)ethyl)piperazin-1-yl)-*i*-(6-(2-((2-(2,6-dioxopiperidin-3-yl)-1,3-dioxoisoindolin-4-yl)oxy)acetamido)hexyl)acetamide (**40**)

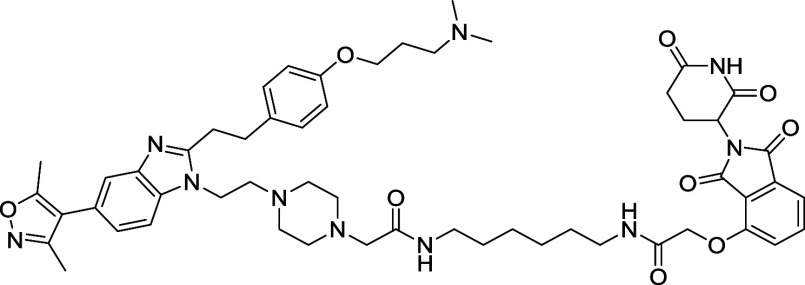



Degrader **40** was synthesized according to General Procedure B using thalidomide 4′-oxyacetamide-alkylC6-amine. Purification by flash column chromatography and elution with 2–6% (7N ammonia in methanol) in dichloromethane, followed by lyophilization, afforded the title compound as a colorless solid (97 mg, 43%). ^1^H NMR (600 MHz, DMSO-*d*_6_): δ 11.14 (br s, 1H), 7.94 (t, *J* = 6 Hz, 1H), 7.81–7.78 (m, 1H), 7.63 (t, *J* = 6 Hz, 1H), 7.55–7.52 (m, 2H), 7.48 (d, *J* = 7 Hz, 1H), 7.37 (d, *J* = 9 Hz, 1H), 7.21 (d, *J* = 9 Hz, 2H), 7.16 (dd, *J* = 8, 2 Hz, 1H), 6.84 (d, *J* = 9 Hz, 2H), 5.09 (dd, *J* = 13, 5 Hz, 1H), 4.76 (s, 2H), 4.65–4.45 (m, 1H), 4.25–4.20 (m, 2H), 3.94 (t, *J* = 6 Hz, 2H), 3.15–3.05 (m, 6H), 3.05–3.00 (m, 2H), 2.90–2.80 (m, 3H), 2.58–2.52 (m, 4H), 2.49–2.30 (m, 12H), 2.23 (s, 3H), 2.16 (s, 6H), 2.05–2.00 (m, 1H), 1.85–1.79 (m, 2H), 1.45–1.33 (m, 4H), 1.27–1.18 (m, 4H). ^13^C NMR (151 MHz, DMSO-*d*_6_): δ 172.8, 169.9, 168.9, 166.8, 166.6, 165.5, 164.6, 158.4, 157.0, 155.6, 155.1, 142.6, 137.0, 134.5, 133.1, 133.0, 129.4, 122.7, 122.6, 120.4, 118.9, 116.8, 116.7, 116.1, 114.3, 110.3, 67.6, 65.6, 61.3, 57.1, 55.6, 52.9, 52.8, 48.8, 45.0, 40.7, 40.1, 38.2, 38.1, 31.8, 31.0, 29.2, 29.0, 28.6, 26.8, 26.1, 26.0, 22.0, 11.3, 10.6. LCMS (5–95% MeCN over 5 min) *t*_R_ = 3.341 min, Purity >99.9%, *m*/*z* (ES+): 1001.2 [M+H^+^]^+^; HRMS-ESI (*m*/*z*): [M+H^+^]^+^ calculated for C_54_H_69_N_10_O_9_, 1001.5249; found, 1001.5238.

##### 2-(4-(2-(2-(4-(3-(Dimethylamino)propoxy)phenethyl)-5-(3,5-dimethylisoxazol-4-yl)-1H-benzo[*d*]imidazol-1-yl)ethyl)piperazin-1-yl)-*N*-(8-(2-((2-(2,6-dioxopiperidin-3-yl)-1,3-dioxoisoindolin-4-yl)oxy)acetamido)octyl)acetamide (**41**)

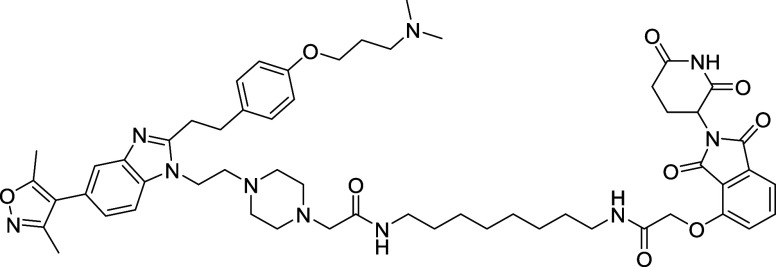



Degrader **41** was synthesized according to General Procedure B using thalidomide 4′-oxyacetamide-alkylC8-amine. Purification by flash column chromatography and elution with 2–6% (7N ammonia in methanol) in dichloromethane, followed by lyophilization, afforded the title compound as a colorless solid (48 mg, 20%). ^1^H NMR (600 MHz, DMSO-*d*_6_): δ 11.12 (br s, 1H), 7.92 (t, *J* = 6 Hz, 1H), 7.79–7.75 (m, 1H), 7.62–7.57 (t, *J* = 6 Hz, 1H), 7.54 (dd, *J* = 5, 3 Hz, 2H), 7.47 (d, *J* = 7 Hz, 1H), 7.36 (d, *J* = 9 Hz, 1H), 7.19 (d, *J* = 9 Hz, 2H), 7.14 (dd, *J* = 8, 2 Hz, 1H), 6.82 (d, *J* = 8 Hz, 2H), 5.10 (dd, *J* = 13, 5 Hz, 1H), 4.75 (s, 2H), 4.65–4.47 (m, 1H), 4.23 (t, *J* = 6 Hz, 2H), 3.92 (t, *J* = 6 Hz, 2H), 3.20–3.08 (m, 6H), 3.06–3.01 (m, 2H), 2.92–2.82 (m, 3H), 2.62–2.52 (m, 4H), 2.48–2.26 (m, 10H), 2.21 (s, 3H), 2.16 (s, 6H), 2.03–1.98 (m, 1H), 1.86–1.80 (m, 2H), 1.44–1.33 (m, 4H), 1.25–1.18 (m, 8H). ^13^C NMR (151 MHz, DMSO-*d*_6_): δ 172.8, 169.9, 168.8, 166.8, 166.6, 165.5, 164.6, 158.4, 157.0, 155.6, 155.1, 142.6, 136.9, 134.5, 133.1, 133.0, 129.4, 122.7, 122.6, 120.4, 118.9, 116.8, 116.7, 116.1, 114.3, 110.3, 67.6, 65.6, 61.3, 57.1, 55.6, 53.0, 52.8, 48.8, 45.0, 40.1, 38.3, 38.1, 31.8, 31.0, 29.2, 29.0, 28.7, 28.6, 26.7, 26.3, 22.0, 11.3, 10.6. LCMS (5–95% MeCN over 5 min) *t*_R_ = 3.478 min, Purity >95%, *m*/*z* (ES+): 1029.5 [M+H^+^]^+^.HRMS-ESI (*m*/*z*): [M+H^+^]^+^ calculated for C_56_H_73_N_10_O_9_, 1029.5562; found, 1029.5603.

##### (2*S*,4*R*)-1-((*S*)-2-(3-(2-(4-(2-(2-(4-(3-(Dimethylamino)propoxy)phenethyl)-5-(3,5-dimethylisoxazol-4-yl)-1*H*-benzo[*d*]imidazol-1-yl)ethyl)piperazin-1-yl)acetamido)propanamido)-3,3-dimethylbutanoyl)-4-hydroxy-*N*-(4-(4-methylthiazol-5-yl)benzyl)pyrrolidine-2-carboxamide (**42**)

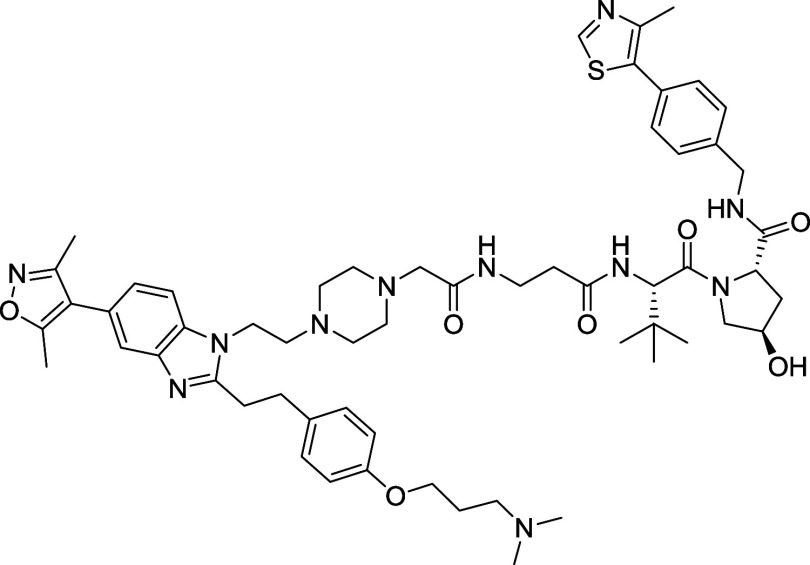



Degrader **42** was synthesized according to General Procedure B using VH 032 amide-alkylC2-amine. Purification by flash column chromatography and elution with 3–7% (7N ammonia in methanol) in dichloromethane, followed by lyophilization, afforded the title compound as a colorless solid (96 mg, 39%). ^1^H NMR (600 MHz, DMSO-*d*_6_): δ 8.96 (s, 1H), 8.57 (t, *J* = 6 Hz, 1H), 8.00 (d, *J* = 9 Hz, 1H), 7.62 (t, *J* = 6 Hz, 1H), 7.59–7.54 (m, 2H), 7.45–7.35 (m, 4H), 7.19 (d, *J* = 9 Hz, 2H), 7.14 (d, *J* = 8 Hz, 1H), 6.82 (d, *J* = 9 Hz, 2H), 5.18–5.14 (m, 1H), 4.52 (d, *J* = 9 Hz, 1H), 4.44–4.39 (m, 2H), 4.32 (br s, 1H), 4.28–4.17 (m, 3H), 3.92 (t, *J* = 6 Hz, 2H), 3.68–3.59 (m, 2H), 3.29–3.22 (m, 2H), 3.15 (t, *J* = 7 Hz, 2H), 3.11–3.07 (m, 2H), 2.85–2.77 (m, 2H), 2.55–2.51 (m, 3H), 2.49–2.40 (m, 10H), 2.40–2.30 (m, 7H), 2.21 (s, 3H), 2.16 (s, 6H), 2.05–2.00 (m, 1H), 1.90–1.85 (m, 1H), 1.82–1.79 (m, 2H), 0.90 (s, 9H). ^13^C NMR (151 MHz, DMSO-*d*_6_): δ 172.0, 170.5, 169.5, 168.9, 164.6, 158.4, 157.1, 155.6, 151.5, 147.7, 142.6, 139.5, 134.5, 133.1, 131.2, 129.7, 129.4, 128.7, 127.4, 122.7, 122.6, 118.9, 116.7, 114.3, 110.3, 68.9, 65.7, 61.2, 58.7, 57.1, 56.4, 55.7, 53.0, 52.9, 45.2, 41.7, 40.8, 40.1, 38.0, 35.3, 35.0, 34.7, 31.8, 28.6, 27.0, 26.4, 16.0, 11.4, 10.6. LCMS (5–95% MeCN over 5 min) *t*_R_ = 3.405 min, Purity >98%, *m*/*z* (ES+): 1072.6 [M+H^+^]^+^; HRMS-ESI (*m*/*z*): [M+H^+^]^+^ calculated for C_58_H_78_N_11_O_7_S, 1072.5806; found, 1072.5883.

##### (2*S*,4*R*)-1-((*S*)-2-(5-(2-(4-(2-(2-(4-(3-(Dimethylamino)propoxy)phenethyl)-5-(3,5-dimethylisoxazol-4-yl)-1*H*-benzo[*d*]imidazol-1-yl)ethyl)piperazin-1-yl)acetamido)pentanamido)-3,3-dimethylbutanoyl)-4-hydroxy-*N*-(4-(4-methylthiazol-5-yl)benzyl)pyrrolidine-2-carboxamide (**43**)

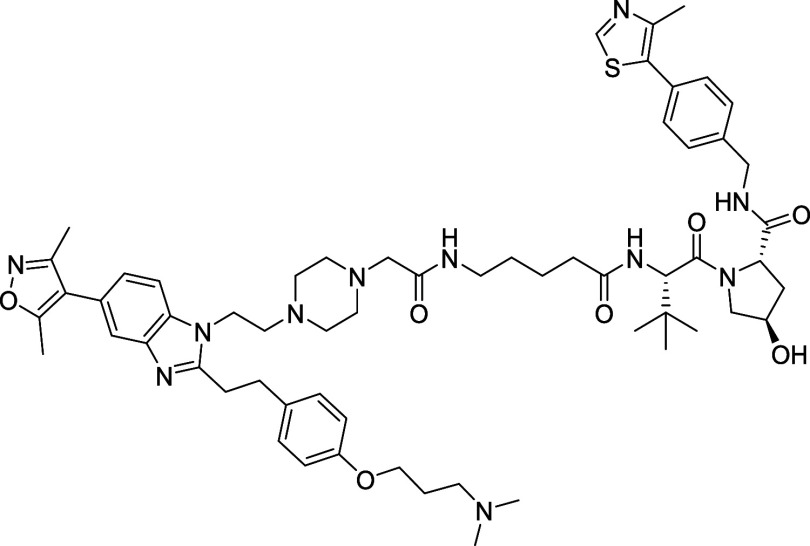



Degrader **43** was synthesized according to General Procedure B using VH 032 amide-alkylC4-amine. Purification by flash column chromatography and elution with 3–7% (7N ammonia in methanol) in dichloromethane, followed by lyophilization, afforded the title compound as a colorless solid (55 mg, 22%). ^1^H NMR (600 MHz, DMSO-*d*_6_): δ 8.96 (s, 1H), 8.57 (t, *J* = 6 Hz, 1H), 7.86 (d, *J* = 9 Hz, 1H), 7.64 (t, *J* = 6 Hz, 1H), 7.55–7.53 (m, 2H), 7.40 (d, *J* = 8 Hz, 2H), 7.36 (d, *J* = 8 Hz, 2H), 7.19 (d, *J* = 9 Hz, 2H), 7.14 (dd, *J* = 8, 2 Hz, 1H), 6.82 (d, *J* = 9 Hz, 2H), 5.17–5.13 (m, 1H), 4.52 (d, *J* = 9 Hz, 1H), 4.46–4.39 (m, 2H), 4.32 (br s, 1H), 4.23 (t, *J* = 6 Hz, 2H), 4.19 (dd, *J* = 16, 5 Hz, 1H), 3.92 (t, *J* = 6 Hz, 2H), 3.66–3.59 (m, 2H), 3.15 (t, *J* = 7 Hz, 2H), 3.10 (d, *J* = 8 Hz, 2H), 3.04 (q, *J* = 7 Hz, 2H), 2.82 (s, 2H), 2.53 (t, *J* = 6 Hz, 2H), 2.48–2.24 (m, 15H), 2.21 (s, 3H), 2.10 (s, 6H), 2.05–2.09 (m, 1H), 2.05–2.00 (m, 1H), 1.90–1.85 (m, 1H), 1.82–1.78 (m, 2H), 1.50–1.33 (m, 4H), 0.90 (s, 9H). 3H signal hidden behind residual water signal. ^13^C NMR (151 MHz, DMSO-*d*_6_): δ 172.0, 169.7, 168.9, 164.6, 158.4, 157.1, 155.6, 151.5, 147.7, 142.6, 139.5, 134.5, 133.1, 131.2, 129.6, 129.4, 128.7, 127.4, 122.7, 122.6, 118.9, 116.7, 114.3, 110.3, 68.9, 65.7, 61.3, 58.7, 57.1, 56.4, 56.3, 55.7, 53.0, 52.9, 45.3, 41.6, 40.1, 38.0, 37.9, 35.3, 35.0, 34.6, 31.8, 29.6, 28.6, 27.0, 26.4, 22.9, 16.0, 11.3, 10.6. LCMS (5–95% MeCN over 5 min) *t*_R_ = 3.562 min, Purity >96%, *m*/*z* (ES+): 1100.6 [M+H^+^]^+^; HRMS-ESI (*m*/*z*): [M+H^+^]^+^ calculated for C_60_H_82_N_11_O_7_S, 1100.6119; found, 1100.6189.

##### (2*S*,4*R*)-1-((*S*)-2-(7-(2-(4-(2-(2-(4-(3-(Dimethylamino)propoxy)phenethyl)-5-(3,5-dimethylisoxazol-4-yl)-1*H*-benzo[*d*]imidazol-1-yl)ethyl)piperazin-1-yl)acetamido)heptanamido)-3,3-dimethylbutanoyl)-4-hydroxy-*N*-(4-(4-methylthiazol-5-yl)benzyl)pyrrolidine-2-carboxamide (**44**)

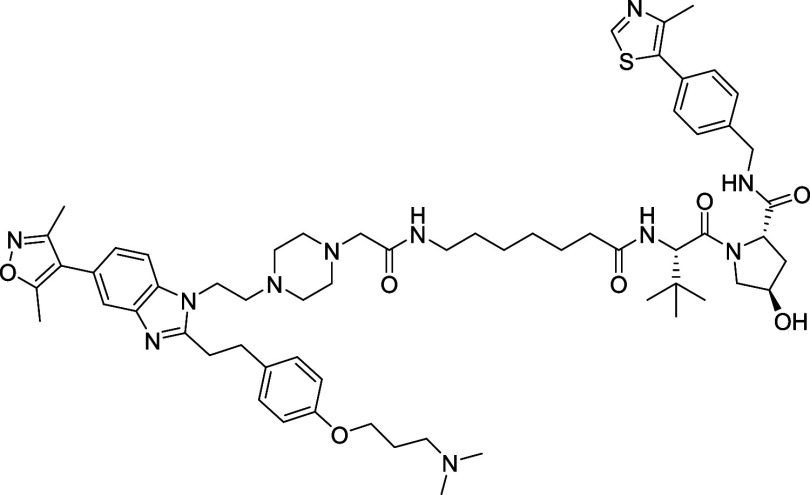



Degrader **44** was synthesized according to General Procedure B using VH 032 amide-alkylC6-amine. Purification by flash column chromatography and elution with 3–7% (7N ammonia in methanol) in dichloromethane, followed by lyophilization, afforded the title compound as a colorless solid (93 mg, 36%). ^1^H NMR (600 MHz, DMSO-*d*_6_): δ 8.96 (s, 1H), 8.57 (t, *J* = 6 Hz, 1H), 7.85 (d, *J* = 9 Hz, 1H), 7.61 (t, *J* = 6 Hz, 1H), 7.54 (dd, *J* = 5, 3 Hz, 2H), 7.40 (d, *J* = 8 Hz, 2H), 7.36 (d, *J* = 8 Hz, 2H), 7.19 (d, *J* = 9 Hz, 2H), 7.14 (dd, *J* = 8, 1.5 Hz, 1H), 6.82 (d, *J* = 9 Hz, 2H), 5.12 (s, 1H), 4.52 (d, *J* = 9 Hz, 1H), 4.45–4.39 (m, 2H), 4.32 (br s, 1H), 4.24 (t, *J* = 6 Hz, 2H), 4.19 (dd, *J* = 16, 5 Hz, 1H), 3.92 (t, *J* = 6 Hz, 2H), 3.59–3.66 (m, 2H), 3.13–3.16 (m, 2H), 3.09 (t, *J* = 7 Hz, 2H), 3.02 (q, *J* = 7 Hz, 2H), 2.82 (s, 2H), 2.52 (t, *J* = 6 Hz, 2H), 2.49–2.24 (m, 14H), 2.21 (s, 3H), 2.10 (s, 6H), 2.05–2.09 (m, 1H), 1.99 (d, *J* = 8 Hz, 1H), 1.85–1.89 (m, 1H), 1.79–1.75 (m, 2H), 1.49–1.39 (m, 2H), 1.35 (t, *J* = 7 Hz, 2H), 1.20 (d, *J* = 4 Hz, 4H), 0.90 (s, 9H). 3H signal hidden behind residual water signal. ^13^C NMR (151 MHz, DMSO-*d*_6_): δ 172.1, 172.0, 169.7, 168.9, 164.6, 158.4, 157.1, 155.6, 151.5, 147.7, 142.6, 139.5, 134.5, 133.1, 131.2, 129.6, 129.4, 128.7, 127.4, 122.7, 122.6, 118.9, 116.7, 114.3, 110.3, 68.9, 65.7, 61.3, 58.7, 57.1, 56.4, 56.3, 55.7, 53.0, 52.8, 45.3, 41.7, 40.1, 38.2, 38.0, 35.2, 34.8, 31.8, 29.1, 28.6, 28.4, 27.0, 26.4, 26.2, 25.4, 16.0, 11.3, 10.6. LCMS (5–95% MeCN over 5 min) *t*_R_ = 3.930 min, Purity = 97%, *m*/*z* (ES+): 1128.6 [M+H^+^]^+^; HRMS-ESI (*m*/*z*): [M+H^+^]^+^ calculated for C_62_H_86_N_11_O_7_S, 1128.6432; found, 1128.6401.

##### (2*S*,4*R*)-1-((*S*)-2-(9-(2-(4-(2-(2-(4-(3-(Dimethylamino)propoxy)phenethyl)-5-(3,5-dimethylisoxazol-4-yl)-1*H*-benzo[*d*]imidazol-1-yl)ethyl)piperazin-1-yl)acetamido)nonamido)-3,3-dimethylbutanoyl)-4-hydroxy-*N*-(4-(4-methylthiazol-5-yl)benzyl)pyrrolidine-2-carboxamide (**45**)

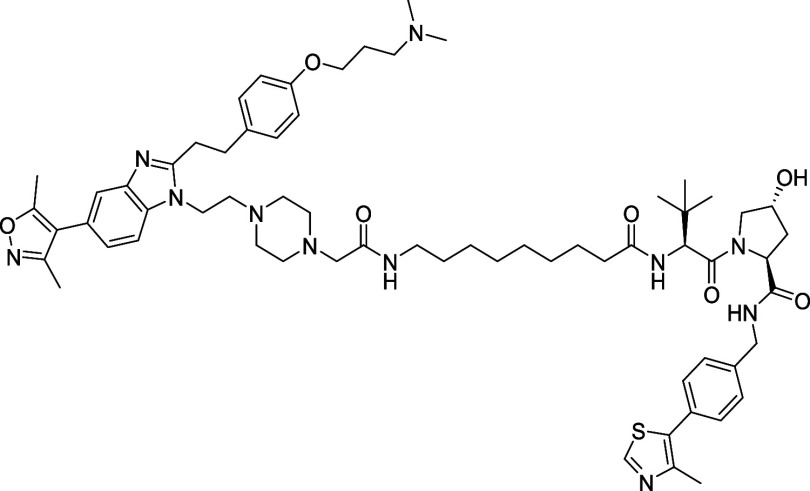



Degrader **45** was synthesized according to General Procedure B using VH 032 amide-alkylC8-amine. Purification by flash column chromatography and elution with 3–7% (7N ammonia in methanol) in dichloromethane, followed by lyophilization, afforded the title compound as a colorless solid (91 mg, 35%). ^1^H NMR (600 MHz, DMSO-*d*_6_): δ 8.98 (s, 1H), 8.58 (t, *J* = 6 Hz, 1H), 7.86 (d, *J* = 9 Hz, 1H), 7.62 (t, *J* = 6 Hz, 1H), 7.54–7.57 (m, 2H), 7.41 (d, *J* = 8 Hz, 2H), 7.38 (d, *J* = 8 Hz, 2H), 7.20 (d, *J* = 8 Hz, 2H), 7.16 (d, *J* = 8 Hz, 1H), 6.84 (d, *J* = 9 Hz, 2H), 5.14 (d, *J* = 3 Hz, 1H), 4.53 (d, *J* = 9 Hz, 1H), 4.39–4.45 (m, 2H), 4.34 (s, 1H), 4.27–4.18 (m, 3H), 3.94 (t, *J* = 6 Hz, 2H), 3.61–3.67 (m, 2H), 3.16 (t, *J* = 8 Hz, 2H), 3.11 (d, *J* = 9 Hz, 2H), 3.04 (q, *J* = 7 Hz, 2H), 2.83 (s, 2H), 2.54 (t, *J* = 6 Hz, 3H), 2.45 (s, 2H), 2.44 (s, 3H), 2.40 (s, 3H), 2.32 (t, *J* = 7 Hz, 6H), 2.23 (s, 4H), 2.12 (s, 6H), 2.00–2.04 (m, 1H), 1.93–1.86 (m, 1H), 1.83–1.78 (m, 2H), 1.52–1.35 (m, 5H), 1.22 (s, 9H), 0.92 (s, 9H). ^13^C NMR (151 MHz, DMSO-*d*_6_): δ 172.1, 172.0, 169.7, 168.8, 164.6, 158.4, 157.1, 155.6, 151.5, 147.7, 142.6, 139.5, 134.5, 133.1, 131.2, 129.6, 129.4, 128.7, 127.4, 122.7, 122.6, 118.9, 116.7, 114.3, 110.3, 68.9, 65.7, 61.3, 58.7, 57.1, 56.4, 56.3, 55.7, 53.0, 52.8, 45.3, 41.6, 40.1, 38.2, 38.0, 35.2, 34.9, 31.8, 29.2, 28.7, 28.7, 27.0, 26.4, 25.5, 16.0, 11.3, 10.6.LCMS (5–95% MeCN over 5 min) *t*_R_ = 4.271 min, Purity = 87%, *m*/*z* (ES+): 1156.6 [M+H^+^]^+^; HRMS-ESI (*m*/*z*): [M+H^+^]^+^ calculated for C_64_H_90_N_11_O_7_S, 1156.6745; found, 1156.6660.

AlphaScreen assays were performed at the University of Oxford, with minor modifications from the manufacturer’s protocol (PerkinElmer, USA). Briefly, all reagents were diluted in the recommended buffer (50 mM HEPES, 100 mM NaCl, 0.1% BSA; pH = 7.4) supplemented with 0.05% CHAPS and allowed to equilibrate to ambient temperature prior to addition to plates. Concentrations of the various proteins, peptides, solvents, and compounds are given in the relevant results sections and are expressed as the final concentrations after the addition of all assay components. Four μL of HIS-tagged protein was added to low-volume 384-well plates (ProxiPlatet-384 Plus, PerkinElmer, USA), followed by 4 μL of either buffer, nonbiotinylated peptide, solvent, or compound. Plates were sealed and incubated at ambient temperature for 30 min, before the addition of 4 μL of biotinylated peptide, resealing, and incubation for a further 30 min. Four μL of streptavidin-coated donor beads (25 mg mL^–1^) and 4 μL of nickel chelate acceptor beads (25 μg/mL) were then added under low light conditions. Plates were foil sealed to protect from light, incubated at ambient temperature for 60 min, and read on a PHERAstar FS plate reader (BMG Labtech, Germany) using an AlphaScreen 680 excitation/570 emission filter set. IC_50_s were calculated in GraphPad Prism 5 (GraphPad Software, USA). Results for compounds dissolved in DMSO were normalized against corresponding DMSO controls prior to IC_50_ determination, which are given as the final concentration of the compound in the 20 μL reaction volume. **Cell lines**. HAP1 cells [male] were obtained from Horizon Discovery and grown in IMDM media supplemented with 10% fetal bovine serum (FBS). All cells were cultured at 37 °C in a 5% CO_2_ atmosphere.

### Protein Expression and Purification

The N-terminal bromodomain of BRD4 used for structural studies was expressed and purified as previously described.^49^ Briefly, human BRD4 BD1 (residues N44-E168) was subcloned into a pNIC28-Bsa4 vector (N-terminal His_6_-tag, followed by a TEV protease cleavage site). The expression plasmid was transformed into *Escherichia coli* BL21(D3)-R3-pRARE2 Rosetta cells. Cells were cultured in Terrific Broth (TB) media at 37 °C to an optical density (OD) of 2.8–3.0, and then expression was induced with 0.5 mM IPTG at 18 °C overnight. Cells were harvested and resuspended in a buffer containing 50 mM HEPES, pH 7.5, 500 mM NaCl, 0.5 mM TCEP, 5% glycerol and subsequently lysed by sonication. The first purification step of the recombinant protein was by Ni^2+^-affinity chromatography. The hexahistidine tag was then removed by TEV protease cleavage overnight, and the cleaved protein was separated by reverse Ni^2+^-affinity purification. The protein was further purified by size exclusion chromatography and elution with a HiLoad 16/600 Superdex 75 column with SEC buffer containing 25 mM HEPES, 150 mM NaCl, 0.5 mM TCEP, and 5% glycerol. Quality control was performed by SDS-polyacrylamide gel electrophoresis and ESI-MS.

### Crystallization and Structure Determination

Crystals of BRD4 BD1 in complex with degraders were grown using the sitting-drop vapor-diffusion technique at 277 K utilizing a mosquito crystallization robot (TTP Labtech, Royston, U.K.). BRD4 BD1 protein (10 mg/mL in SEC buffer) was incubated with inhibitors at a final concentration of 1–2 mM prior to setting up crystallization trials. Detailed crystallization conditions for each inhibitor/degrader are listed in Supporting Information Table S1. Crystals were cryo-protected with mother liquor supplemented with 23% ethylene glycol and flash-frozen in liquid nitrogen. X-ray diffraction data sets were collected at 100 K at beamline X06DA of the Swiss Light Source, Villigen, Switzerland, and at beamline I03 of the Diamond Light Source, Oxford, United Kingdom. The obtained diffraction data were integrated with either XDS^50^ or autoPROC^51^ and scaled with AIMLESS,^52^ which is part of the CCP4 package.^53^ Depending on the space group, the structures were then solved by molecular replacement using PHASER^54^ or by difference Fourier analysis using PHENIX^55^ with PDB entry 8P9H(56) as a starting model. Structure refinement was performed using iterative cycles of manual model building with COOT^57^ and refinement with PHENIX. Dictionary files for the compounds were generated using the Grade Web Server (http://grade.globalphasing.org). X-ray data collection and refinement statistics are listed in Supporting Information Table S2.

### *In Vitro* Ubiquitination

50 μL *in vitro* reactions included the following components and concentrations: UBE1 (E-304–050, R&D Systems), 100 nM; UBE2D1 (E2–616, R&D Systems), 1 μM; CRBN/DDB1/CUL4A(neddylated)/RBX1 complex (E3–441 and E3–500, premixed), 50 nM; FLAG-BRD4 (residues 49–460, SP-600, R&D Systems), 2 μM; ubiquitin (U-100H, R&D Systems), 50 μM; ATP (B-20, R&D Systems), 10 mM; degrader compound, variable concentrations. The buffer used was HEPES, 50 mM, pH 7.5; NaCl, 50 mM; TCEP, 1 mM. A premix containing all components other than degrader and ATP was added to individual wells in a 96-well plate. Degrader was then added at concentrations from 40 nM–40 μM (a no-compound control was also included). The plate was left for 10 min at room temperature and then brought to 37 °C for 5 min before initiating reactions with ATP addition. (dH_2_O was substituted for ATP in a negative control). Reactions were carried out for 60 min then terminated with reducing SDS sample buffer. The same assay was performed for VHL-recruiting degraders, replacing the CRBN/DDB1/CUL4A(neddylated)/RBX1 complex with VHL/Elongin B/Elongin C/CUL2/RBX1 complex (E3–600 and E3–420, R&D Systems, premixed), 100 nM. Analysis was on a WES (ProteinSimple) with 12–230 kDa, 25 capillary module (SM-W004, ProteinSimple): diluted samples 1:60 and used 3 μL per analysis (<4 ng FLAG-BRD4 per capillary). Anti-FLAG primary (MAB8529, R&D Systems) and Anti-Rabbit Detection Module (DM-001, ProteinSimple).

## Supporting Information Available

The ESI contains ^1^H, ^13^C NMR, HPLC purity, and mass spectral data of final compounds (PDF)
Molecular Formula Strings (CSV)
The atomic coordinates and structure factors of the first bromodomain of human BRD4 in complex with different degraders have been deposited in the Protein Data Bank (PDB) under accession codes 9F1J (**14**), 9F1K (**29**), 9F1L (**34**), 9F1M (**44**), and 9F1N (**45**) (PDB)

## Supplementary Material

jm5c00395_si_002.csv

jm5c00395_si_003.pdb

## Abbreviations Used

BRD4 BD1: Bromodomain-containing protein 4 first bromodomain
CBP/EP300: CREB-binding protein/E1A-binding protein P300
CRBN: Cereblon
FRET: Fluorescence resonance energy transfer
IRF4: Interferon regulatory factor 4
c-MYC: Myelocytomatosis oncogene cellular homologue
VHL: Von Hippel–Lindau
