# Supplementary material for: Structure-Guided Design of ISOX-DUAL-Based Degraders Targeting BRD4 and CBP/EP300: A Case of Degrader Collapse
Source: J Med Chem. 2025 Apr 17;68(9):9638–60. doi: 10.1021/acs.jmedchem.5c00395 (PMC12067448; doi:10.1021/acs.jmedchem.5c00395)
Supplement: Supplementary file 1 — jm5c00395_si_001.pdf [file jm5c00395_si_001.pdf]

Structure-guided design of ISOX-DUAL-based degraders targeting BRD4 and CBP/EP300. A case of degrader collapse.

Anthony K. Edmonds[a,b], Dimitrios-Ilias Balourdas[c,d], Graham P. Marsh[b], Robert Felix[b], Bradley Brasher[e], Jeff Cooper[e], Cari Graber-Feesl[e], Madhu Kollareddy[f], Karim Malik[f], Helen Stewart[g], Timothy J. T. Chevassut[g], Ella Lineham[h], Simon Morley[h], Oleg Fedorov[i], James Bennett[i], Mohan B. Rajasekaran[j], Samuel Ojeda[k], Drew A. Harrison[l], Christopher J. Ott[k,l], Andreas C. Joerger[c,d*], Hannah J. Maple[b*], John Spencer[a,j*]

[a]Chemistry Department, School of Life Sciences, University of Sussex, Brighton, BN1 9QJ, U.K.

[b]Bio-Techne (Tocris), The Watkins Building, Atlantic Road, Avonmouth, Bristol BS11 9QD, UK.

[c]Institute of Pharmaceutical Chemistry, Goethe University, Max-von-Laue-Str. 9, 60438 Frankfurt am Main, Germany.

[d]Structural Genomics Consortium (SGC), Buchmann Institute for Life Sciences, Max-von-Laue-Str. 15, 60438 Frankfurt am Main, Germany.

[e]Bio-Techne (R&D Systems), 614 McKinley Place NE, Minneapolis, 55413, USA.

[f]Cancer Epigenetics Laboratory, School of Cellular and Molecular Medicine, University of Bristol, Bristol, BS8 1TD

[g]Brighton and Sussex Medical School, University of Sussex, Brighton, BN1 9PS, U.K.

[h]Biochemistry Department, School of Life Sciences, University of Sussex, Brighton, BN1 9QQ, U.K.

[i] Centre for Medicines Discovery, Nuffield Department of Medicine, NDM Research building, Old Road Campus, Oxford OX3 7FZ, UK.

[j]Sussex Drug Discovery Centre, School of Life Sciences, University of Sussex, Brighton, BN1 9QJ, U.K.

[k]Krantz Family Center for Cancer Research, Massachusetts General Hospital, Charlestown, MA, 02129, USA.

[l] Department of Medicine, Harvard Medical School, Boston, MA, 02115, USA.

Email:Joerger@pharmchem.uni-frankfurt.de;Hannah.Maple@bio-techne.com; j.spencer@sussex.ac.uk.

Table of contents

Supporting Table S1. Crystallization conditions S2

Supporting Table S2. X-ray data collection and refinement statistics.......................................S3

Supporting Figure S1. Superimposition of the complexes of BRD4 BD1 with JQ1.......................S4

Figure S2. Stacking interactions between thalidomide moieties..S5

Scanned spectra for synthesized compounds..S6

Supporting Table S1. Crystallization conditions for BRD4 BD1-inhibitor complexes

**Compound**	**Temperature**	**Reservoir buffer**	**Drop volume ratio** (protein:reservoir solution)
**14**	4 °C	25% PEG 3350, 0.25 M sodium nitrate, 15% ethylene glycol, 0.1 M bis-tris propane pH 8.2	2:1
**29**	4 °C	25% PEG 3350, 0.3 M sodium nitrate, 15% ethylene glycol, 0.1 M bis-tris propane pH 8.5	2:1
**34**	4 °C	25% PEG 3350, 0.2 M sodium nitrate, 15% ethylene glycol, 0.1 M bis-tris propane pH 7.9	1:1
**44**	4 °C	24% PEG 3350, 0.3 M sodium nitrate, 15% ethylene glycol, 0.1 M bis-tris propane pH 8.5	2:1
**45**	4 °C	25% PEG 3350, 0.3 M sodium malonate pH 7, 10% ethylene glycol, 0.1 M bis-tris propane pH 8.5	1:1

Supporting Table S2. X-ray data collection and refinement statistics

BRD4 BD1 complex	**14**	**29**	**34**	**44**	**45**
Data Collection					
Space Group	$P2_12_12_1$	$P2_12_12_1$	$C2$	$P2_1$	$P2_1$
a, b, c (Å)	38.9, 50.5, 58.5	39.0, 50.7, 59.7	56.8, 43.9, 56.3	72.4, 48.7, 73.0	71.9, 48.9, 73.0
α, β, γ (°)	90.0, 90.0, 90.0	90.0, 90.0, 90.0	90.0, 102.2, 90.0	90.0, 109.4, 90.0	90.0, 109.1, 90.0
Molecules/asymmetric unit	1	1	1	4	4
Resolution (Å)[a]	38.88-1.13 (1.15-1.13)	39.00-1.61 (1.64-1.61)	34.42-1.30 (1.32-1.30)	68.84-1.90 (1.94-1.90)	69.01-1.71 (1.74-1.71)
Unique reflections[a]	43,620 (2,020)	15,948 (813)	33,423 (1,660)	37,837 (2,429)	51,876 (2,748)
Completeness (%)[a]	99.4 (94.5)	100 (99.9)	100 (99.9)	99.3 (99.7)	99.5 (99.5)
Multiplicity[a]	9.4 (9.2)	6.4 (6.5)	6.6 (6.4)	7.1 (6.7)	7.0 (6.9)
R_{merge}[a]	0.059 (0.694)	0.041 (0.786)	0.034 (0.822)	0.088 (0.931)	0.074 (0.941)
R_{pim}[a]	0.020 (0.238)	0.018 (0.333)	0.014 (0.351)	0.036 (0.387)	0.030 (0.385)
CC(1/2)[a]	0.999 (0.901)	1.000 (0.841)	1.000 (0.893)	0.999 (0.673)	0.999 (0.710)
Mean $I/\sigma(I)$[a]	16.9 (3.2)	20.9 (2.3)	23.3 (2.1)	12.0 (2.0)	13.2 (1.9)
Refinement					
R_{work}, (%)[b]	15.8	17.5	15.7	19.6	19.4
R_{free}, (%)[b]	18.5	21.3	18.2	23.7	23.2
No. of atoms					
Protein[c]	1,077	1,040	1,066	4,046	4,095
Water	107	73	136	264	360
Ligands	42	47	65	148	203
Ethylene glycol	8	4	16	20	16
Ions	0	0	0	3	4
RMSD bonds (Å)	0.006	0.007	0.005	0.008	0.007
RMSD angles (°)	0.86	0.86	0.77	0.92	0.88
Mean B (Å^2)	17.2	31.6	25.6	28.4	27.6
Ramachandran favored (%)[d]	98.4	98.4	98.4	98.6	98.8
Ramachandran outliers (%)[d]	0.0	0.0	0.0	0.0	0.0
PDB entry	9F1J	9F1K	9F1L	9F1M	9F1N

[a]Values in parentheses are for the highest-resolution shell.
[b]R_{work} and $R_{free} = \sum||F_{obs}| - |F_{calc}||/\sum|F_{obs}|$, where R_{free} was calculated with 5% of the reflections chosen at random and not used in the refinement.
[c]Number includes alternative conformations.
[d]MolProbity statistics

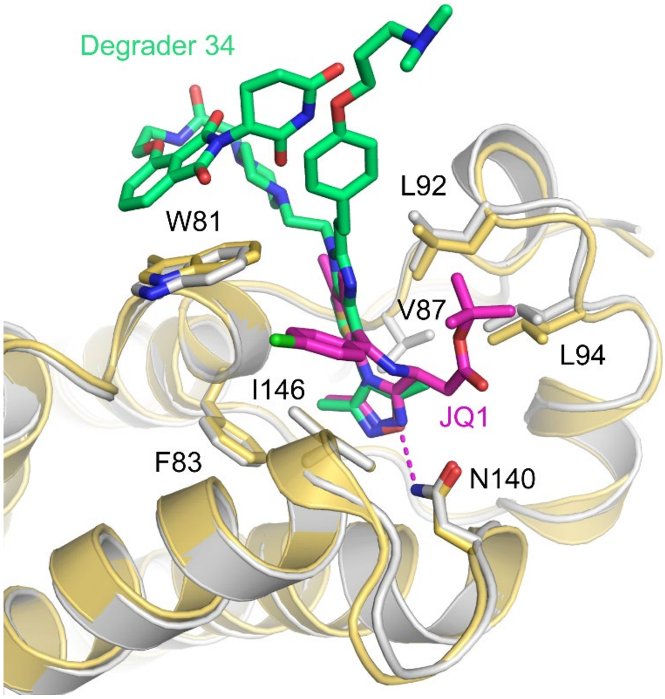

Figure S1. Superimposition of the complexes of BRD4 BD1 with JQ1 (PDB entry 3MXF) and degrader **34**, highlighting that both molecules share an interaction with Trp81, albeit packing from different sides. Compound **34** is shown in green with the corresponding protein chain in gray, and JQ1 in magenta with the corresponding protein chain in light yellow. The side chains of selected interacting residues are highlighted as stick models.

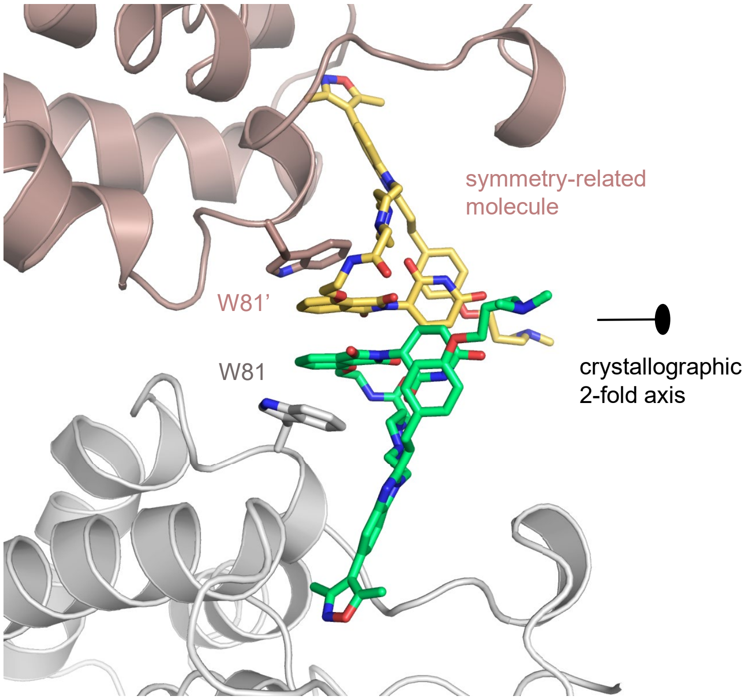

Figure S2. Stacking interactions between thalidomide moieties from two symmetry-related molecules in the crystal structure of BRD4 BD1 with degrader **34**.

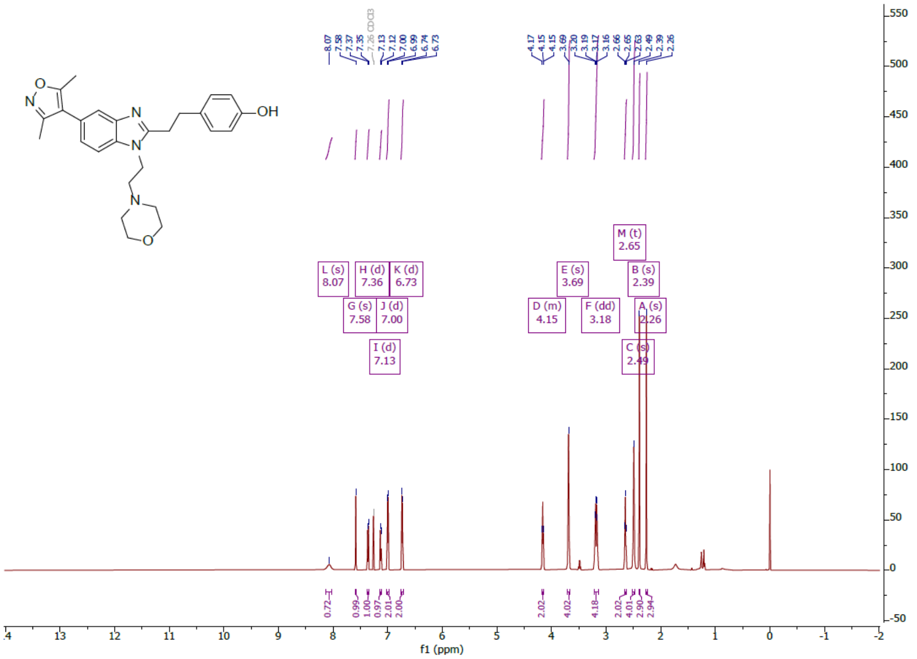

^{1}H NMR spectrum for **6**.

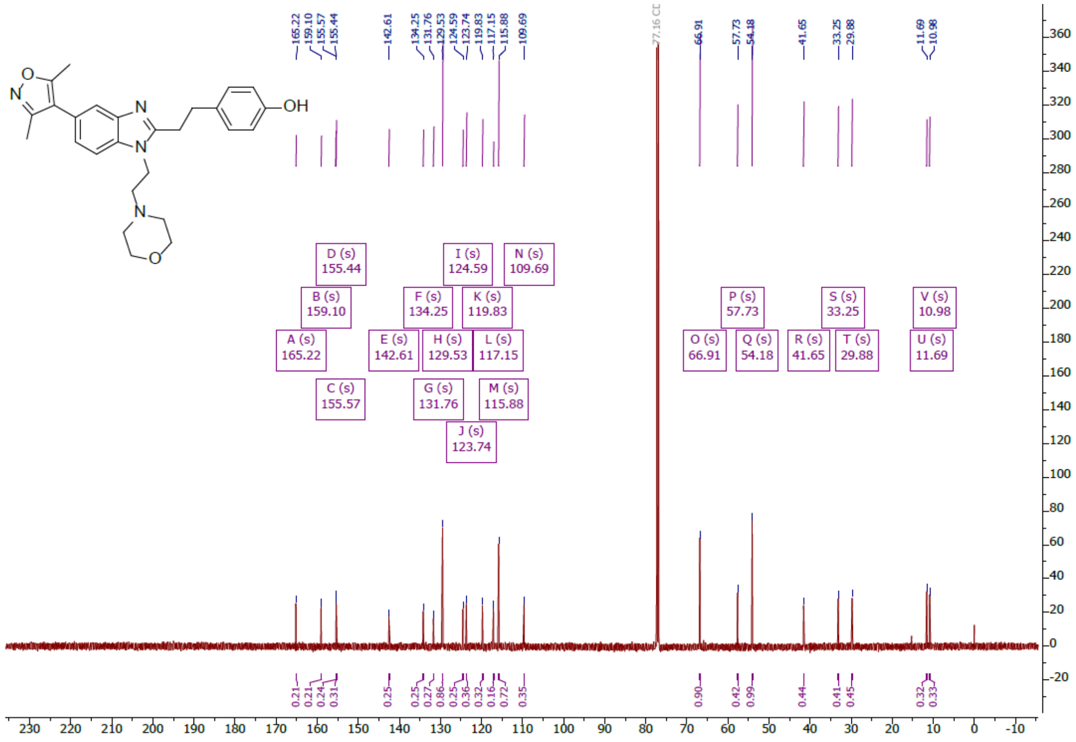

^{13}C NMR spectrum for **6**.

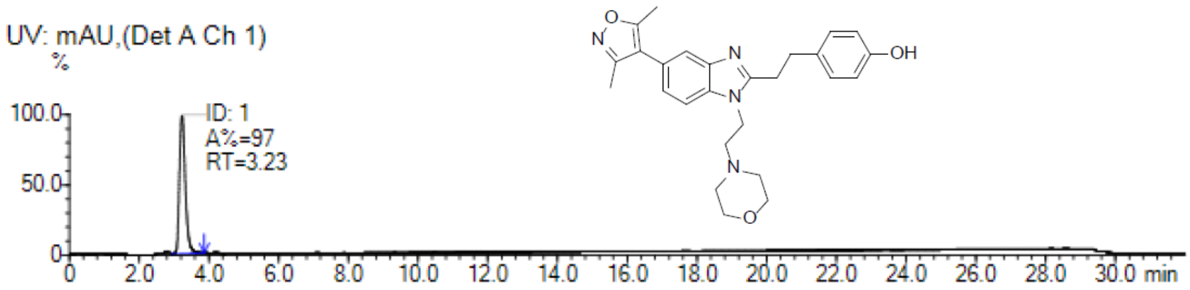

Peak ID	RT (min)	Channel	Area (%)
1	3.23	1	97.0

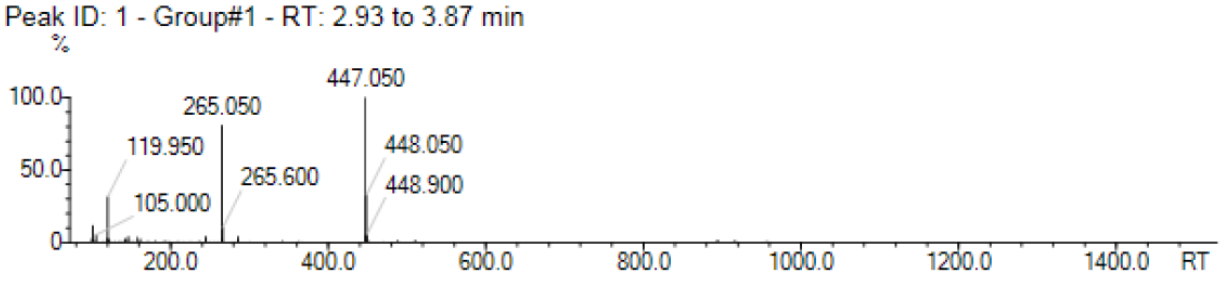

LC MS for **6**.

Scanned spectra for compounds

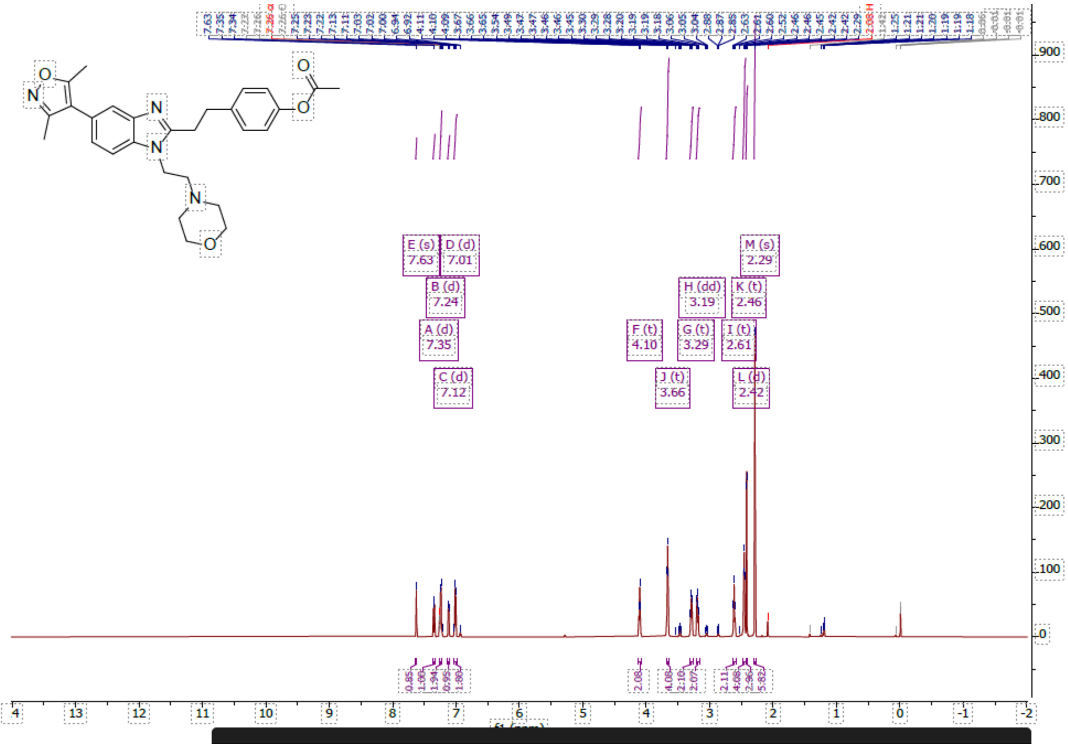

^{1}H NMR spectrum (**7**)

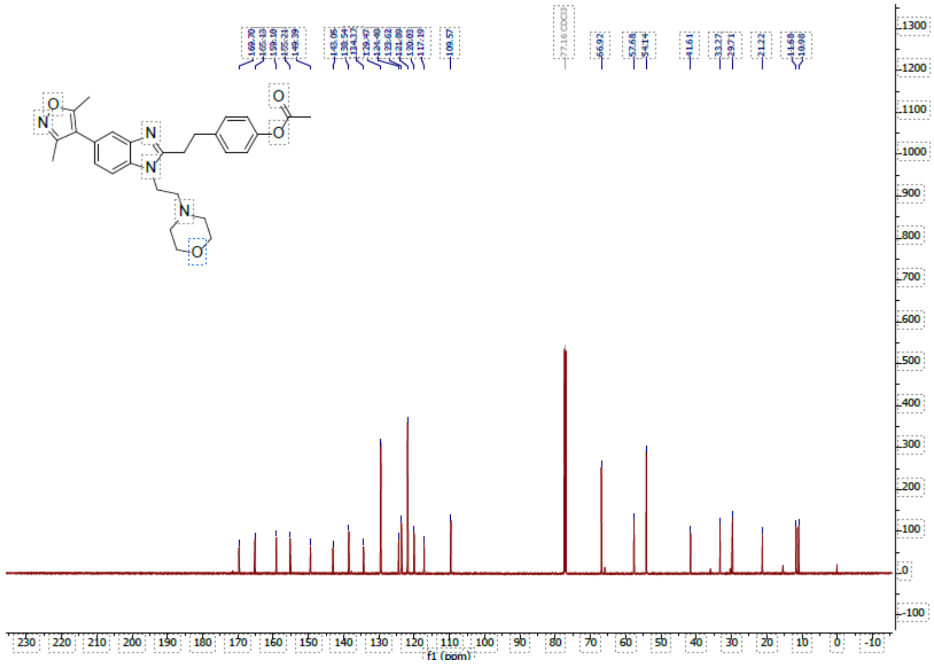

^{13}C NMR spectrum (**7**)

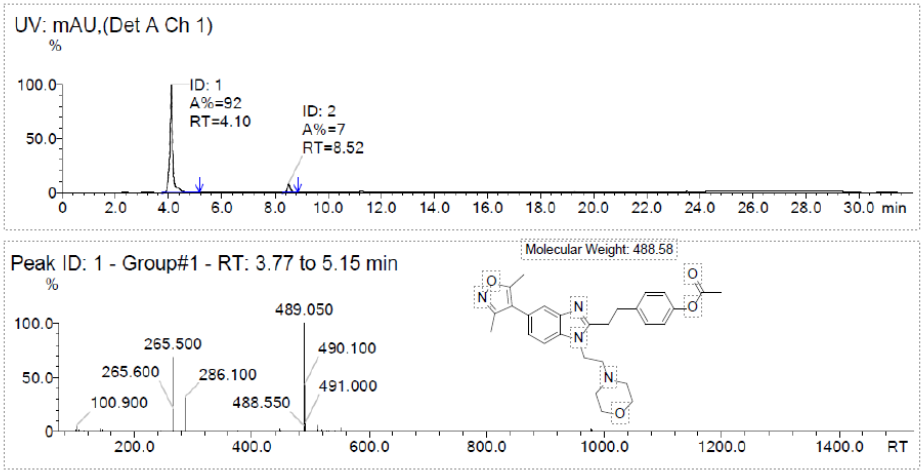

LC-MS for compound **7**

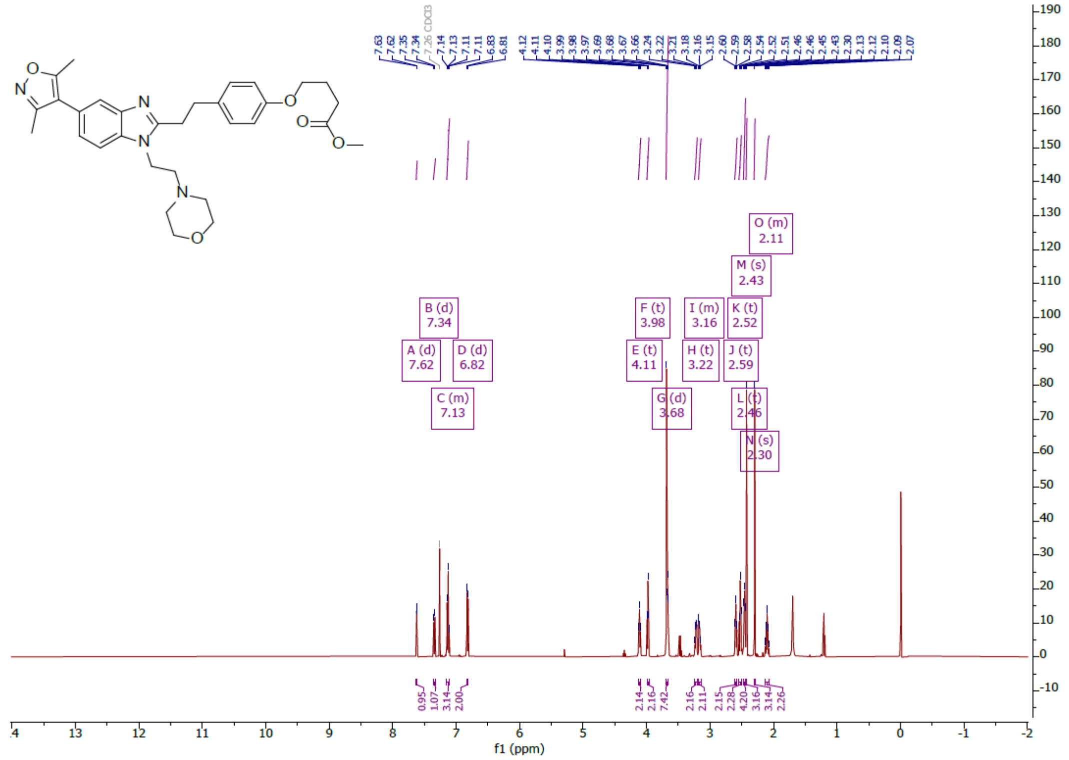

^{1}H NMR spectrum for **8**.

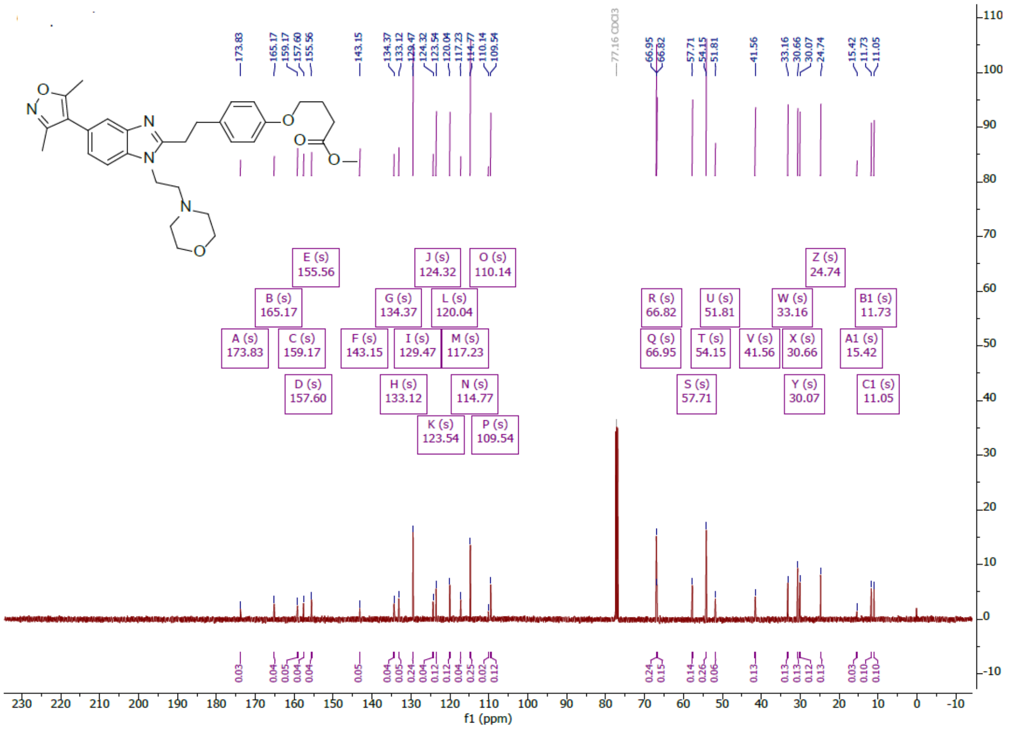

^{13}C NMR spectrum for **8**.

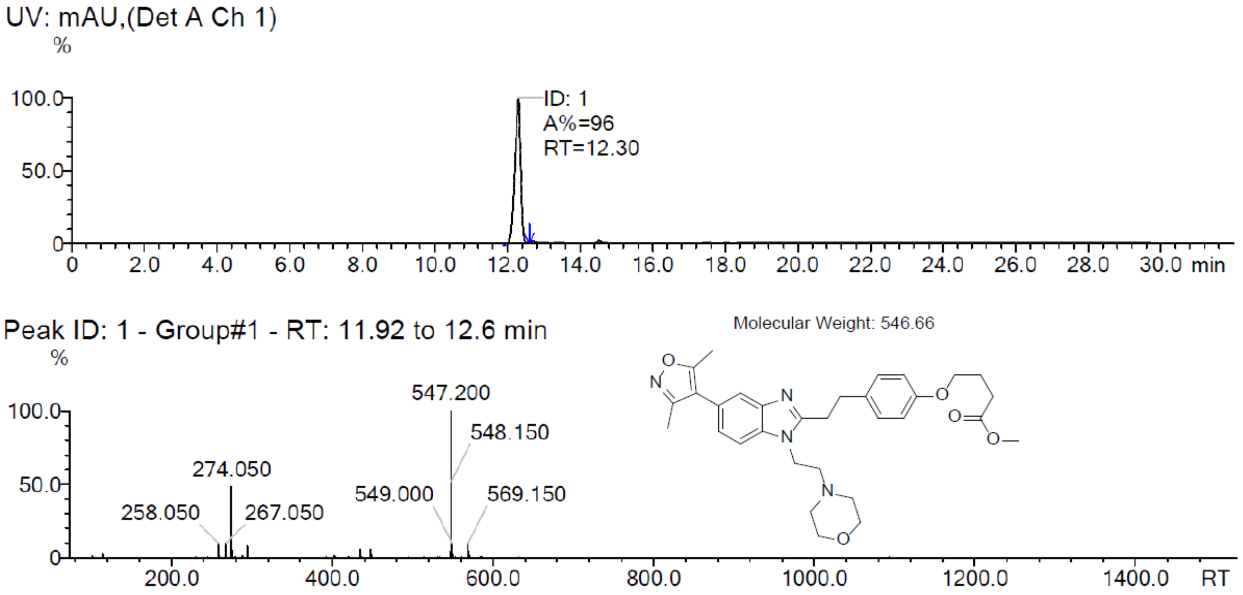

LC MS for Compound **8**.

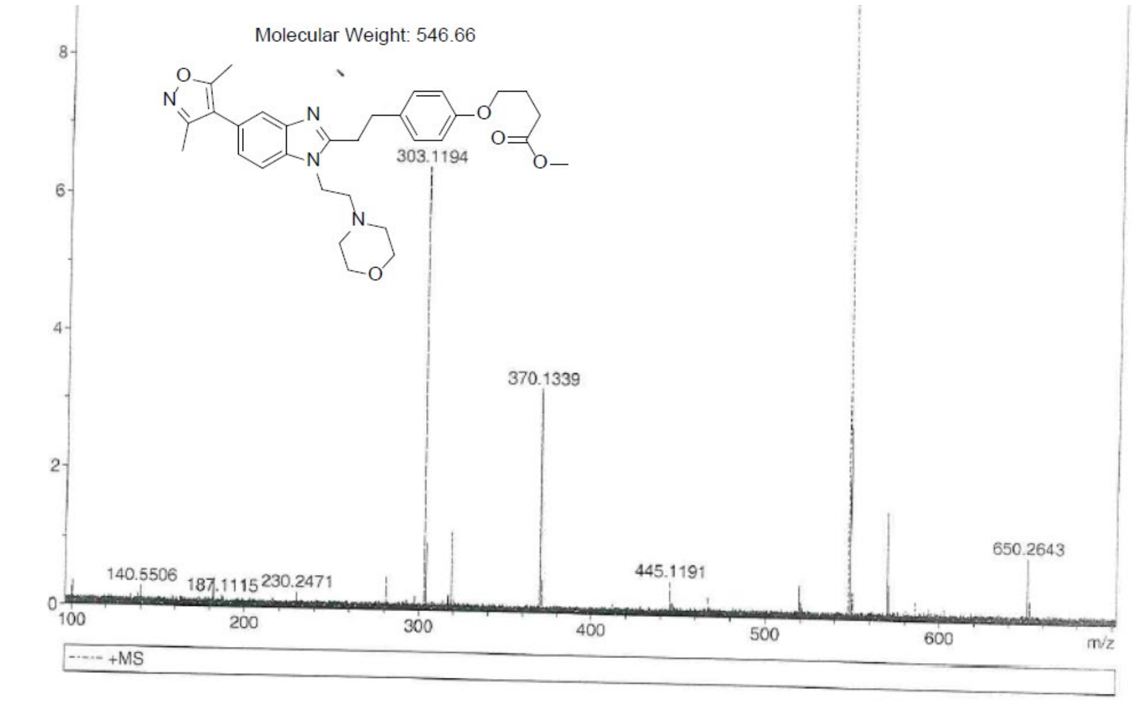

Sum Formula	Sigma	m/z	Err [ppm]	Mean Err [ppm]	Err [mDa]	rdb	N Rule	e⁻
C 31 H 39 N 4 O 5	0.029	547.2915	4.17	3.43	1.88	14.50	ok	even

HRMS for Compound **8**.

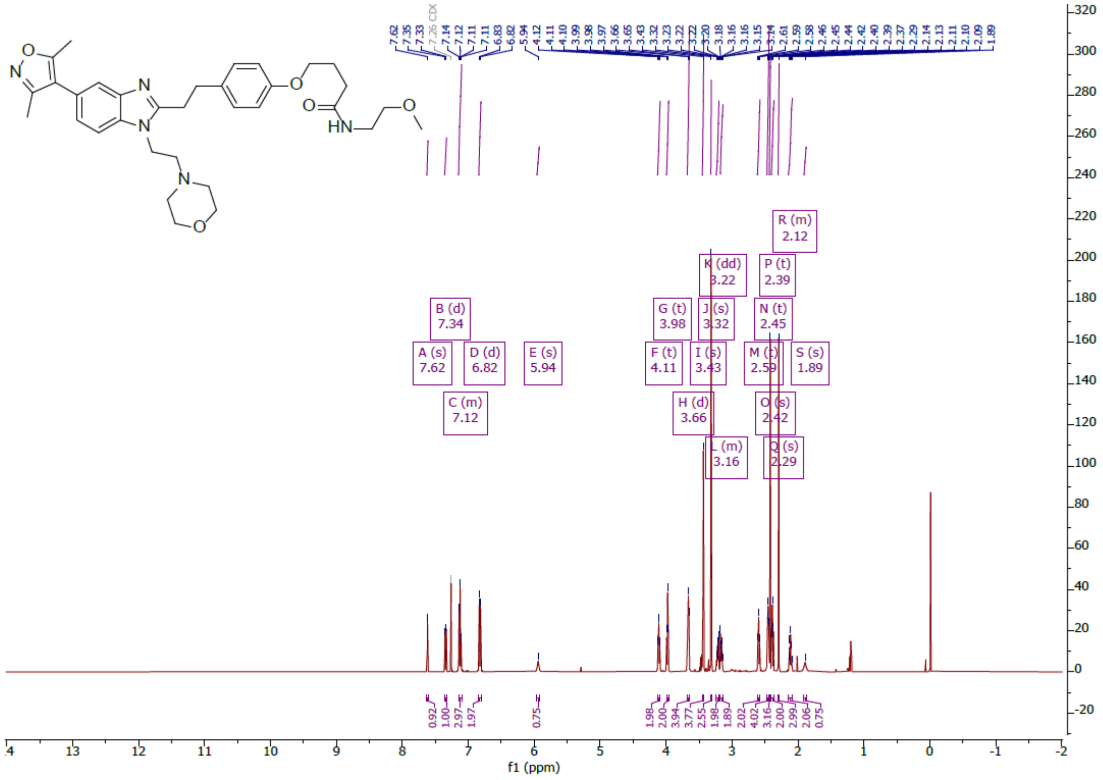

^{1}H NMR spectrum for compound **10**.

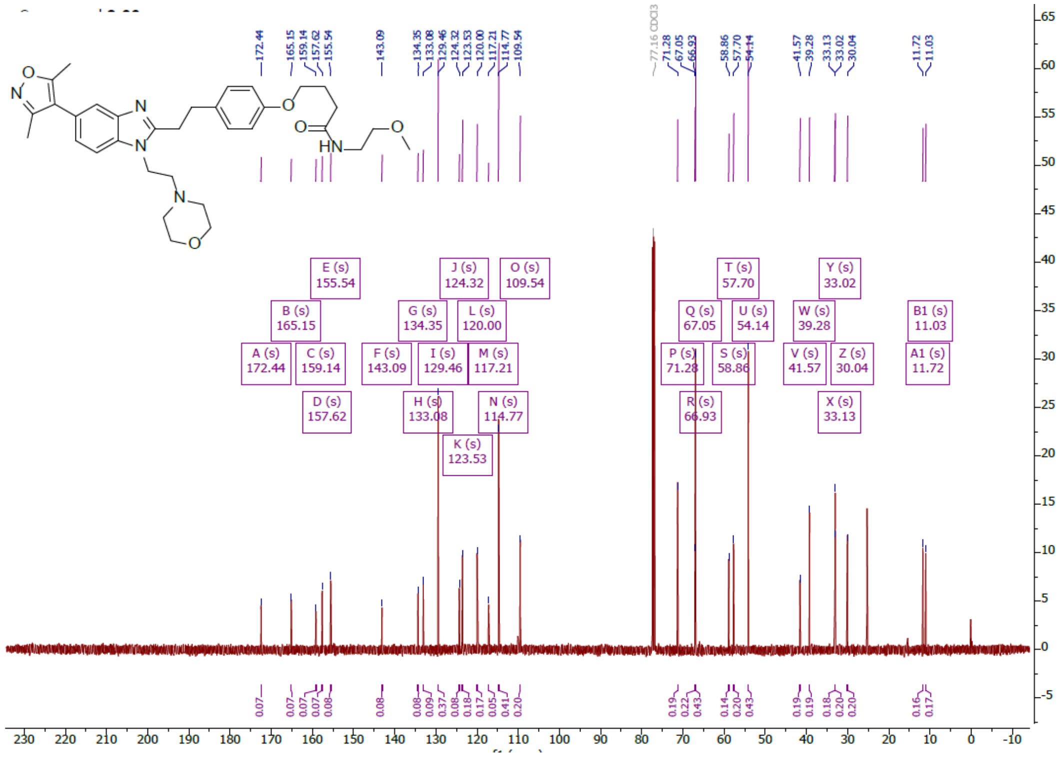

^{13}C spectrum for **10**.

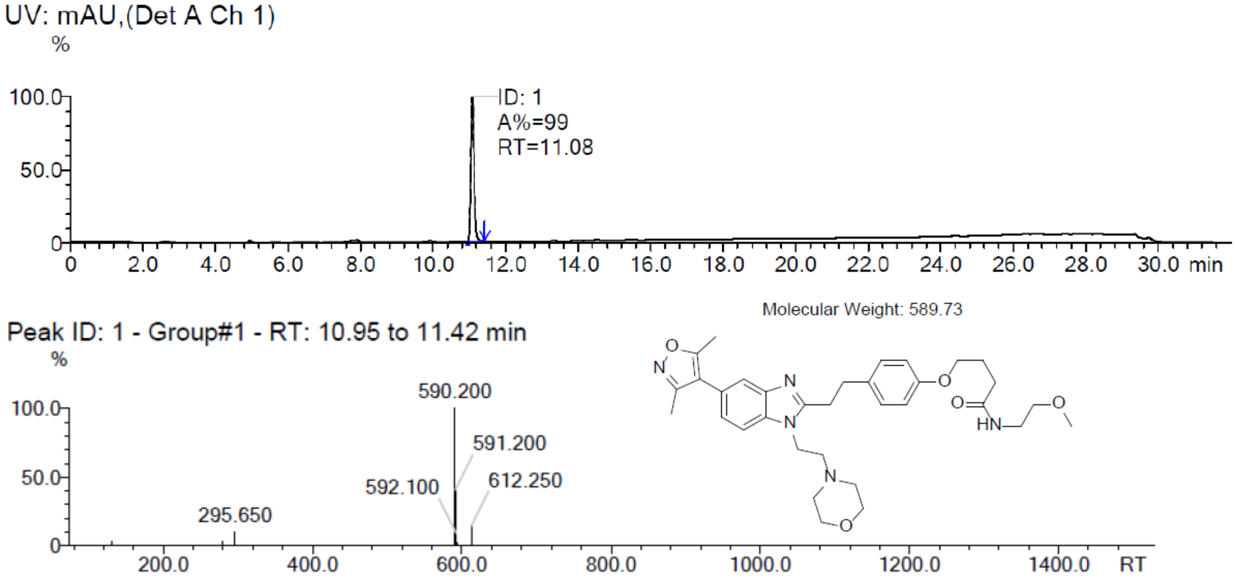

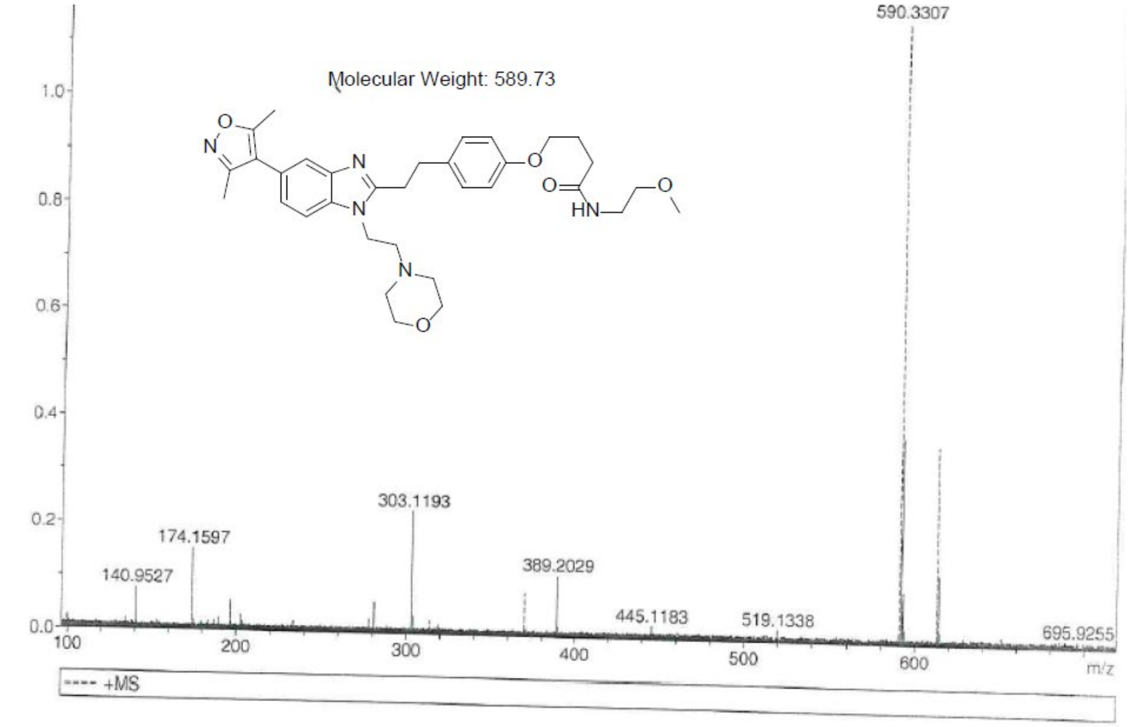

Sum Formula	Sigma	m/z	Err [ppm]	Mean Err [ppm]	Err [mDa]	rdb	N Rule	e⁻
C 33 H 43 N 5 Na 1 O 5	0.019	612.3156	3.54	2.95	1.81	14.50	ok	even

LCMS and HRMS for **10**.

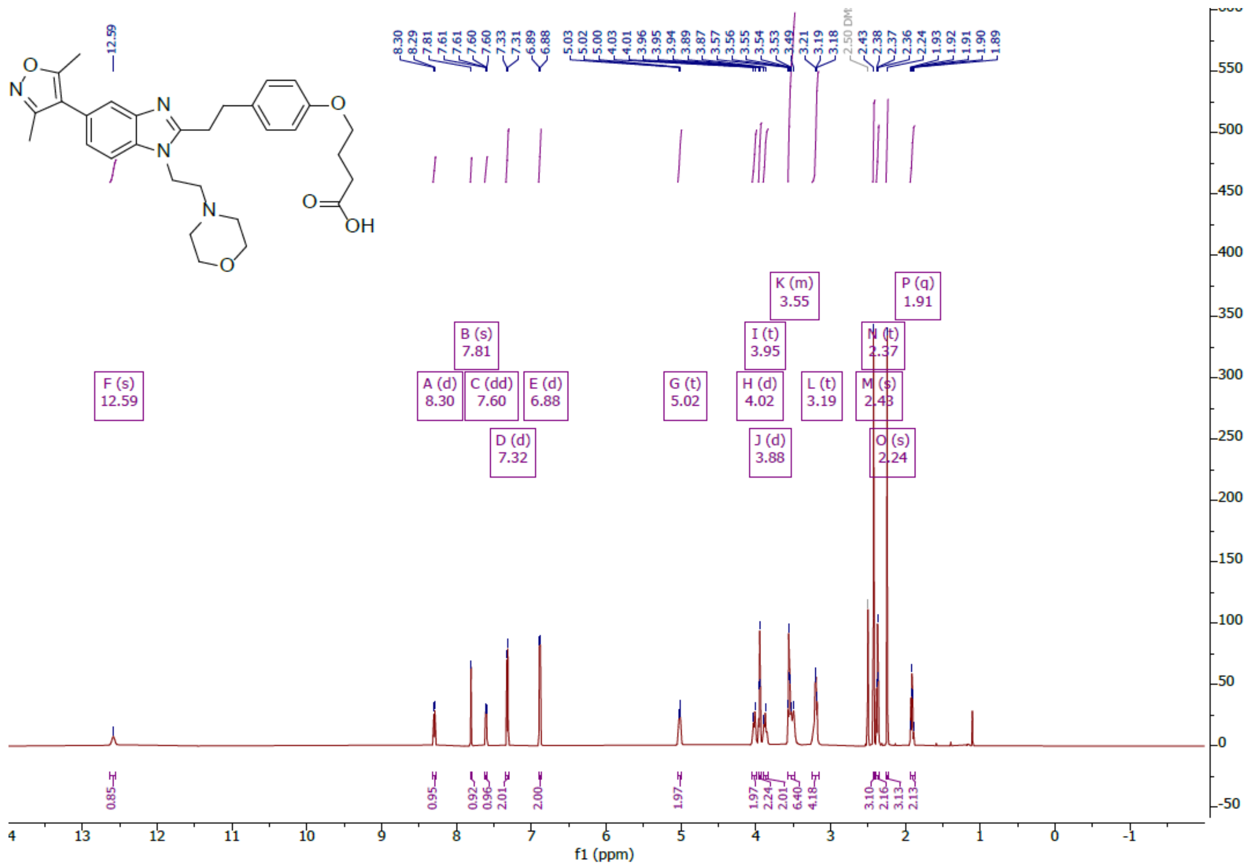

^{1}H NMR spectrum of **12**.

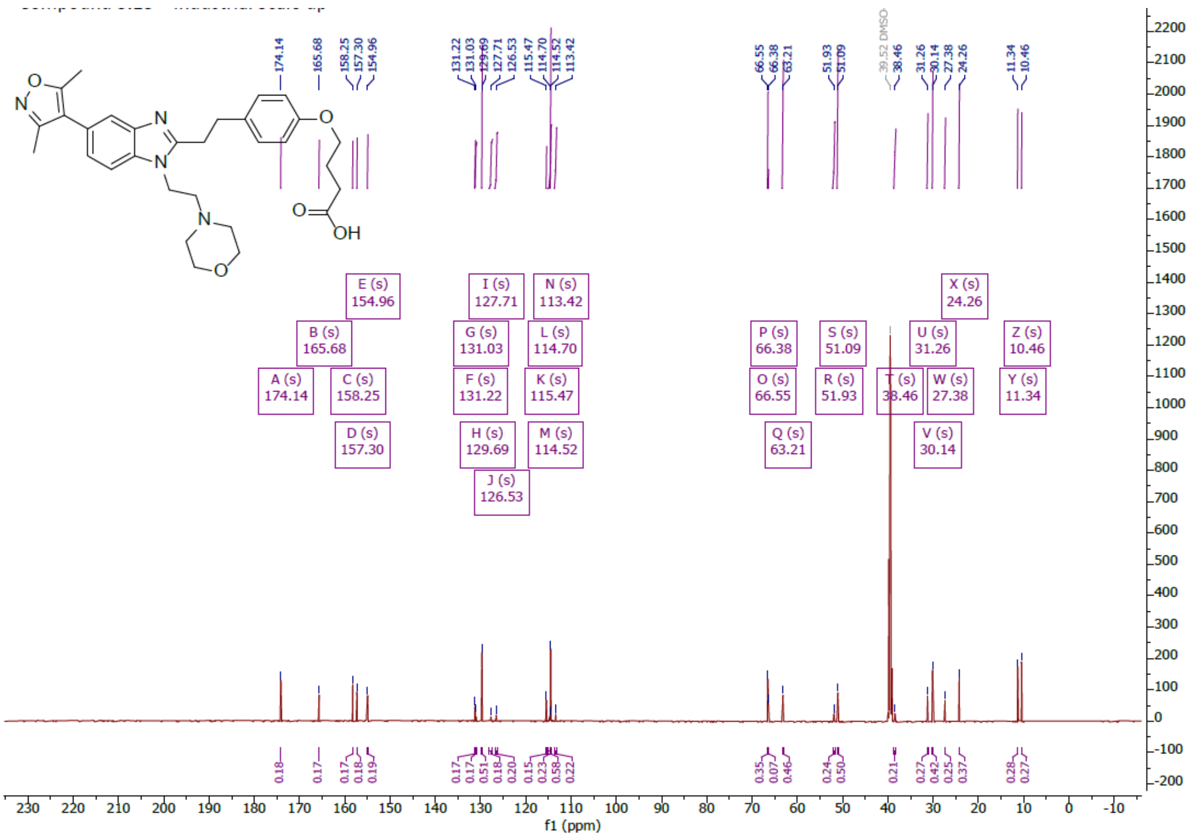

^{13}C NMR spectrum of **12**.

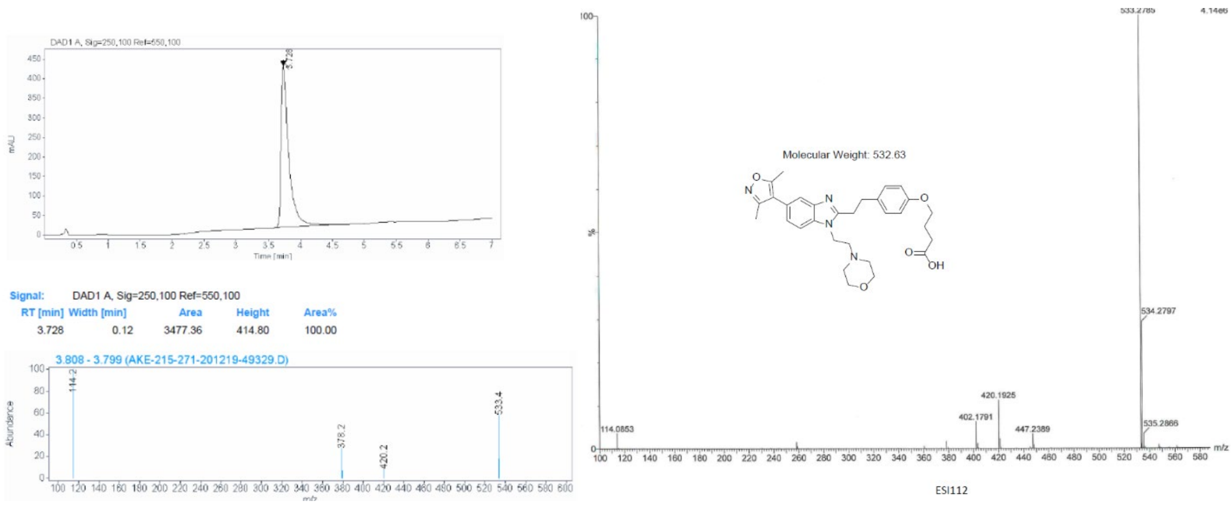

LCMS, HRMS for **12**.

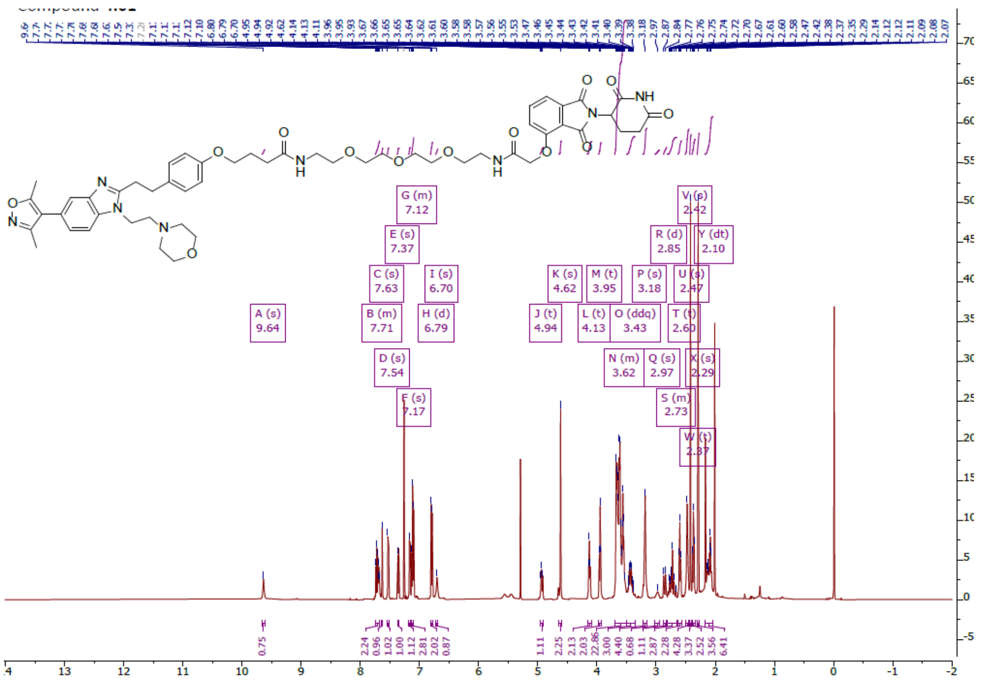

^{1}H NMR spectrum of **13**.

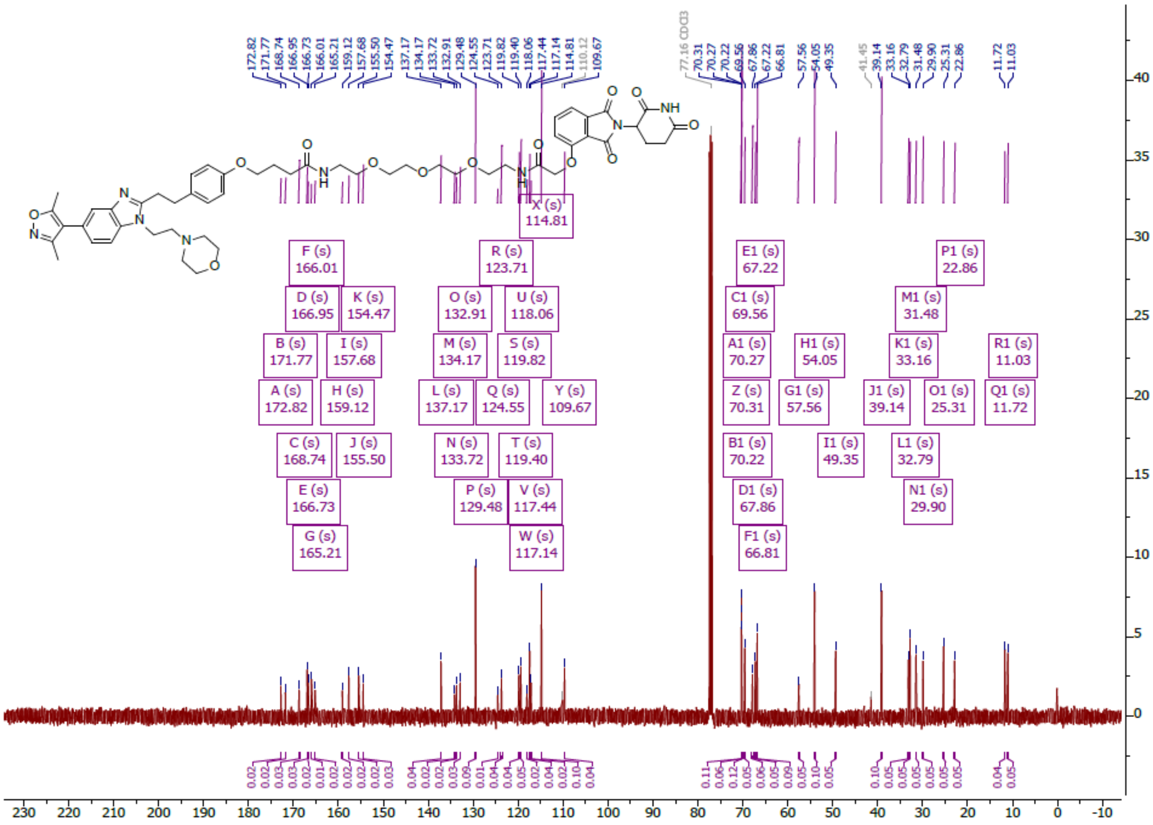

^{13}C NMR spectrum of **13**.

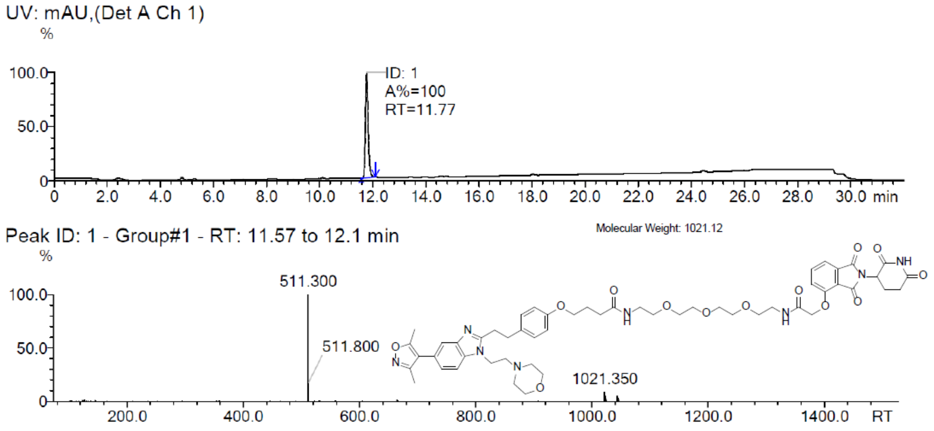

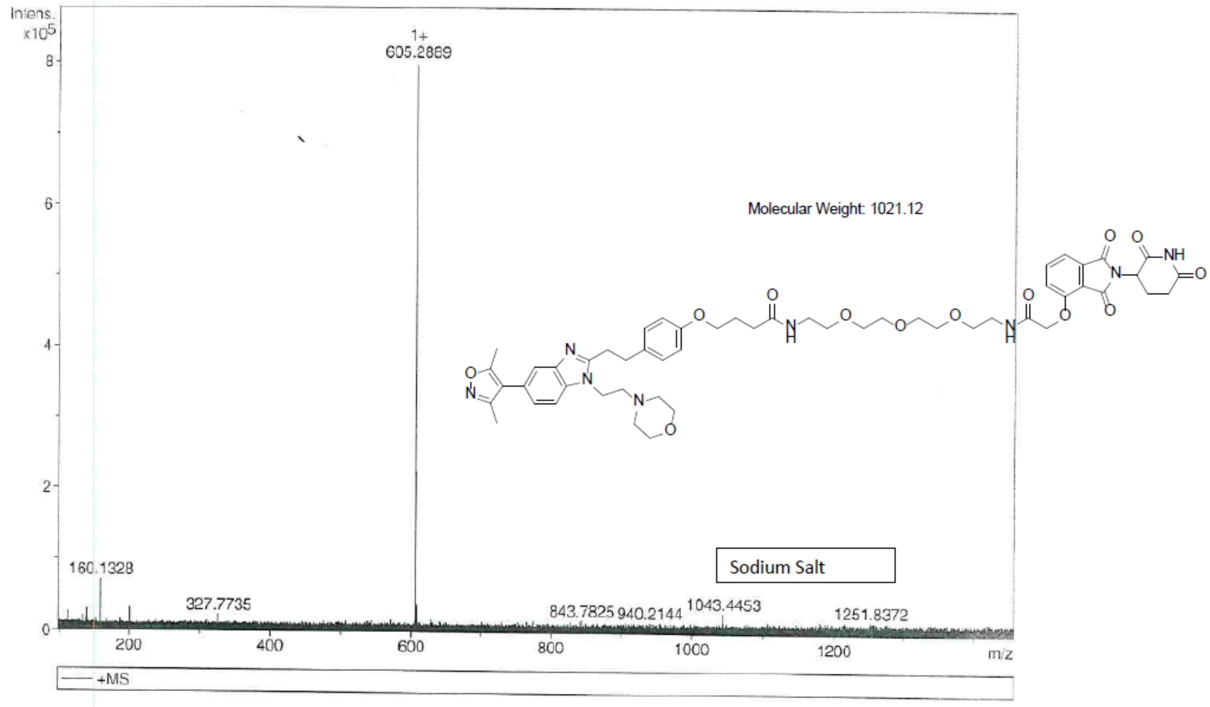

Sum Formula	Sigma	m/z	Err [ppm]	Mean Err [ppm]	Err [mDa]	rdb	N Rule	e⁻
C 53 H 64 N 8 Na 1 O 13	0.099	1043.4485	3.11	3.39	3.54	25.50	ok	even

ESI186

LCMS/HRMS for **13**.

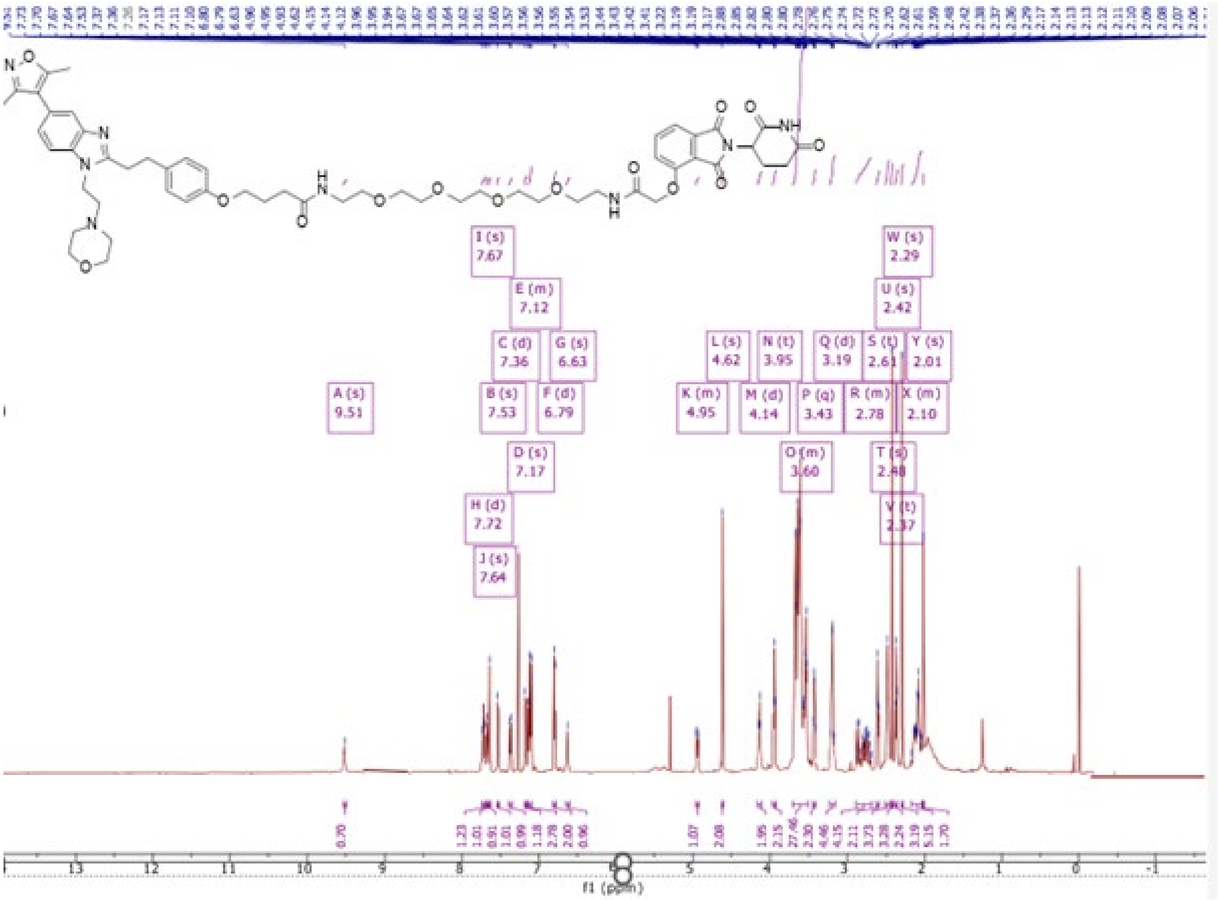

^{1}H NMR spectrum of **14**.

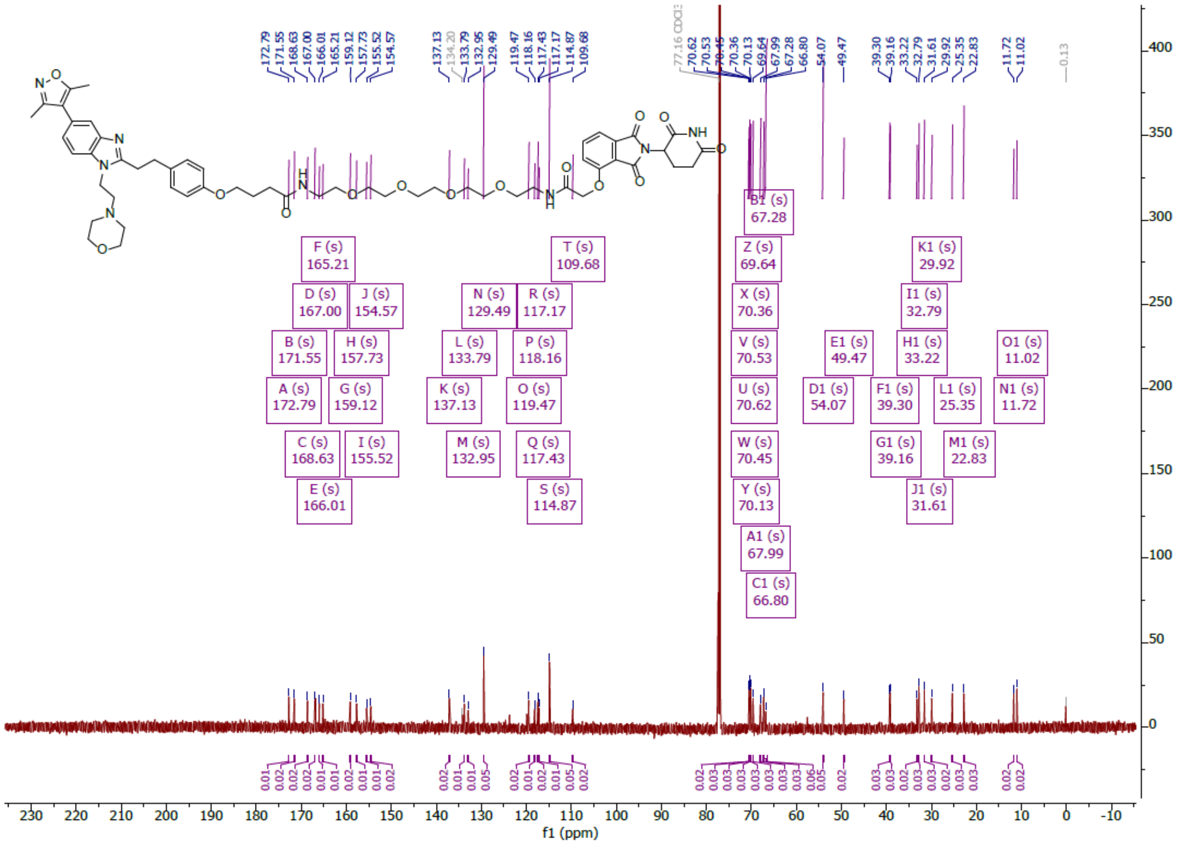

^{13}C spectrum for **14**.

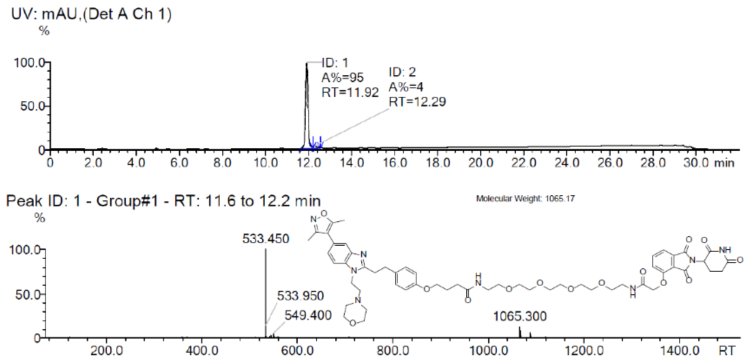

LC/MS for **14**.

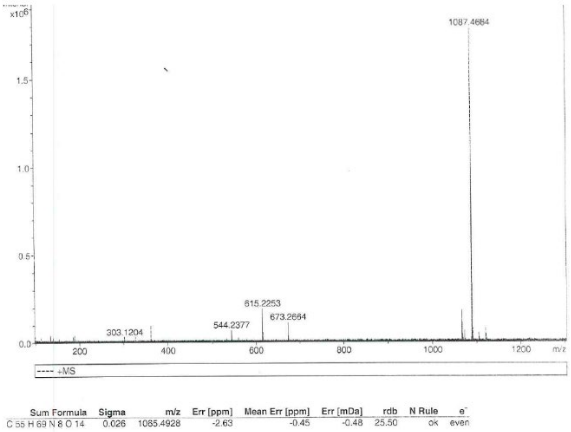

Sum Formula	Sigma	m/z	Err [ppm]	Mean Err [ppm]	Err [mDa]	rdb	N Rule	e⁻
C 55 H 69 N 8 O 14	0.026	1085.4928	-2.63	-0.45	-0.48	25.50	ok	even

HRMS for **14**.

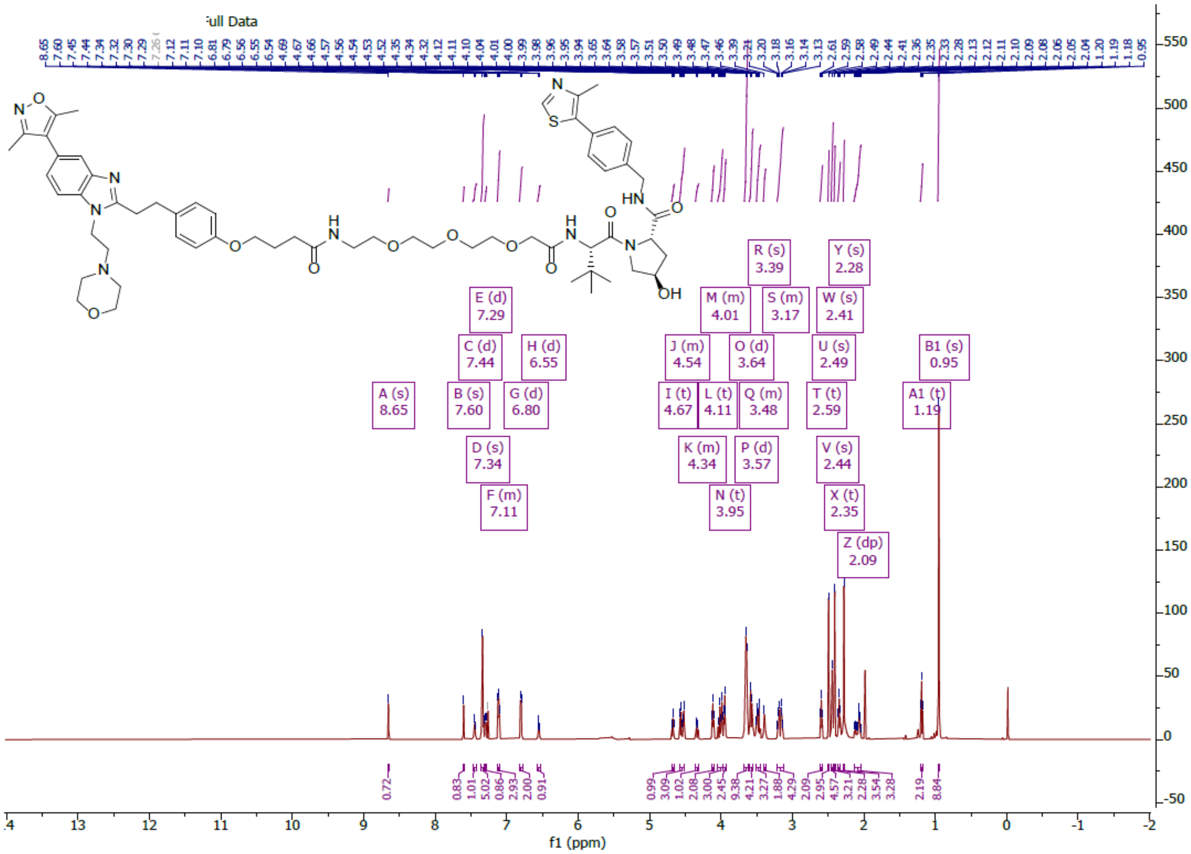

^{1}HNMR spectrum for **15**.

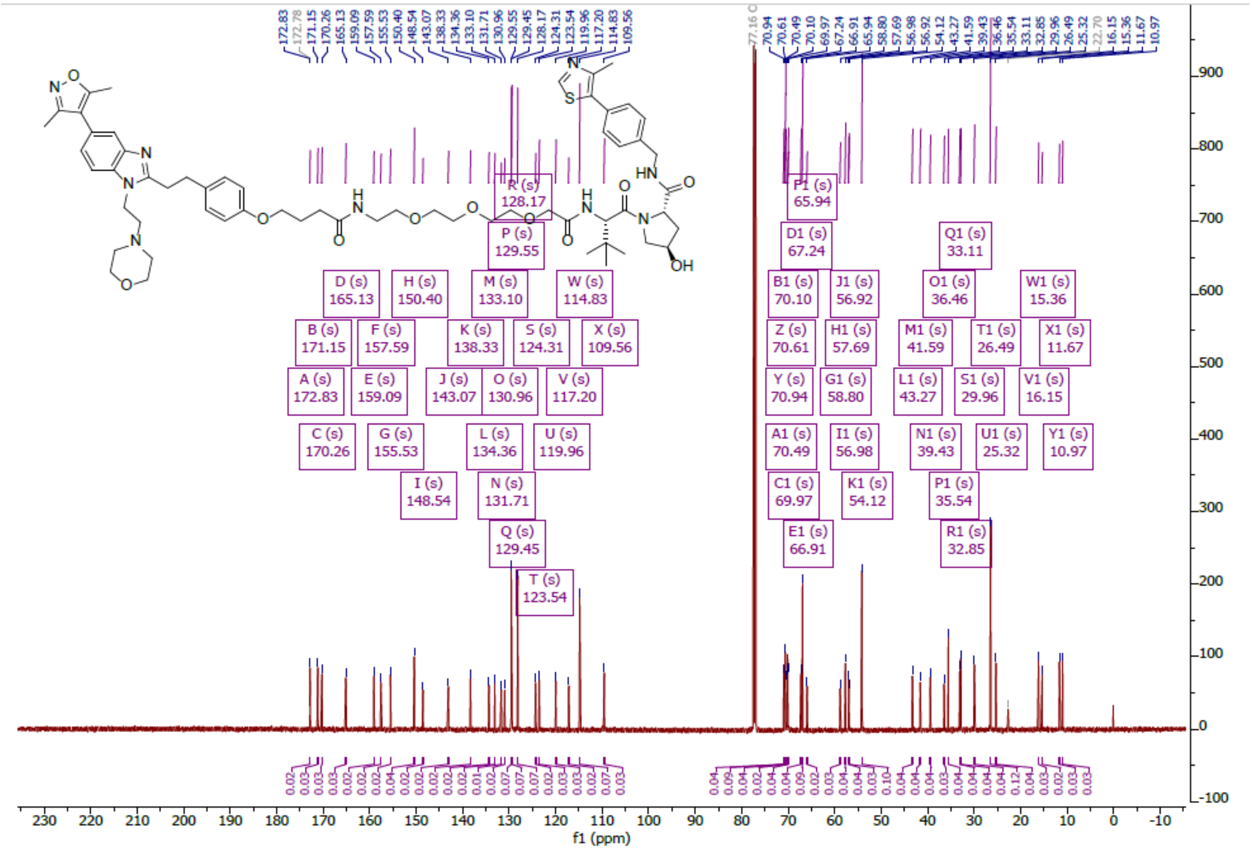

^{13}C NMR spectrum for **15**.

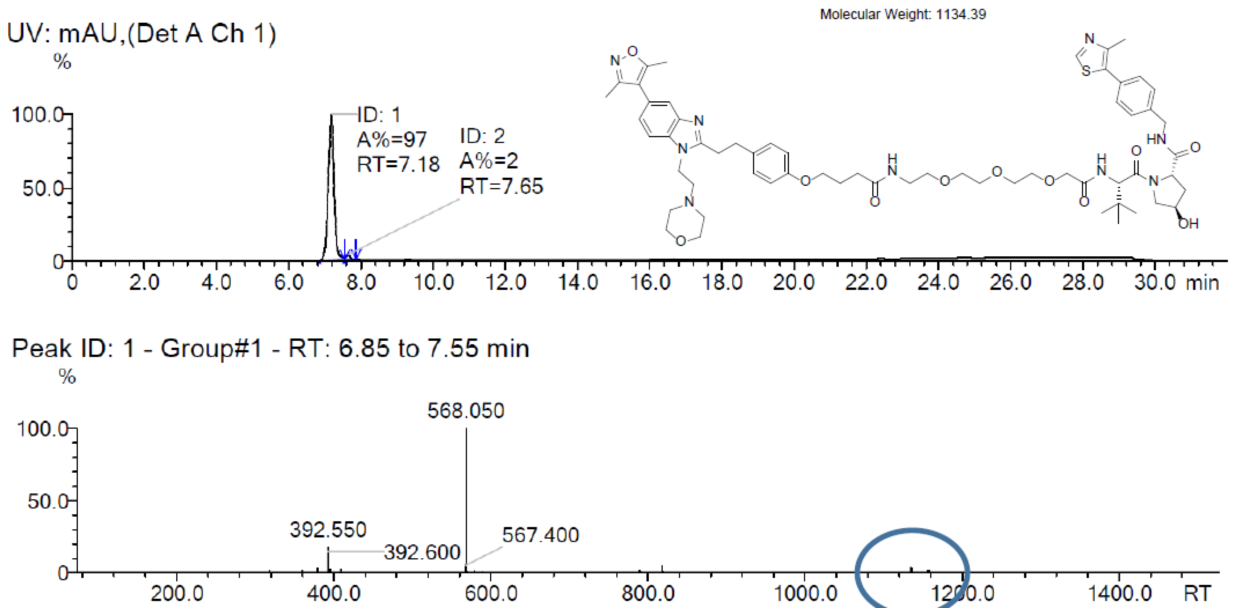

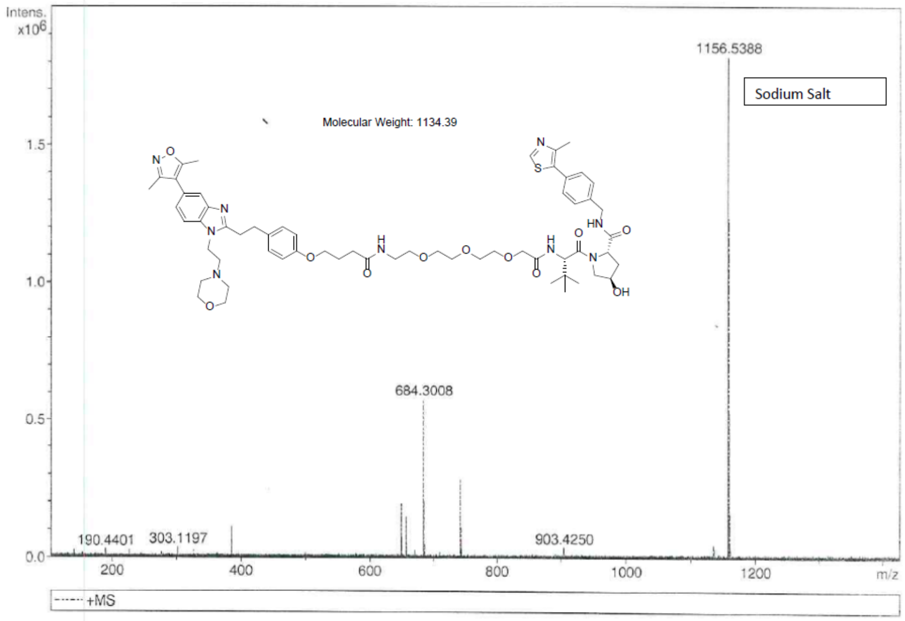

LCMS/HRMS for **15**.

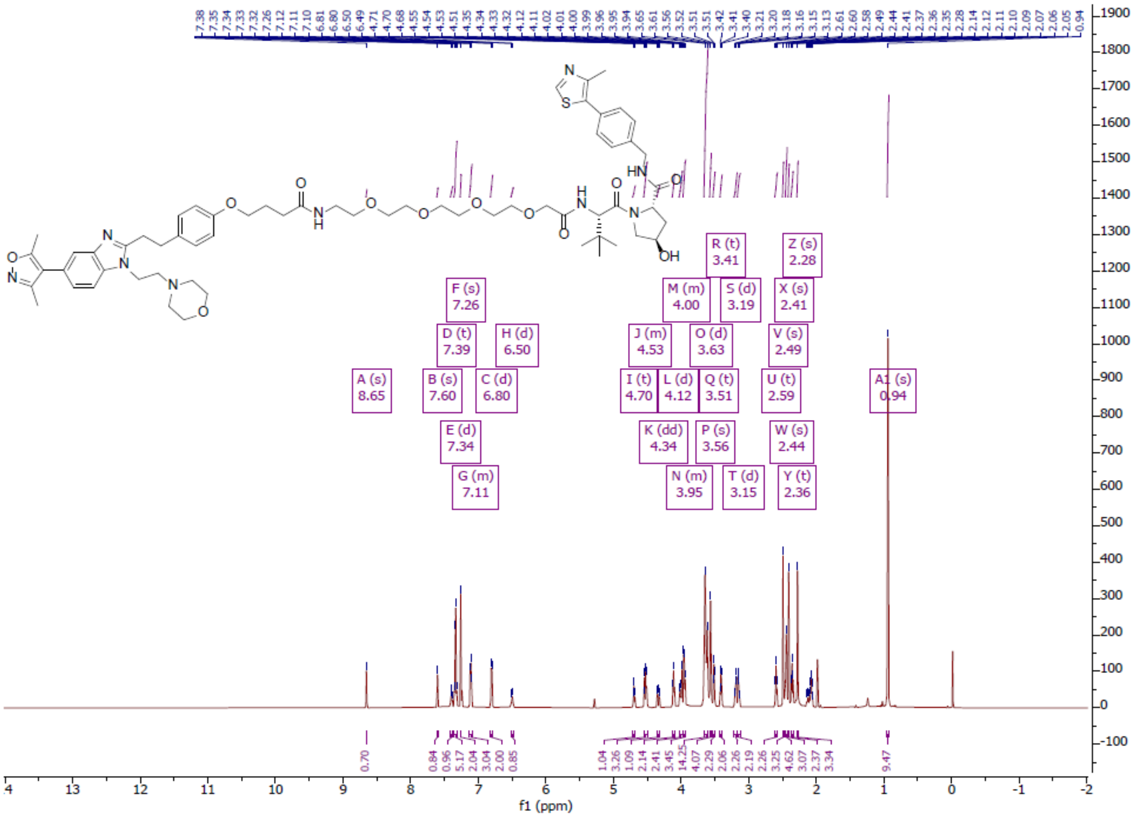

^{1}H NMR spectrum for **16**.

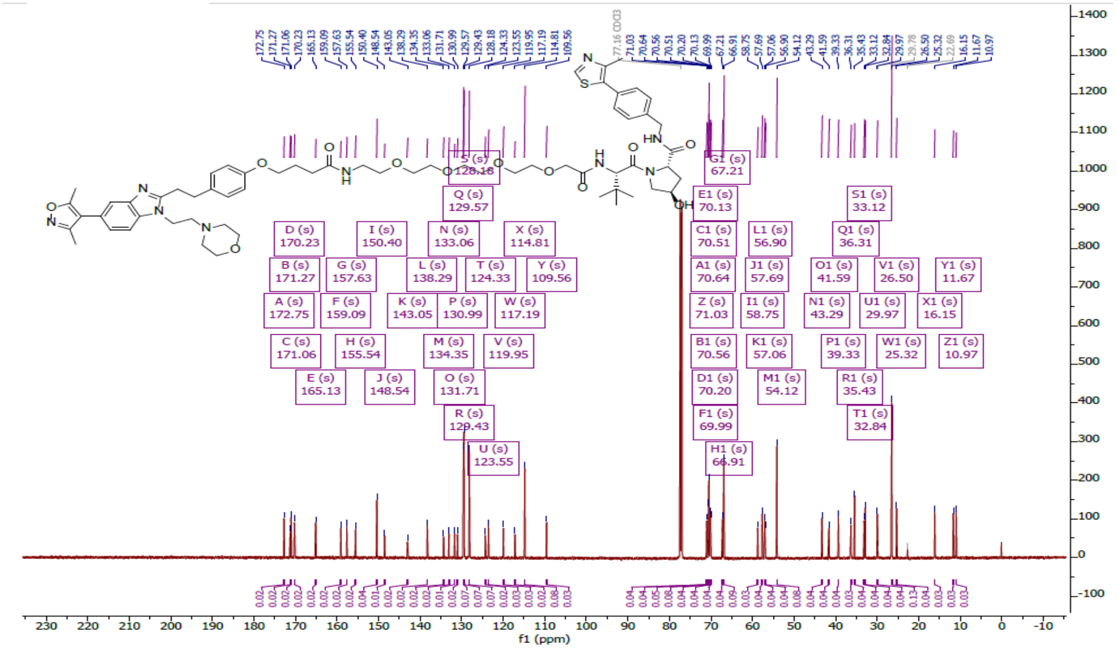

^{13}C NMR spectrum for **16**.

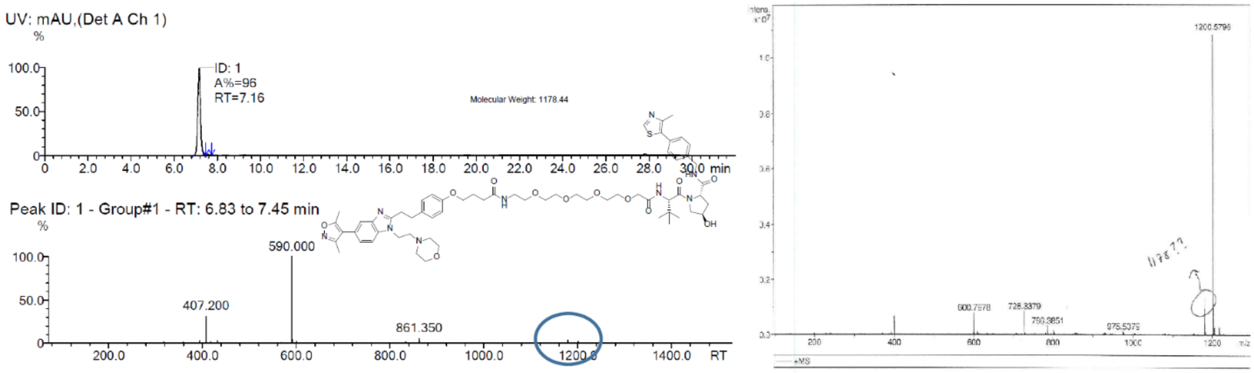

LCMS/HRMS for **16**.

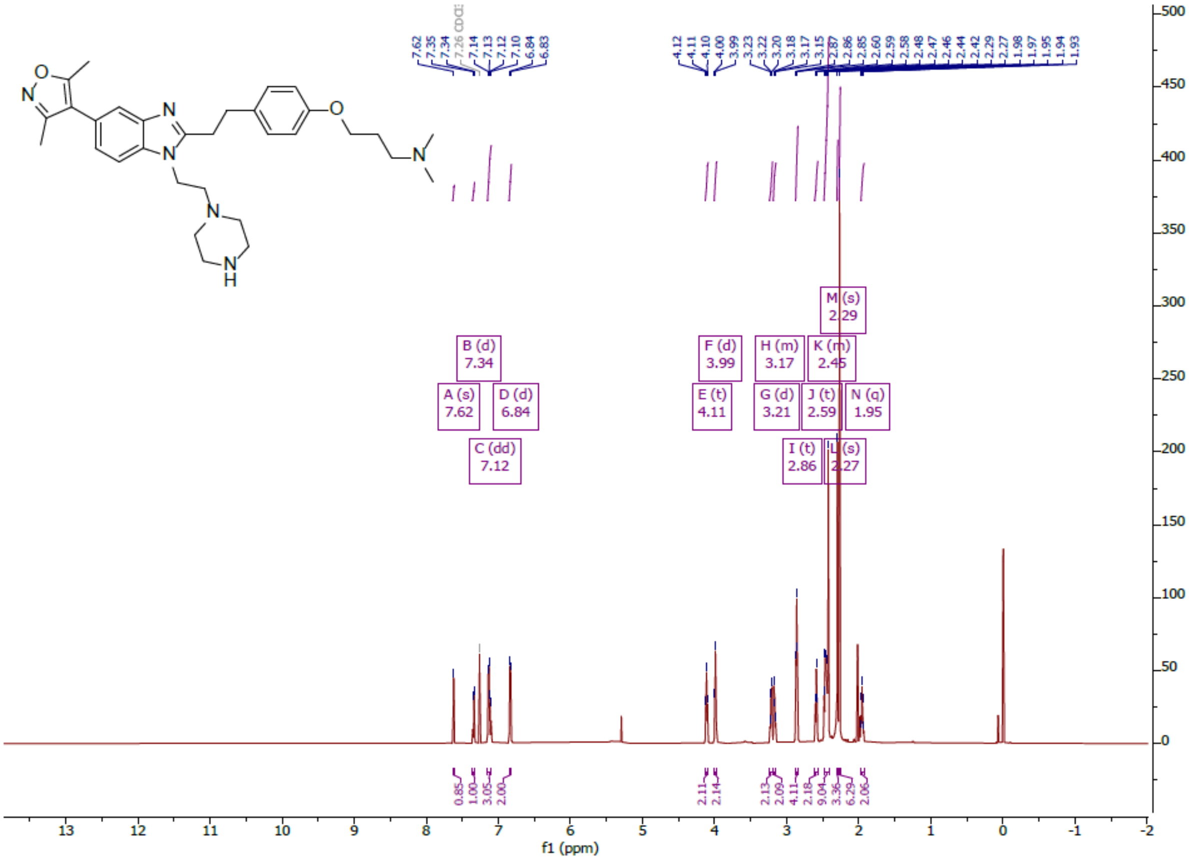

^{1}H NMR spectrum for **21**.

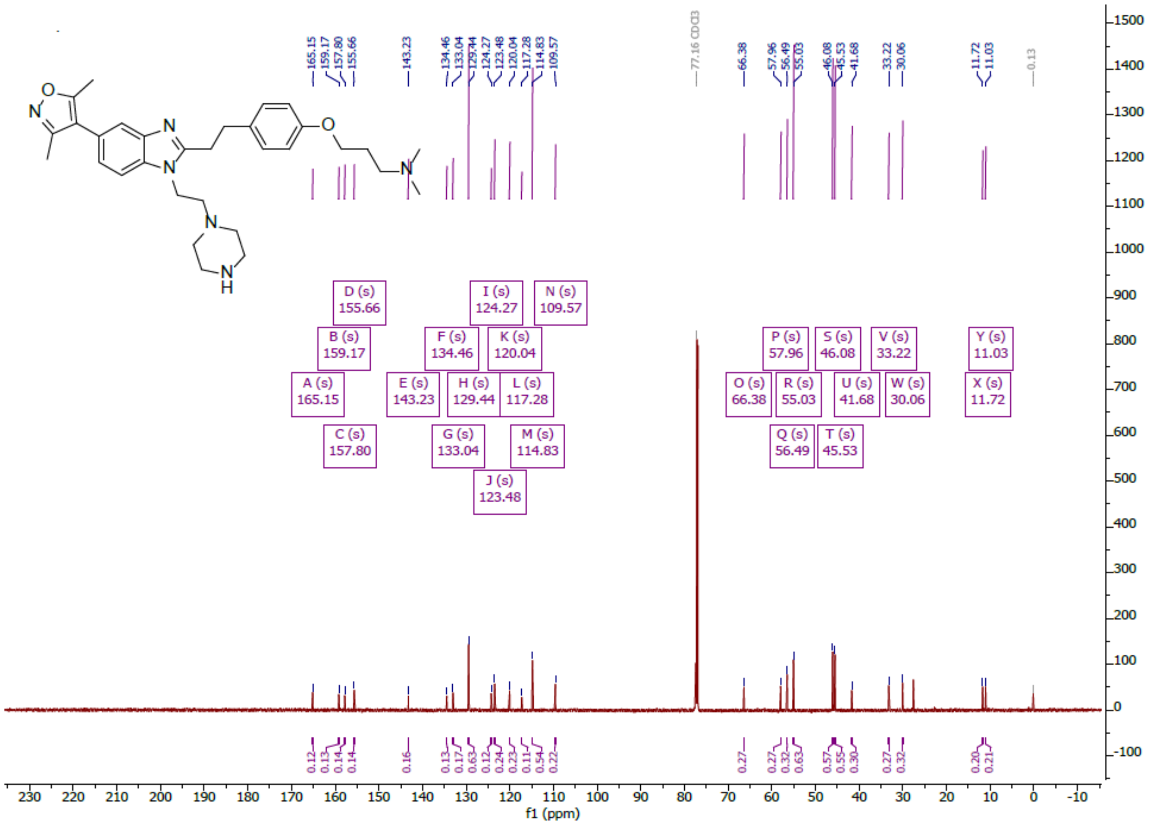

^{13}C NMR spectrum for **21**.

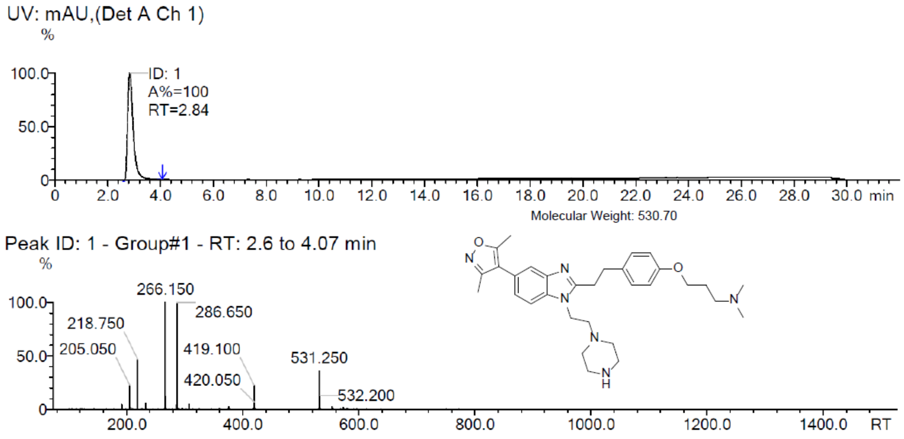

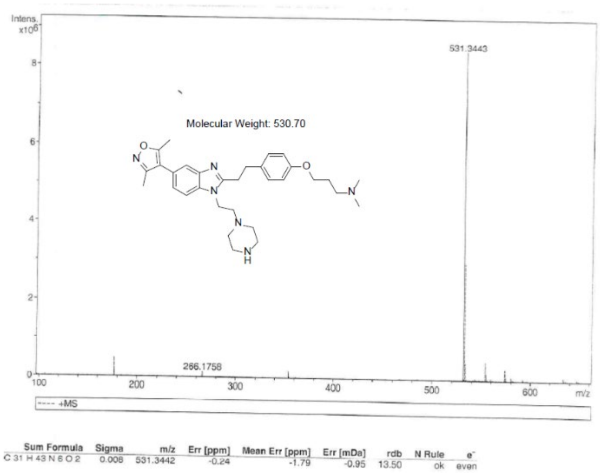

LCMS/HRMS for **21**.

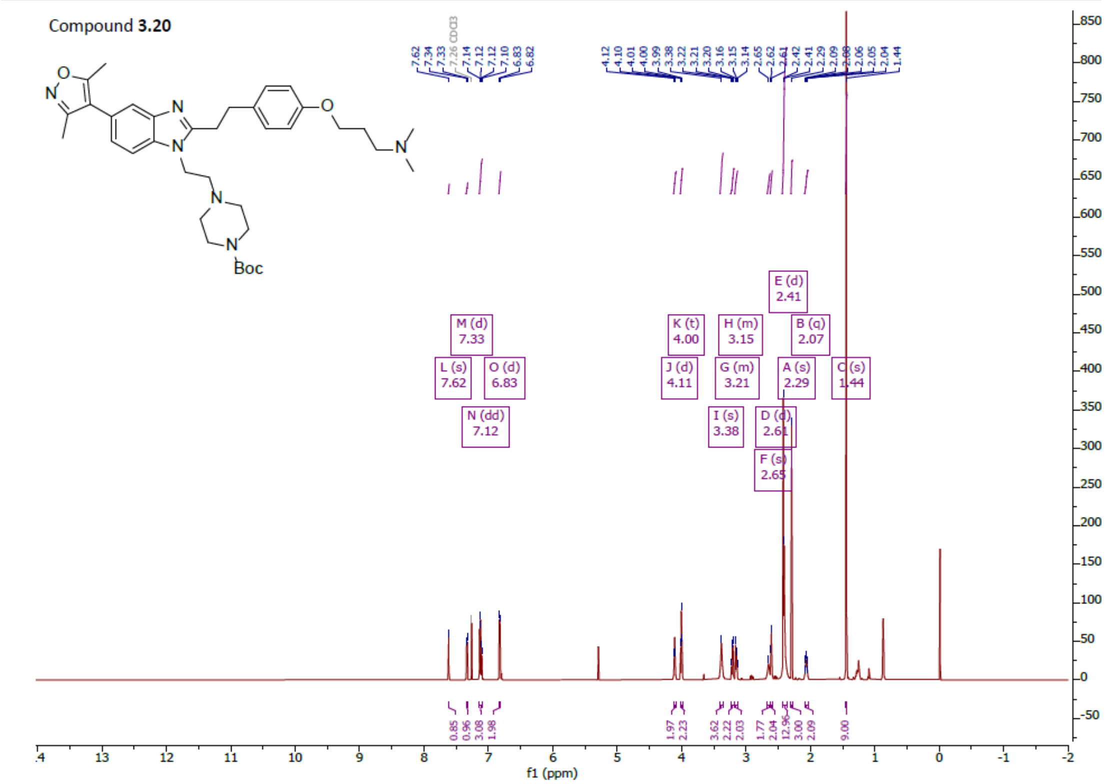

^{1}H NMR spectrum for **22**.

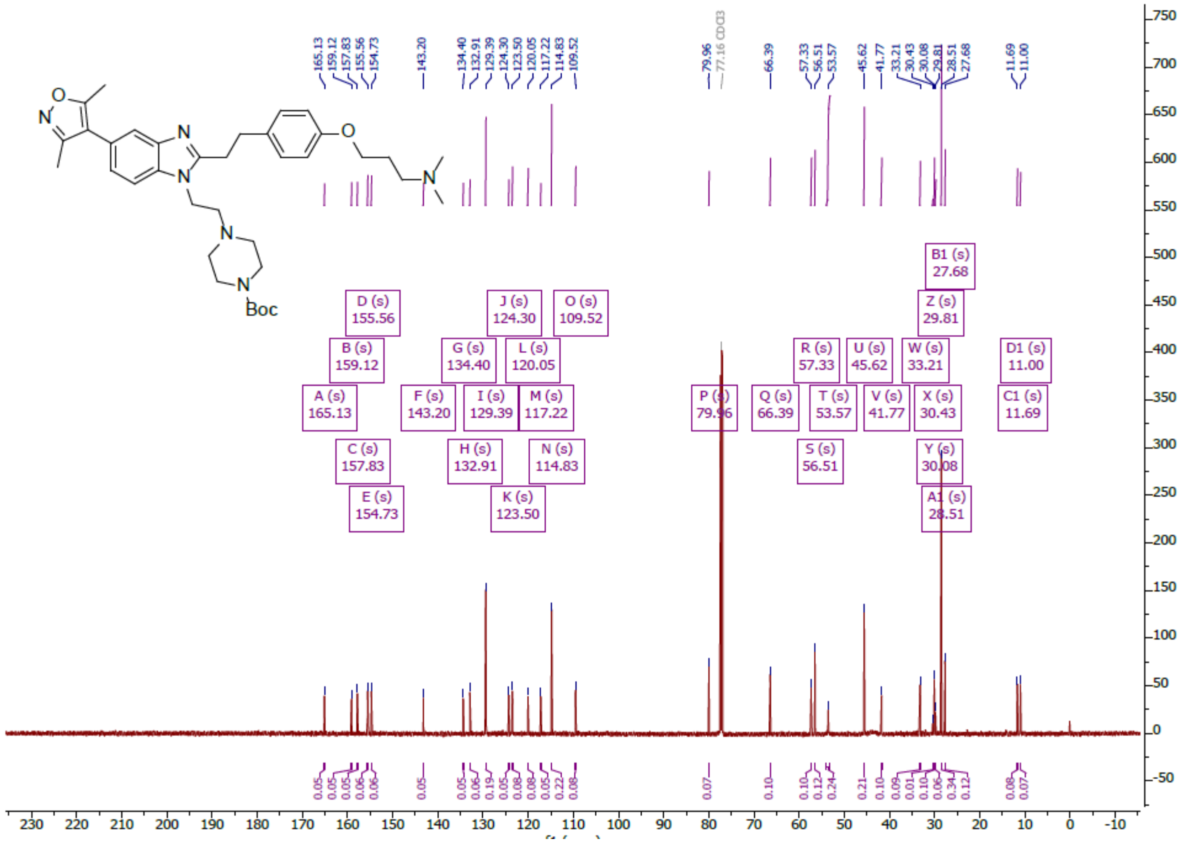

^{13}C NMR spectrum for **22**.

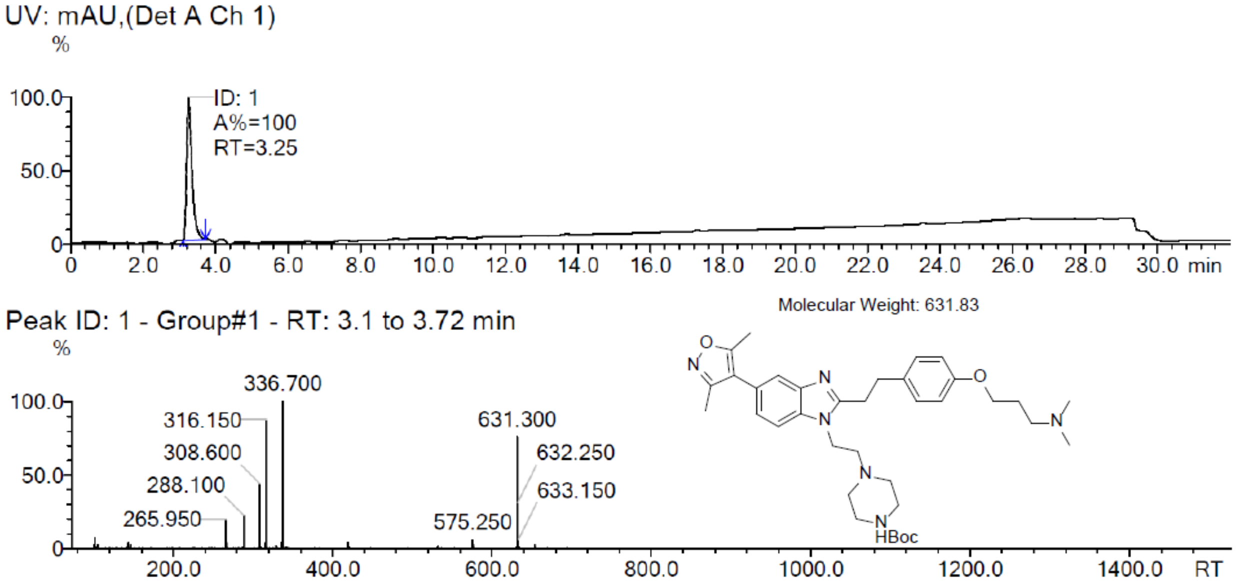

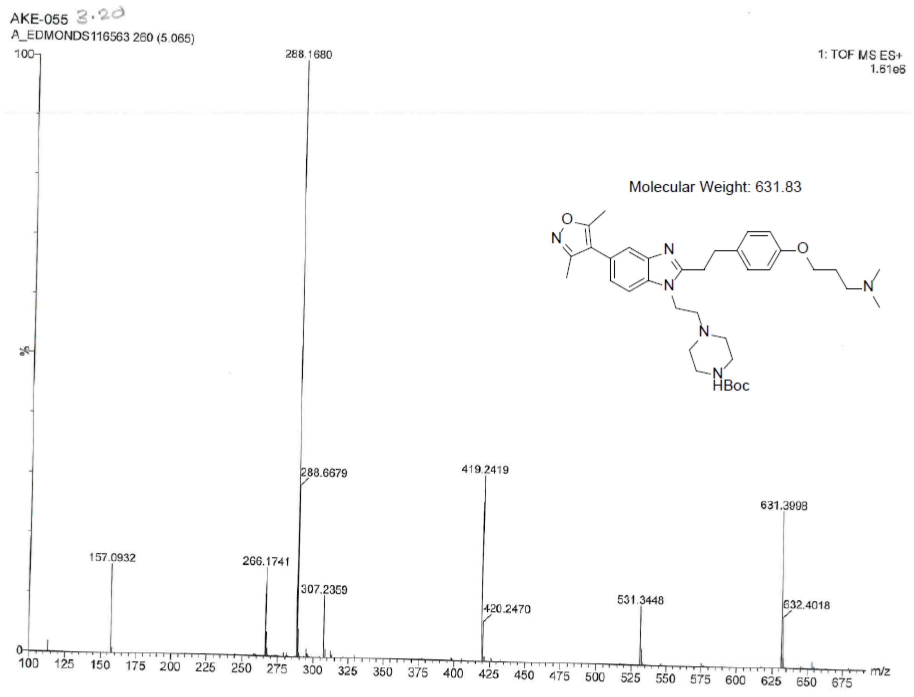

LCMS/HRMS for **22**.

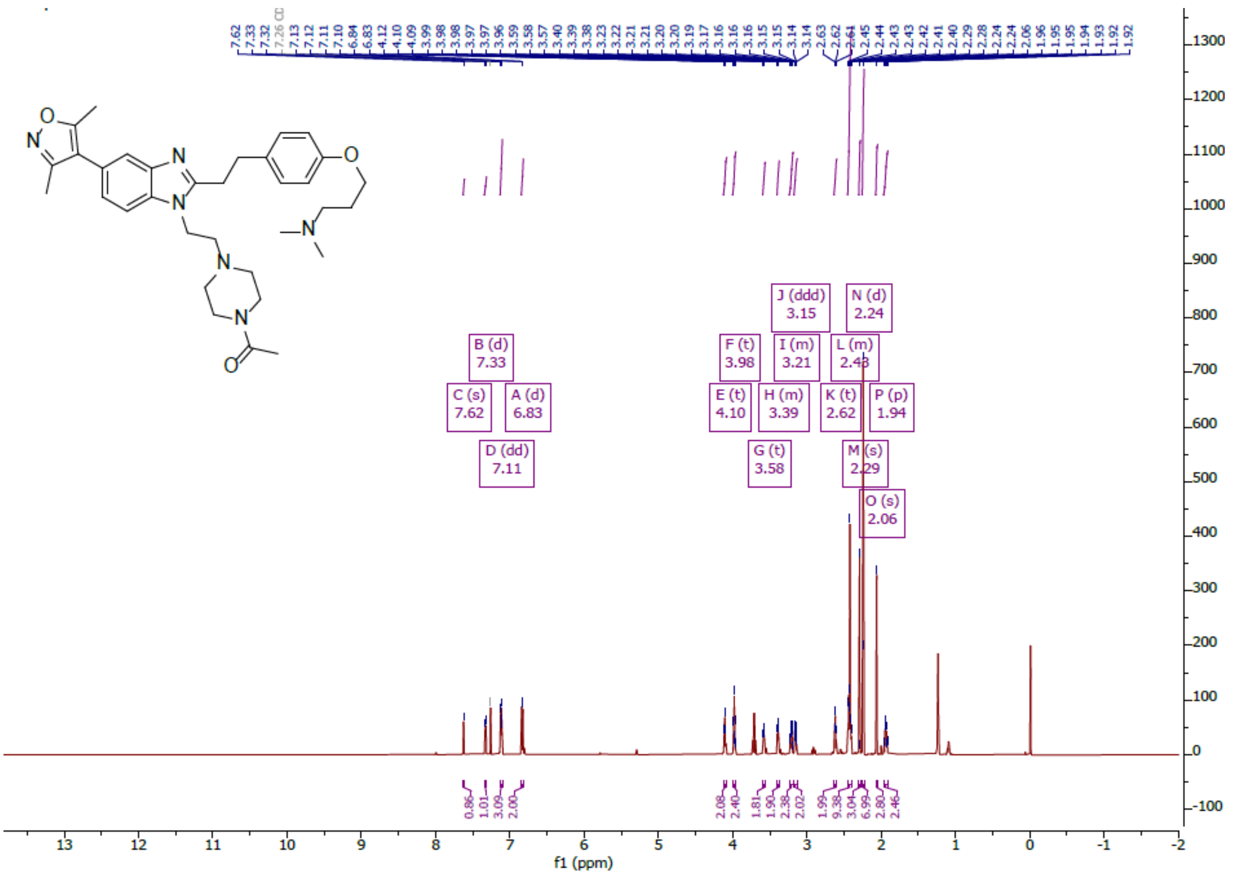

^{1}H NMR spectrum for **23**.

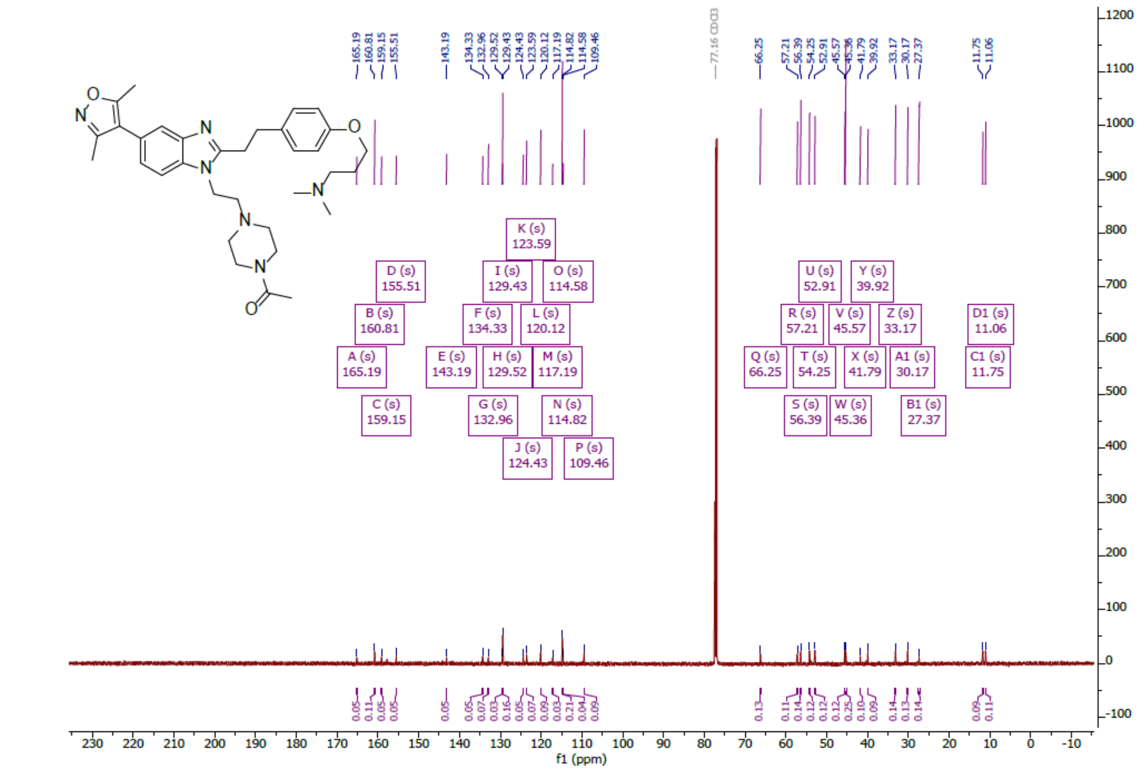

^{13}C NMR spectrum for **23**.

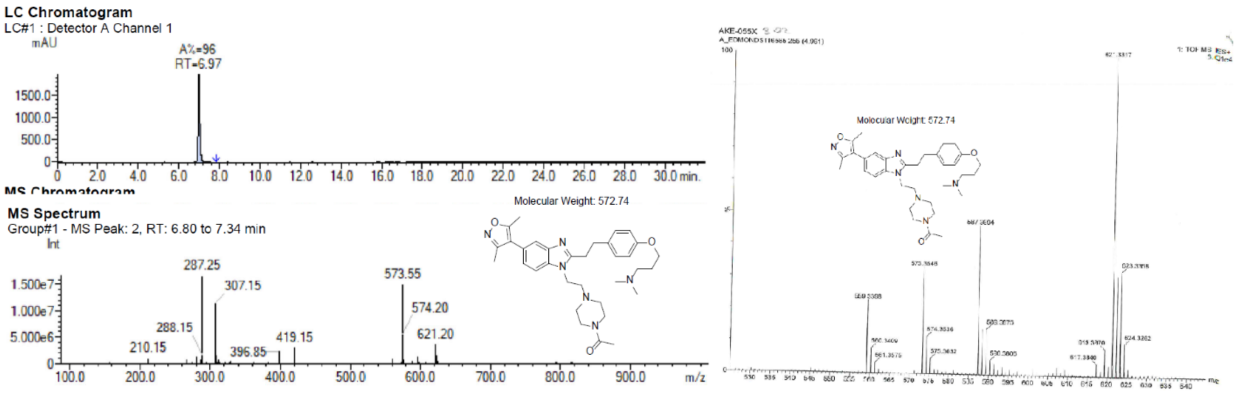

LCMS/HRMS for **23**.

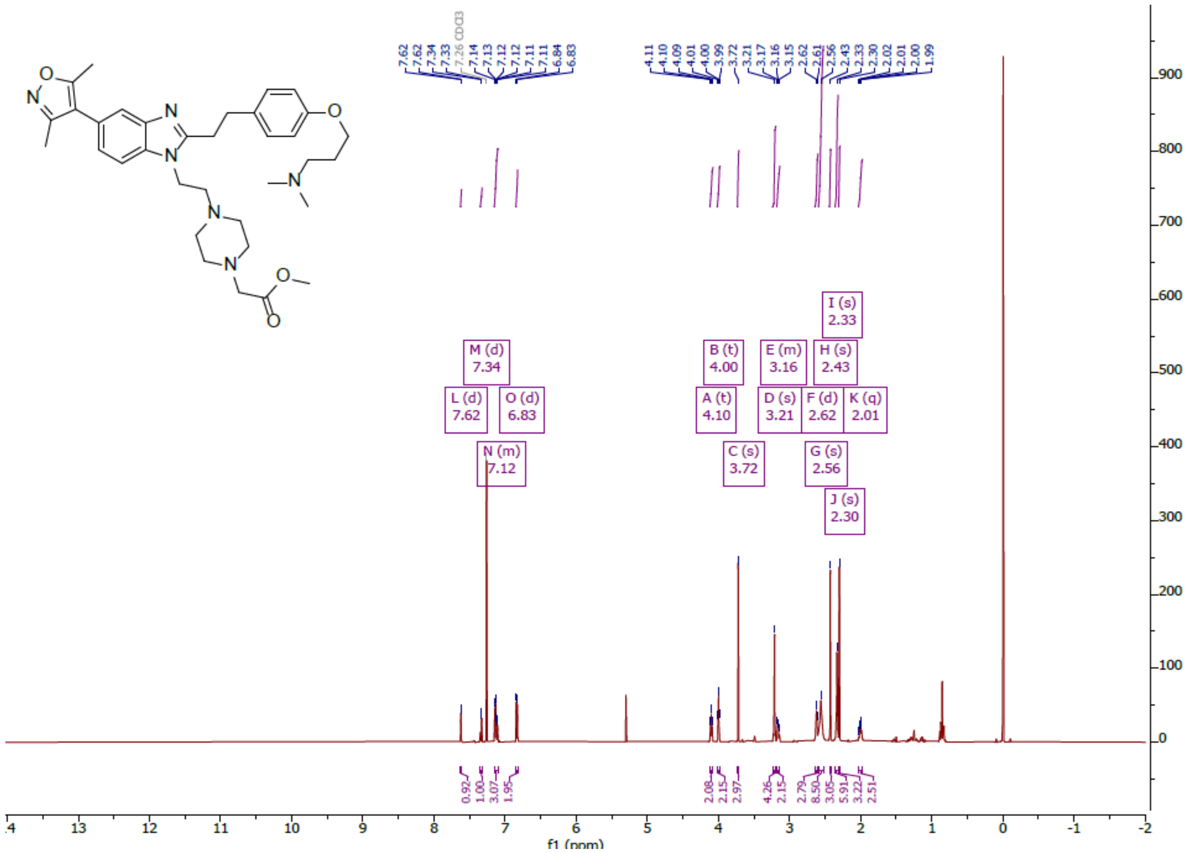

1H NMR spectrum for **28**.

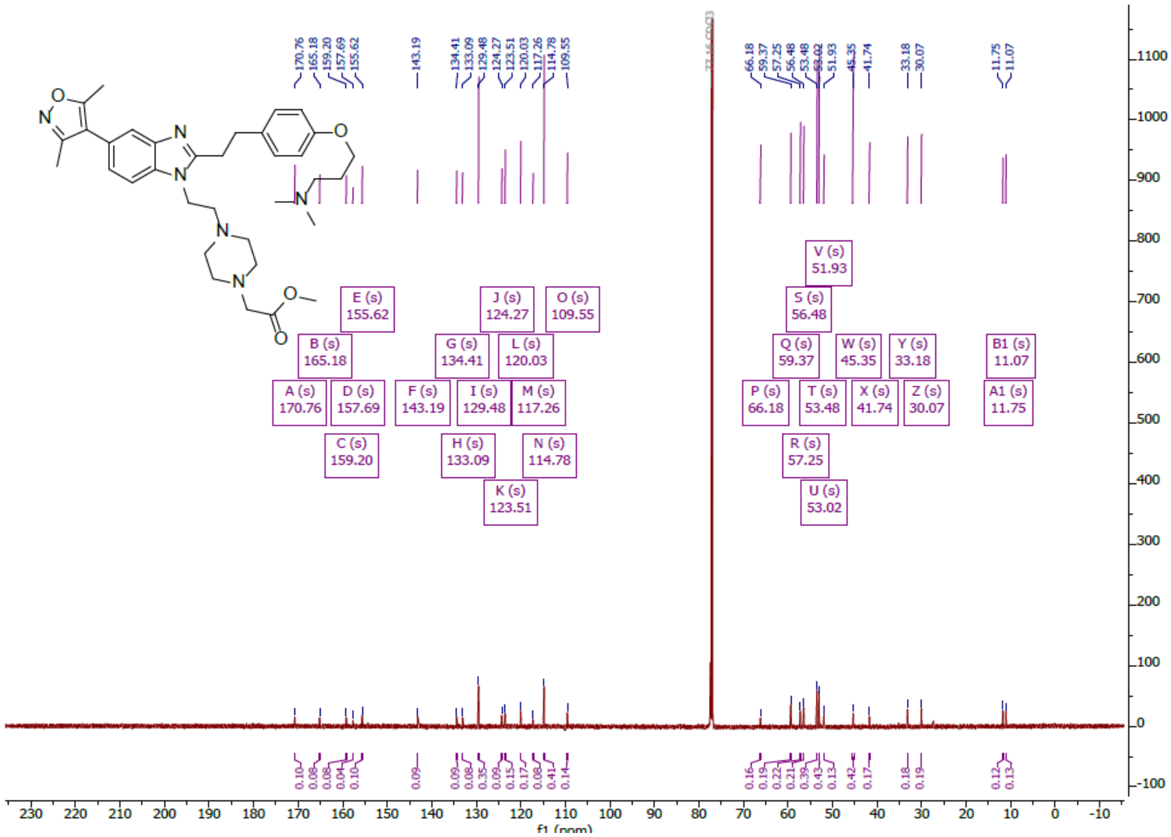

^{13}C spectrum for **28**

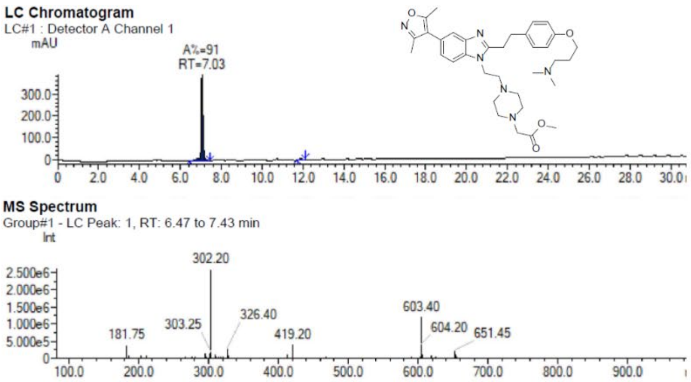

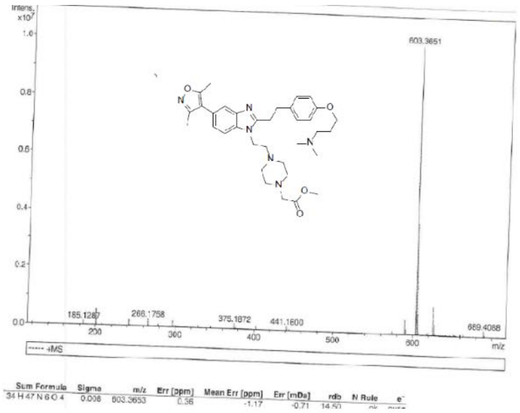

LCMS/HRMS for **28**.

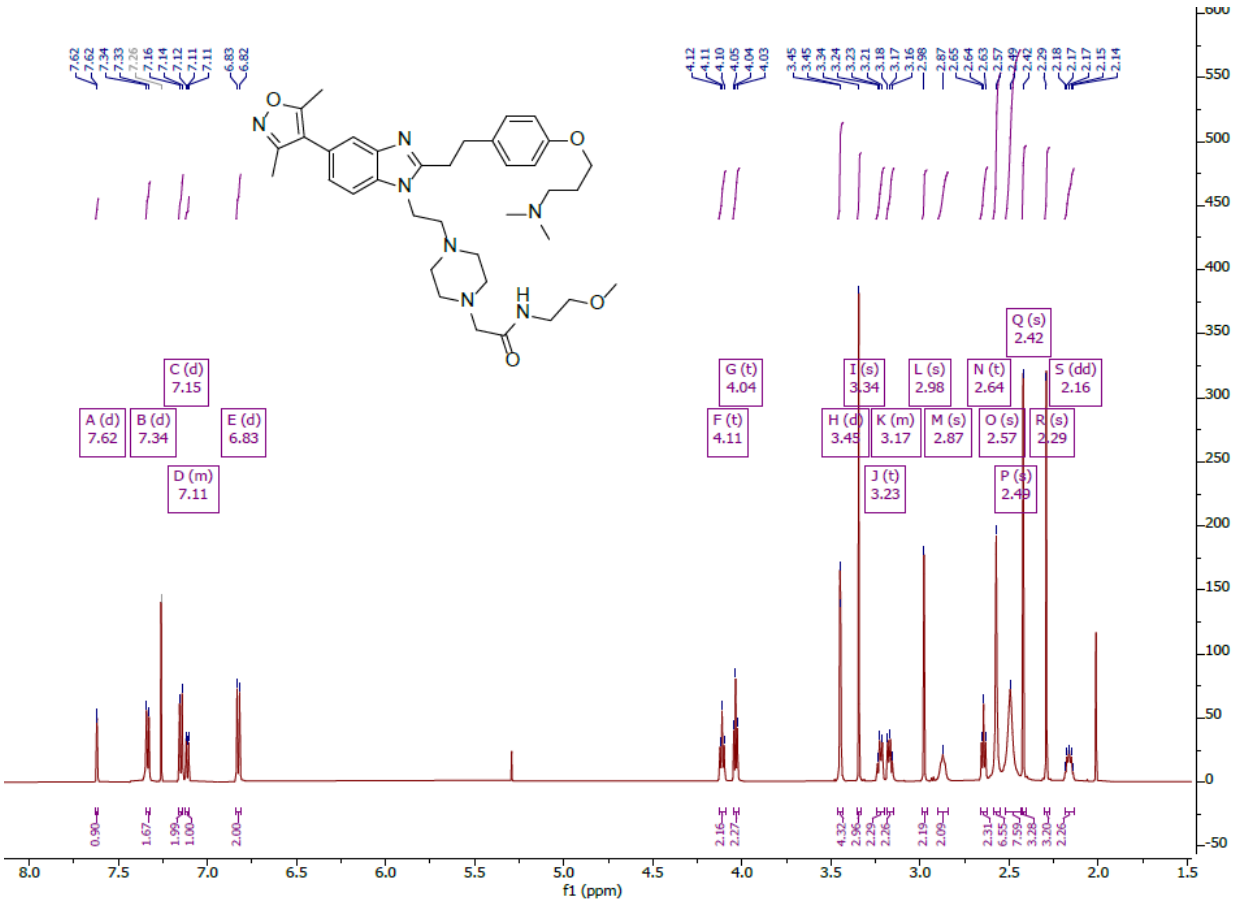

^{1}H NMR spectrum for **29**.

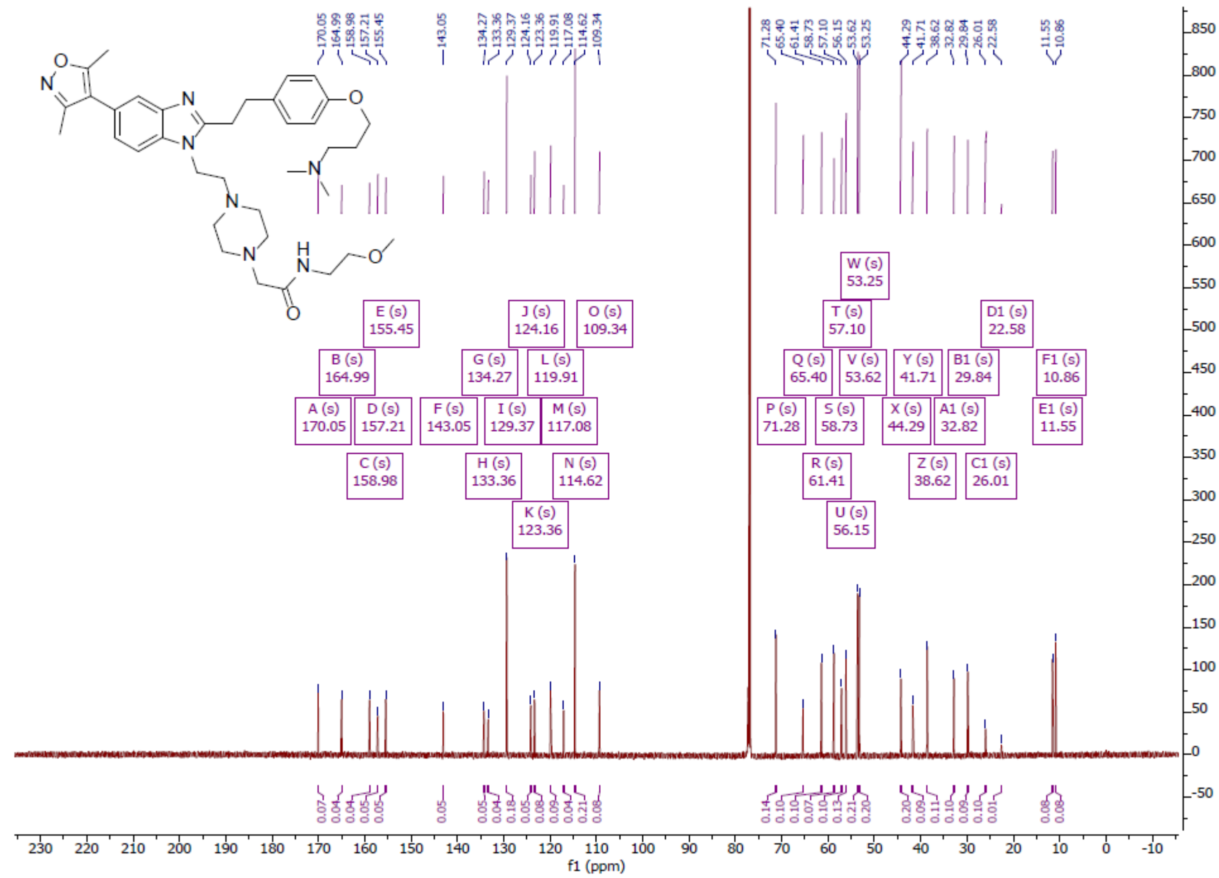

^{13}C NMR spectrum for **29**.

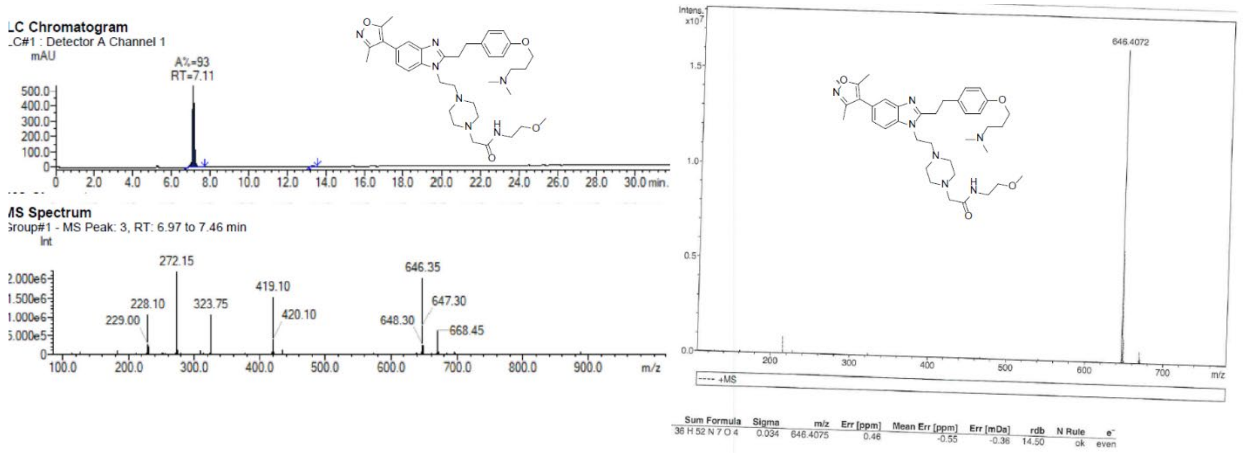

LC-MS/HMRS for **29**.

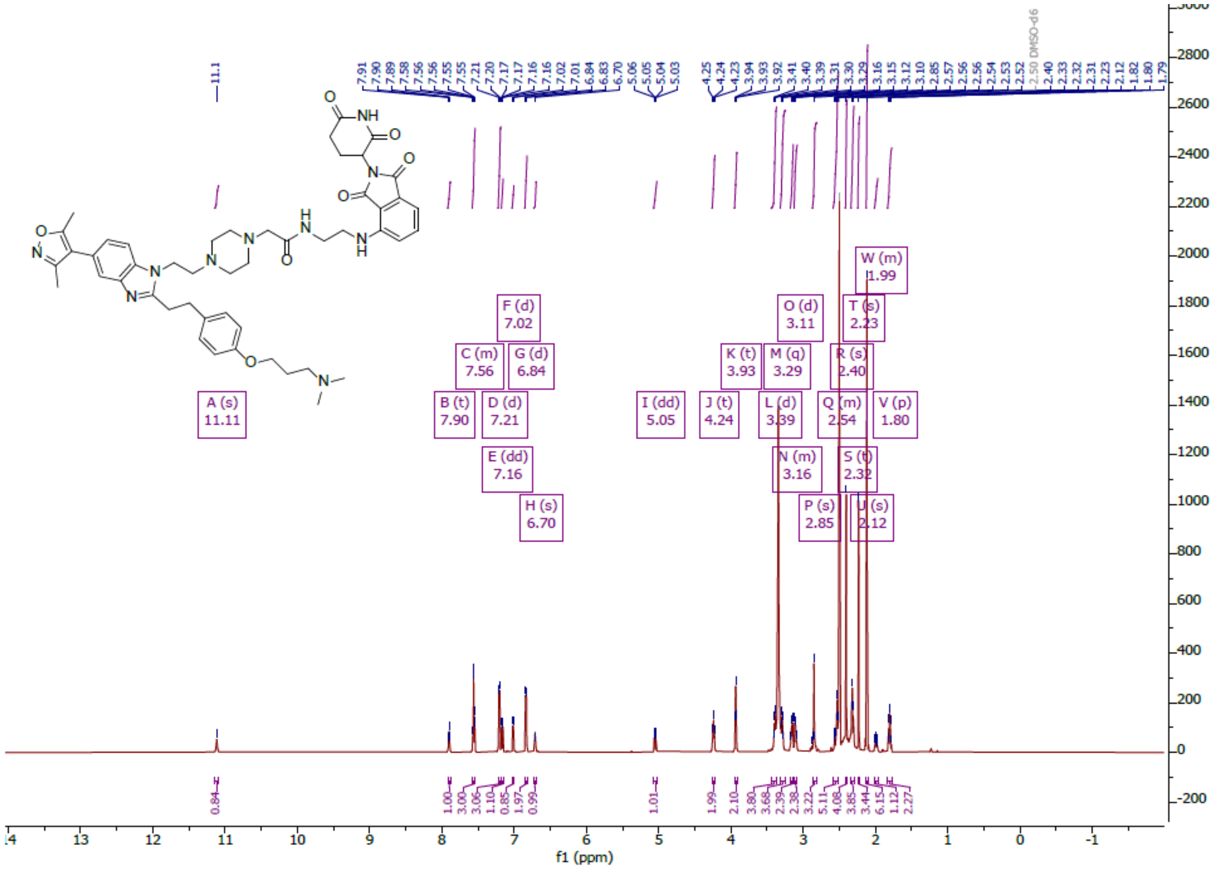

^{1}H NMR spectrum for **30**.

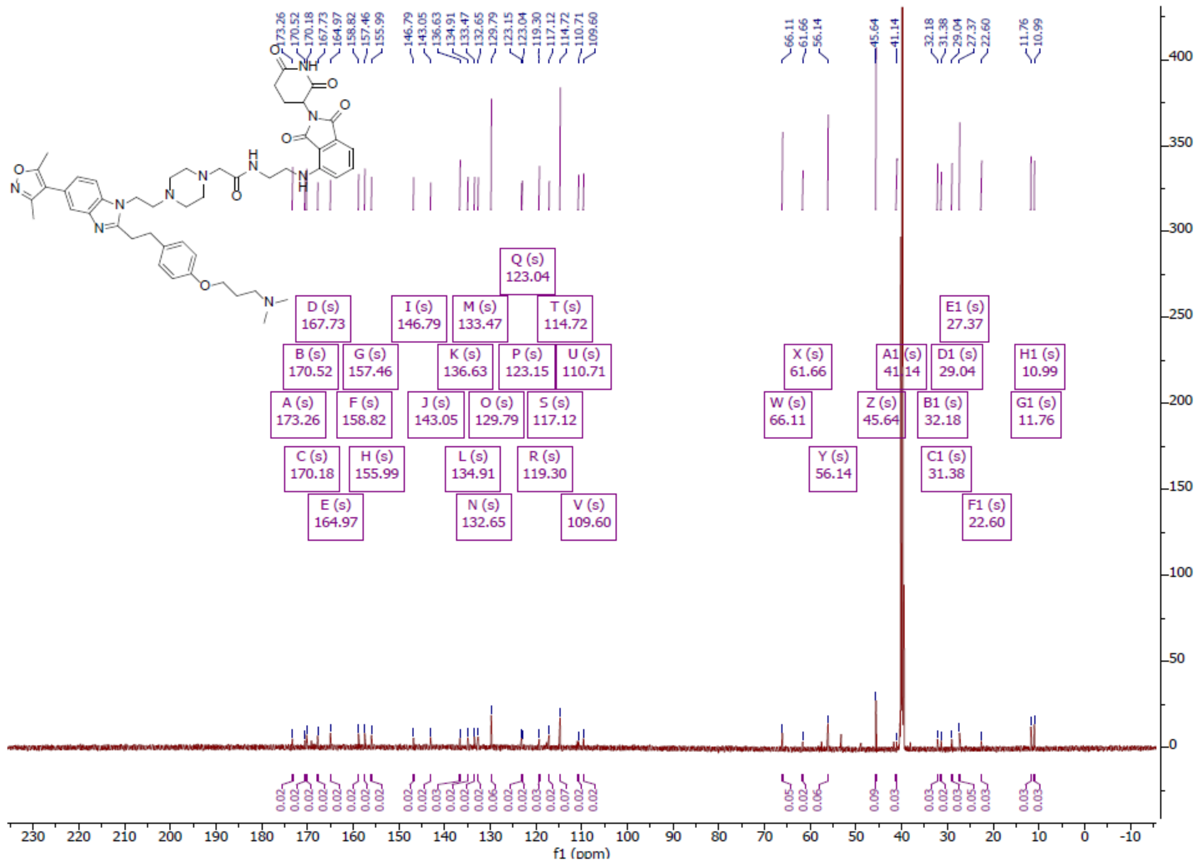

^{13}C NMR spectrum for **30**.

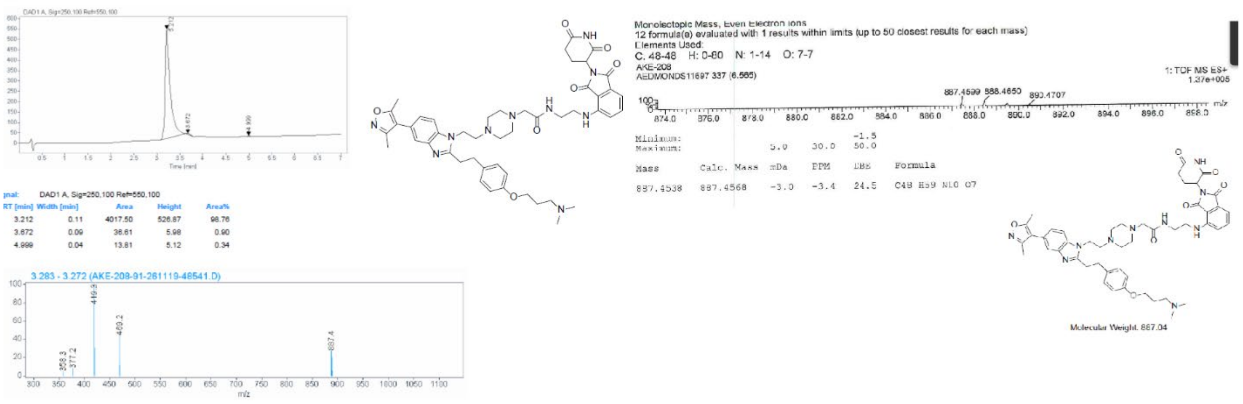

LC-MS/HRMS for **30**.

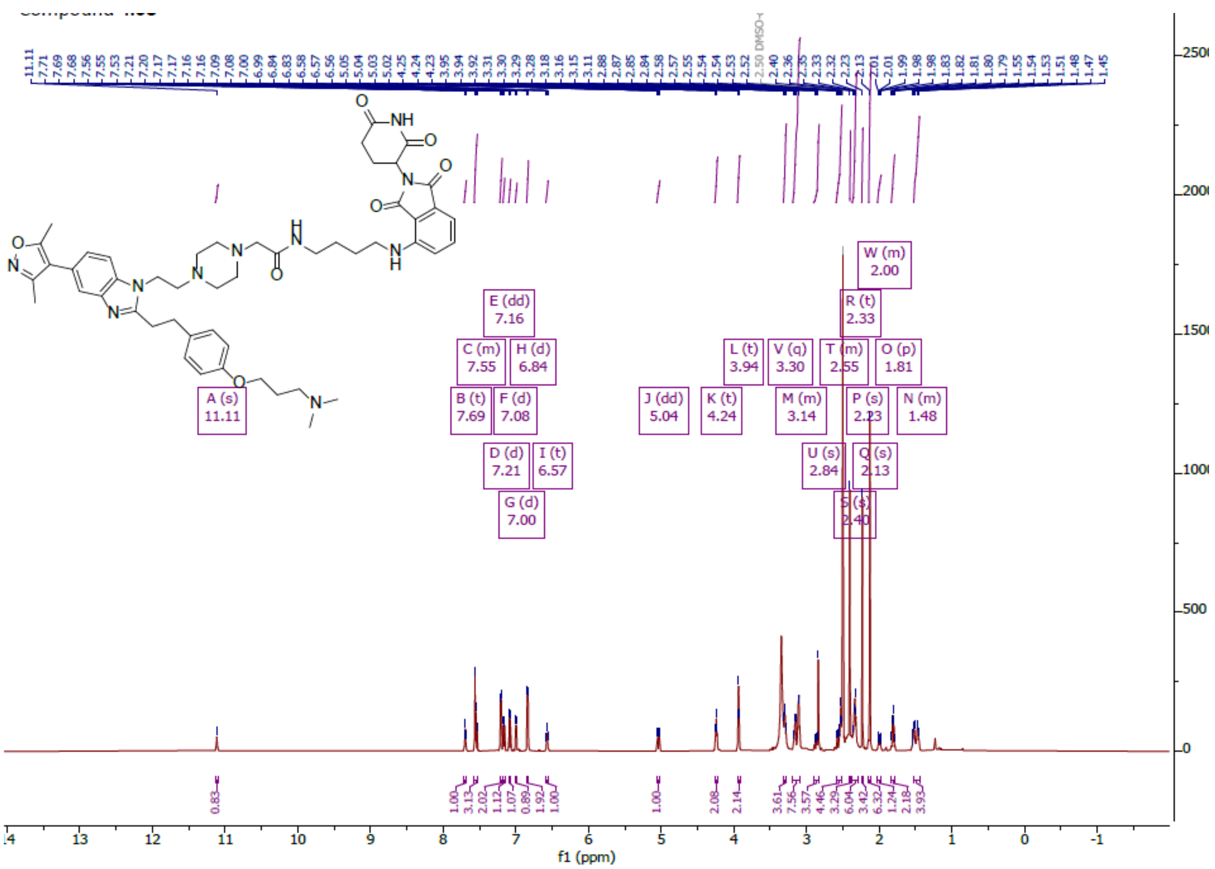

^{1}H NMR spectrum for **31**.

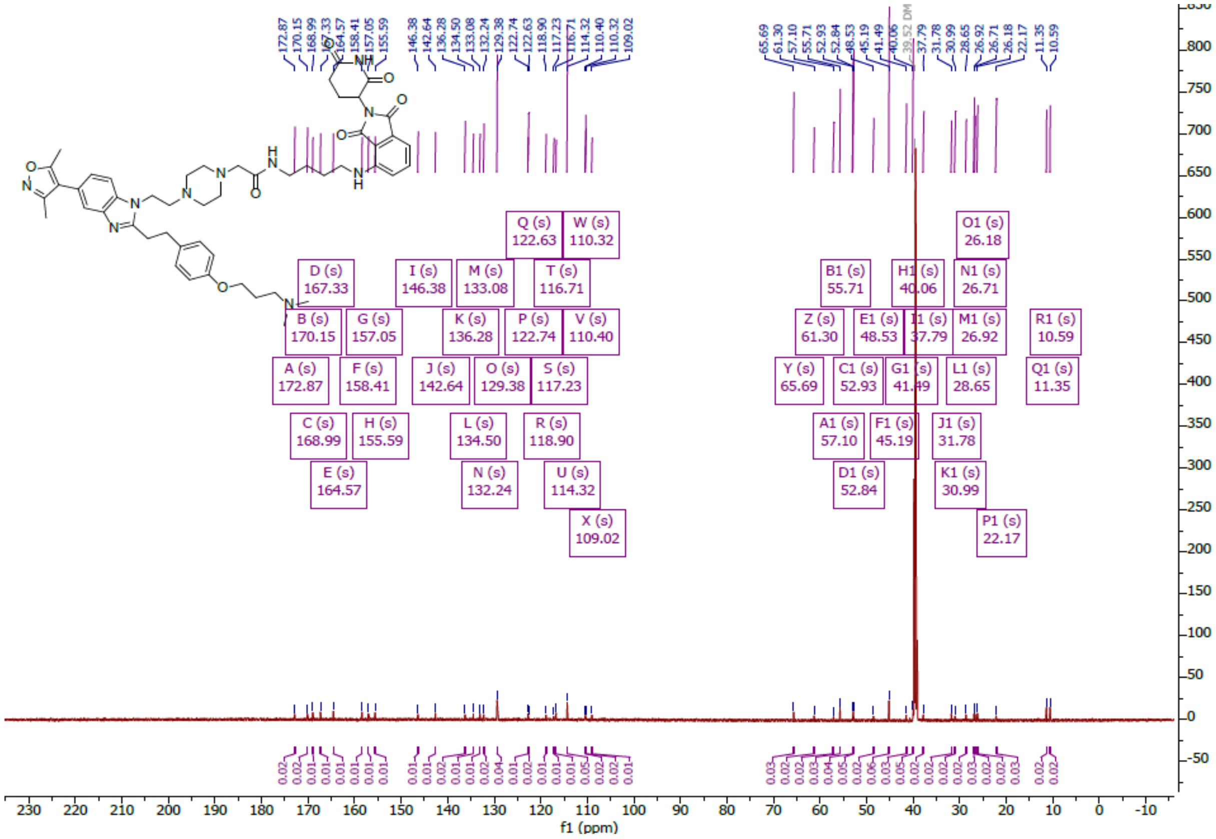

^{13}C spectrum for **31**.

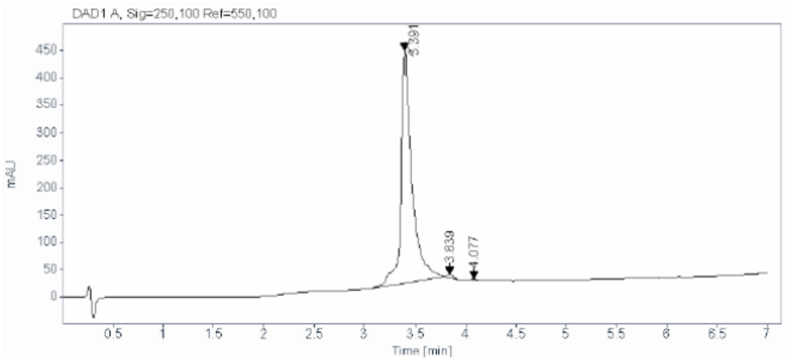

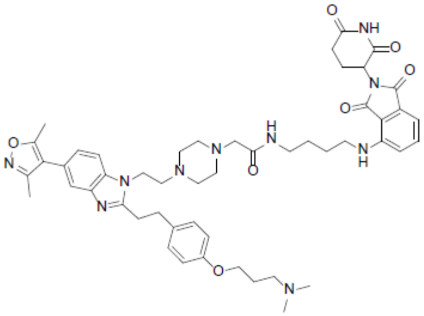

Signal: DAD1 A, Sig=250,100 Ref=550,100

RT [min]	Width [min]	Area	Height	Area%
3.391	0.11	3196.80	425.59	98.85
3.839	0.07	28.41	6.43	0.88
4.077	0.06	8.77	2.28	0.27

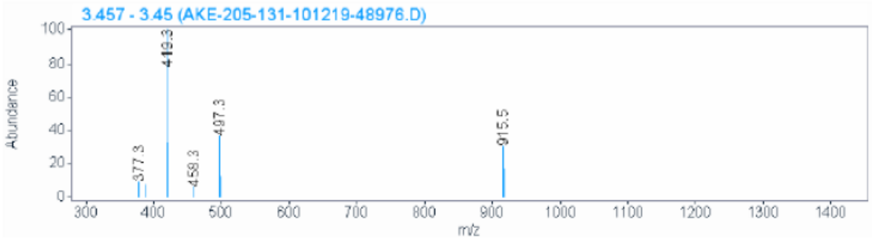

Monoisotopic Mass, Even Electron Ions
12 formula(e) evaluated with 1 results within limits (up to 50 closest results for each mass)
Elements Used:
C: 50-50 H: 0-80 N: 1-14 O: 7-7
AKE-205
AEDMONDS11698 341 (6.632)

1: TOF MS ES+
8.37e+004

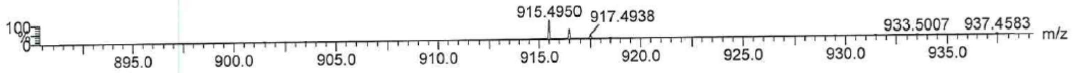

	Calc. Mass	mDa	PPM	DBE	Formula
Minimum:				-1.5	
Maximum:		5.0	30.0	50.0	
Mass					
915.4888	915.4881	0.7	0.8	24.5	C50 H63 N10 O7

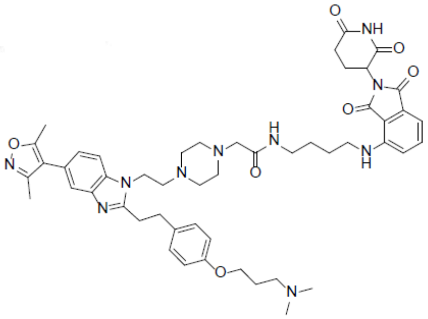

Molecular Weight: 915.09

LCMS/HRMS for **31**.

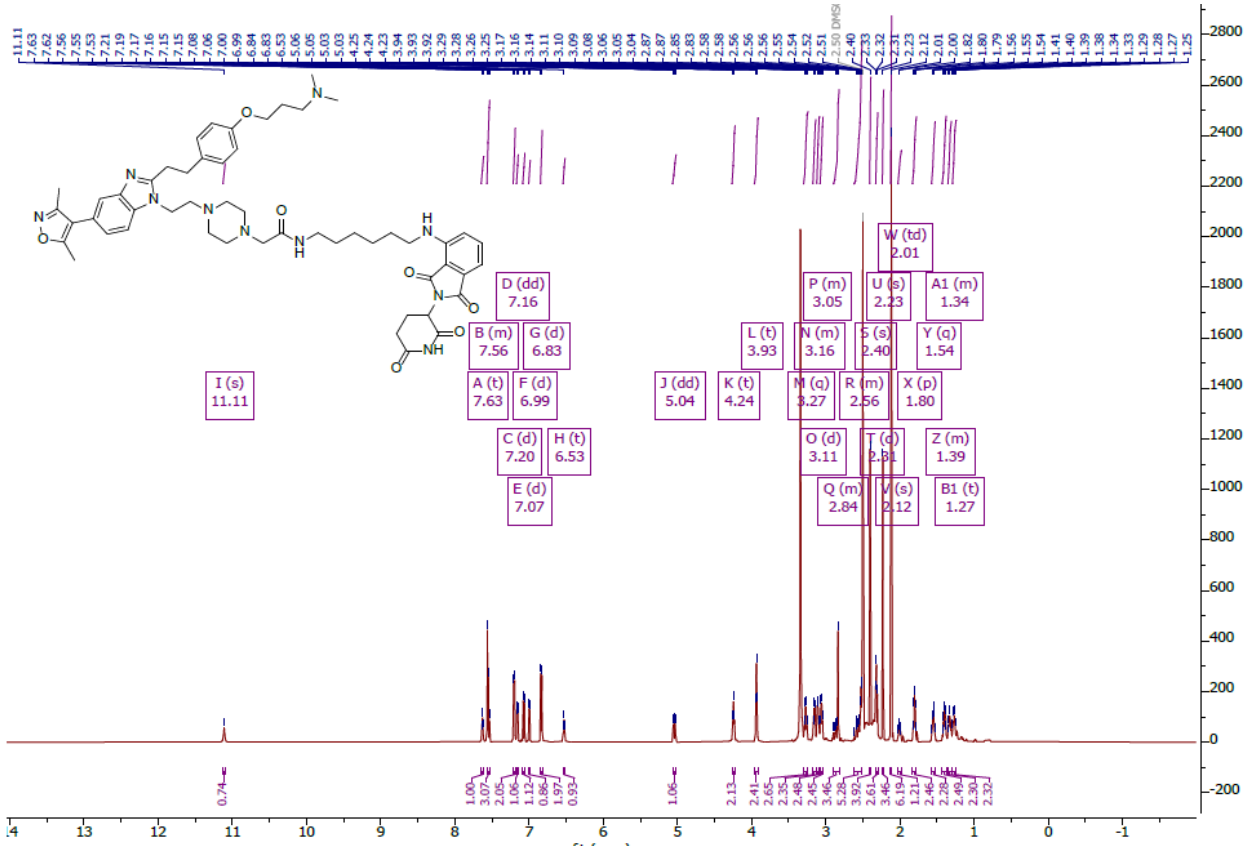

^{1}H NMR for **32**.

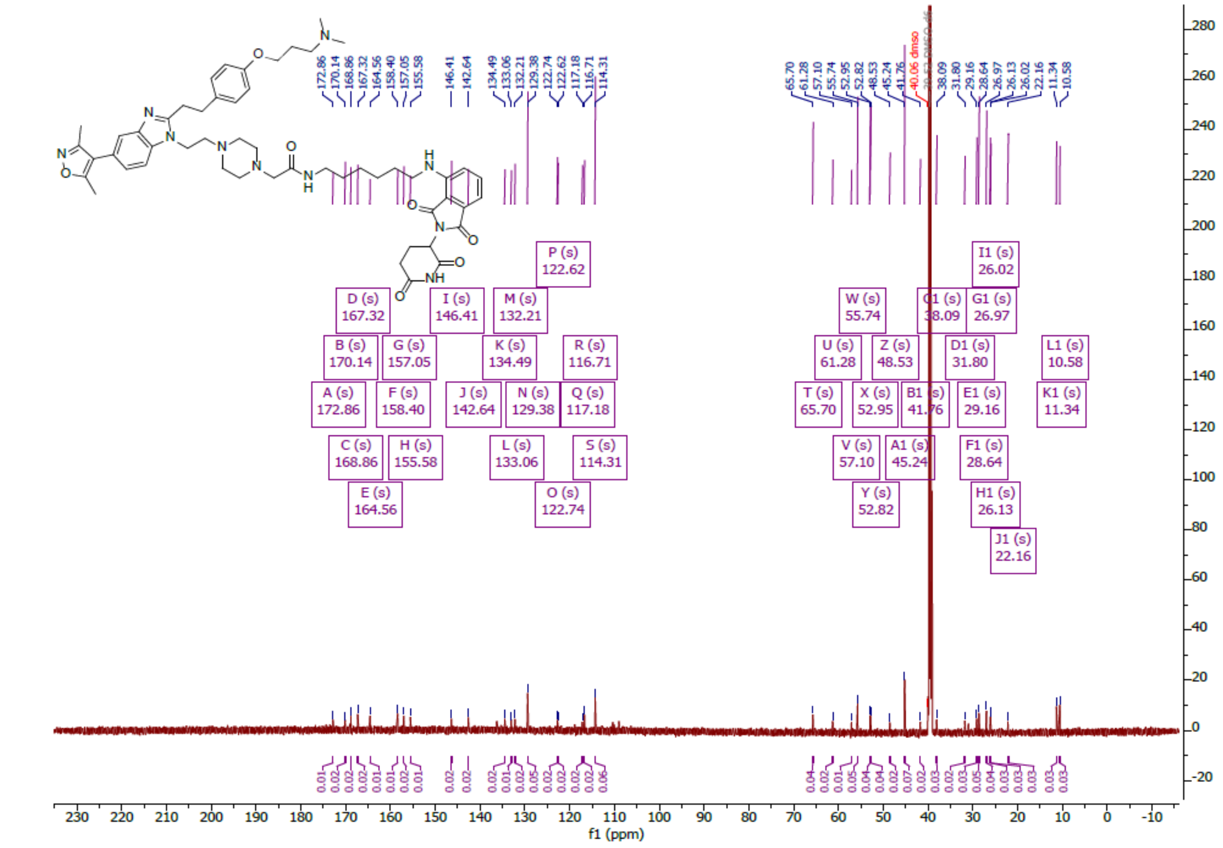

^{13}C NMR spectrum for **32**.

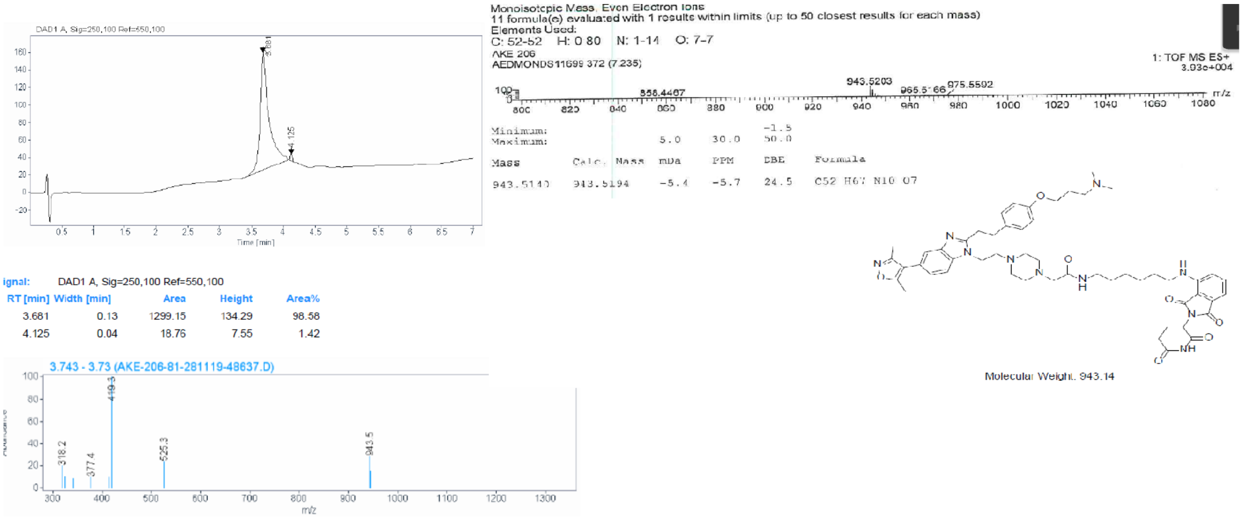

LCMS/HRMS for **32**.

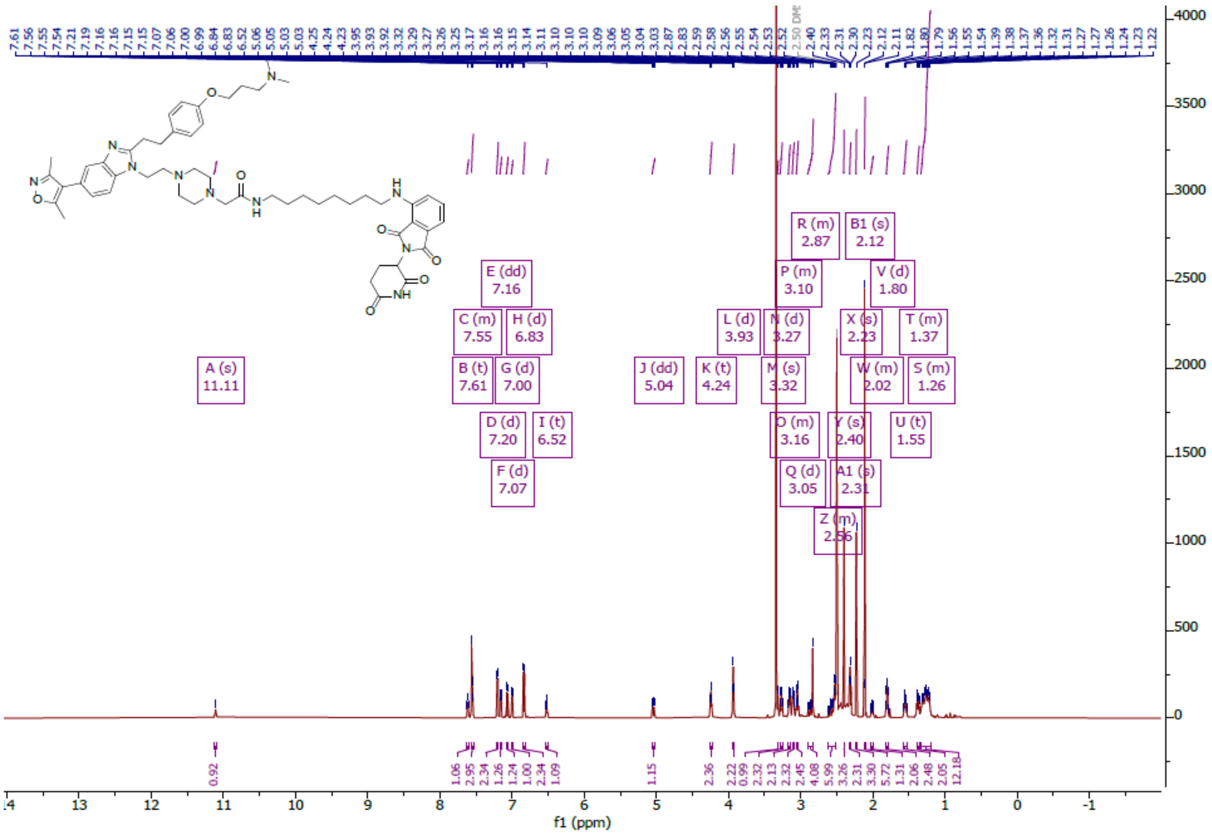

^{1}H NMR spectrum for **33**.

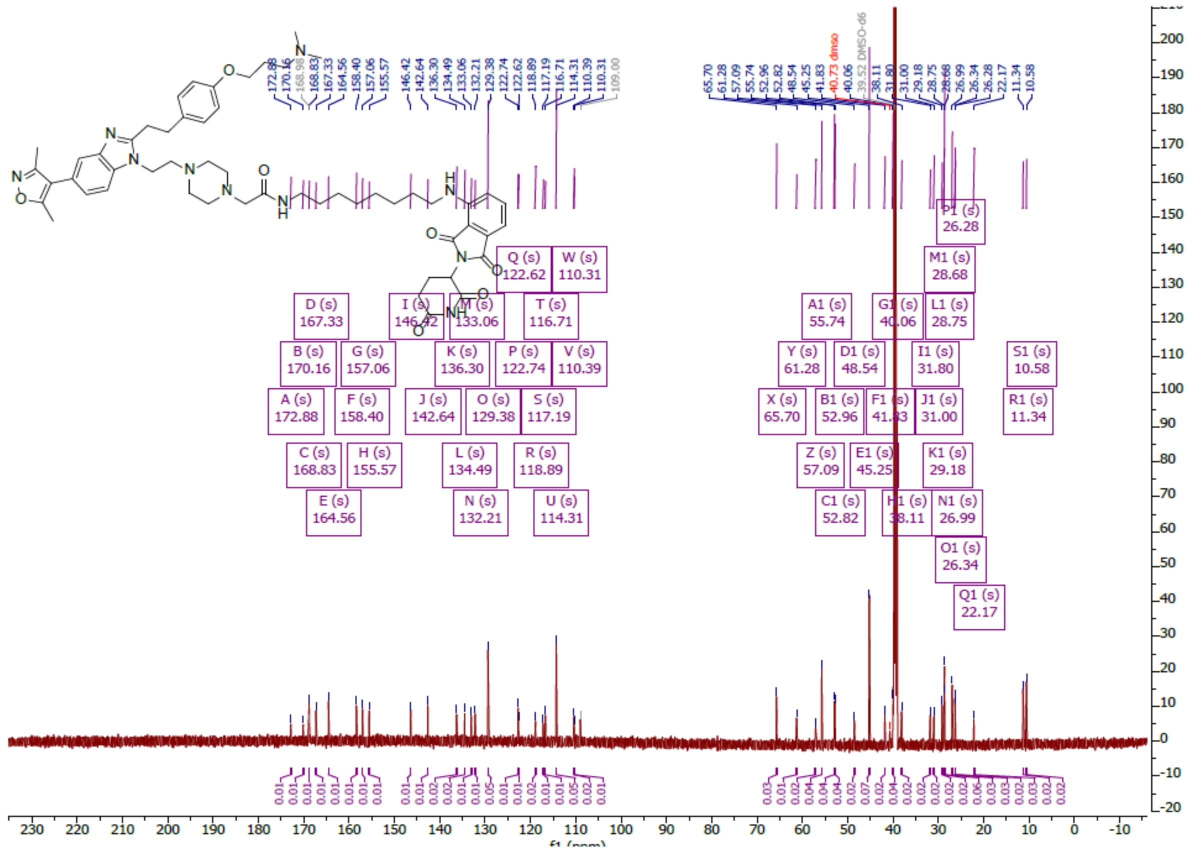

^{13}C NMR for **33**.

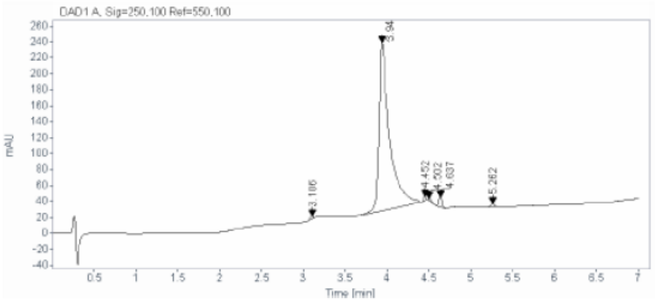

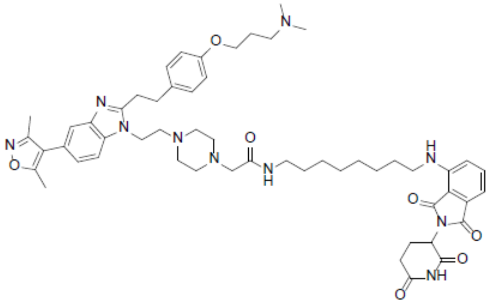

Signal: DAD1 A, Sig=250,100 Ref=550,100

RT [min]	Width [min]	Area	Height	Area%
3.106	0.04	7.55	2.82	0.39
3.940	0.12	1844.66	211.28	96.54
4.452	0.04	10.51	4.83	0.55
4.502	0.04	5.41	2.30	0.28
4.637	0.04	31.45	12.19	1.65
5.262	0.04	11.12	4.13	0.58

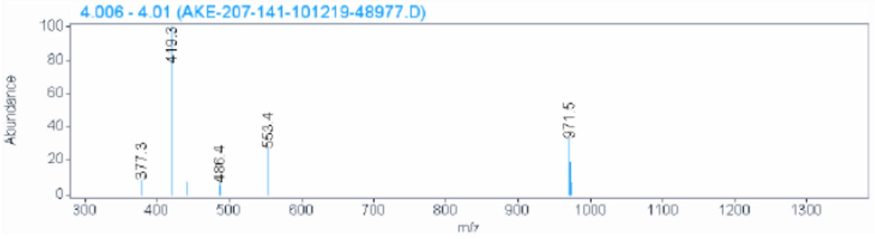

Monoisotopic Mass, Even Electron Ions
11 formula(e) evaluated with 1 results within limits (up to 50 closest results for each mass)
Elements Used:
C: 54-54 H: 0-80 N: 1-14 O: 7-7
AKE-207
AEDMONDS116100 326 (6.339) 1: TOF MS ES+ 3.65e+005

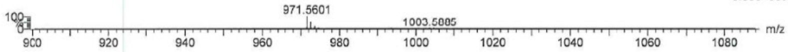

Minimum:				-1.5	
Maximum:		5.0	30.0	50.0	
Mass	Calc. Mass	mDa	PPM	DBE	Formula
971.5538	971.5507	3.1	3.2	24.5	C54 H71 N10 O7

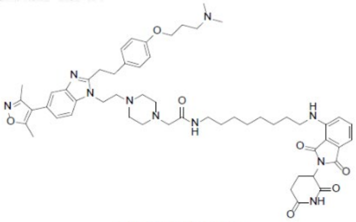

Molecular Weight: 971.20

LCMS/HRMS for **33**.

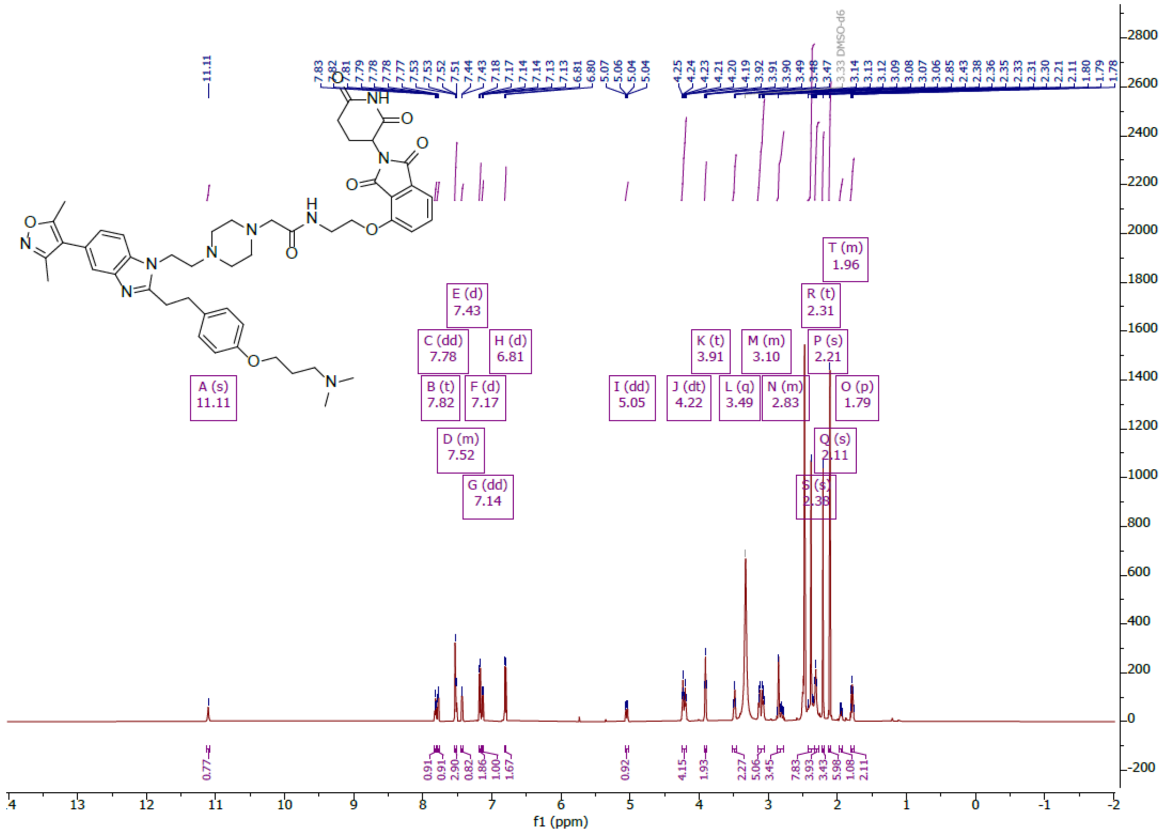

^{1}H NMR spectrum for **34**.

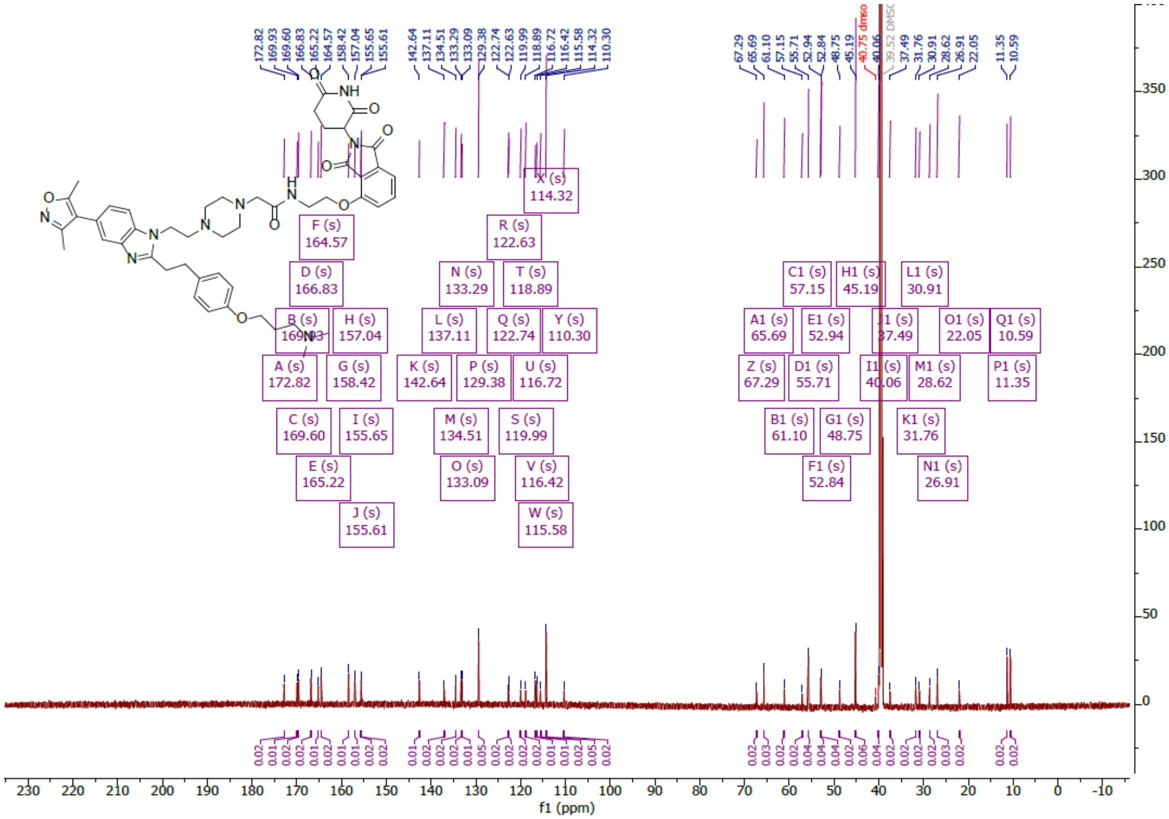

^{13}C NMR for **34**.

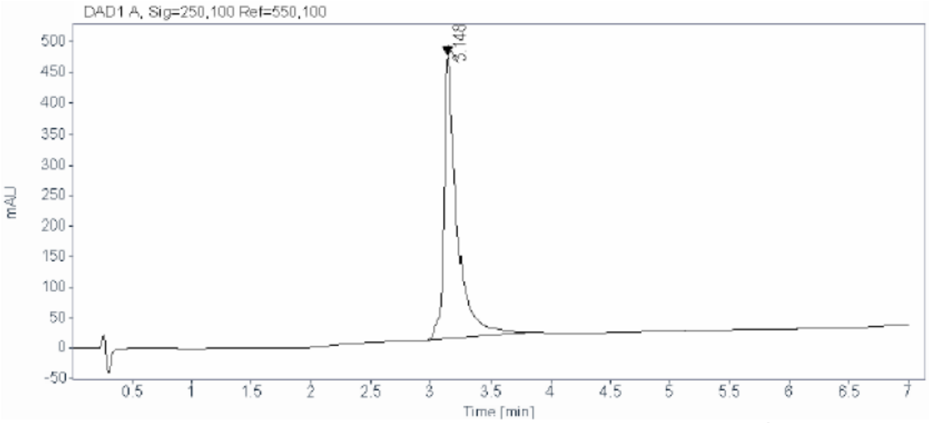

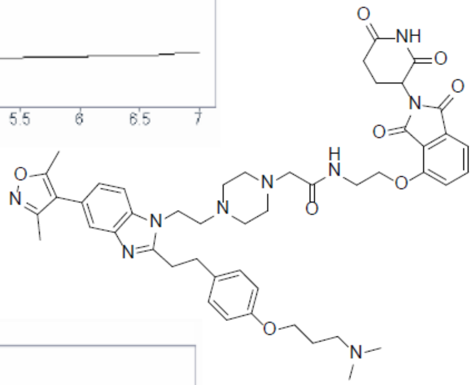

Signal: DAD1 A, Sig=250,100 Ref=550,100

RT [min]	Width [min]	Area	Height	Area%
3.148	0.11	3501.70	463.30	100.00

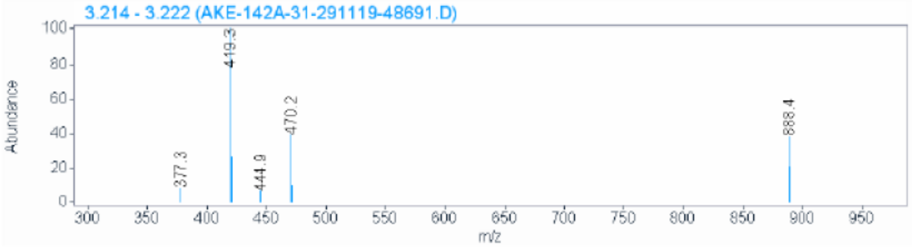

Monoisotopic Mass, Even Electron Ions
2 formula(e) evaluated with 1 results within limits (up to 50 closest results for each mass)
Elements Used:
C: 48-48 H: 0-80 N: 9-9 O: 8-8 Na: 0-1
AKE-142
AEDMONDS116105 331 (6.443)

1: TOF MS ES+
3.03e+004

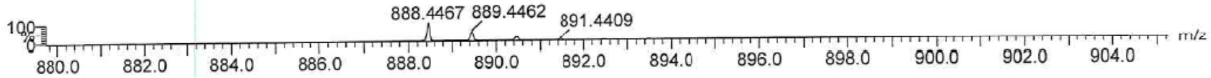

	Mass	Calc. Mass	mDa	PPM	DBE	Formula
Minimum:					-1.5	
Maximum:			5.0	30.0	50.0	
	888.4406	888.4408	-0.2	-0.2	24.5	C48 H58 N9 O8

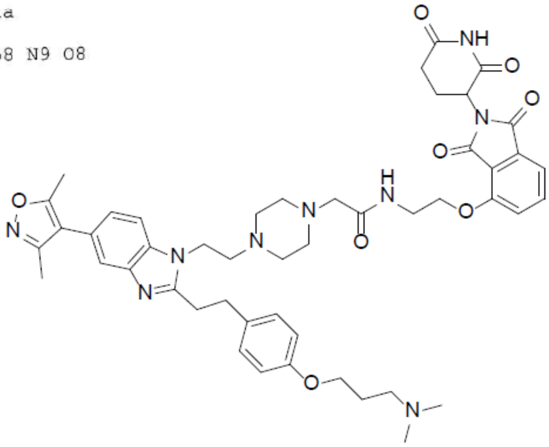

Molecular Weight: 888.02

LCMS/HRMS for **34**.

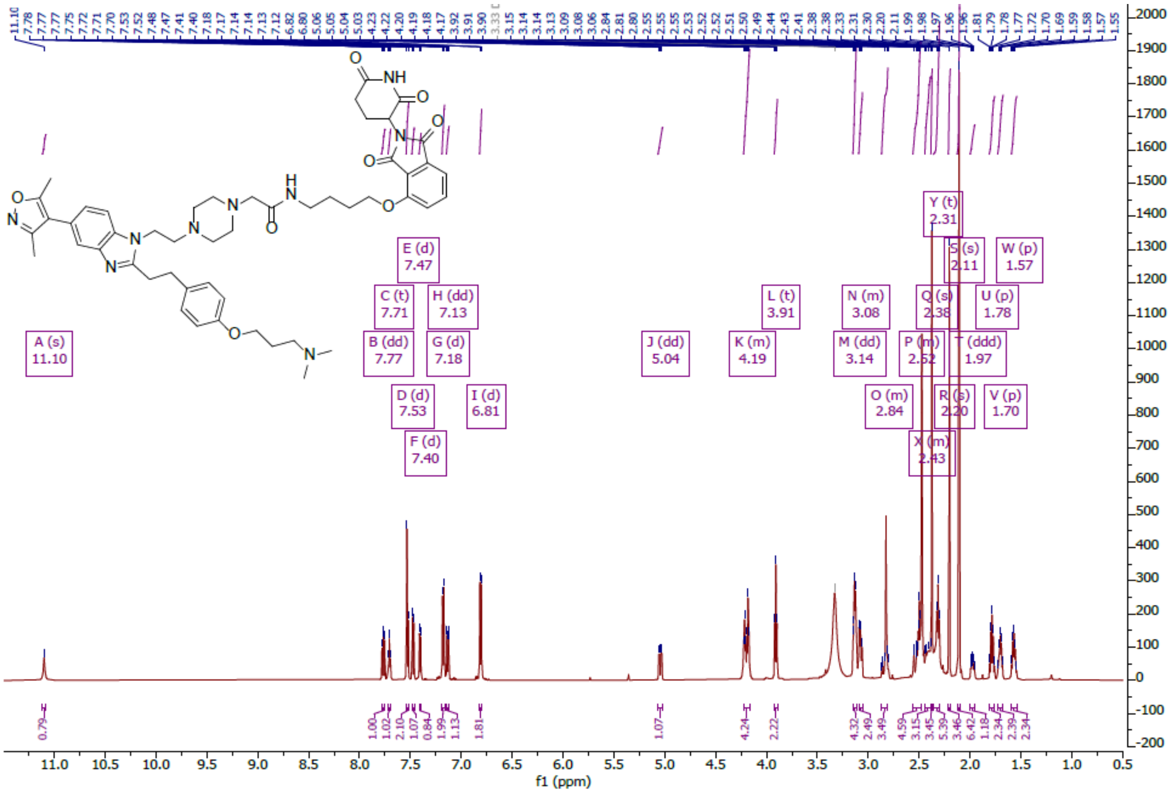

^{1}H NMR spectrum for **35**.

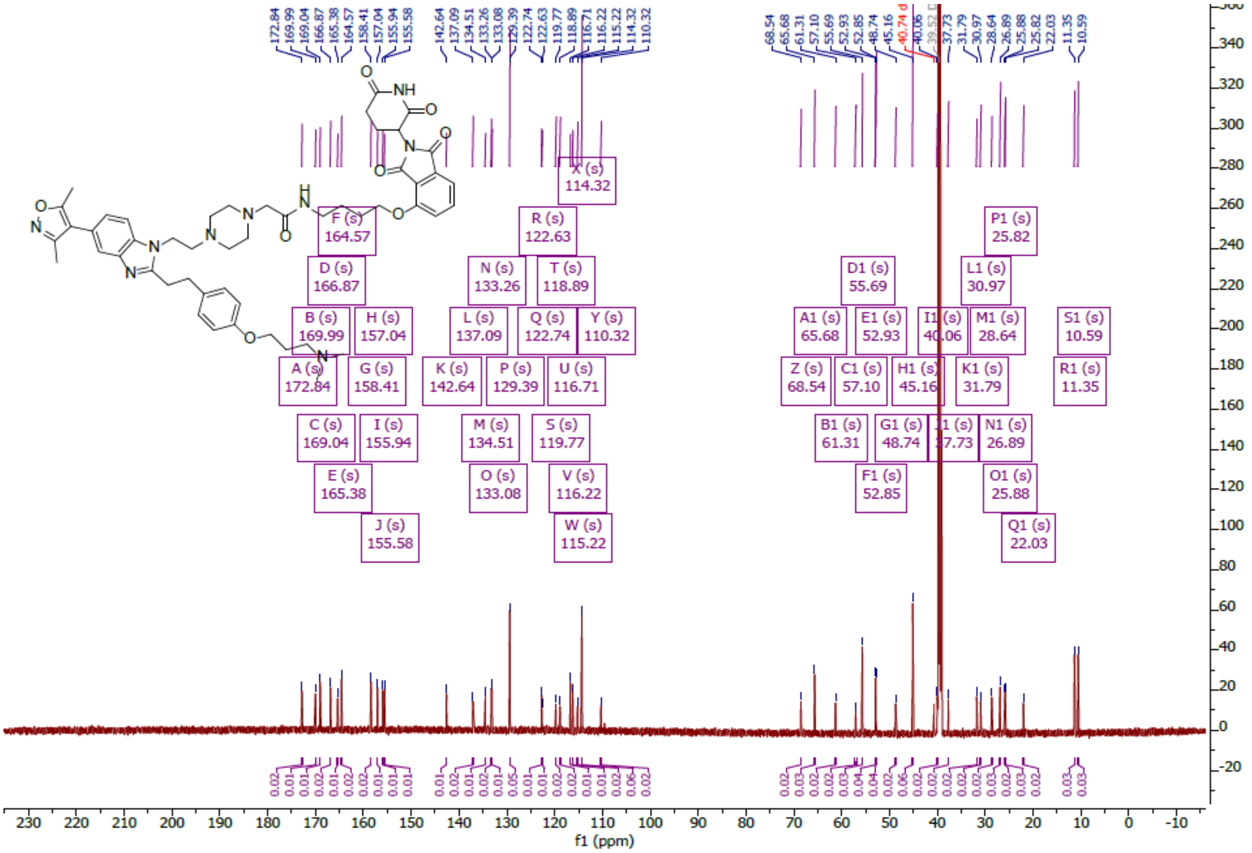

^{13}C NMR for **35**.

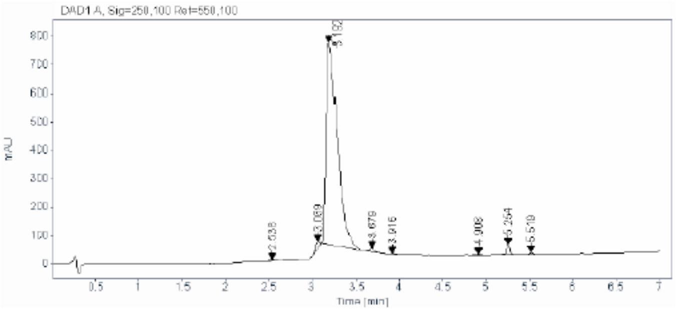

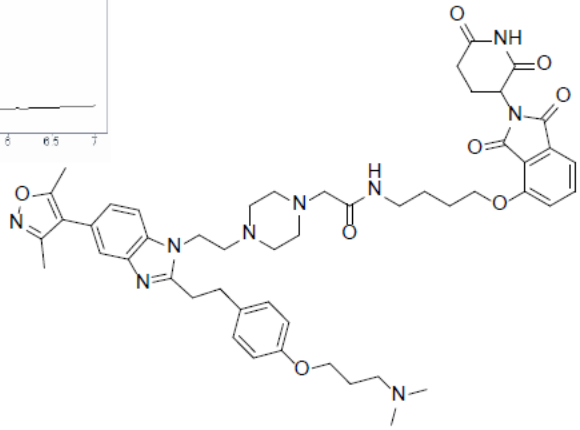

Signal: DAD1 A, Sig=250,100 Ref=550,100

RT [min]	Width [min]	Area	Height	Area%
2.538	0.05	6.89	2.23	0.11
3.069	0.05	72.19	22.88	1.14
3.192	0.14	6085.69	713.19	95.90
3.679	0.06	50.81	11.88	0.80
3.916	0.06	7.30	1.94	0.12
4.908	0.05	7.92	2.48	0.12
5.254	0.05	100.09	32.97	1.58
5.519	0.05	15.11	5.28	0.24

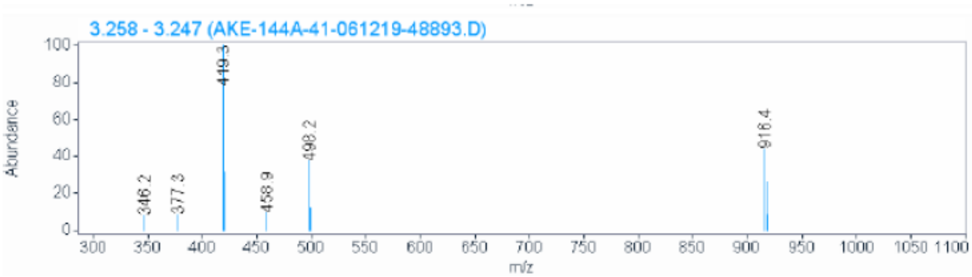

Monoisotopic Mass, Even Electron Ions
26 formula(e) evaluated with 1 results within limits (up to 50 closest results for each mass)
Elements Used:
C: 50-52 H: 0-80 N: 8-10 O: 8-10
AKE-144
AEDMONDS116106 274 (5.341)

1: TOF MS ES+
2.12e+004

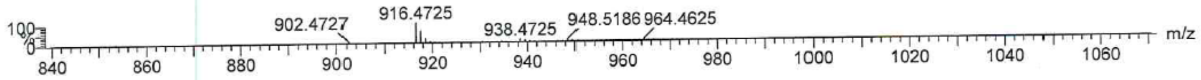

Minimum:				-1.5	
Maximum:		5.0	30.0	50.0	
Mass	Calc. Mass	mDa	PPM	DBE	Formula
916.4725	916.4721	0.4	0.4	24.5	C50 H62 N9 O8

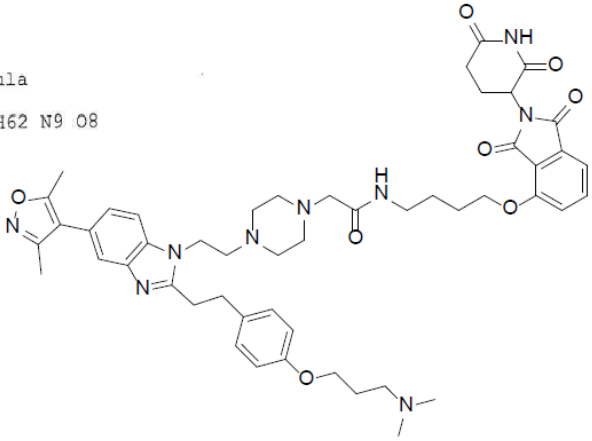

Molecular Weight: 916.07

LCMS/HRMS for **35**.

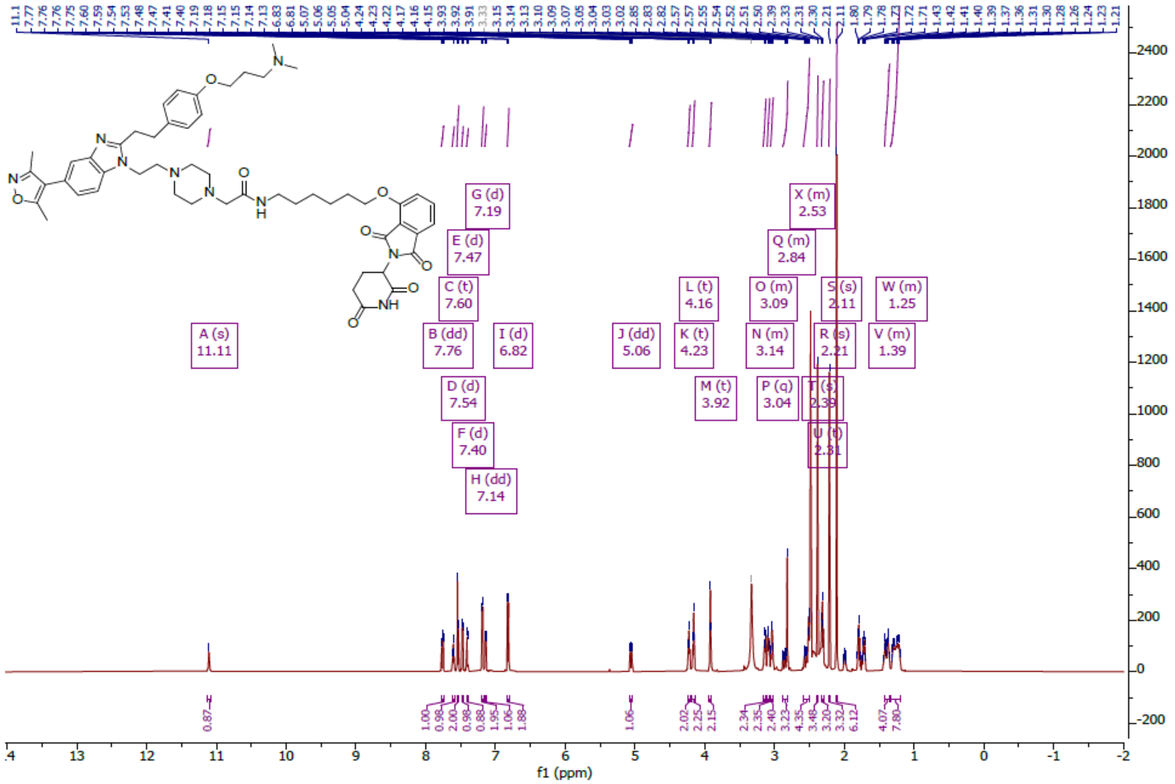

^{1}H NMR spectrum for **36**.

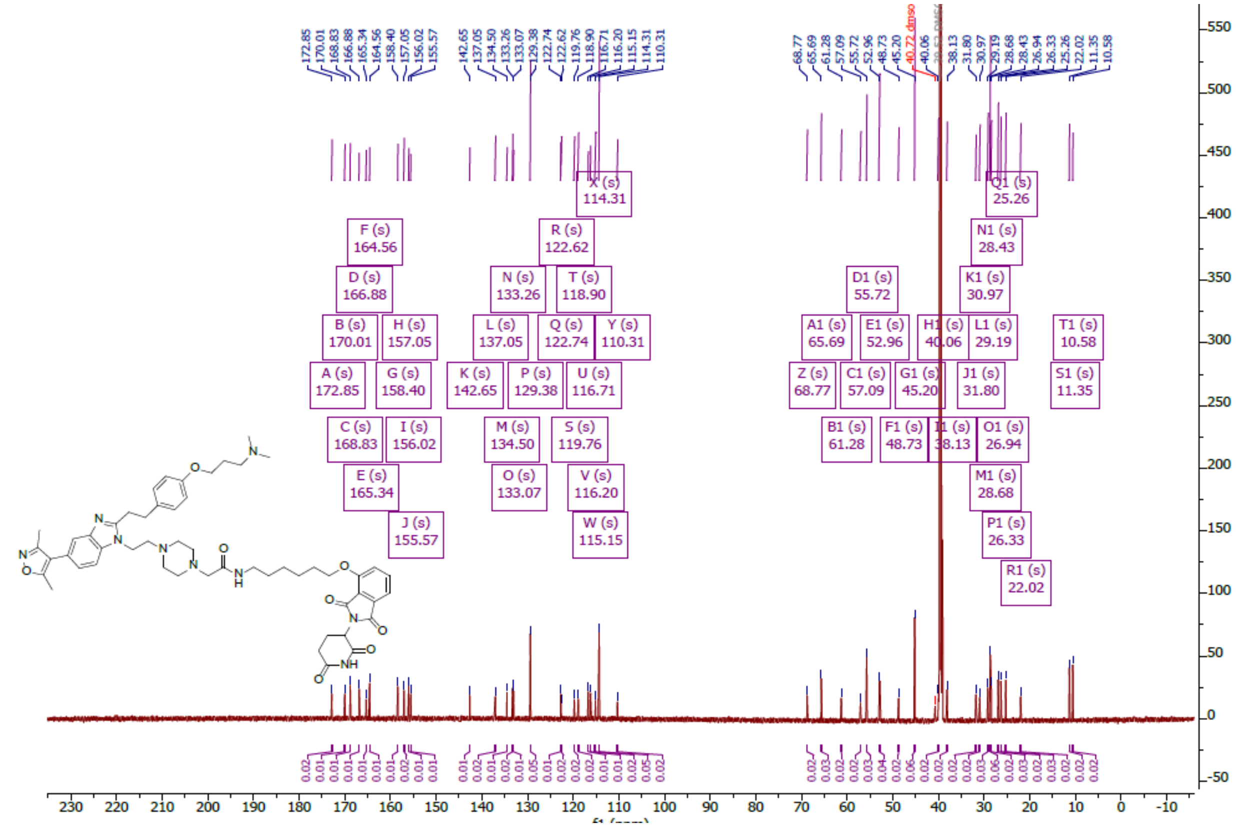

^{13}C NMR for **36**.

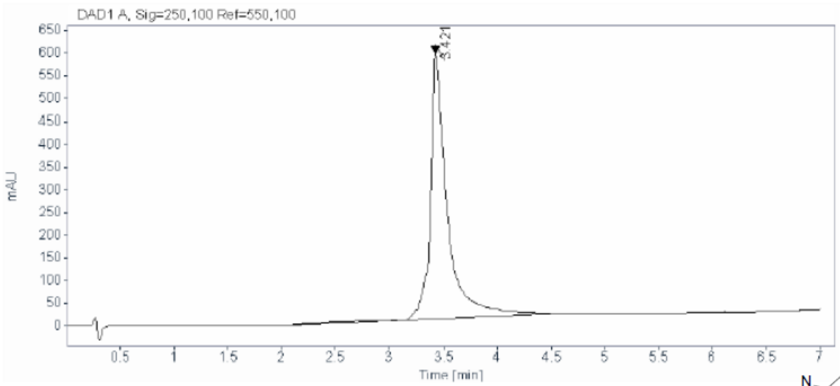

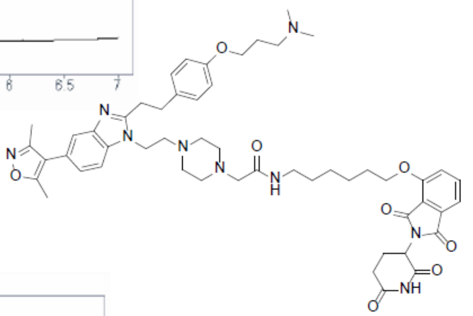

Signal: DAD1 A, Sig=250,100 Ref=550,100

RT [min]	Width [min]	Area	Height	Area%
3.421	0.16	6512.30	584.88	100.00

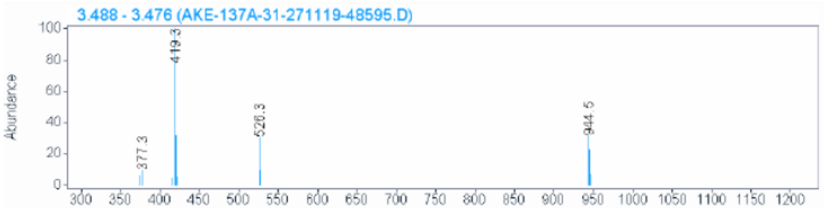

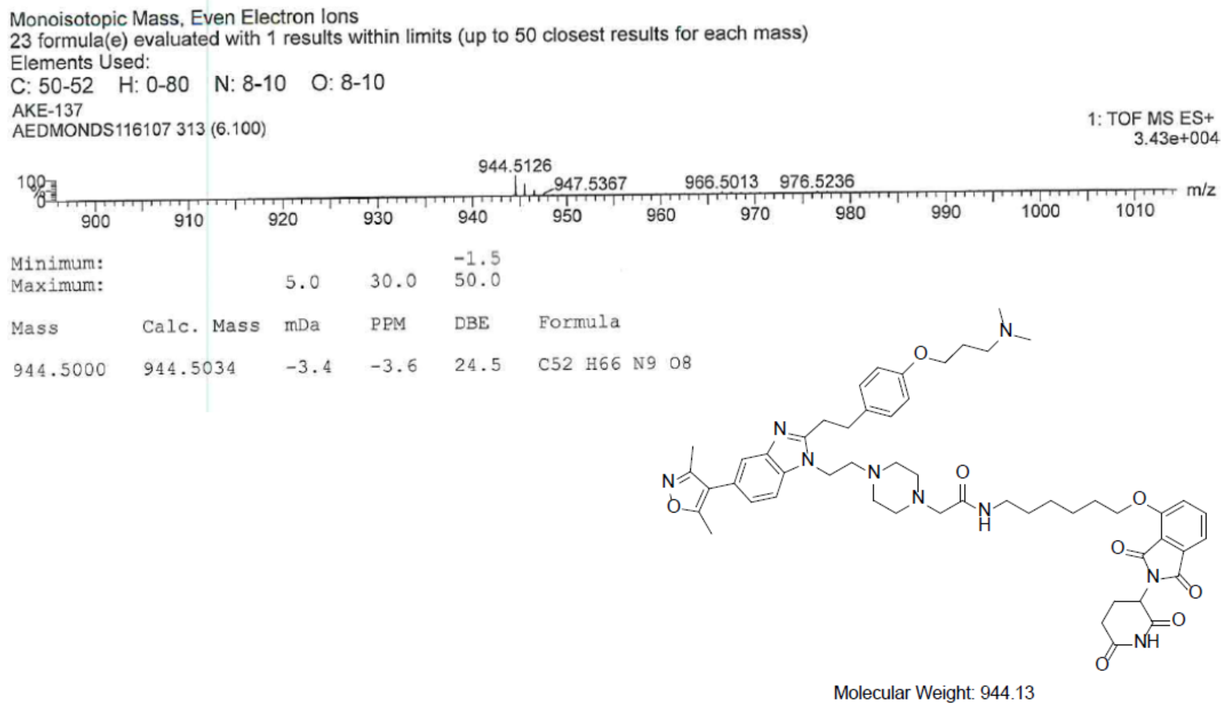

Monoisotopic Mass, Even Electron Ions
23 formula(e) evaluated with 1 results within limits (up to 50 closest results for each mass)
Elements Used:
C: 50-52 H: 0-80 N: 8-10 O: 8-10
AKE-137
AEDMONDS116107 313 (6.100)

1: TOF MS ES+
3.43e+004

Minimum:				-1.5	
Maximum:		5.0	30.0	50.0	
Mass	Calc. Mass	mDa	PPM	DBE	Formula
944.5000	944.5034	-3.4	-3.6	24.5	C52 H66 N9 O8

LCMS/HRMS for **36**.

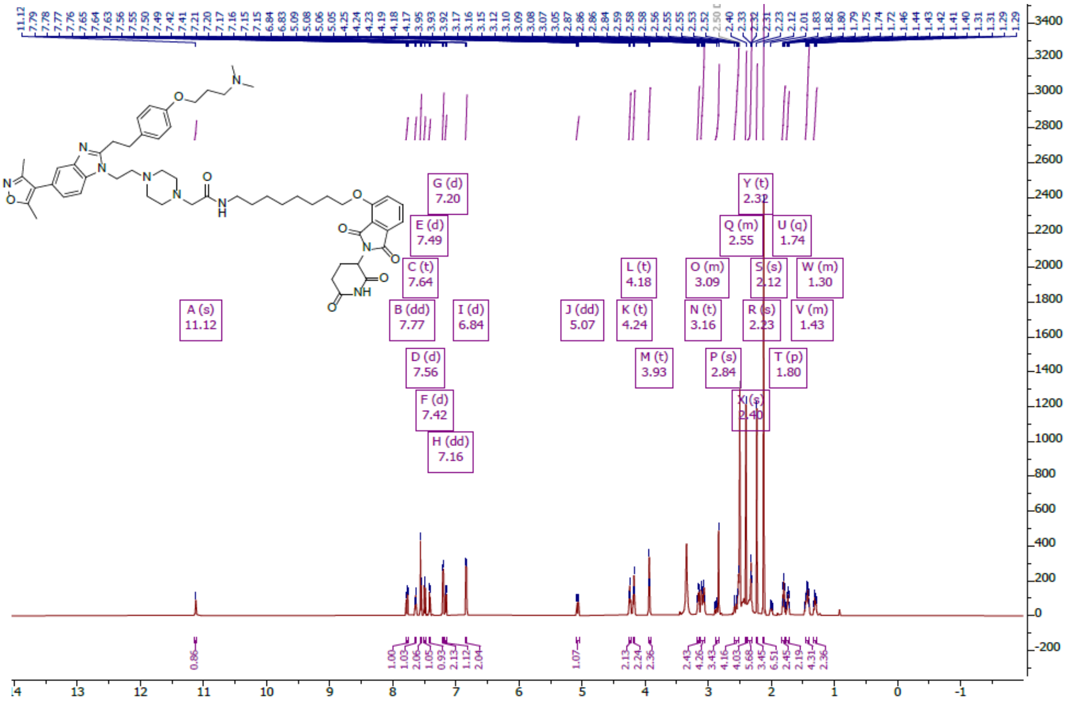

^{1}H NMR spectrum for **37**.

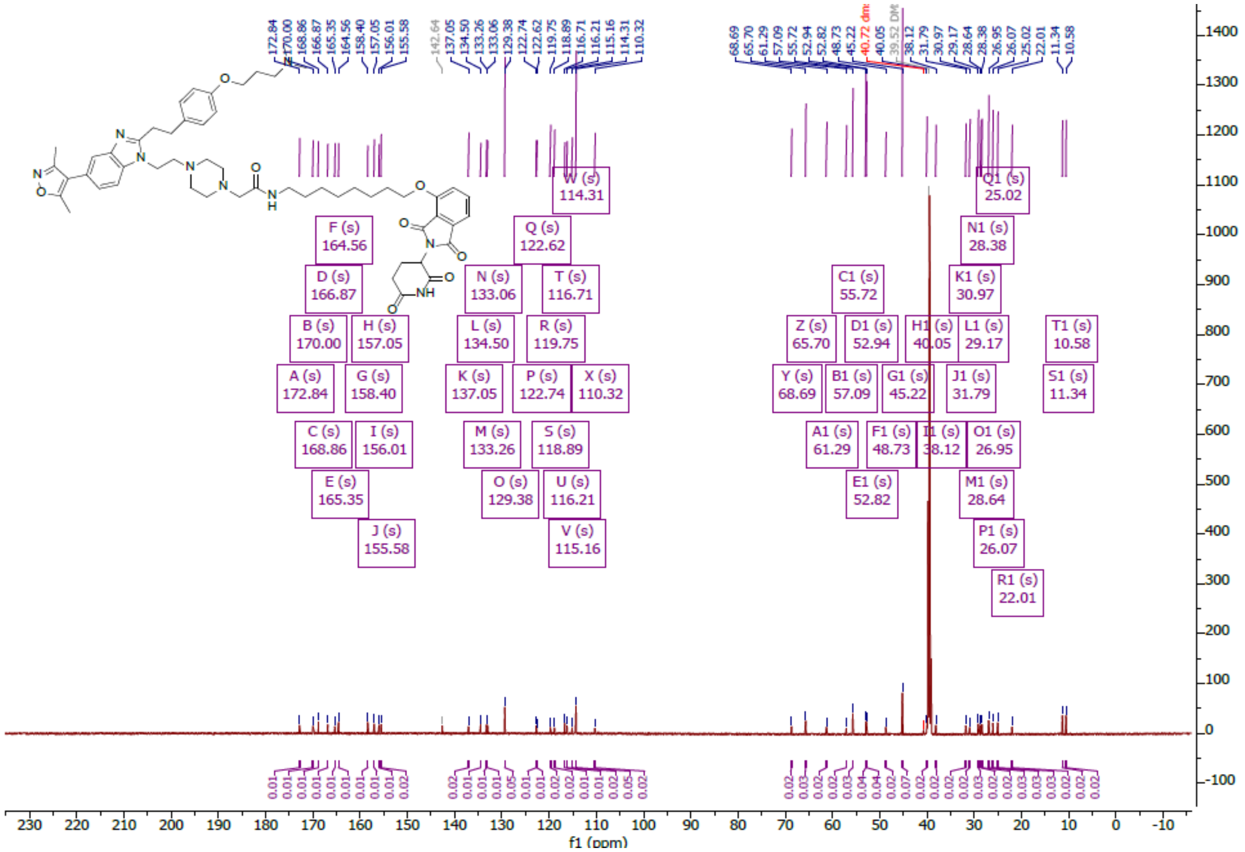

^{13}C NMR for **37**.

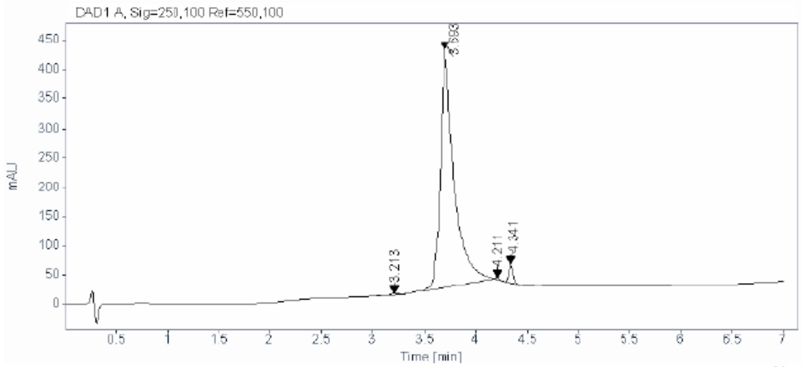

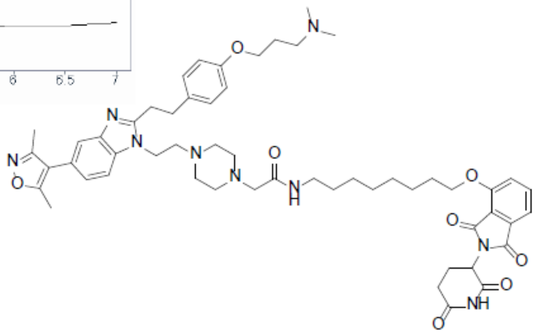

Signal: DAD1 A, Sig=250,100 Ref=550,100

RT [min]	Width [min]	Area	Height	Area%
3.213	0.09	14.92	2.41	0.37
3.693	0.13	3897.08	405.87	97.17
4.211	0.04	7.81	3.08	0.19
4.341	0.05	90.66	31.87	2.26

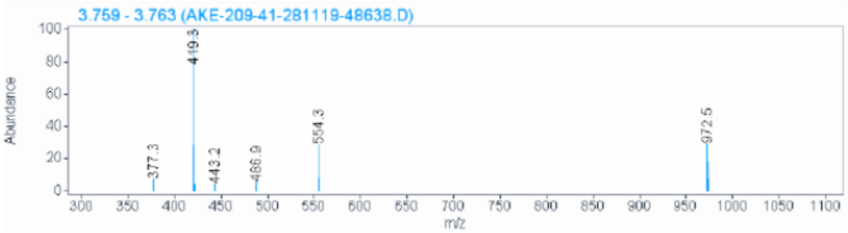

Monoisotopic Mass, Even Electron Ions
8 formula(e) evaluated with 1 results within limits (up to 50 closest results for each mass)
Elements Used:
C: 54-54 H: 0-80 N: 8-10 O: 8-10
AKE-209
AEDMONDS116108 344 (6.683)

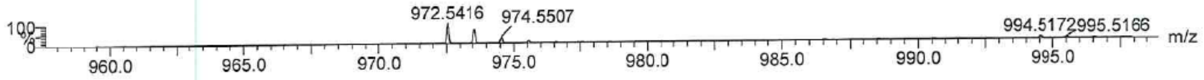

Minimum:				-1.5	
Maximum:		5.0	30.0	50.0	
Mass	Calc. Mass	mDa	PPM	DBE	Formula
972.5352	972.5347	0.5	0.5	24.5	C54 H70 N9 O8

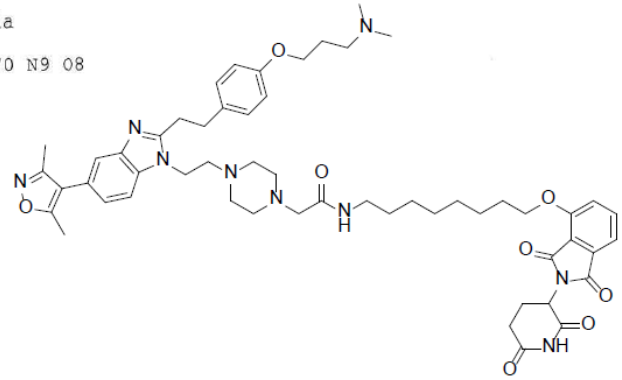

Molecular Weight: 972.18

LCMS/HRMS for **37**.

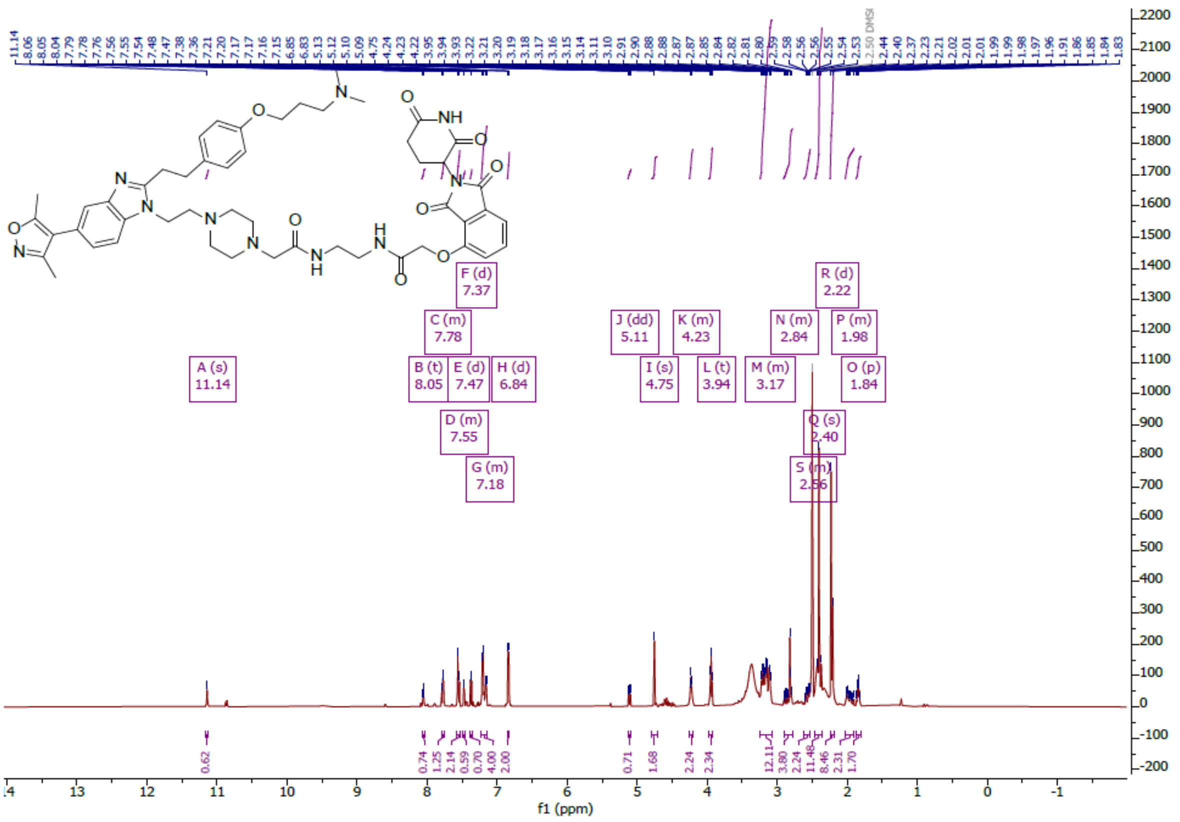

^{1}H NMR spectrum for **38**.

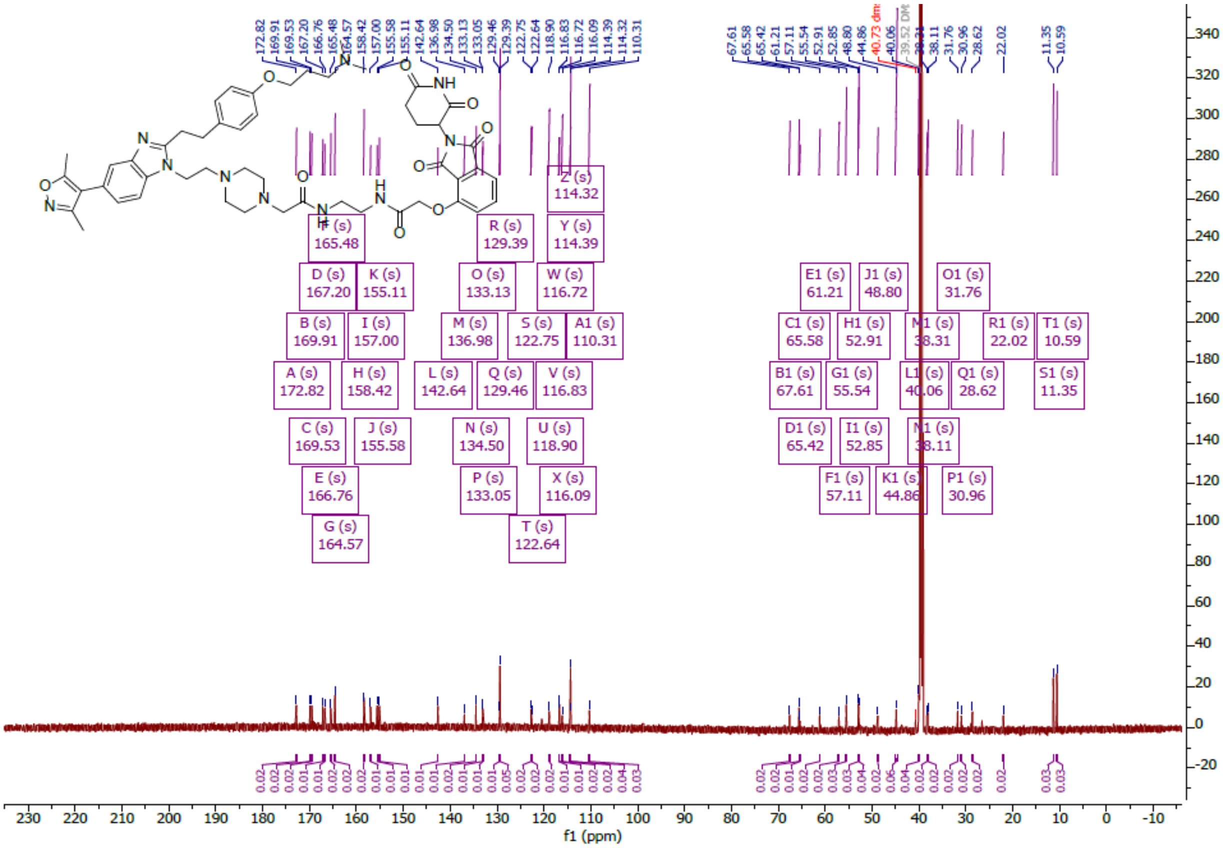

^{13}C NMR for **38**.

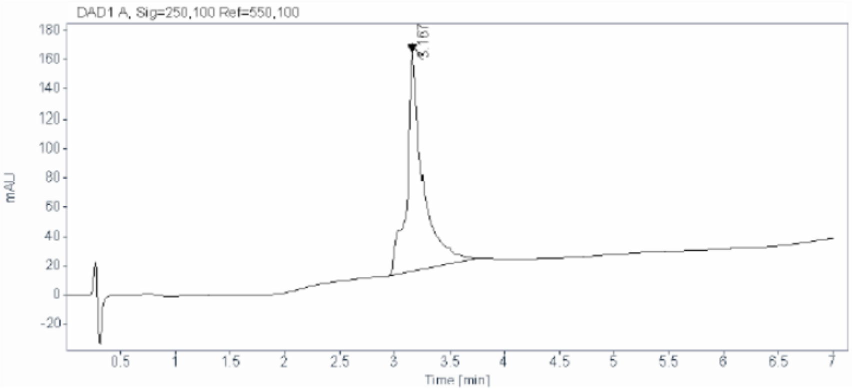

Signal: DAD1 A, Sig=250,100 Ref=550,100

RT [min]	Width [min]	Area	Height	Area%
3.167	0.13	1495.19	149.00	100.00

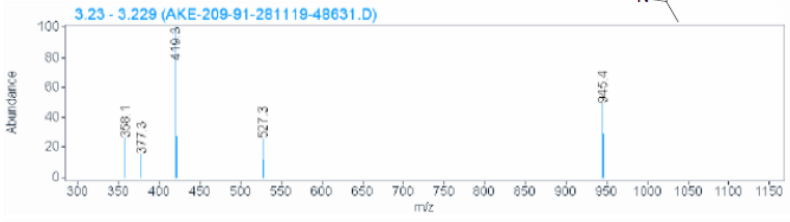

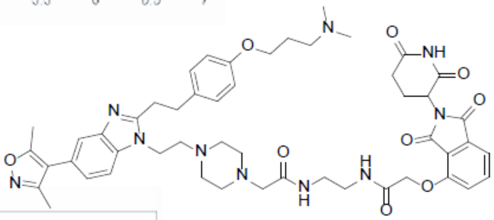

Monoisotopic Mass, Even Electron Ions
6 formula(e) evaluated with 1 results within limits (up to 50 closest results for each mass)
Elements Used:
C: 50-50 H: 0-80 N: 8-10 O: 8-10
AEK-210
AEDMONDS116123 304 (5.908)

1: TOF MS ES+
1.74e+004

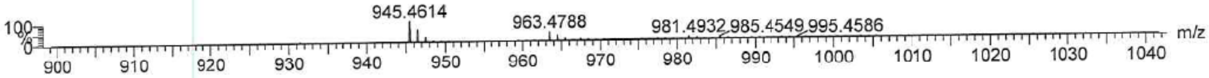

Minimum:				-1.5	
Maximum:		5.0	30.0	50.0	
Mass	Calc. Mass	mDa	PPM	DBE	Formula
945.4614	945.4623	-0.9	-1.0	25.5	C50 H61 N10 O9

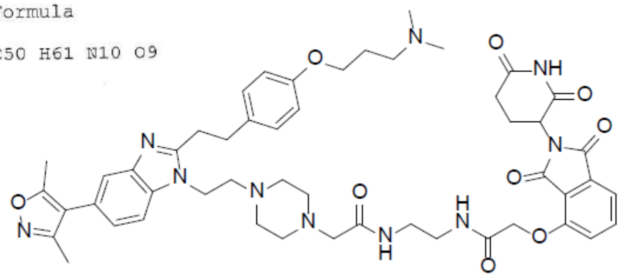

Molecular Weight: 945.07

LCMS/HRMS for **38**.

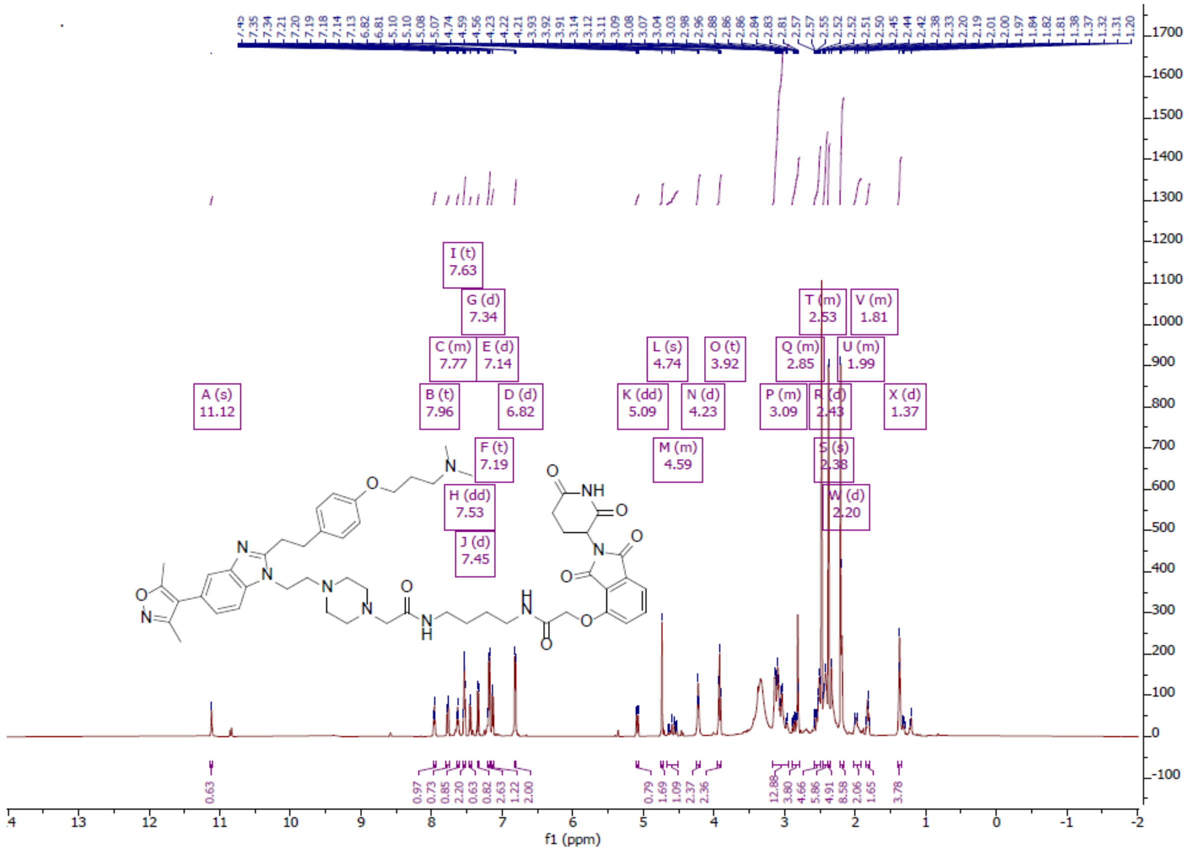

^{1}H NMR spectrum for **39**.

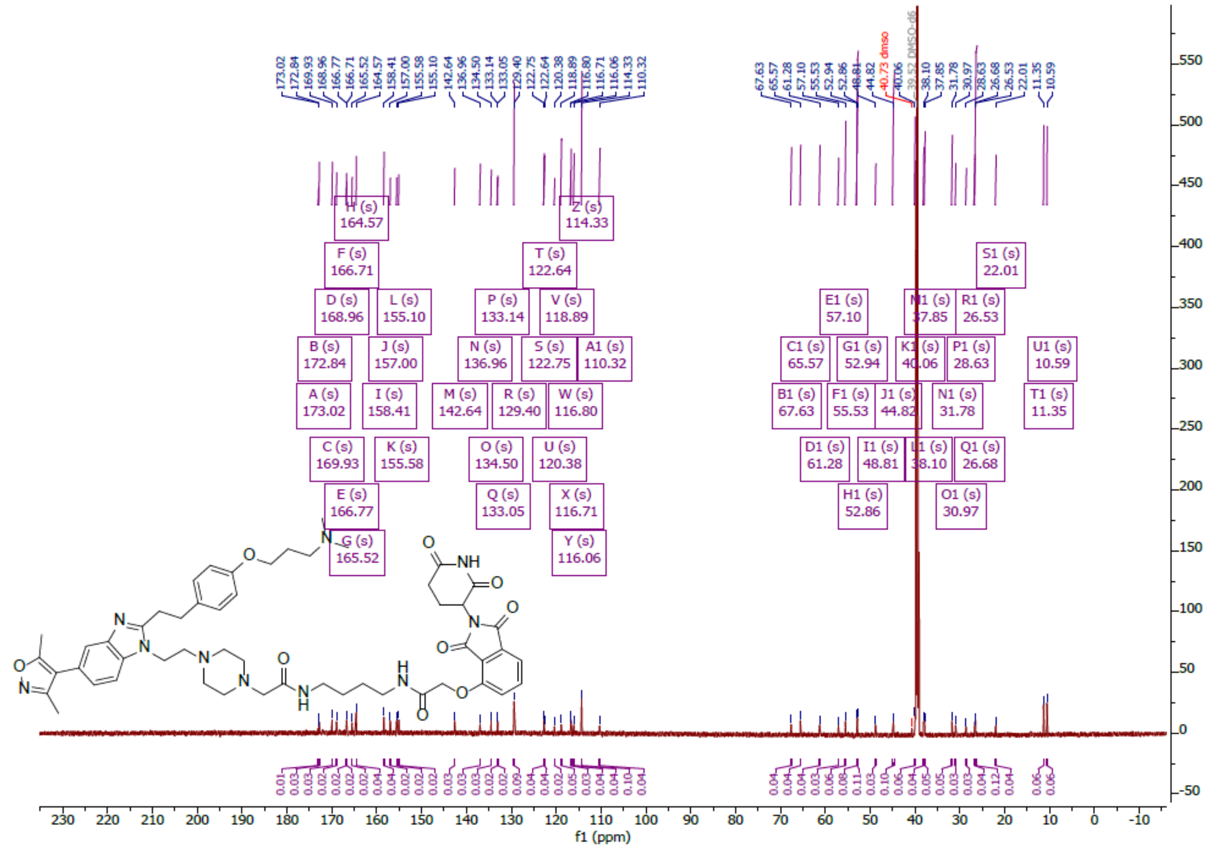

^{13}C NMR for **39**.

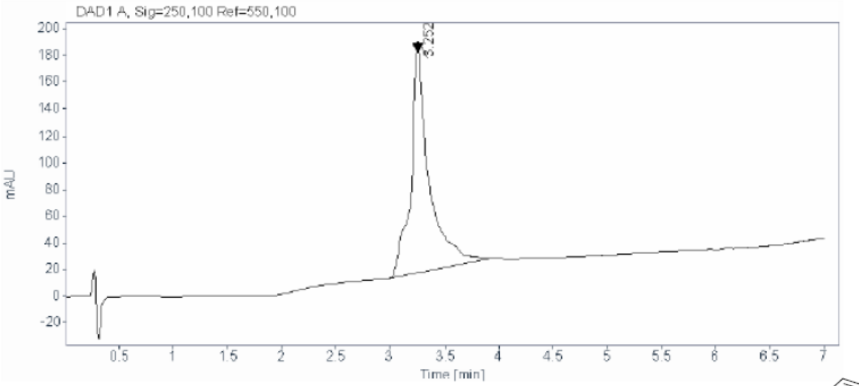

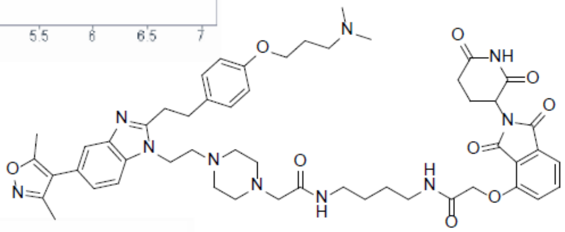

Signal: DAD1 A, Sig=250,100 Ref=550,100

RT [min]	Width [min]	Area	Height	Area%
3.252	0.15	1869.27	165.59	100.00

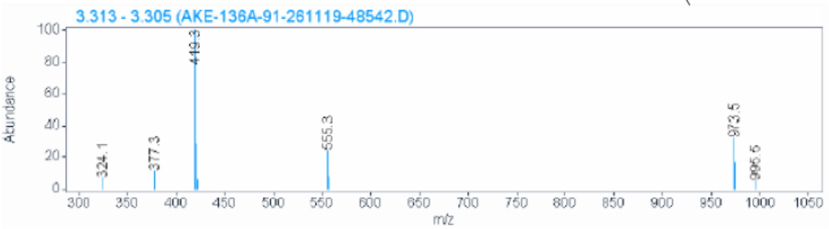

Monoisotopic Mass, Even Electron Ions
2 formula(e) evaluated with 1 results within limits (up to 50 closest results for each mass)
Elements Used:
C: 52-52 H: 0-80 N: 10-10 O: 9-9 Na: 0-1
AEK-136
AEDMONDS116124 351 (6.821)

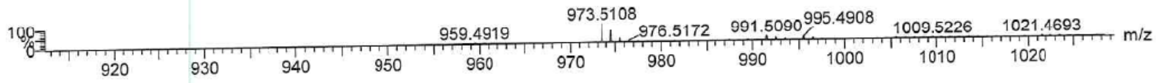

	Mass	Calc. Mass	mDa	PPM	DBE	Formula
Minimum:					-1.5	
Maximum:			5.0	30.0	50.0	
	973.4917	973.4936	-1.9	-2.0	25.5	C52 H65 N10 O9

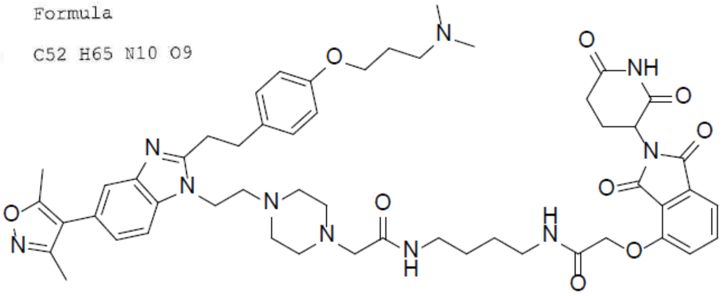

Molecular Weight: 973.13

LCMS/HRMS for **39**.

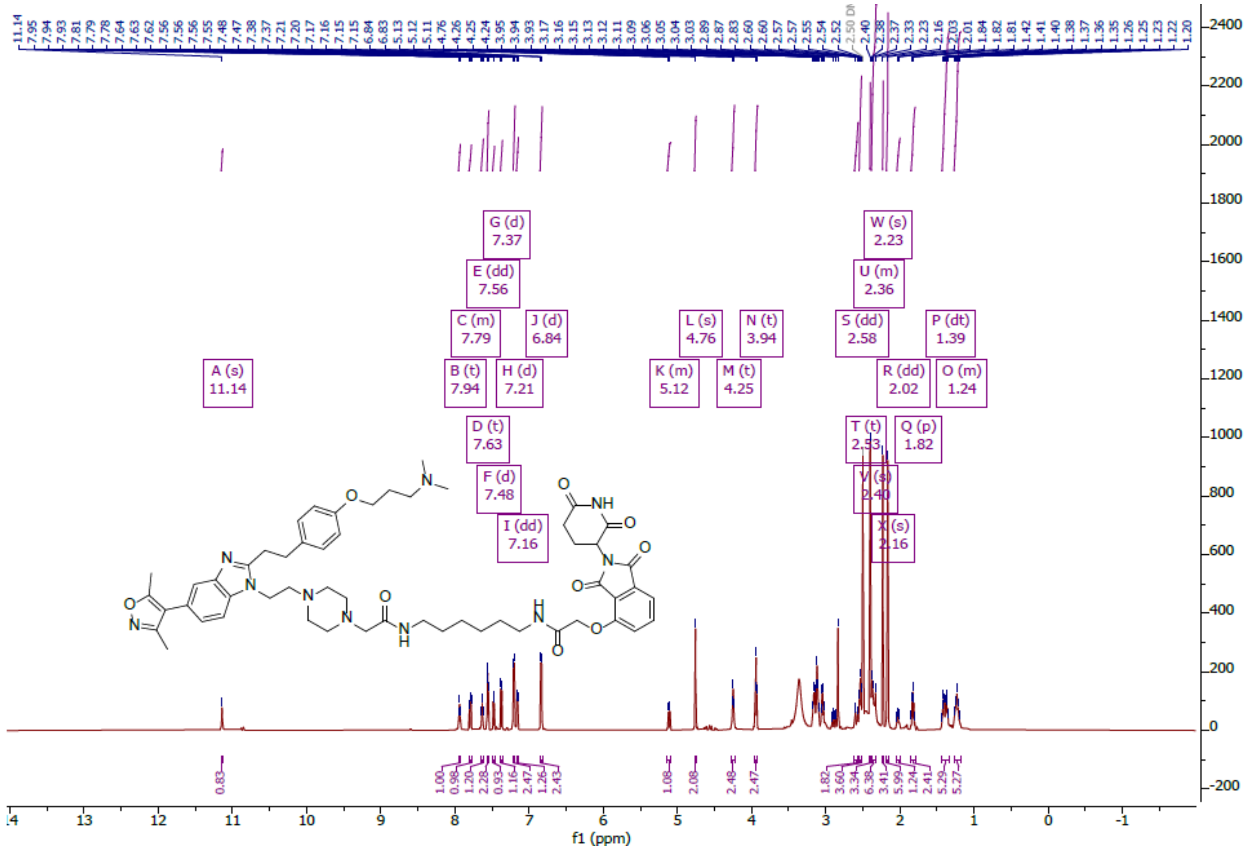

^{1}H NMR spectrum for **40**.

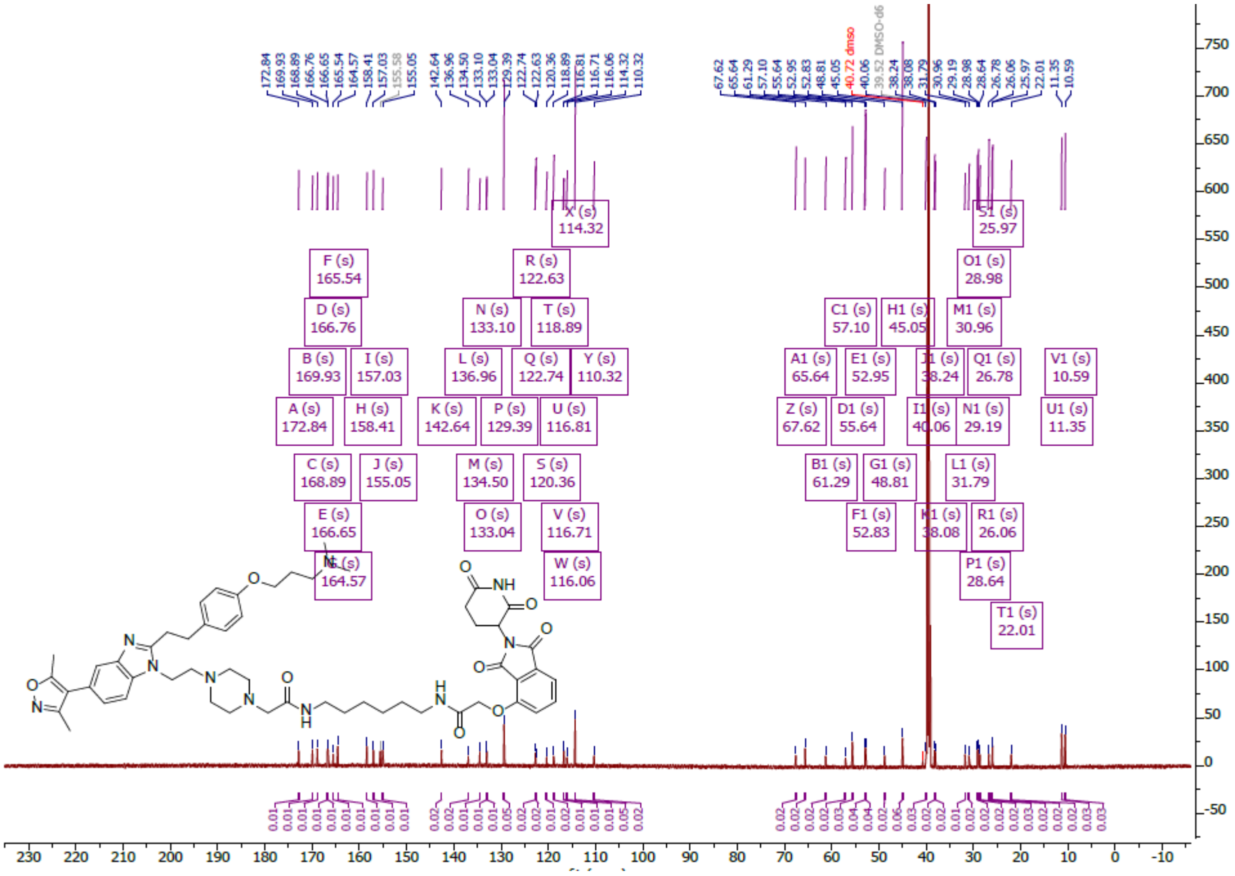

^{13}C NMR for **40**.

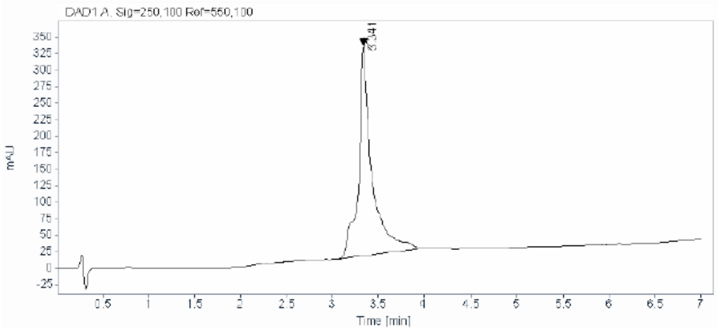

Signal: DAD1 A, Sig=250,100 Ref=550,100

RT [min]	Width [min]	Area	Height	Area%
3.341	0.14	3261.46	318.97	100.00

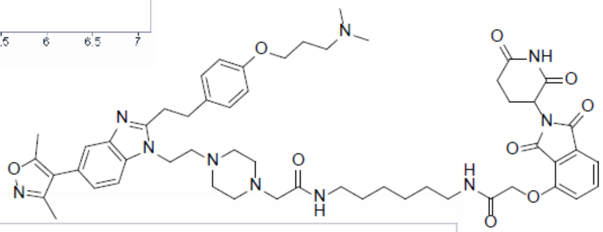

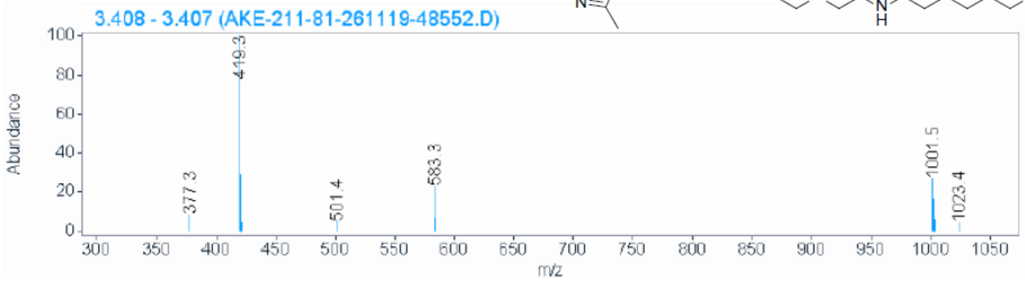

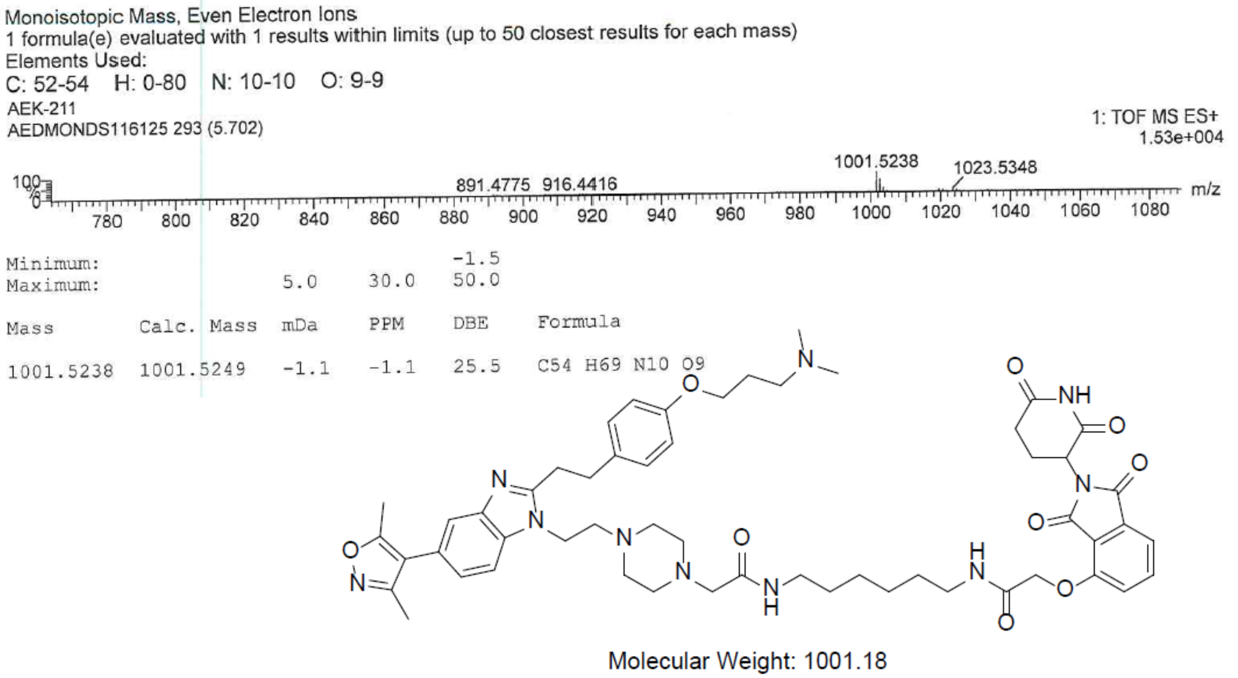

LCMS/HRMS for **40**.

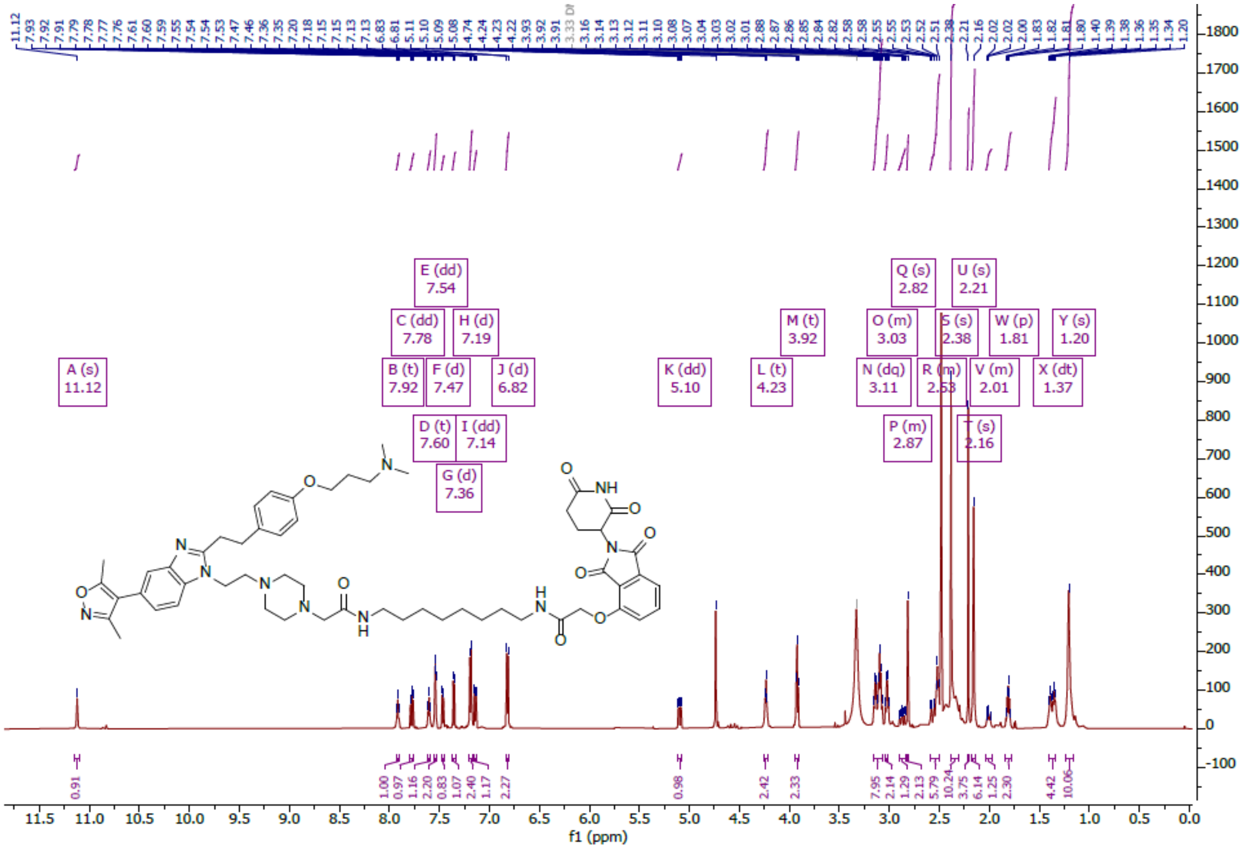

^{1}H NMR spectrum for **41**.

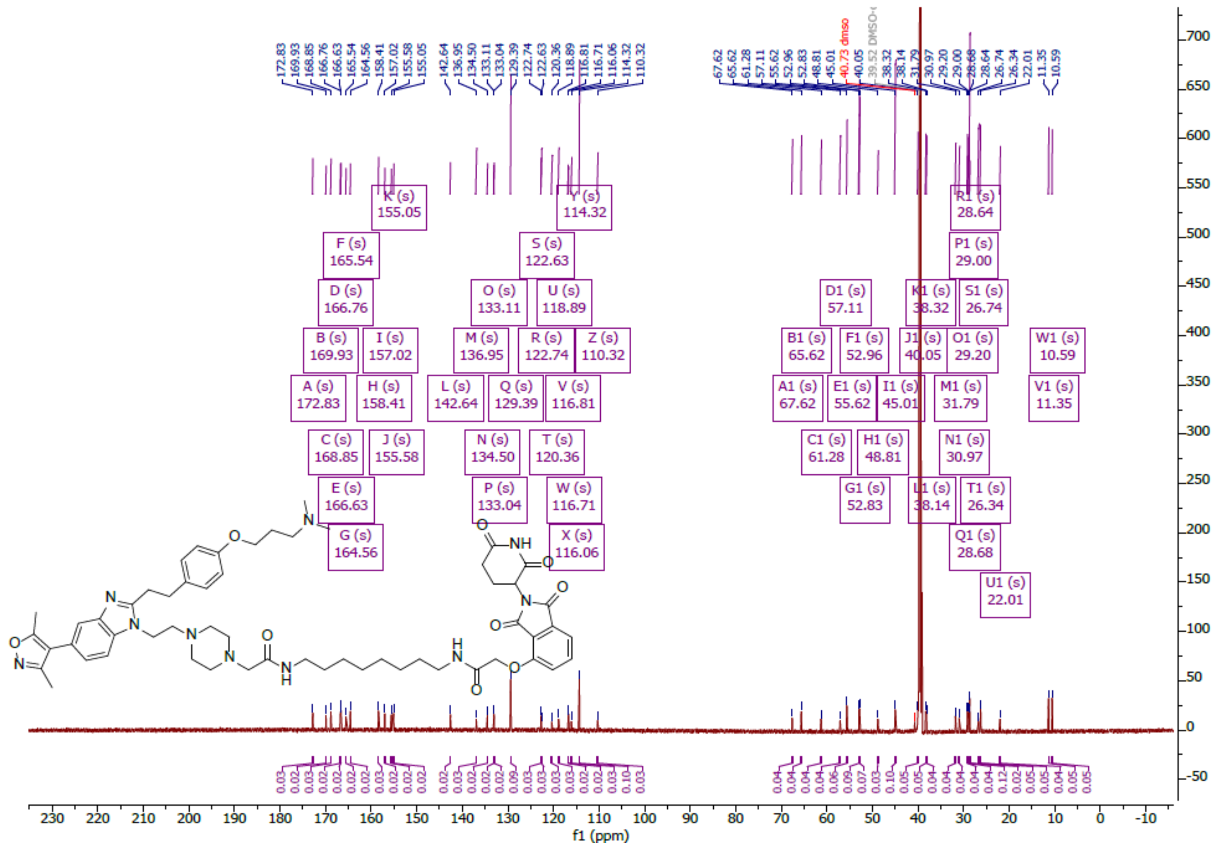

^{13}C NMR for **41**.

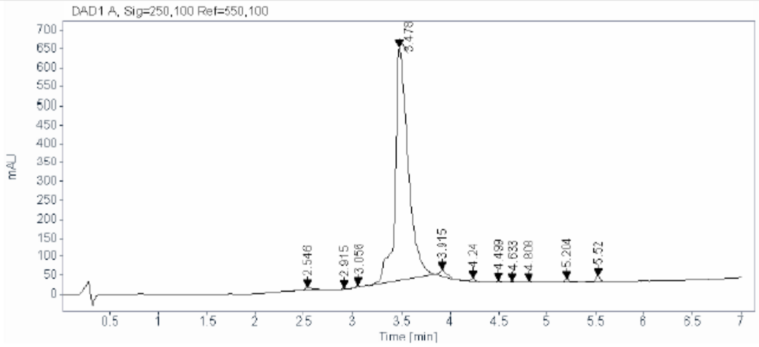

Signal: DAD1 A, Sig=250,100 Ref=550,100

RT [min]	Width [min]	Area	Height	Area%
2.546	0.09	60.96	9.34	1.02
2.915	0.04	7.18	2.66	0.12
3.056	0.04	13.72	4.88	0.23
3.478	0.14	5661.88	619.29	95.19
3.915	0.07	86.25	16.20	1.45
4.240	0.05	27.76	7.92	0.47
4.499	0.05	13.77	3.89	0.23
4.633	0.04	5.85	2.31	0.10
4.808	0.04	5.06	2.21	0.09
5.204	0.05	21.39	7.55	0.36
5.520	0.05	44.01	14.42	0.74

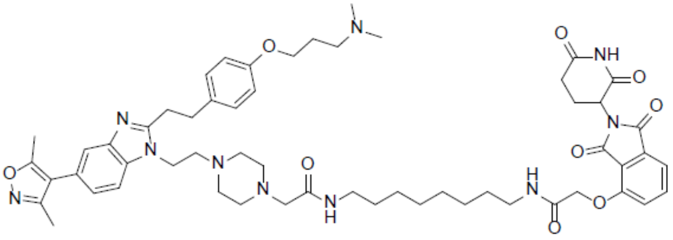

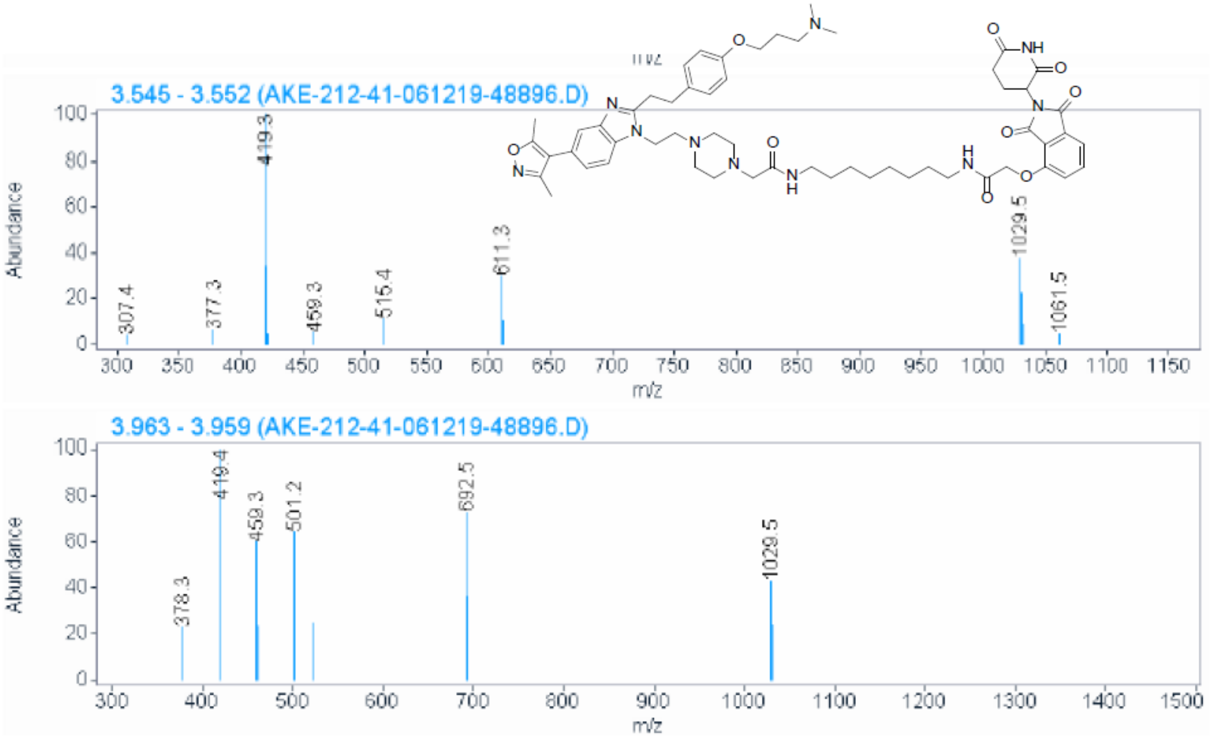

Monoisotopic Mass, Even Electron Ions
1 formula(e) evaluated with 1 results within limits (up to 50 closest results for each mass)
Elements Used:
C: 56-56 H: 0-80 N: 10-10 O: 9-9
AEK-212
AEDMONDS116126 310 (6.029)

1: TOF MS ES+
4.57e+004

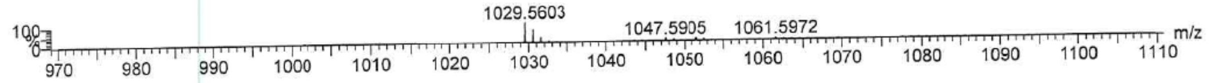

Minimum:				-1.5	
Maximum:		5.0	30.0	50.0	
Mass	Calc. Mass	mDa	PPM	DBE	Formula
1029.5603	1029.5562	4.1	4.0	25.5	C56 H73 N10 O9

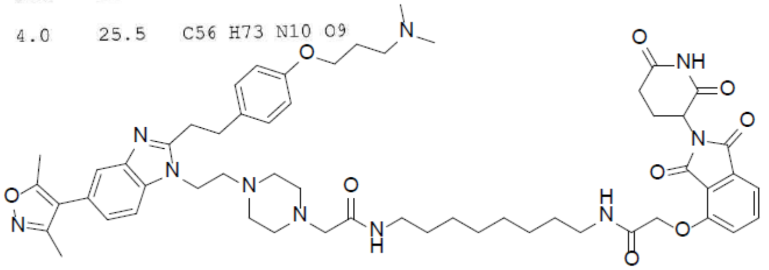

Molecular Weight: 1029.23

LCMS/HRMS for **41**.

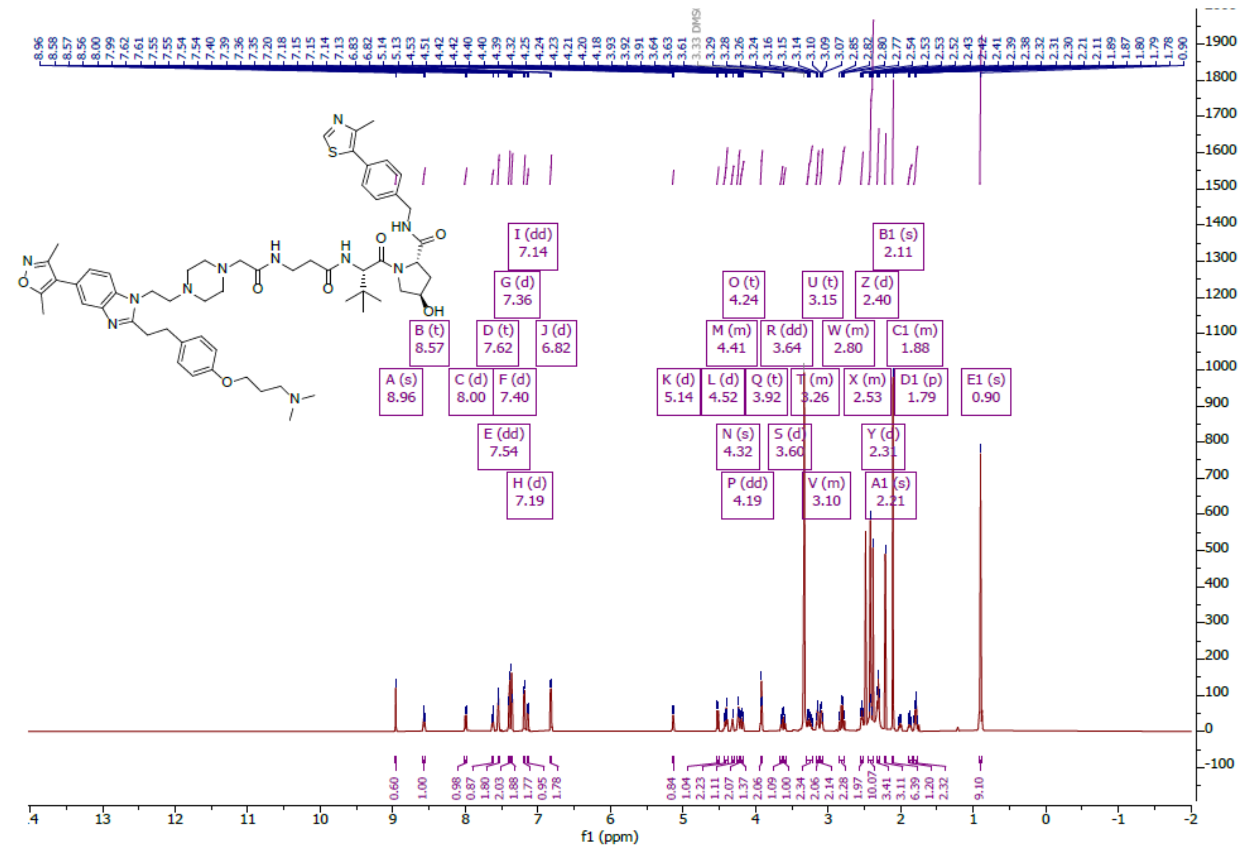

^{1}H NMR spectrum for **42**.

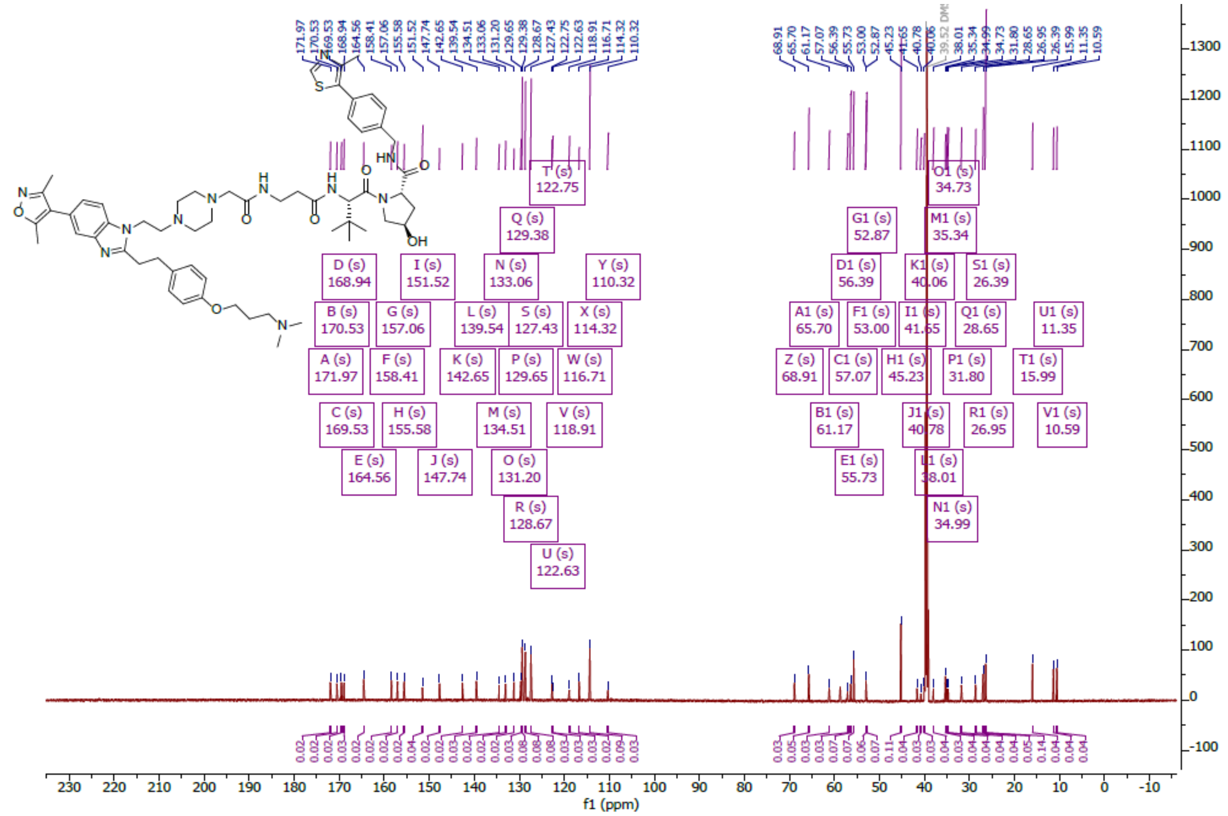

^{13}C NMR for **42**.

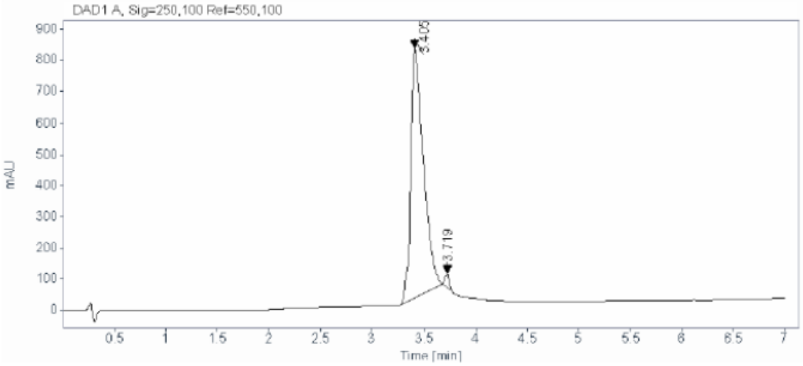

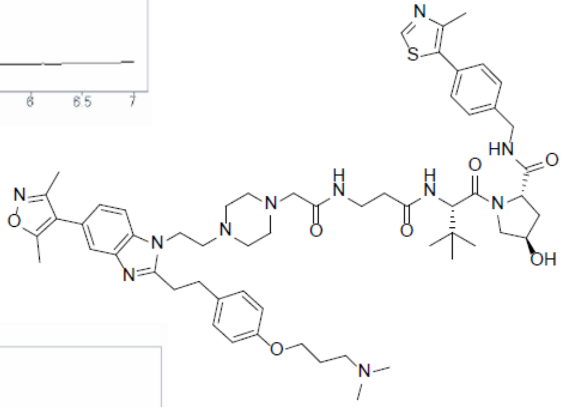

Signal: DAD1 A, Sig=250,100 Ref=550,100

RT [min]	Width [min]	Area	Height	Area%
3.405	0.12	6773.30	801.59	98.12
3.719	0.04	130.04	46.96	1.88

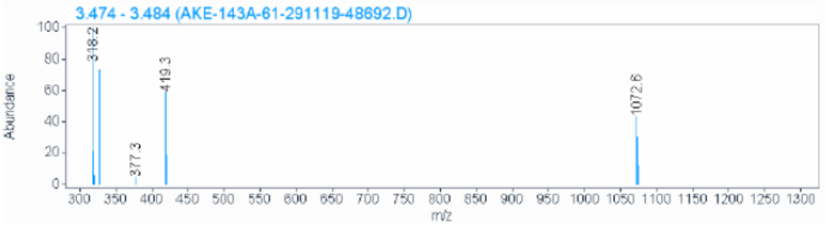

Monoisotopic Mass, Even Electron Ions
1 formula(e) evaluated with 1 results within limits (up to 50 closest results for each mass)
Elements Used:
C: 58-58 H: 0-80 N: 11-11 O: 7-7 S: 1-1
AEK-127
AEDMONDS116127 323 (6.288)
1: TOF MS ES+
6.59e+003

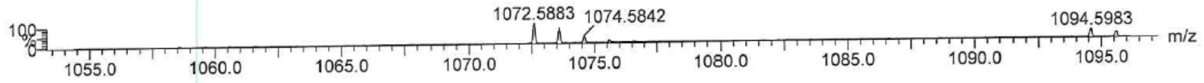

Minimum:				-1.5	
Maximum:		5.0	30.0	50.0	
Mass	Calc. Mass	mDa	PPM	DBE	Formula
1072.5815	1072.5806	0.9	0.8	25.5	C58 H78 N11 O7 S

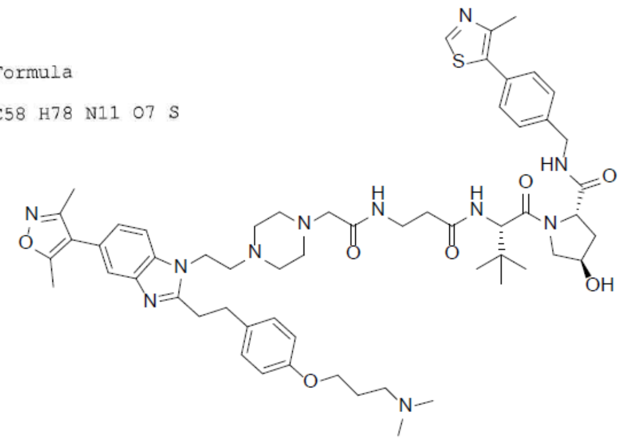

Molecular Weight: 1072.37

LCMS/HRMS for **42**.

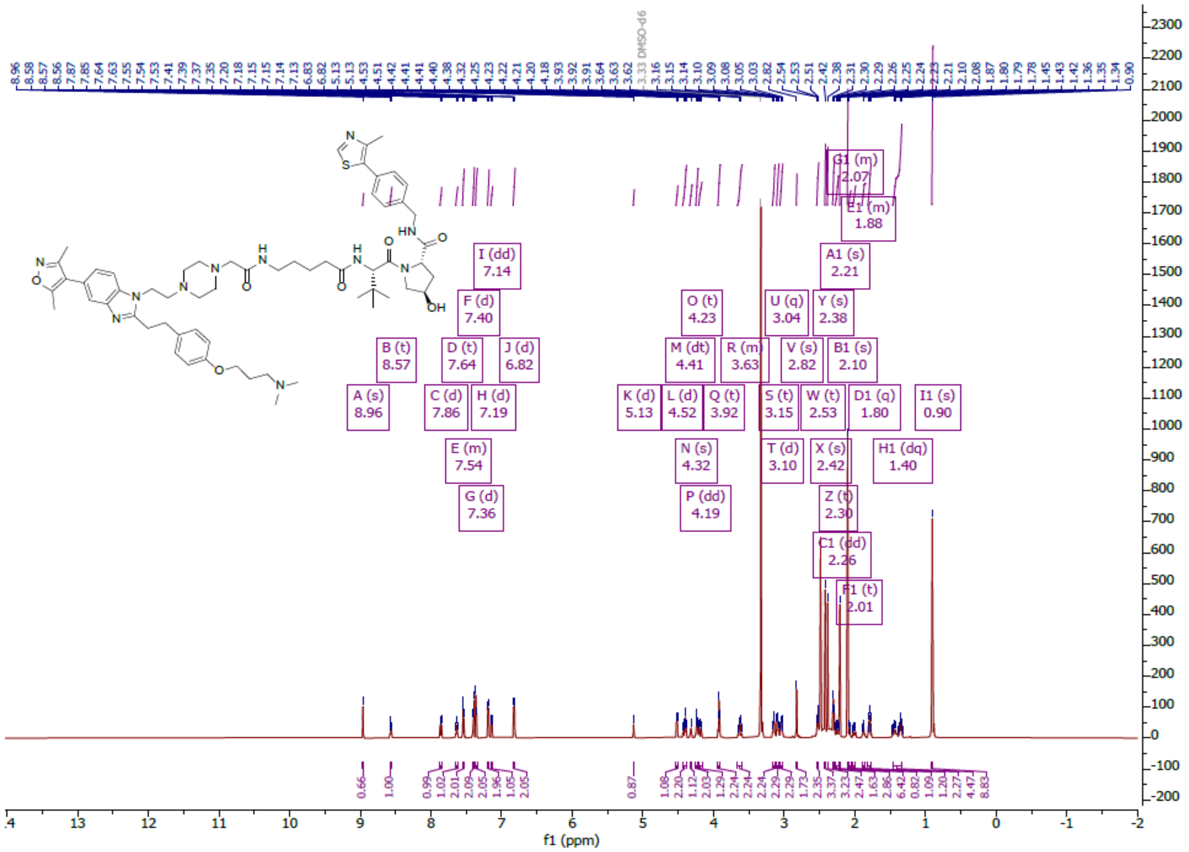

^{1}H NMR spectrum for **43**.

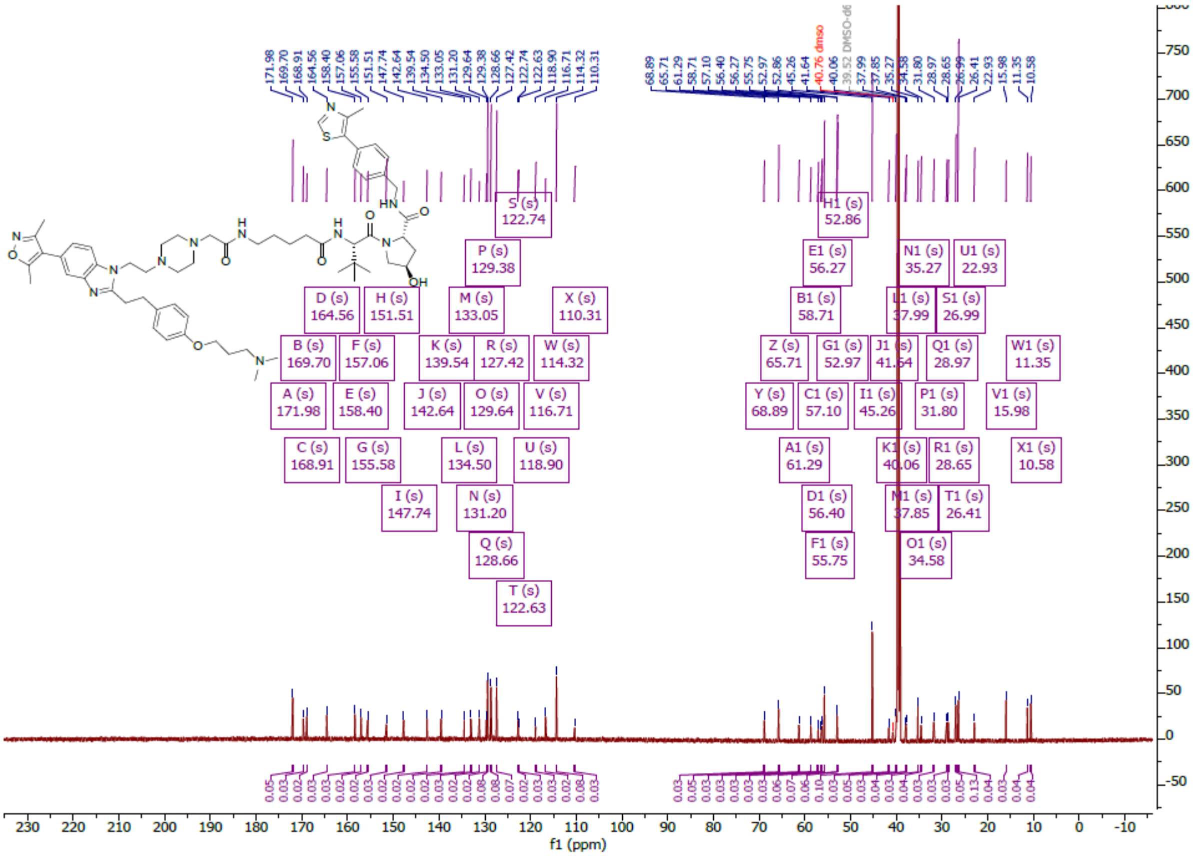

^{13}C NMR for **43**.

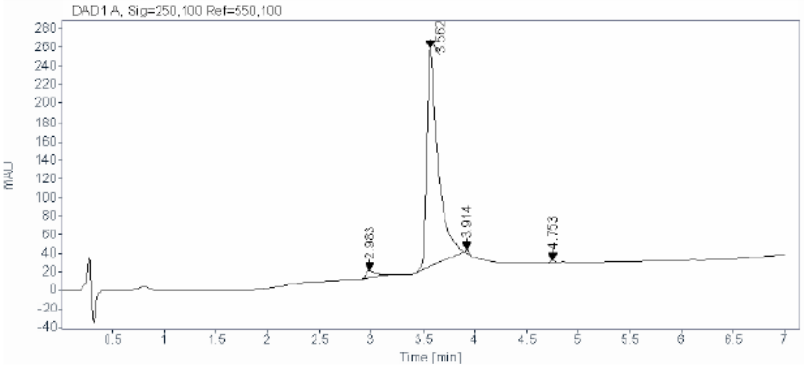

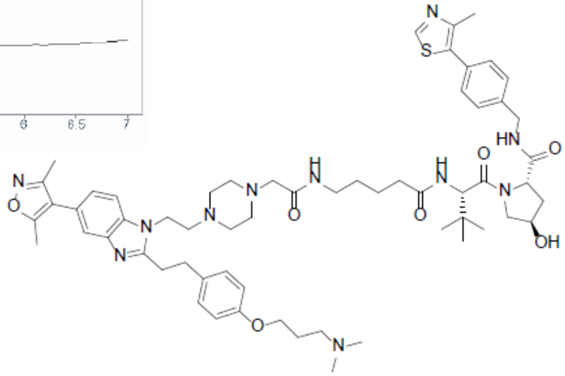

Signal: DAD1 A, Sig=250,100 Ref=550,100

RT [min]	Width [min]	Area	Height	Area%
2.983	0.10	53.05	7.55	2.93
3.562	0.10	1738.61	233.65	96.12
3.914	0.05	8.48	3.12	0.47
4.753	0.05	8.66	2.95	0.48

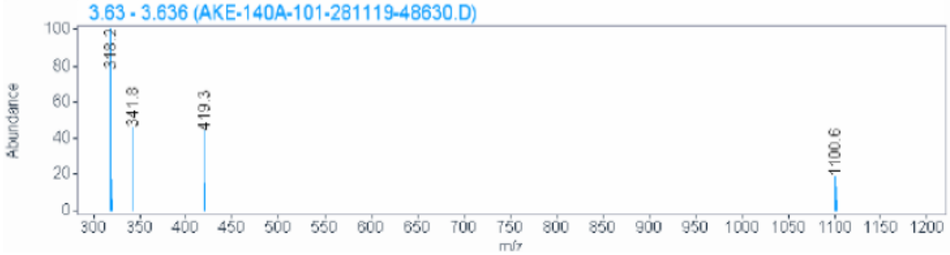

Monoisotopic Mass, Even Electron Ions
3 formula(e) evaluated with 1 results within limits (up to 50 closest results for each mass)
Elements Used:
C: 60-62 H: 0-100 N: 11-11 O: 7-7 S: 1-1
AEK-140
AEDMONDS116128 269 (5.237)

1: TOF MS ES+
4.49e+003

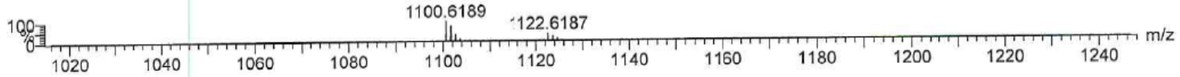

	Mass	Calc. Mass	mDa	PPM	DBE	Formula
Minimum:					-1.5	
Maximum:			5.0	30.0	50.0	
	1100.6189	1100.6119	7.0	6.4	25.5	C60 H82 N11 O7 S

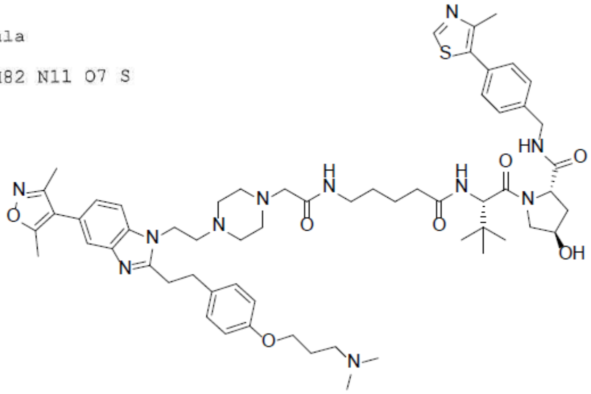

Molecular Weight: 1100.42

LCMS/HRMS for **43**.

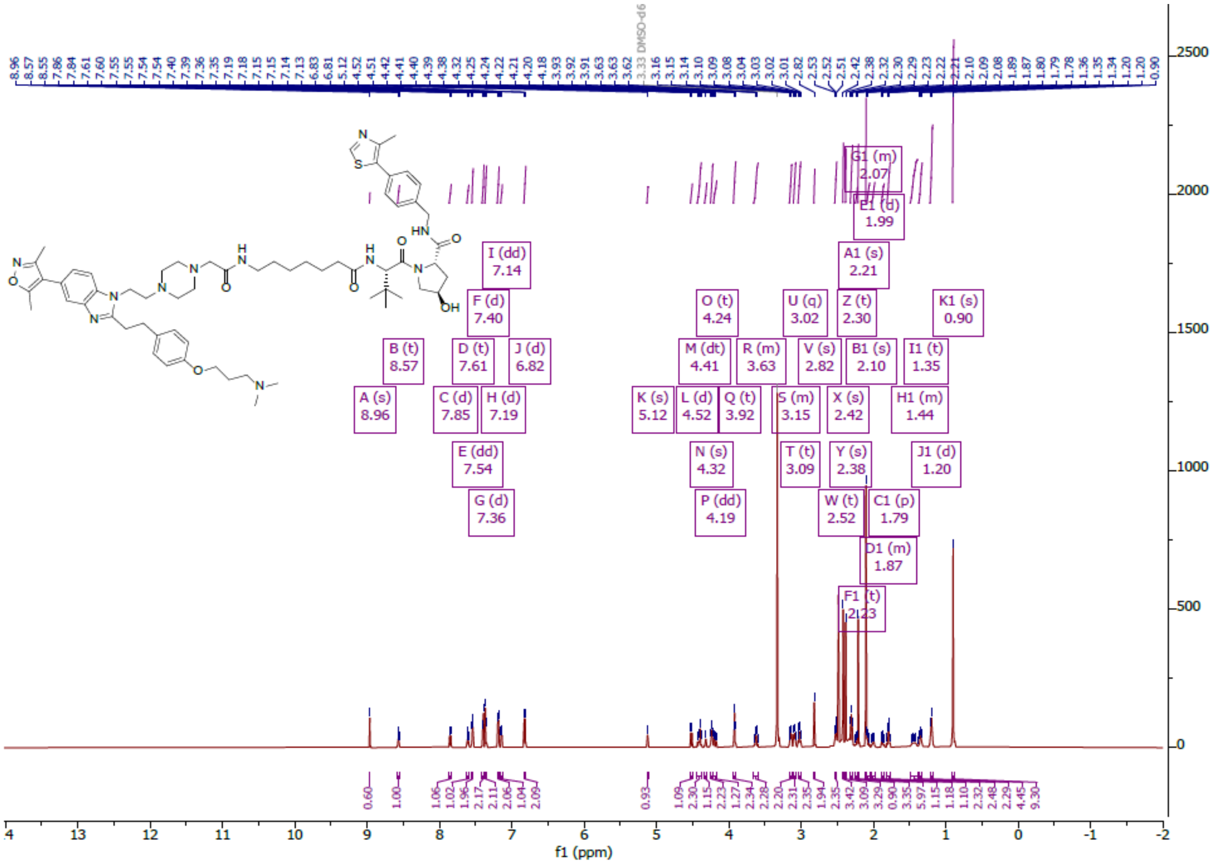

^{1}H NMR spectrum for **44**.

Compound **4.71**

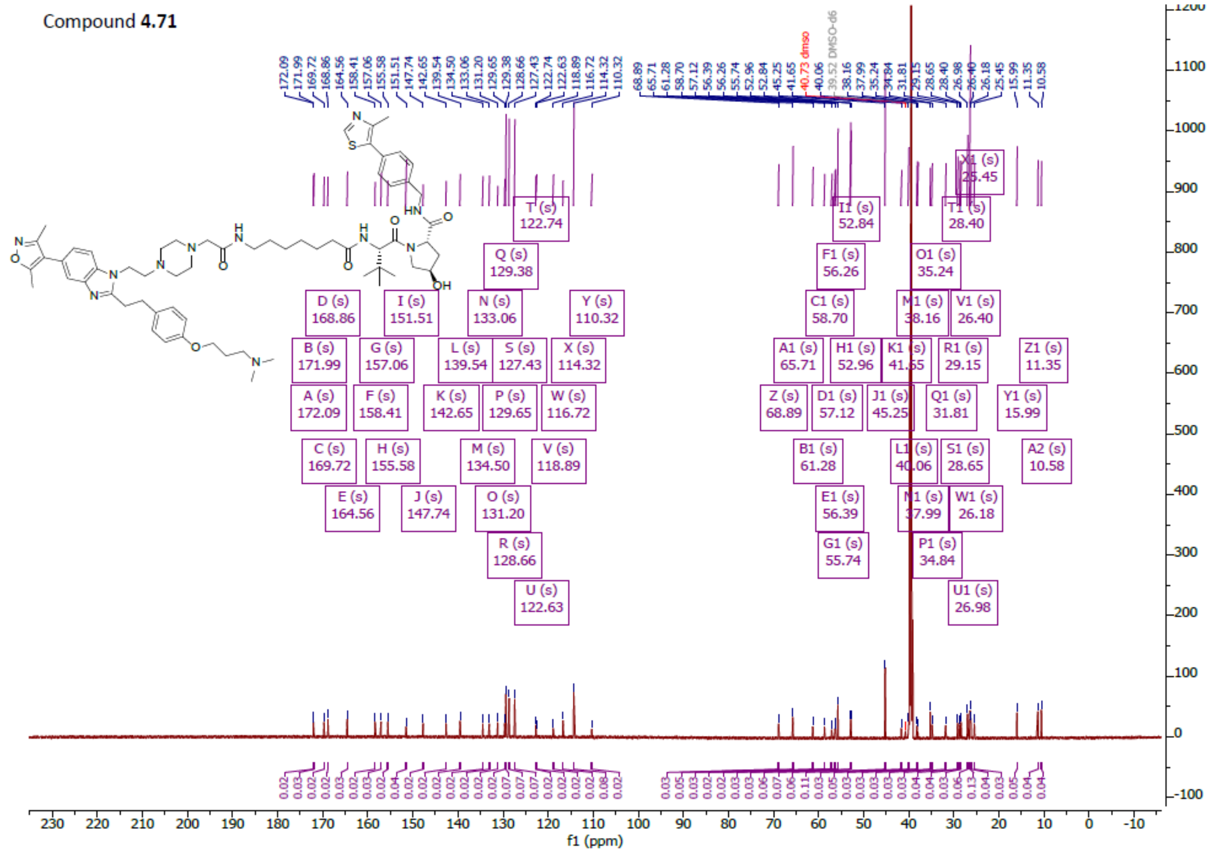

^{13}C NMR for **44**.

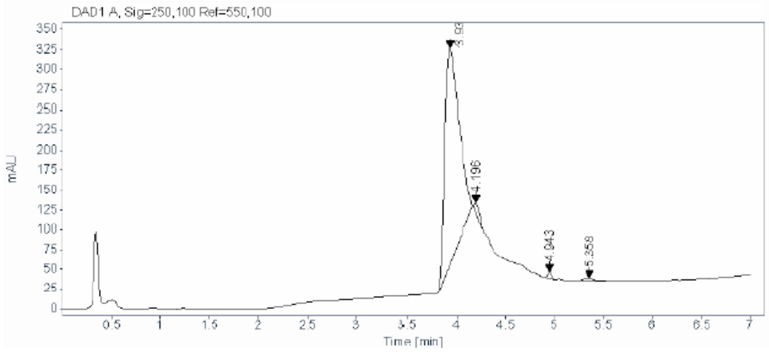

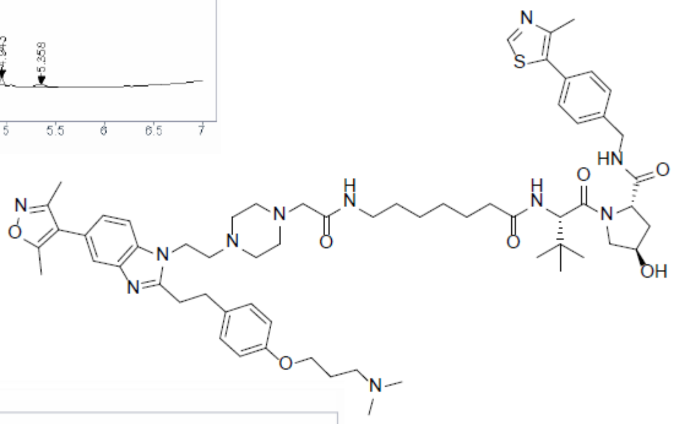

Signal: DAD1 A, Sig=250,100 Ref=550,100

RT [min]	Width [min]	Area	Height	Area%
3.930	0.15	2714.42	271.79	96.88
4.196	0.07	46.97	11.44	1.68
4.943	0.04	20.42	7.85	0.73
5.358	0.09	20.03	3.06	0.71

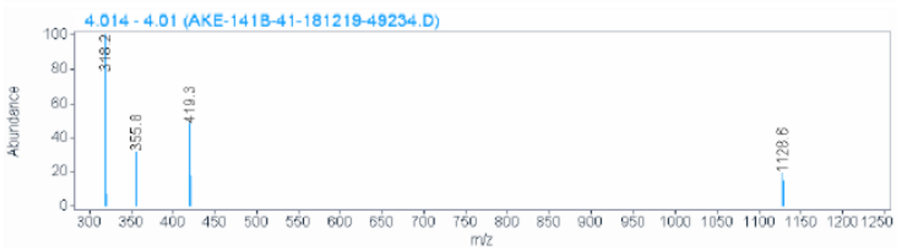

Single Mass Analysis
Tolerance = 30.0 PPM / DBE: min = -1.5, max = 50.0 4.71
Element prediction: Off

Monoisotopic Mass, Even Electron Ions
2 formula(e) evaluated with 1 results within limits (up to 50 closest results for each mass)
Elements Used:
C: 60-62 H: 0-100 N: 11-11 O: 7-7 S: 1-1
AEK-141
AEDMONDS116129 259 (5.048)

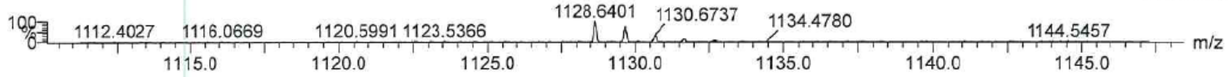

Minimum:				-1.5	
Maximum:		5.0	30.0	50.0	
Mass	Calc. Mass	mDa	PPM	DBE	Formula
1128.6401	1128.6432	-3.1	-2.7	25.5	C62 H86 N11 O7 S

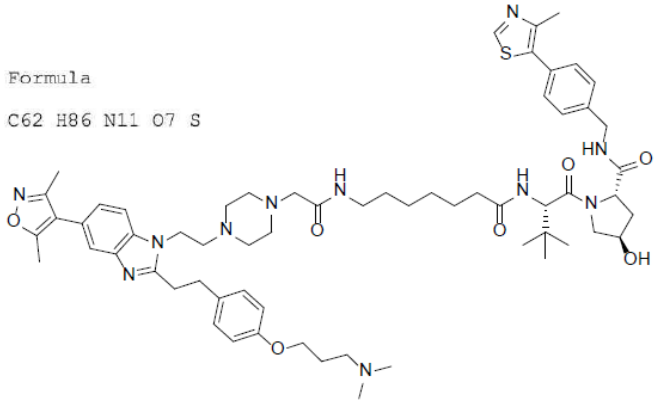

Molecular Weight: 1128.47

LCMS/HRMS for **44**.

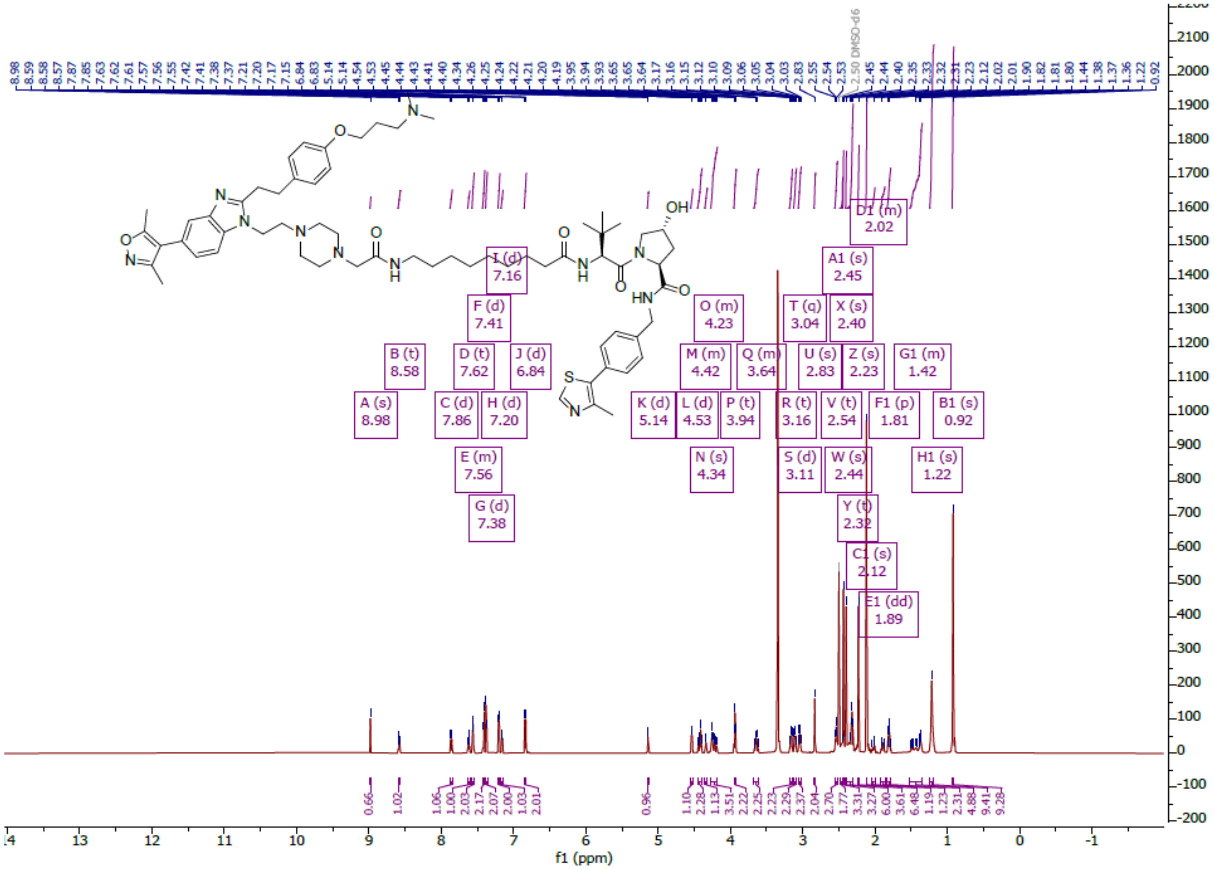

^{1}H NMR spectrum for **45**.

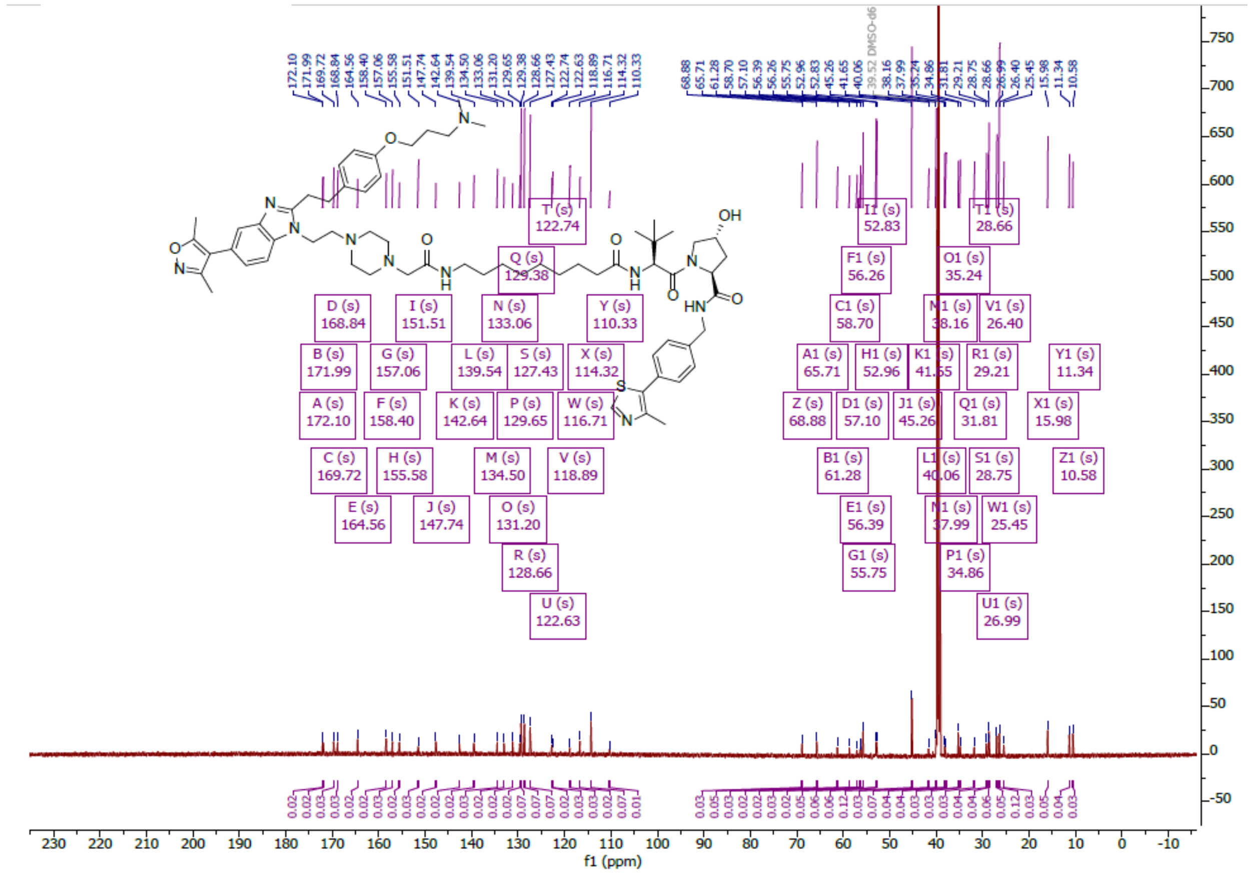

^{13}C NMR for **45**.

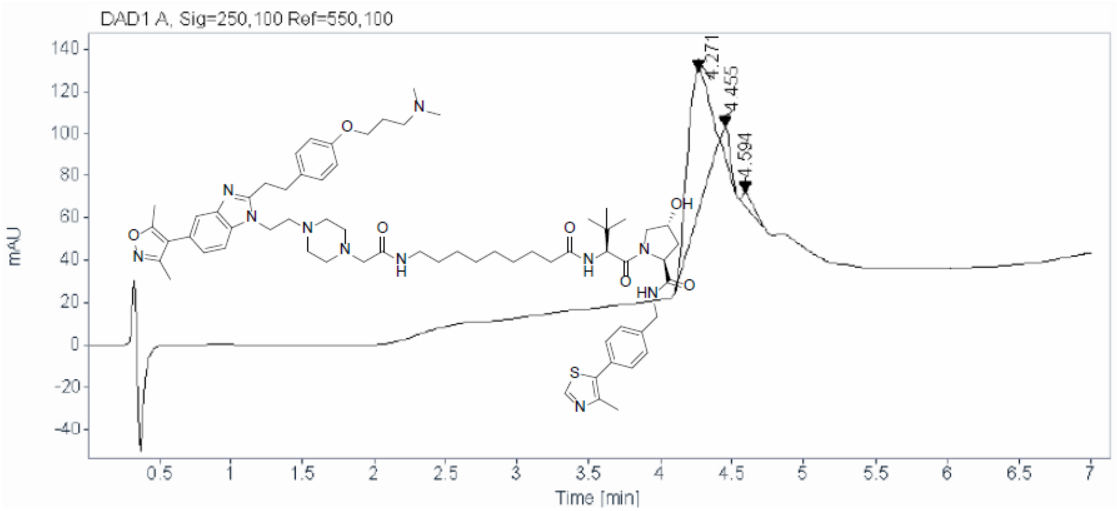

Signal:	DAD1 A, Sig=250,100 Ref=550,100			
RT [min]	Width [min]	Area	Height	Area%
4.271	0.18	671.59	65.10	87.45
4.455	0.06	55.29	14.22	7.20
4.594	0.11	41.10	6.03	5.35

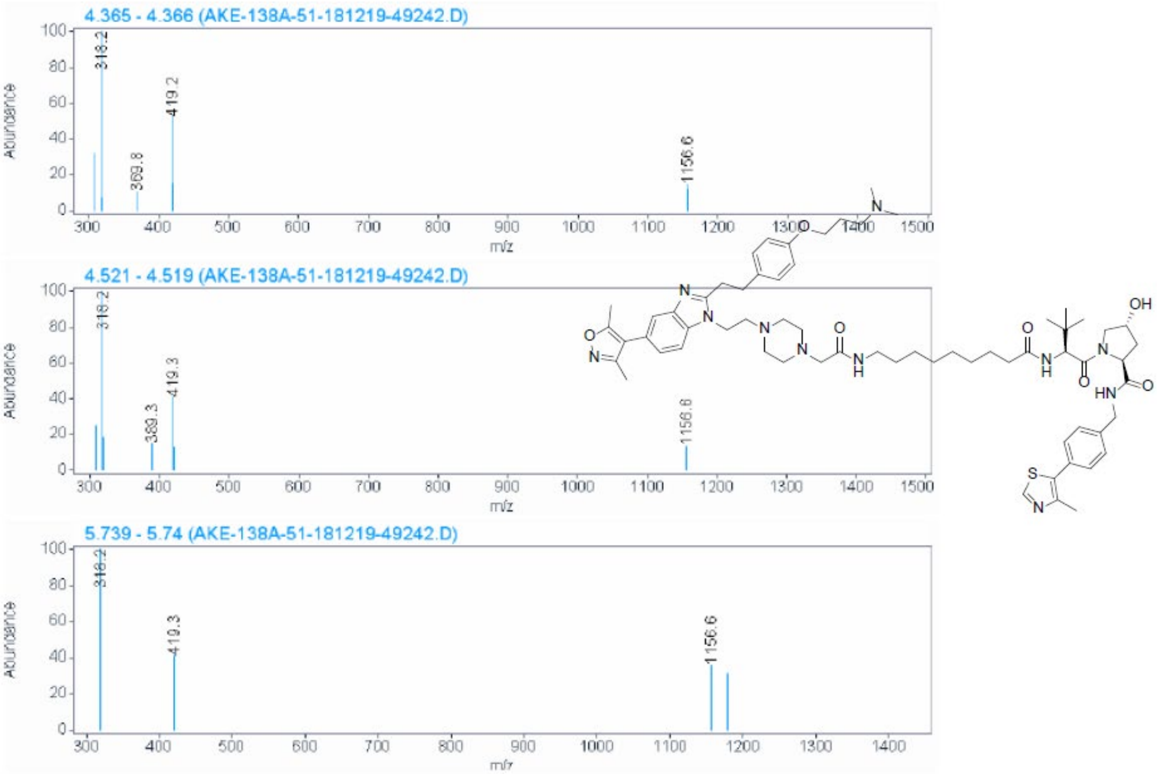

Monoisotopic Mass, Even Electron Ions
1 formula(e) evaluated with 1 results within limits (up to 50 closest results for each mass)
Elements Used:
C: 60-64 H: 0-100 N: 11-11 O: 7-7 S: 1-1
AEK-138
AEDMONDS116130 242 (4.721)

1: TOF MS ES+
5.65e+002

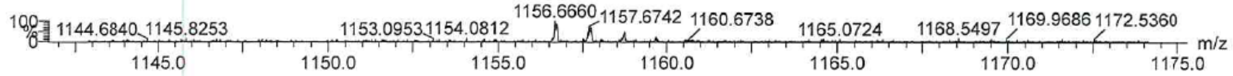

Minimum:				-1.5	
Maximum:		5.0	30.0	50.0	
Mass	Calc. Mass	mDa	PPM	DBE	Formula
1156.6660	1156.6745	-8.5	-7.3	25.5	C64 H90 N11 O7 S

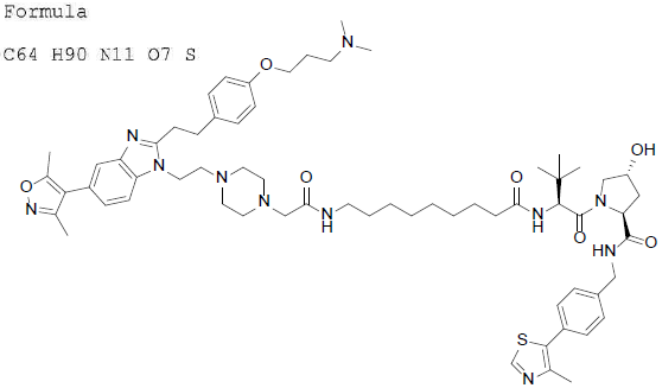

Molecular Weight: 1156.53

LCMS/HRMS for **45**.
